# Exploring Novel GSK-3β Inhibitors for Anti-Neuroinflammatory and Neuroprotective Effects: Synthesis, Crystallography, Computational Analysis, and Biological Evaluation

**DOI:** 10.1021/acschemneuro.4c00365

**Published:** 2024-08-19

**Authors:** Izabella Góral, Tomasz Wichur, Emilia Sługocka, Przemysław Grygier, Monika Głuch-Lutwin, Barbara Mordyl, Ewelina Honkisz-Orzechowska, Natalia Szałaj, Justyna Godyń, Dawid Panek, Paula Zaręba, Anna Sarka, Paweł Żmudzki, Gniewomir Latacz, Katarzyna Pustelny, Adam Bucki, Anna Czarna, Filipe Menezes, Anna Więckowska

**Affiliations:** † Department of Physicochemical Drug Analysis, Faculty of Pharmacy, Jagiellonian University Medical College, 9 Medyczna St., Krakow 30-688, Poland; ‡ Doctoral School of Medical and Health Sciences, Jagiellonian University Medical College, 16 Lazarza St., Krakow 31-530, Poland; § Malopolska Centre of Biotechnology, Jagiellonian University, Gronostajowa 7a, Krakow 30-387, Poland; ∥ Doctoral School of Exact and Natural Sciences, Jagiellonian University, Lojasiewicza 11, Krakow 30-348, Poland; ⊥ Department of Pharmacobiology, Faculty of Pharmacy, Jagiellonian University Medical College, 9 Medyczna St., Krakow 30-688, Poland; # Department of Technology and Biotechnology of Drugs, Faculty of Pharmacy, Jagiellonian University Medical College, 9 Medyczna St., Krakow 30-688, Poland; ∇ Department of Medicinal Chemistry, Faculty of Pharmacy, Jagiellonian University Medical College, 9 Medyczna St., Krakow 30-688, Poland; ○ Department of Physical Biochemistry, Faculty of Biochemistry, Biophysics and Biotechnology, Jagiellonian University, Gronostajowa 7 St., Krakow 30-387, Poland; ◆ Helmholtz Munich, Molecular Targets and Therapeutics Center, Institute of Structural Biology, Ingolstädter Landstr. 1, Neuherberg 85764, Germany

**Keywords:** glycogen synthase kinase-3β, Alzheimer’s disease, crystallography, neurodegeneration, anti-inflammatory activity, ADME, EDDA

## Abstract

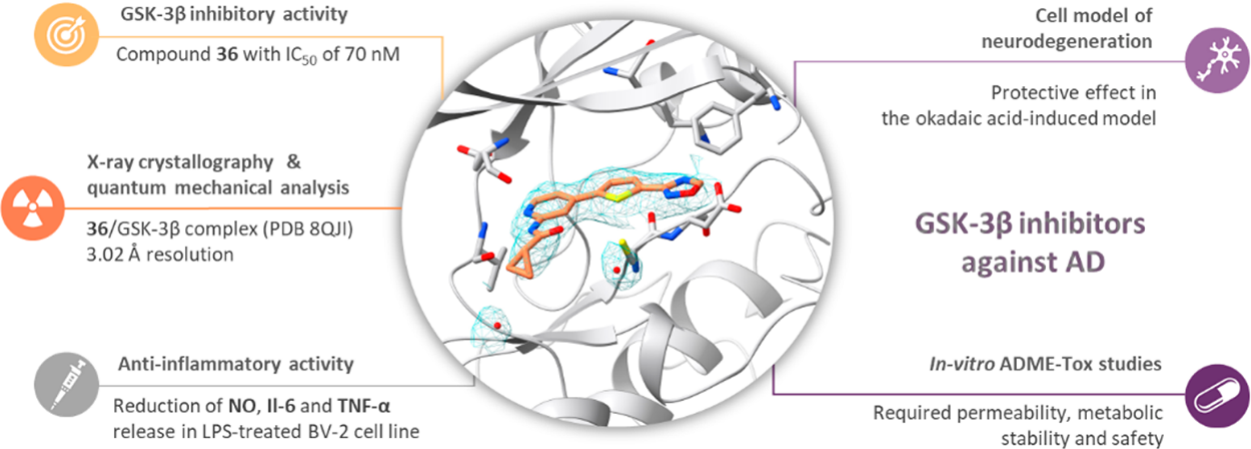

In the pathogenesis of Alzheimer’s disease, the overexpression of glycogen synthase kinase-3β (GSK-3β) stands out due to its multifaced nature, as it contributes to the promotion of amyloid β and tau protein accumulation, as well as neuroinflammatory processes. Therefore, in the present study, we have designed, synthesized, and evaluated a new series of GSK-3β inhibitors based on the *N*-(pyridin-2-yl)cyclopropanecarboxamide scaffold. We identified compound **36**, demonstrating an IC_50_ of 70 nM against GSK-3β. Subsequently, through crystallography studies and quantum mechanical analysis, we elucidated its binding mode and identified the structural features crucial for interactions with the active site of GSK-3β, thereby understanding its inhibitory potency. Compound **36** was effective in the cellular model of hyperphosphorylated tau-induced neurodegeneration, where it restored cell viability after okadaic acid treatment and showed anti-inflammatory activity in the LPS model, significantly reducing NO, IL-6, and TNF-α release. In ADME-tox in vitro studies, we confirmed the beneficial profile of **36,** including high permeability in PAMPA (*Pe* equals 9.4) and high metabolic stability in HLMs as well as lack of significant interactions with isoforms of the CYP enzymes and lack of considerable cytotoxicity on selected cell lines (IC_50_ > 100 μM on HT-22 cells and 89.3 μM on BV-2 cells). Based on promising pharmacological activities and favorable ADME-tox properties, compound **36** may be considered a promising candidate for in vivo research as well as constitute a reliable starting point for further studies.

## Introduction

Alzheimer’s disease (AD) is among the top 10 leading causes of death globally, and due to unfavorable demographic trends, it is becoming an increasingly significant sociological and economic burden.^1,2^ It is the most common form of dementia characterized by progressive cognitive decline accompanied by behavioral and memory impairments gradually leading to loss of independence in everyday functioning and the need for constant care.^3^ The complexity of Alzheimer’s disease hinders its treatment, as well as the search for new therapeutic solutions. Currently, accessible first-line anti-AD drugs, cholinesterase inhibitors and the NMDA receptor antagonist memantine are aimed at reducing symptoms and only allow for temporary compensation of impaired cognitive functions.^4,5^

From a molecular perspective, AD is characterized by misfolding and accumulation of β-amyloid peptide (Aβ) and tau protein with accompanying neuroinflammation and loss of neurons.^6^ According to the amyloid cascade hypothesis, the accumulation of various forms of Aβ in the brain is the primary cause that leads to the formation of intracellular neurofibrillary tangles (NFTs) and, consequently, neuronal death.^7^ This has for decades fueled interest in Aβ as a biological target in the search for an effective AD therapy and has recently led to the approval of two monoclonal antibodies targeting amyloid-β deposits: aducanumab and lecanemab. Even though they certainly constitute a breakthrough in the treatment of the disease, their effectiveness is still under scrutiny, they are burdened with severe side effects and a considerable cost of treatment.^8−10^ Therefore, the development of a small-molecule drug targeting processes underlying AD is highly desirable.

Glycogen synthase kinase-3β (GSK-3β) is a serine/threonine kinase that is overexpressed in the brain of AD patients and contributes to the development of Alzheimer’s disease by the promotion of Aβ and tau protein aggregation and neuroinflammation as well as its involvement in memory and synaptic plasticity.^11−13^ It promotes Aβ plaque formation by stimulating the BACE1 enzyme to cleave amyloid precursor protein (APP) via the amyloidogenic pathway and by phosphorylation of APP, which affects neuronal excitability and impairs control of calcium homeostasis.^14,15^ GSK-3β abnormal activity leads to hyperphosphorylation of tau protein which results in the formation of neurofibrillary tangles and the loss of its fundamental property–stabilization of microtubules.^16,17^ NFT aggregation and microtubule disassembly are regarded as the major processes underlying the degeneration of neurons.^18^ It was demonstrated that inhibition of GSK-3β reduces phosphorylation of tau and the production of Aβ peptides and restores cognitive deficits in 3xTg, 5XFAD, and APP/PS1 double transgenic mice models.^19−22^

GSK-3β activity also plays a significant role in the modulation of immune response within the central nervous system (CNS).^23^ Its crucial role in regulating both pro- and anti-inflammatory cytokines in vivo was demonstrated in 2005, in a model using Toll-like receptor (TLR) agonists.^24^ Inhibition of GSK-3β led to a substantial reduction of pro-inflammatory IL-1β, IL-6, TNF-α, IL-12 and IFN-γ accompanied by a profound increase of anti-inflammatory IL-10.^25−28^ Notably, Aβ aggregates are among the factors capable of binding to and activating TLRs, which, in turn, mobilize microglia to produce reactive oxygen species (ROS), nitric oxide (NO) and pro-inflammatory cytokines.^29,30^ These events alter the permeability of the blood-brain barrier and induce lipid peroxidation and DNA damage, ultimately leading to the death of nervous cells.^31,32^ Also, it was demonstrated in animal models that the inhibition of GSK-3β facilitates the induction of long-term potentiation (LTP)—a fundamental component of synaptic plasticity.^33^ This connection elucidates the previously observed association between GSK-3β overexpression and spatial memory deficits, providing insight into the role of GSK-3β in learning and memory formation.^34^

Given the multifaceted role of GSK-3β in the processes contributing to the onset and progression of Alzheimer’s disease, it represents an excellent biological target in the pursuit of disease treatment. The interest in GSK-3β led to the discovery of numerous distinct classes of GSK-3β inhibitors.^35,36^ Certain GSK-3β inhibitors, such as tideglusib, effectively reduced brain levels of tau phosphorylation, amyloid deposition, neuronal cell death, and memory deficits in animal models of AD.^37,38^ While these inhibitors have progressed to phase II of clinical trials^39−41^ none have yet reached the market. In our ongoing quest for effective anti-Alzheimer’s therapy, we have designed, synthesized, and evaluated a new series of GSK-3β inhibitors in vitro and in cellulo*.* From this research, we have identified compound **36** as a promising lead candidate. We have characterized its biological activity and binding mode using crystallography data, along with a preliminary evaluation of its ADMET properties.

## Results and Discussion

### Design

**Figure 1:**
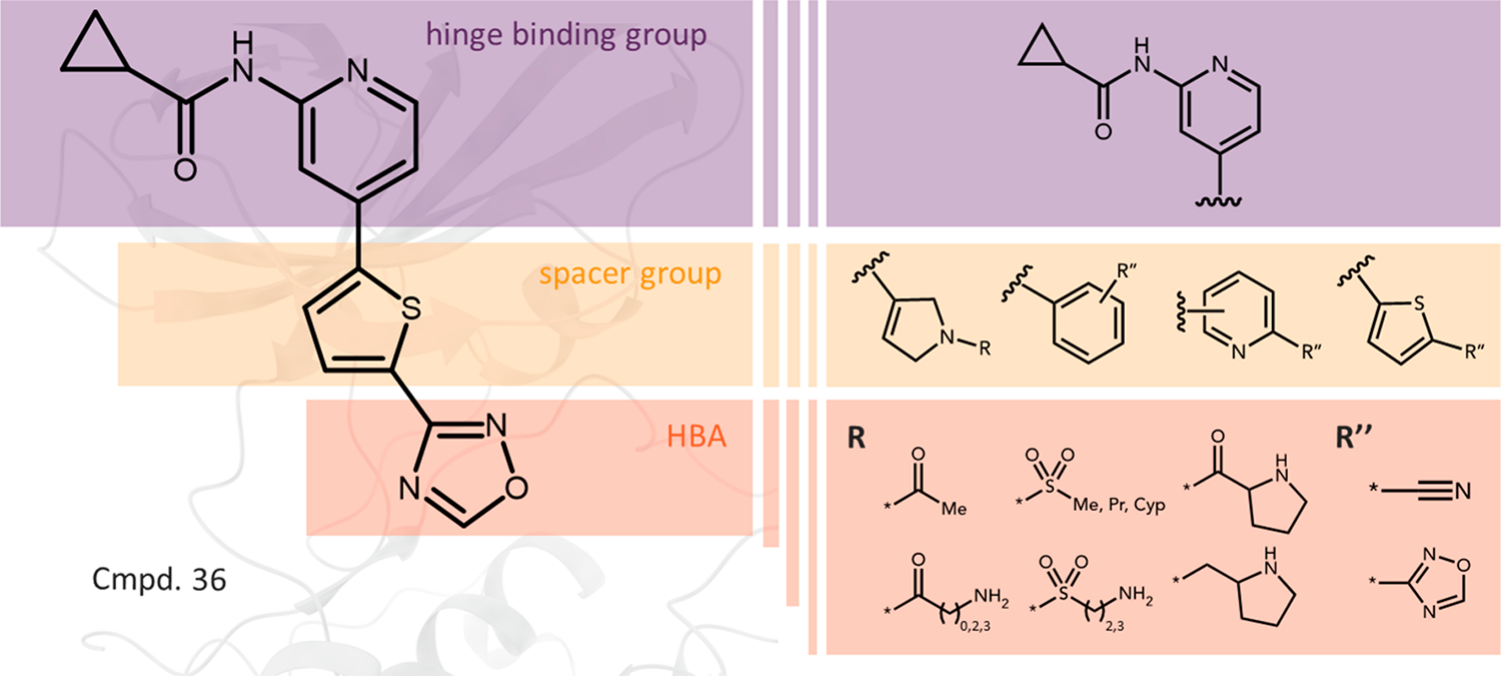
Design of new GSK-3β inhibitors highlighting structural elements contributing to the effective binding.

The design of novel GSK-3β inhibitors was inspired by the model proposed by Sivaprakasam et al.^42^ It comprises three structural elements contributing to the effective binding with GSK-3β: (1) the hinge binding group, (2) the spacer group, and (3) the hydrogen bond acceptor (HBA) complementary to Lys85, exemplified by compound **36** (Figure 1). In our studies, as the hinge binding group, we used the *N*-(pyridin-2-yl)cyclopropanecarboxamide fragment providing HBA and hydrogen bond donors (HBD) for interactions with the main chain of Val135. For a spacer group, we have selected 2,5-dihydro-1*H*-pyrrole, phenyl, pyridyl, and thiophene rings, and as HBAs, we used amide, sulphonamide, amine, nitrile, and 1,2,4-oxadiazole groups (Figure 1). The latter is used as an amide group bioisotere with increased stability^43^ and in this particular case, the replacement leads to a reduction of the number of HBD and may lead to improved pharmacokinetics. Based on the enzyme structure and molecular modeling studies, we have identified Asn186 and Asp200 as potential additional handles for interactions and we introduced additional amine groups that could satisfy them.

### Chemistry

**Scheme 1:**
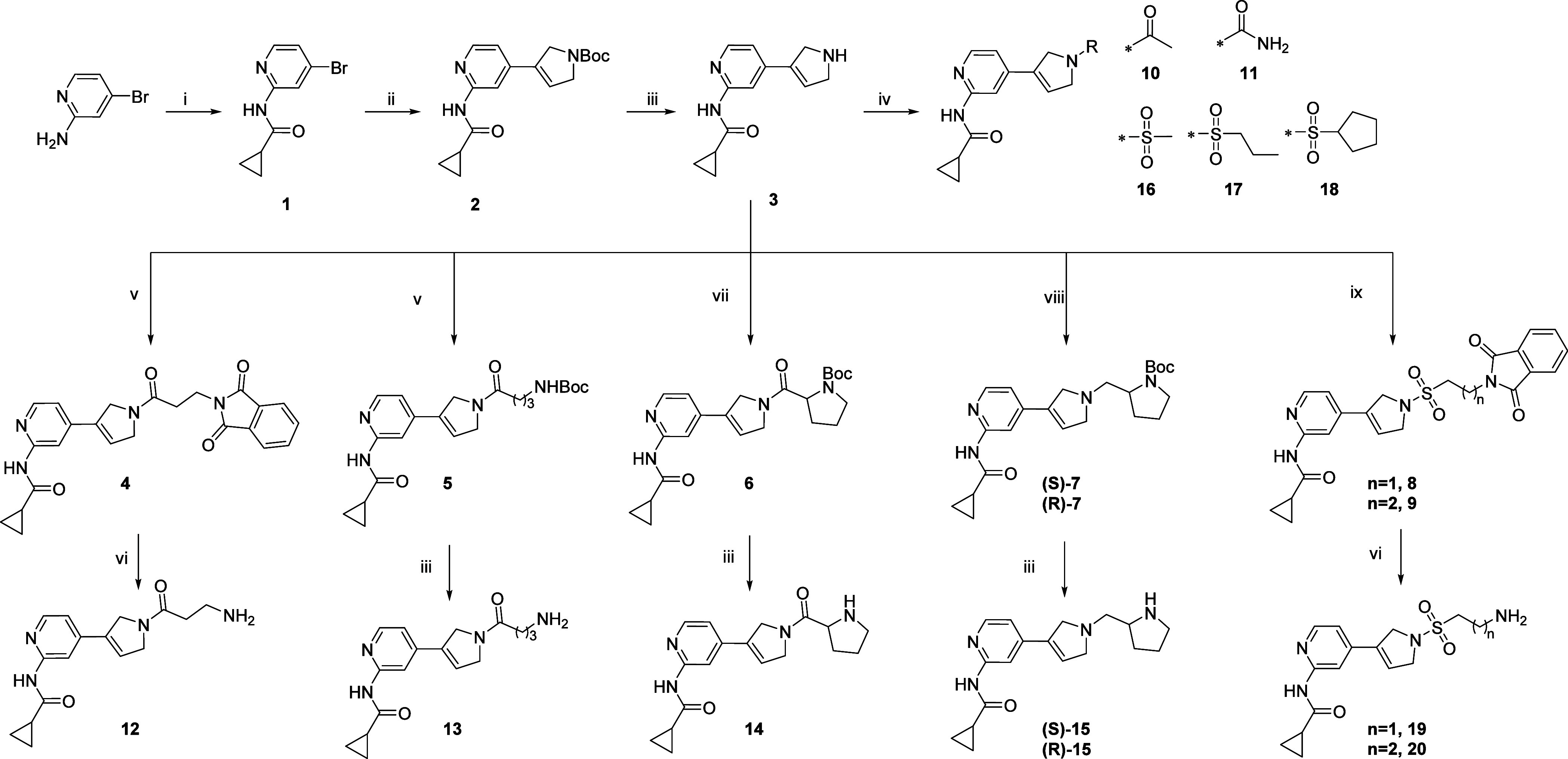
Reagents and conditions: (i) cyclopropanecarbonyl chloride, pyridine, DCM, 0 °C—rt, overnight; (ii) *tert*-butyl 3-(4,4,5,5-tetramethyl-1,3,2-dioxaborolan-2-yl)-2,5-dihydro-1*H*-pyrrole-1-carboxylate, Cs_2_CO_3_, Pd(dppf)Cl_2_, dioxane_(anh.)_, 90 °C, 3 h; (iii) 37% HCl, EtOAc or MeOH, rt or reflux, 1 h; (iv) acetyl chloride (for **10**), pyridine, DCM, 0 °C—rt, overnight/(trimethylsilyl)isocyanate (for **11**), TEA, THF_(anh.)_, rt, 8 h/appropriate sulfonyl chloride, TEA, DCM, 0 °C–rt, 1 h; (v) 3-phthalimidopropionic acid (for 4) or 4-((tert-butoxycarbonyl)amino)butanoic acid (for **5**), EDC hydrochloride, DMAP, DCM_(anh.)_, rt, overnight; (vi) NH_2_NH_2_·H_2_O, EtOH, 78 °C, 2 h; (vii) (*tert*-butoxycarbonyl)proline, EDC hydrochloride, DMAP, DIEA, DCM_(anh.)_, rt, overnight; (viii) (*S*)-*tert*-butyl 2-formylpyrrolidine-1-carboxylate (for **(*S*)-7**) or (*R*)-*tert*-butyl 2-formylpyrrolidine-1-carboxylate (for **(*R*)-7**), CH_3_COOH, NaCNBH_3_, MeOH, 0 °C—rt, overnight; (ix) 2-(1,3-dioxoisoindolin-2-yl)ethane-1-sulfonyl chloride (for **8**) or 3-(1,3-dioxoisoindolin-2-yl)propane-1-sulfonyl chloride (for **9**), DIPEA, DCM_(anh.)_, rt, overnight.

The key intermediate for the synthesis of 2,5-dihydro-1*H*-pyrrole-based compounds is compound **3** which was prepared according to Scheme 1. Commercially available 4-bromopyridin-2-amine was acylated by cyclopropanecarbonyl chloride in the presence of pyridine. Subsequently, the obtained 4-bromo derivative **1** was used in the Suzuki–Miyaura cross-coupling reaction with commercial *tert*-butyl-2,5-dihydro-1*H*-pyrrole-1-carboxylate-3-pinacol ester, in the presence of cesium carbonate and the Pd(dppf)Cl_2_ catalyst in dioxane. The next step involved Boc deprotection of **2** with HCl to give amine **3**. Then, **3** underwent various synthetic pathways leading to the final compounds with differently substituted amine moieties. Acylation with acetyl chloride allowed to obtain a short-chain amide **10**. Condensation with (trimethylsilyl)isocyanate under argon in THF afforded the urea derivative **11**. Sulfonylation with the appropriate sulfonyl chlorides in the presence of TEA or DIPEA led to the final compounds **16**–**18** and intermediates **8** and **9**, which after hydrazinolysis led to **19** and **20**. Condensation of **3** with 3-phthalimidopropionic acid, Boc-protected γ-aminobutyric acid and (*tert*-butoxycarbonyl)proline in the presence of EDC as an activating agent led to **4**, **5**, and **6**, respectively. Subsequent deprotection using hydrazine hydrate in EtOH (for **4**) and HCl in MeOH (for **5** and **6**) yielded compounds **12**, **13**, and **14**. Reductive amination with enantiomerically pure (*S*)- and (*R*)-Boc-pyrrolidine aldehydes in the presence of NaCNBH_3_ followed by Boc-deprotection with HCl in MeOH yielded final compounds **(***S***)-15** and **(***R***)-15**.

**Scheme 2:**
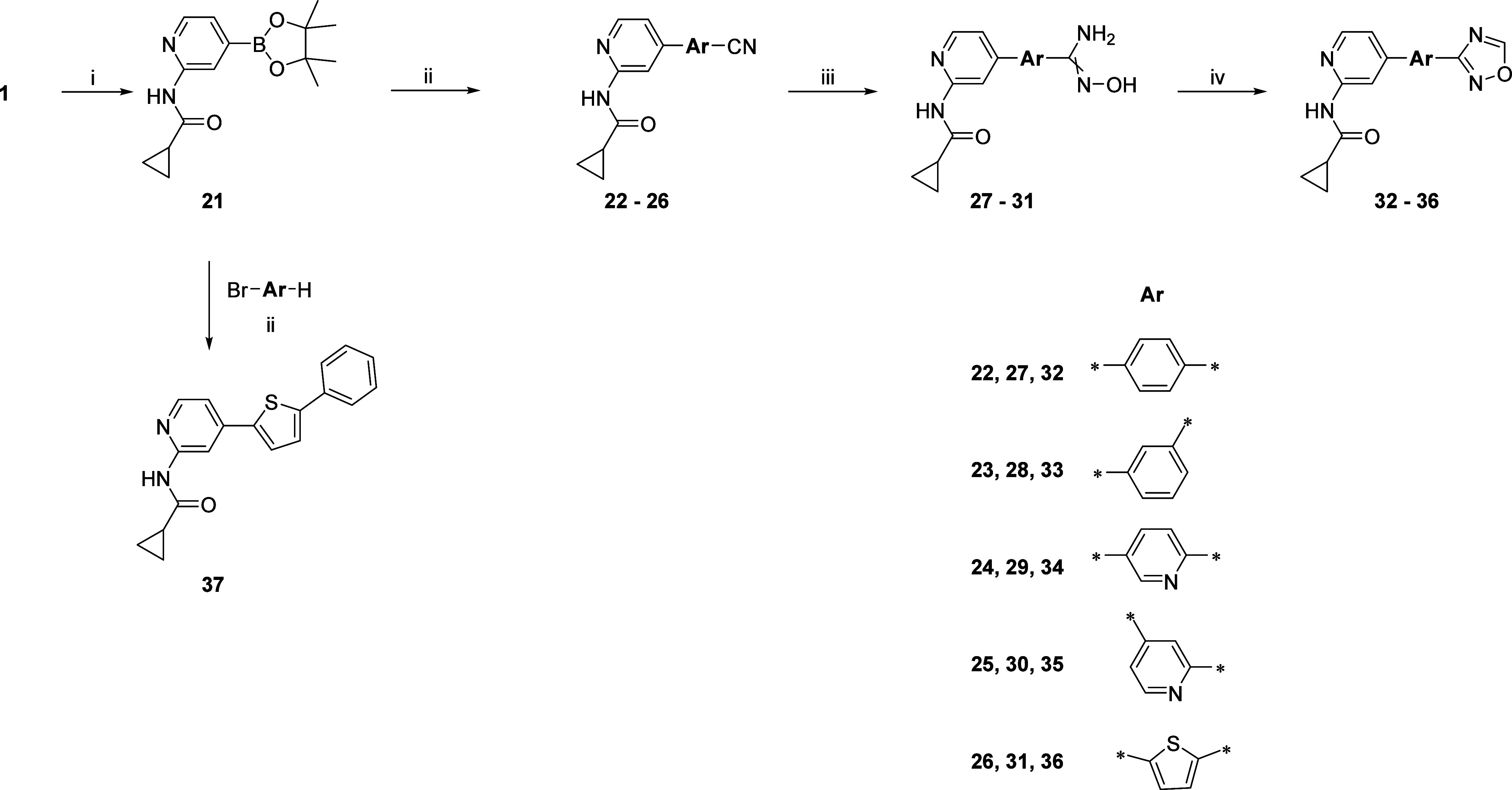
Reagents and conditions: (i) bis(pinacolato)diboron, CH_3_COOK, Pd(dppf)Cl_2_, dioxane_(anh.)_, 100 °C, overnight; (ii) appropriate aryl bromide, K_2_CO_3_, Pd(dppf)Cl_2_, DMF_(anh.)_, 80 °C, overnight; (iii) hydroxylamine hydrochloride, NaHCO_3_, EtOH, reflux, 6 h; (iv) trimethyl orthoformate, BF_3_·Et_2_O, 55 °C, 30 min.

The final compounds **32–36** were obtained in a four-step synthetic route starting with Suzuki–Miyaura cross-coupling of **1** with bis(pinacolato)diboron in the presence of Pd(dppf)Cl_2_ catalyst, yielding the pinacol ester **21** (Scheme 2). In the next step, **21** was cross-coupled with the appropriate, commercially available aryl bromides containing −CN groups, using the same catalyst. The obtained nitriles **22**–**26** were refluxed with hydroxylamine hydrochloride and NaHCO_3_ in EtOH to yield amidoximes **27**–**31**, which were then cyclized in the presence of trimethyl orthoformate and BF_3_·Et_2_O, to final oxadiazoles **32**–**36**. Cross-coupling of **21** with 2-bromo-5-phenylthiophene led to compound **37**.

### X-ray Crystallography of GSK-3β in Complex with Compound **36**

**Figure 2:**
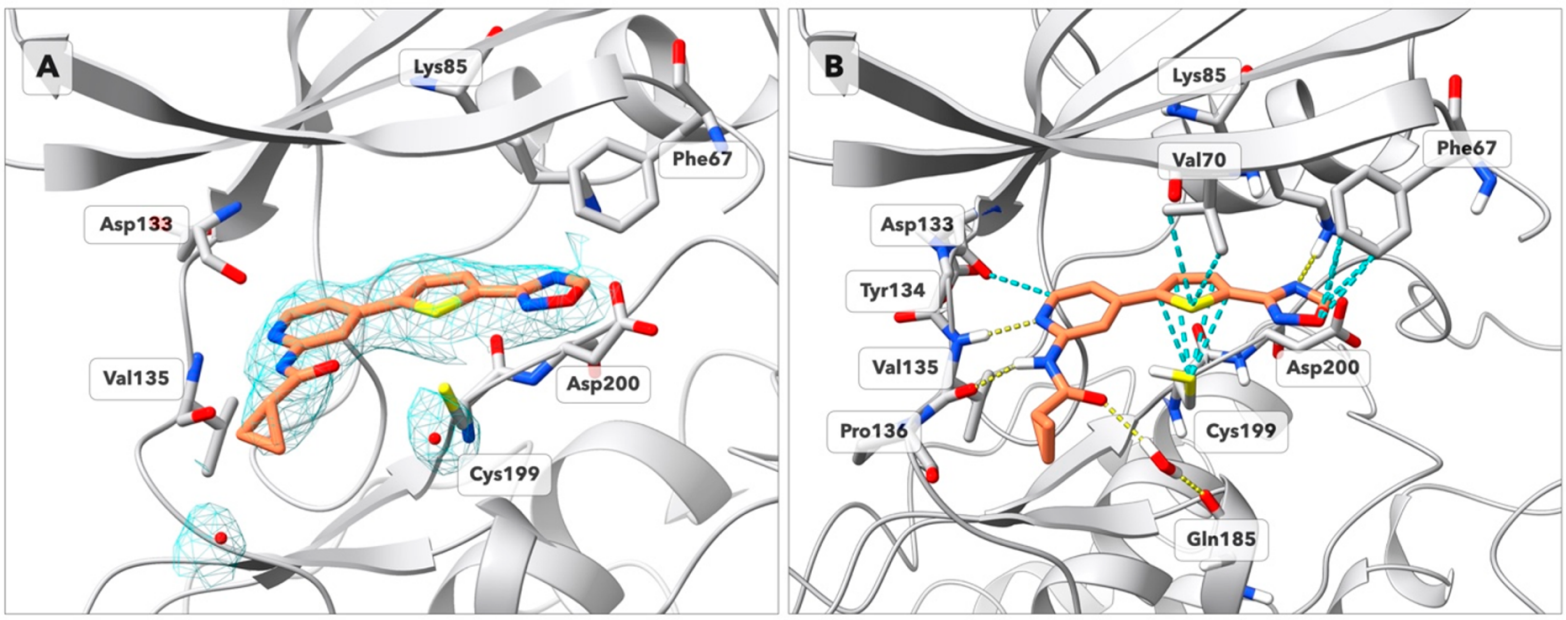
Crystal structure of GSK-3β in complex with **36** (deposited wth PDB ID: 8QJI). (A) Electron density for the ligand is shown as a mesh, 2Fo-Fc: + 1.0σ (cyan); Fo-Fc omit-map: + 3.0σ; Fo-Fc omit-map: −3.0σ (not visible at this contour level). The electronic density around the 1,2,4-oxadiazole ring does not allow for the differentiation of its orientation based solely on experimental electronic density. (B) Binding mode of **36** in the deposited structure in the ATP-binding pocket of GSK-3β after refinement with Maestro. Hydrogen bonds are shown as yellow dashed lines. Favorable contacts (van der Waals overlap >−0.3 Å) are shown as cyan-colored dashed lines. Residues 58–65 are omitted for clarity. Tyr134 and Gln185 are represented without the side chain.

The structure of compound **36** complexed with GSK-3β was solved at 3.02 Å resolution (PDB ID: 8QJI; for details, see Table S1 in the Supporting Information, SI) by molecular replacement (Figure 2). The catalytic domain of the new GSK-3β crystal structure adopts the characteristic bilobal fold. The electron density map shows the phosphorylation of Tyr216 that supports the active conformation of the A-loop, forming interactions with Arg220 and Arg223.^44,45^ The ligand’s electron density unambiguously determines the geometric orientation of compound **36** in the catalytic pocket of the enzyme. The compound occupies the ATP-binding site formed in the hinge region between the N- and C-lobes of the kinase domain. The aminopyridine fragment is oriented toward Asp133, Tyr134, and Val135 of the hinge region. At a distance of 4.00 Å from the carbonyl oxygen of **36** we identify an oxygen atom that represents a water molecule for which the electron density is observed. The thiophene and the 1,2,4-oxadiazole rings are coplanar, with the latter located in the phosphate-binding region of GSK-3β where the residues Lys85, Asp200, and Phe67 create the anchoring motif. The 1,2,4-oxadiazole ring is capturing Lys85 with an H-bond and the thiophene ring of the inhibitor forms favorable London dispersion interactions with Cys199 and Val70. An additional favorable contribution stabilizing the ligand in the hinge region is provided by the CH–O H-bond between the Asp133 main chain oxygen and the C(2) hydrogen of the pyridine ring, reflecting the tridentate harboring motif in the adenine binding region.^46^

**Figure 3:**
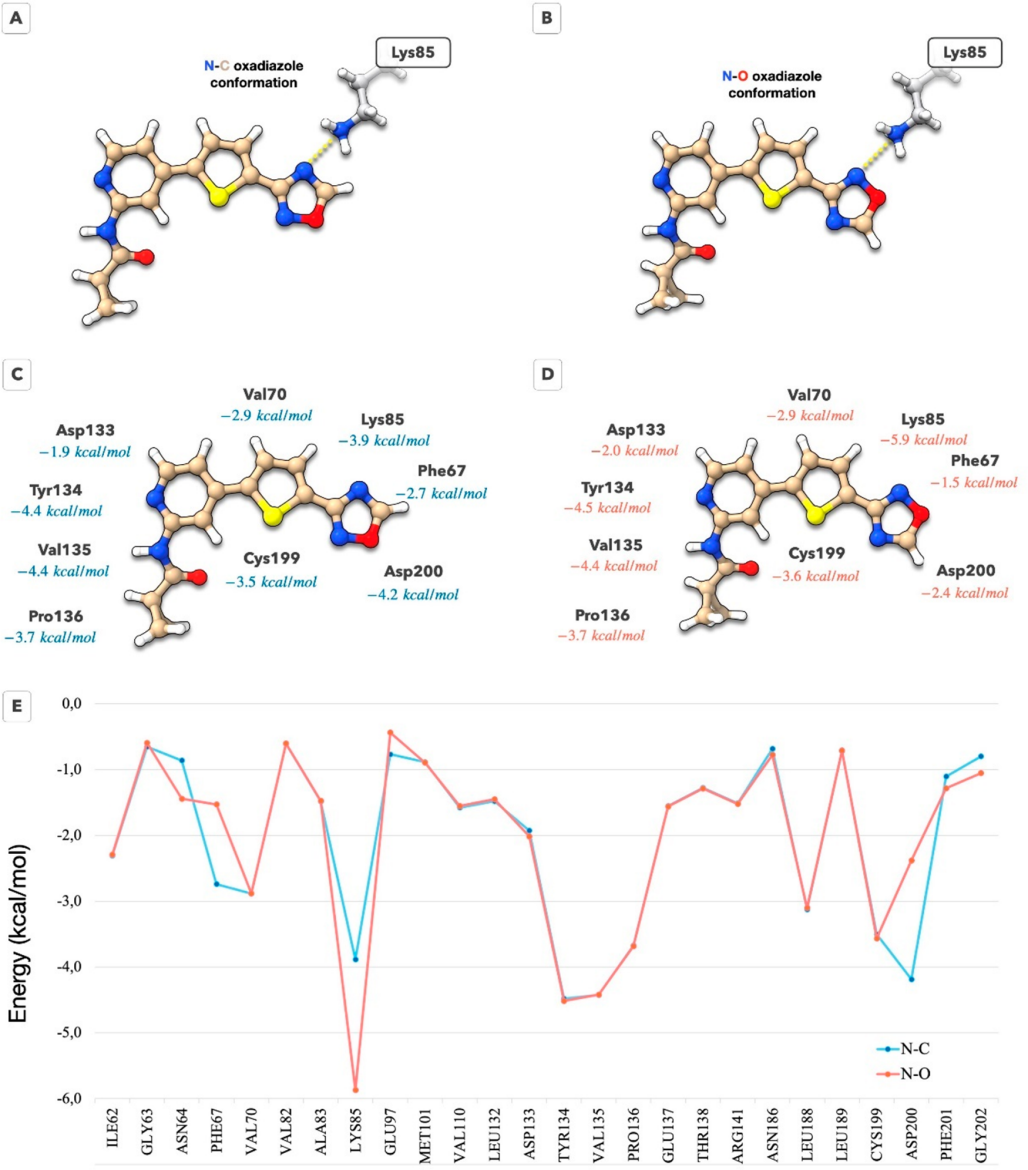
In-pocket analysis of the protein–ligand complex to determine the ligand–residue interaction patterns. (A, B) Schematic representation of the two possible binding modes reflecting orientations of the oxadiazole ring: (A) schematic representation of the N–C oxadiazole ring orientation; (B) schematic representation of the N–O oxadiazole ring orientation. (C) Ligand residue interactions for the N–C binding mode of the 1,2,4-oxadiazole. (D) Ligand residue interactions for the N–O binding mode of the 1,2,4-oxadiazole. (E) Comparison of the ligand-residue interaction energies for all residues within 5 Å from the ligand.

Although the position of the ligand in the pocket is entirely clear from the solved electronic density, the relative orientation of terminal groups cannot be unequivocally determined unless the resolution is <1.0 Å.^47^ Two orientations are distinguishable for the 1,2,4-oxadiazole: (i) the O atom in oxadiazole is exposed to the solvent, leaving the N–C bond oriented toward catalytic Lys85 (N–C orientation, Figure 3A); (ii) the O atom in oxadiazole is turned toward the catalytic Lys85 (N–O orientation, Figure 3B).^48^ To explore the potential orientations of this group, we employed our recently introduced in-pocket analysis (IPA).^49^ This method partitions the protein–ligand complex and examines the shortest ligand–residue contacts, augmented by quantum mechanical structure refinement to determine optimal proton positions. We utilized this approach to investigate the feasible orientations of the 1,2,4-oxadiazole ring, comparing N–C versus N–O configurations. Detailed results are depicted in Figure 3.

The summed interaction energies calculated by the IPA for both orientations are very close, measuring at −53.1 and −52.9 kcal/mol for the N–C and N–O binding modes, respectively. Additionally, the deformation energies are identical for both orientations, at 8.4 kcal/mol each. This suggests that compound **36** likely exhibits a dual binding mode, with oxadiazole capable of adopting either orientation. Figure 3C–E illustrates that the N–O binding mode offers advantages for interactions with Asn64 and, notably, with the catalytic Lys85. Conversely, the N–C conformation favors interactions with Phe67 and Asp200. Considering the resolution of the crystal structure and our calculations, we conclude that compound **36** likely demonstrates a dual interaction mode with the catalytic lysine.

### Biological Evaluation and SAR Analysis

We evaluated the pharmacological properties of the compounds in vitro against GSK-3β using the GSK-3β Kinase Enzyme System followed by ADP-Glo bioluminescent assay.^50^ The principle of the assay is to determine the amount of ADP formed from ATP in the kinase reaction. After the initial screening performed at 10 μM, we determined the IC_50_ values for compounds with inhibitory activity above 50%. The results are shown in Table 1. Additionally, compounds **11** and **36** in complex with GSK-3β were analyzed by the proteins’ melting temperature determination using Thermal Shift Assay (see Figure S1 in the SI). They both increased the melting point of the protein by 13 and 14 degrees respectively (in reference to DMSO). Such thermal stabilization further confirms the strong binding of the molecules to the kinase.

**Table 1:**
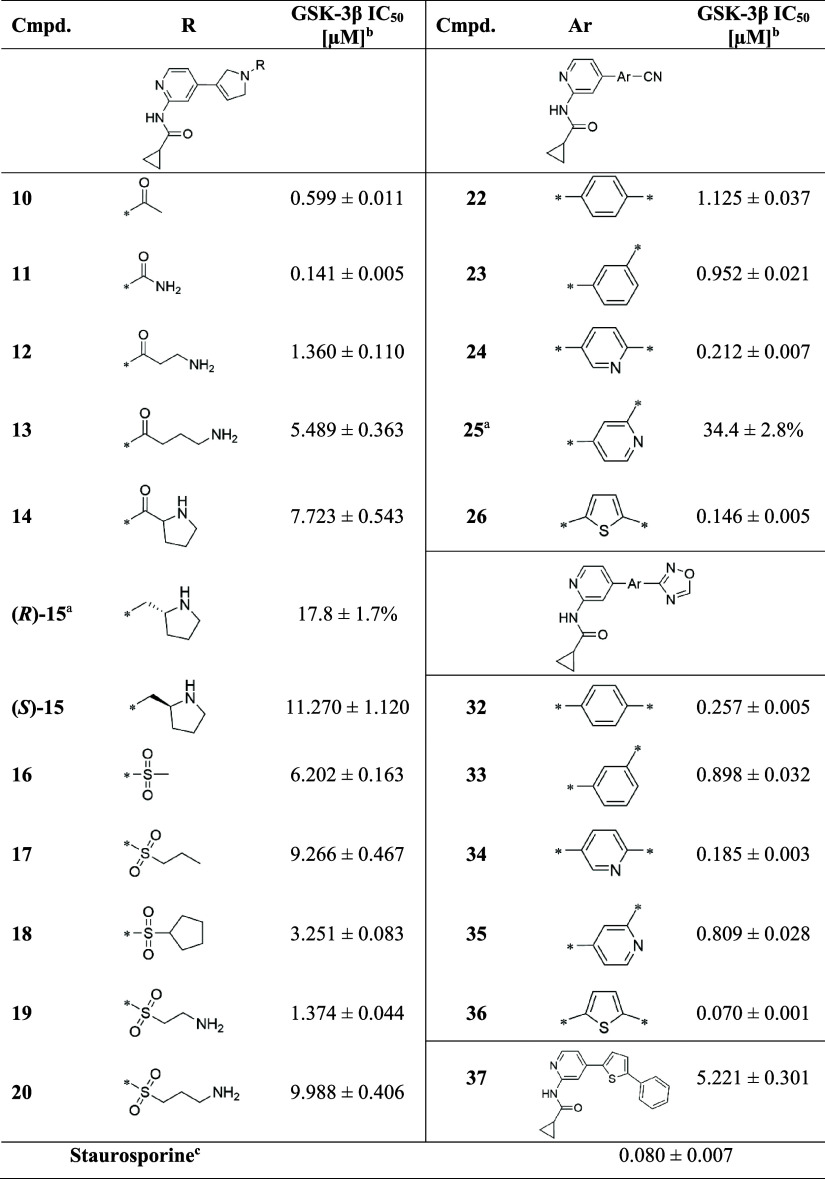
Inhibition of GSK-3β by Compounds **10**–**20**, **22**–**26**, and **32**–**37**

a Percent of enzyme inhibition at 10 μM of inhibitor concentration; mean value ± standard deviation (SD) of triplicates.

b IC_50_ inhibitory concentration of GSK-3β kinase; mean value ± standard error of the mean (SEM) of triplicates;

c Reference, Biokom, Janki, Poland.

**Figure 4:**
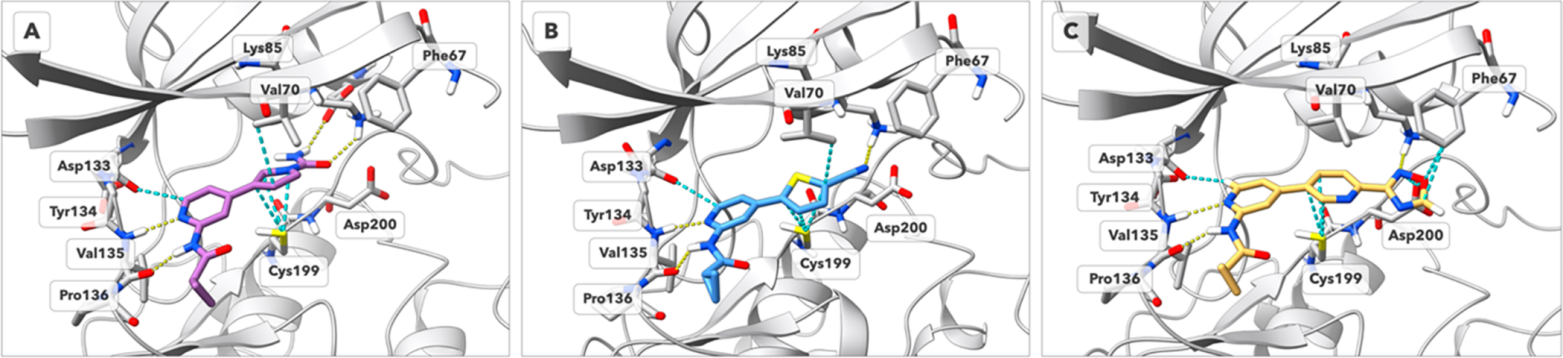
(A–C) Predicted binding modes of selected inhibitors (A) **11**, (B) **26**, and (C) **34** with the refined crystal structure of GSK-3β (PDB ID: 8QJI) generated with Glide. Hydrogen bonds are shown as yellow dashed lines. Favorable contacts (van der Waals overlap >−0.3 Å) are shown as cyan-colored dashed lines. Residues 58–65 are omitted for clarity.

The most potent compound developed in this study is **36** with an IC_50_ of 70 nM. Kinetic studies confirmed a competitive type of inhibition, allowing for the determination of a *K*_i_ value (*K*_i_ = 60.3 nM) consistent with the IC_50_ (for details including Lineweaver–Burk and Cornish–Bowden plots see Figures S26 and S27 in the SI). After refinement, the crystal structure of the complex **36**/GSK-3β (PDB ID: 8QJI) was used to analyze the interaction pattern that stands for the potency of the compound.^51^ As already discussed in the previous sections, the binding motif to Val135 is the most critical interaction between the ligands and GSK-3β, which was also previously reported in other studies.^42,52^ This part of the molecule is also stabilized by an H-bond with a solvation water molecule bridging with the main chain oxygen of Gln185. We observe that the replacement of oxadiazole with a phenyl group (compound **37**), consequently hindering the formation of hydrogen bonds with the catalytic lysine, results in a reduction in activity. H-bonds with Lys85 are preserved for analogues of **36** with the thiophene ring replaced by phenyl (**32**) and pyridyl (**34**) rings substituted at *para* positions, resulting in IC_50_ = 257 and 185 nM, respectively (Figure 4C). In *meta*-substituted derivatives, **33** and **35**, the oxadiazole is located at a distance that breaks any interaction with Lys85, and the compounds lose their activity. Similar SAR is observed for nitrile derivatives, with the most potent being compound **26** (IC_50_ = 146 nM, Figure 4B).

Within the 2,5-dihydro-1*H*-pyrrole derivatives, the most potent inhibitors are **10** (IC_50_ = 599 nM) and **11** (IC_50_ = 141 nM, Figure 4A), and a comparative SAR analysis of this pair is detailed in SC2 of the SI. The binding mode of these compounds is identical to that of **36** and its analogues, with the interactions within the hinge region preserved, the 2,5-dihydro-1*H*-pyrrole stabilized by attractive dispersion forces with the side chain of Cys199, and H-bonds formed between Lys85 and the carbonyl oxygen of the terminal *N*-acetyl- (Figure 4A) and *N*-carbamoyl-substituents. Removal of the carbonyl oxygen or its replacement with a sulfonyl moiety leads to a decrease or loss of the inhibitory activity, as observed in compounds **(*****R*****)-15**, **(*****S*****)-15**, and **16–20**. A comprehensive analysis of this modification, conducted on compounds **10** and **16**, is also detailed in the SI (SC4).

Though informative, the structural models analyzed thus far lack the quantifying power of intermolecular forces necessary to fully understand the design principles behind effective GSK-3β inhibitors. For instance, distinguishing compounds **36**, **34**, and **32** is not possible without the use of more in-depth tools. Such a comprehensive analysis is undertaken in the following section.

### Quantum Mechanical SAR: Energy Decomposition and Deconvolution Analysis

**Figure 5:**
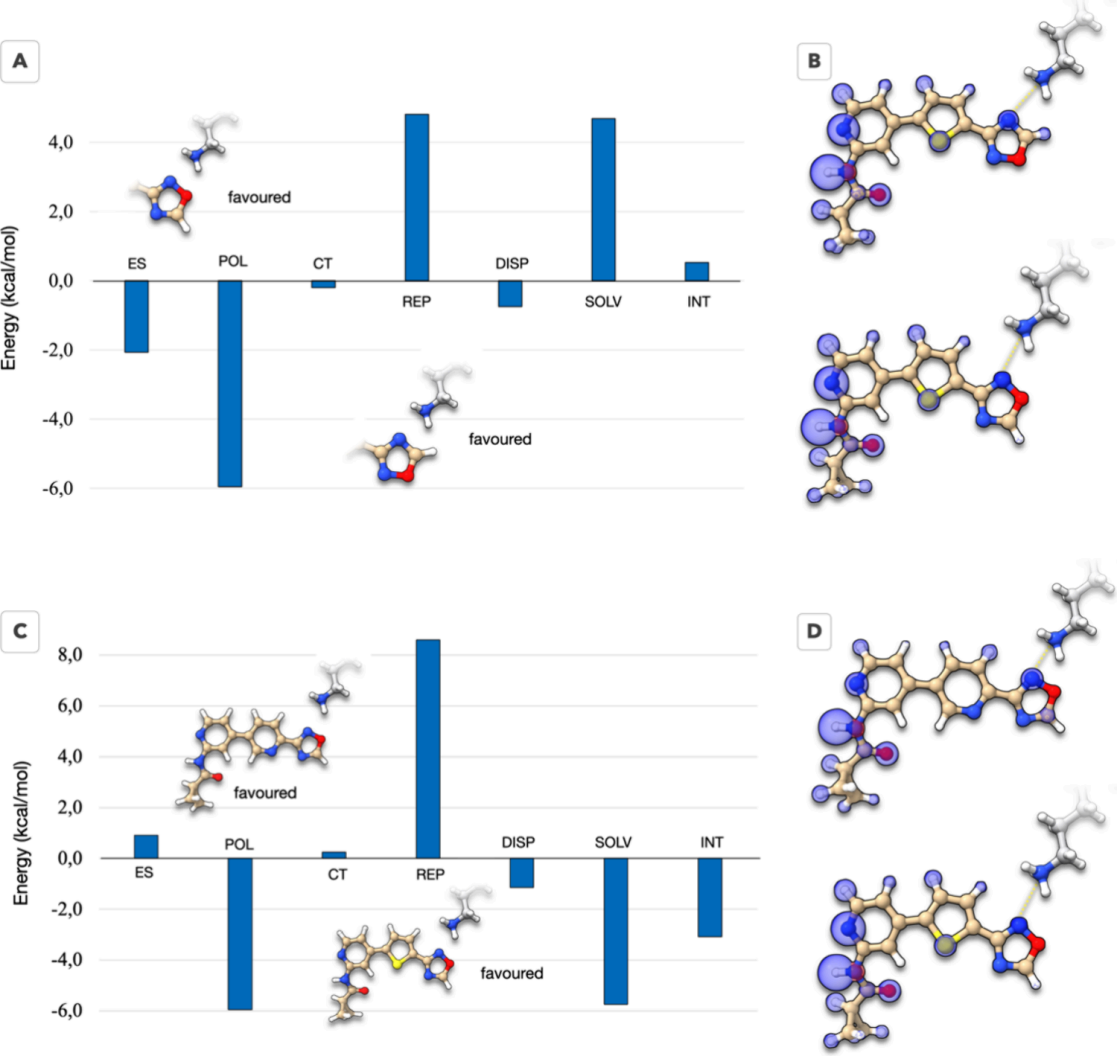
(A) Comparative EDDA for the two possible orientations of the oxadiazole ring. Though there is a minor preference for the N–O binding mode, the difference in binding energies is minimal. (B) Comparison of the total interaction energy maps, showing a minor difference in the oxadiazole ring, and a slight increase in the hinge binding pyridine group. (C) Comparative EDDA between compounds **36** and **34**. (D) Comparison of the total interaction maps for compounds **36** and **34** shows that compound **34** promotes the interaction with the catalytic lysine over the interaction with the hinge.

To further rationalize the experimental SAR data and to understand which elements of binding are key to the design of high-affinity ligands we utilized our Energy Decomposition and Deconvolution Analysis (EDDA) algorithm. EDDA is a partition scheme that effectively splits binding energies into several components, each of which is associated with a specific physical force.^45^ A brief description of the rationale behind the algorithm is available in the SI (Section SC2). An in-depth analysis of some of the SAR data from the previous sections is provided in the SI too, namely an analysis of the amide vs urea ligands (SC2 in the SI), the extension of the former to ethylaminium (SC3), and a comparison between amide and sulphonamide (SC4 in the SI). Here we focus on the key elements accounting for the activity of compound **36**. Figure 5A,B offers an alternative perspective over the two binding modes of ligand **36** discussed in the crystallography section.

The calculations show a slight preference toward the N–O binding mode of the oxadiazole in compound **36**. Comparing the respective total interaction maps reveals that when the oxadiazole binds through the N–O side, the interaction with Lys85 is weakened and balanced by a strengthening of the H-bond with Val135 of the hinge. We stress, however, that these differences are quite minimal, which is also reflected in the relative binding energy of the two binding modes. In the SI we show additional maps for other interactions (SC5), which are also barely distinguishable between the proposed binding modes. This is already indicative that the advantage brought by the oxadiazole ring is the duality in how it captures the catalytic lysin: the N–C binding mode offers a stronger hydrogen bond, as is reflected by the shorter distance obtained with Maestro (2.25 vs 2.61 Å). Overall, the calculations indicate a compensation of several driving forces, leading to equally stable binding conformations. This results in an entropic advantage for the oxadiazole group over other functionalities, e.g., an amide group. To further verify the dual binding mode of the oxadiazole in the phosphate region of the binding pocket, we run additional calculations using a minimal molecular model of this pocket conserving only essential interactions (geometries available, details in Methodology section). All calculations, DFT and ab initio, point toward the dual orientation of the oxadiazole in the binding site (see details in SC8, SI).

However, such a binding mechanism is exclusive to compound **36**. The EDDA calculations on the analogous compound **34** show a clear preference for the N–O binding mode, which offers stabilization of over 2 kcal/mol (see SC6 in the SI). It is instructive to compare the protein–ligand interactions for compounds **36** and **34** for the N–O binding mode (see Figure 5C,D). The calculations reproduce the order of experimental affinities and indicate that compound **36** offers more favorable lipophilic interactions, pays smaller desolvation penalties, and has a stronger attachment to the hinge region (see SC7 in the SI for more details). Furthermore, the calculated deformation energies show a large penalty for compound **34** to fit the pocket. An analogous analysis conducted for additional selected compounds reveals similar conclusions regarding the energetic costs of binding (see the discussions in Sections SC2–SC4 in SI). Consequently, the introduction of the thiophene spacer not only offers a better lipophilic contact with the binding pocket of GSK-3β, but it also minimizes the deformation penalty for the ligand to fit the pocket leading to an overall better protein–ligand shape complementarity.

The calculations ran indicate that the key elements for compound **36**’s affinity are (1) the preferential atomic and ring size offered by the thiophene spacer—a 5-member ring with a sulfur atom that maximizes the contacts to the pocket, simultaneously optimizing the protein–ligand shape complementarity; (2) the dual binding mode of the oxadiazole ring, making it in this specific case an improved bioisostere of the amide group by favoring entropic contributions to the binding.

### In Cellulo Studies

#### Cytotoxicity in HT-22 and BV-2 Cells

The cytotoxic effect of the most potent GSK-3β inhibitors **11**, **34**, **36** was measured in two cell lines, the mouse hippocampal neuronal cells HT-22 and the mouse microglial cells BV-2 using PrestoBlue cell viability reagent. The compounds were tested at 5 concentrations (0.1, 1, 10, 50, and 100 μM). No significant decrease in cell viability was observed in the whole range of the concentrations for compounds **34** and **36** in HT-22 cells (Table 2). Compound **11** displayed an IC_50_ of 45.8 μM, which is over 300 times higher than its effective activity against GSK-3β. A more pronounced effect of the compounds was observed on the BV-2 cell, although the compounds did not affect cell viability up to 10 μM, and their IC_50_s were at least 160 times higher than the effective GSK-3β inhibitory concentration.

**Table 2:** Cytotoxicity of **11**, **34**, and **36** in HT-22 and BV-2 Cells^a^

**compound**	**11**	**34**	**36**
cytotoxicity in HT-22 cells			
IC_50_ [μM] *x̅* ± SEM	45.8 ± 9.7	>100	>100
cytotoxicity in BV-2 cells			
IC_50_ [μM] *x̅* ± SEM	23.4 ± 1.7	39.2 ± 5.2	89.3 ± 4.9

a Data expressed as the means ± SEM; *N* ≥ 6.

#### Evaluation of Inhibitory Activity toward Okadaic Acid-Induced Hyperphosphorylation

**Figure 6:**
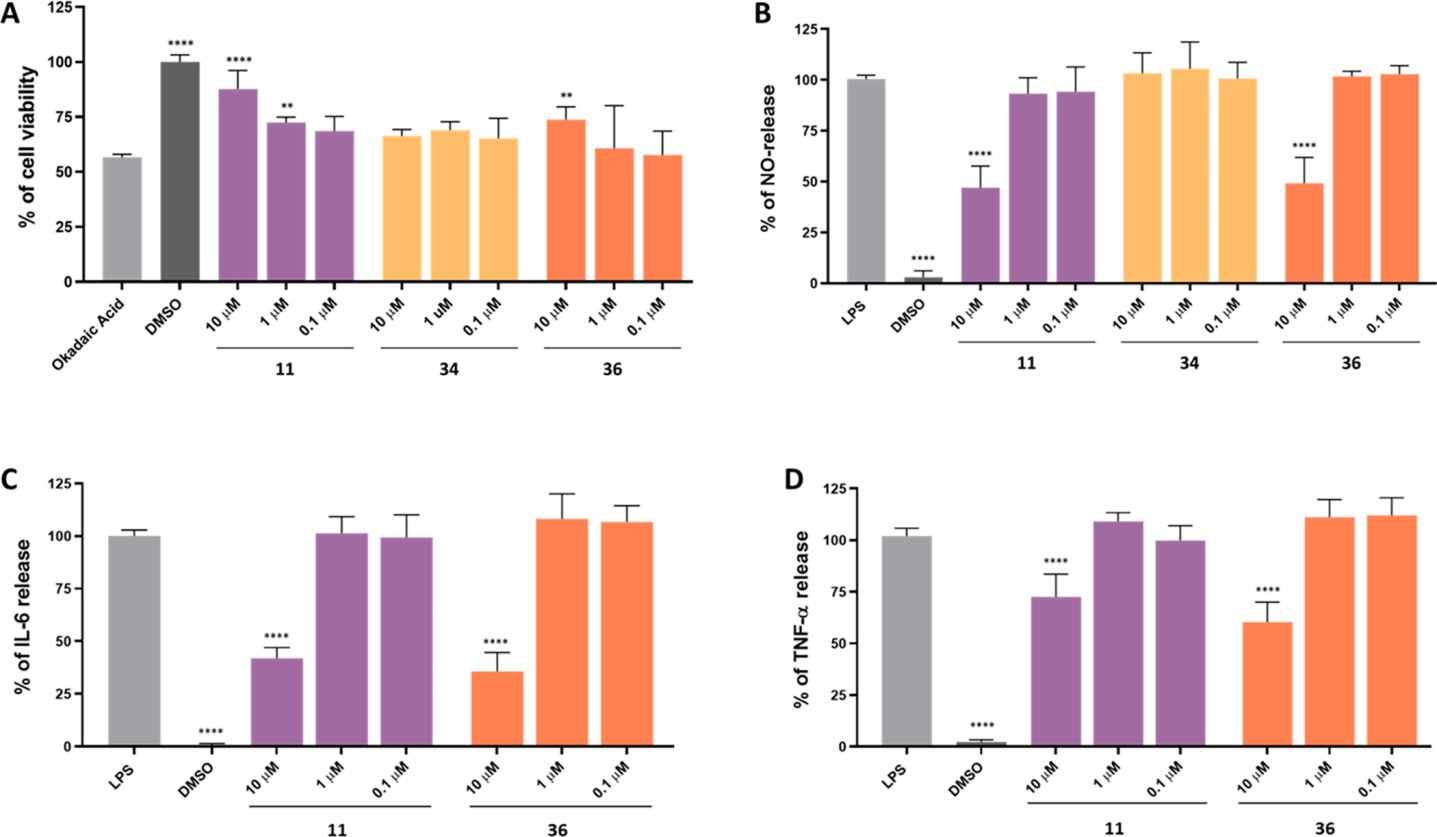
(A) Effect of compounds **11**, **34**, **36** (0.1, 1, 10 μM) on okadaic acid-induced hyperphosphorylation. Before the addition of compounds, HT-22 cells were treated for 3 h with 400 nM of okadaic acid. Cell viability was determined by Presto Blue assay after 24 h. (B) Effect of compounds **11**, **34**, and **36** (0.1, 1, 10 μM) on NO-release (%) in LPS-treated (100 ng/mL) BV-2 cell line. NO-release was measured using a fluorometric assay with 2,3-diaminonaphthalene (DAN). (C, D) Effects of compounds **11** and **36** (0.1, 1, 10 μM) on IL-6 and TNF-α release in LPS-treated (100 ng/mL) BV-2 cell line. The IL-6 and TNF-α levels were measured using LANCE Ultra TR-FRET Detection Kit (PerkinElmer). Statistical analysis was performed using GraphPad Prism 9.0.0. All values are expressed as mean with SD. Differences among groups were evaluated by one-way ANOVA followed by posthoc analysis (Dunnett’s multiple comparison tests) vs. control group (LPS on BV-2 cells and okadaic acid on HT-22 cells) and were considered statistically significant if *p* < 0.05 (*p < 0.05, **p < 0.01, ***p < 0.001, and ****p < 0.0001).

Okadaic acid is a phosphatase inhibitor that leads to hyperphosphorylation and accumulation of neurofilaments similar to those observed in AD brain. Thus, it is used in a cell model of hyperphosphorylated tau-induced neurodegeneration.^53,54^ We used this model to confirm the ability of compounds **11**, **34**, and **36** to reverse the effect of okadaic acid in cellulo (Figure 6A). The test was performed on the HT-22 cell line treated with okadaic acid at the concentration of 400 nM and the compounds at the concentration of 0.1, 1, and 10 μM. Cell viability was measured by the PrestoBlue cell viability reagent. A statistically significant effect in terms of an increase in cells’ viability was observed for compound **11** at the concentration of 10 and 1 μM and for compound **36** at 10 μM.

#### Evaluation of Anti-Inflammatory Activity in BV-2 Microglial Cells

An important role in the pathogenesis of AD has been attributed to neuroinflammation resulting from the activation of astrocytes and microglial cells.^55^ These processes lead to increased production of proinflammatory cytokines such as TNF-α or IL-6 that activate processes, e.g., tau hyperphosphorylation, causing injury and cell death.^56^ A standard model of neuroinflammation is based on lipopolysaccharide-stimulated BV-2 microglial cells. In this study, we used this model to evaluate the anti-inflammatory properties of compounds **11**, **34**, and **36**.^57^ Our evaluation focused on monitoring the levels of key inflammatory markers, including nitric oxide (NO, Figure 6B) and cytokines, TNF-α and IL-6 (Figure 6C,D). Notably, compounds **11** and **36** exhibited the most pronounced anti-inflammatory effects, significantly reducing the release of NO, TNF-α, and IL-6 at a concentration of 10 μM, as illustrated in Figure 6**B–D**. Compound **34** did not show any impact on NO release and was therefore not subjected to further testing.

### Preliminary In Vitro ADMET Profiling

For selected compounds, **11** and **36**, we performed in vitro ADMET profiling studies including permeability, metabolic stability and influence on CYP activity (Table 3).

**Table 3:** Results of In Vitro ADMET Profiling for **11** and **36** (PAMPA, Metabolic Stability)

compound	**11**	**36**
PAMPA^a^		
*Pe*^b^ (10^–6^ cm/s) ± SD	0.53 ± 0.82	9.40 ± 0.89
CNS (±)	–	+
metabolic stability in human microsomes		
% of the compound remaining after 2 h of incubation^c^	100	88.6

a PAMPA assay (precoated PAMPA Plate System Gentest, Corning, Tewksbury, MA, USA).

b The permeability coefficient (*Pe*) values determined for compounds **11** and **36**. Caffeine was used as the well-permeable reference compound (*Pe* = (10.44 ± 1.88) × 10^–6^ cm/s). Data is expressed as a mean of three replicates (*n* = 3) ± SD (10^–6^ cm/s).

c Reference compound: verapamil (23.9%).^58^

#### Permeability

We assessed the permeability of selected compounds in the Parallel Artificial Membrane Permeability Assay (PAMPA) described by Chen et al. using caffeine as a well-permeable reference (*Pe* = (10.44 ± 1.88) × 10^–6^ cm/s). Based on the obtained permeability coefficients (*Pe*), we classified compound **36** as well permeable, with *Pe* value similar to that of caffeine (Table 3). According to the results, compound **11** might not penetrate through the biological membranes. It might result from a low lipophilicity coupled with a relatively high total polar surface area (c log *P* = 0.35, TPSA = 88.32, calculated with Marvin 17.21.0, Chemaxon; https://www.chemaxon.com) which encourages a tendency to remain in the aqueous solution.

#### Metabolic Stability

The primary site of drug metabolism in humans is the liver. Therefore, we used human liver microsomes (HLM) to determine the metabolic stability of selected compounds (Table 3). The compounds were incubated with HLMs for 2 h, and the resulting mixtures were analyzed with UPLC-MS (for details including the UPLC-MS spectra, see Table S10 and Figures S17–S22 in the SI). Interestingly, compound **11** did not undergo any metabolic transformation after the incubation time. Compound **36** was metabolized in only 11%. The tested compounds proved to be stable when compared to the marketed drug verapamil (76% of the compound was metabolized).

#### Influence on CYP3A4, CYP2D6, and CYP2C9 Activity

Interactions with cytochrome enzymes are significant contributors to drug–drug interactions (DDIs), a crucial concern for patients who are on multiple medications. We determined the compounds’ influence on the most important CYP isoforms 3A4, 2D6, and 2C9, using the CYP450 inhibition luminescence assay from Promega (for details see Figures S23–S25 and Table S11 in the SI). The inhibitory effect of compounds **11** and **36** was observed only at the highest tested concentration (25 μM) on CYP2C9 and CYP3A4 (for **36** also at 10 μM), while no effect on CYP2D6 was detected at any concentration.

### Kinase Selectivity Evaluation

**Figure 7:**
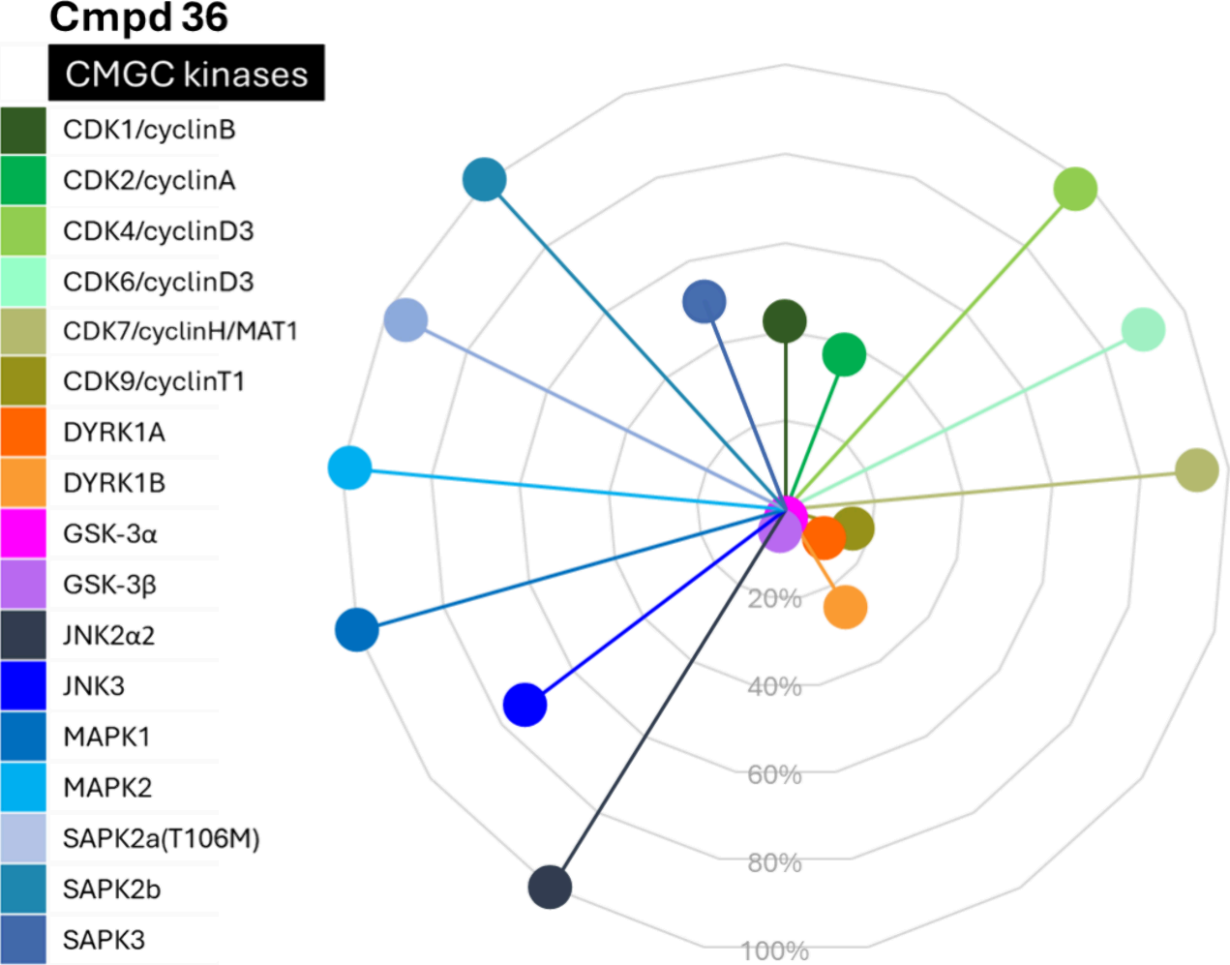
Kinase selectivity for compound **36**. Data are presented as percent of kinases’ activity in the presence of 1 μM of compound **36**. Kinases’ panel (CMGC group, human enzymes): CDK1/cyclinB, CDK2/cyclinA, CDK4/cyclinD3, CDK6/cyclinD3, CDK7/cyclinH/MAT1, CDK9/cyclinT1, DYRK1A, DYRK1B, GSK-3α, GSK-3β, JNK2α2, JNK3, MAPK1, MAPK2, SAPK2a(T106M), SAPK2b, SAPK3.

Based on the above-described in vitro and in cellulo studies, compound **36** was selected for evaluation of selectivity against a panel of the related kinases from the CMGC group. We selected those kinases with the greatest potential for interaction based on structural homology, as indicated by the ChemPartner panel. The screening was performed at a concentration of 1 μM, at which the compound displays 92% inhibition of GSK-3β (Figure 7 and Table S12 in the SI). This allowed us to identify other kinases inhibited similarly to GSK-3β. The studies revealed the selectivity of compound **36** against most of the tested kinases (less than 50% of inhibition) including CDK4, CDK6, CDK7, JNK2α2, JNK3, MAPK1, MAPK2, SAPK2a, SAPK2b and SAPK3. At the same time, it confirmed the high inhibitory potency against both GSK-3α and GSK-3β kinases, which is not surprising given their 98% homology within their respective catalytic domains. Current research suggests that they share very similar, if not entirely redundant, functions in numerous cellular processes, making the inhibition of both justified.^59,60^ Similarly, due to the role of DYRK in Aβ and tau formation,^61,62^ the inhibition of DYRK kinases might be of additional value in Alzheimer’s disease studies. Inhibition of CDK1, 2, and 9 kinases (58, 63, and 84% respectively) needs attention and optimization in further studies.

## Conclusions

In the course of our research, aiming at identifying compounds with the potential to effectively treat Alzheimer’s disease, we uncovered a noteworthy compound **36**. As a GSK-3β inhibitor (IC_50_ = 70 nM) it has the potential to interfere with processes directly implicated in the onset and progression of the disease, including the aggregation of amyloid-β and tau proteins, as well as neuroinflammatory processes. Compound **36** proved to be effective in a cell model of hyperphosphorylated tau-induced neurodegeneration where it restored cell viability after okadaic acid treatment. Further evaluation revealed its anti-inflammatory activity in the cell-based LPS model, significantly reducing NO, IL-6, and TNF-α release at 10 μM. The compound displayed beneficial ADME properties determined in vitro, including high permeability in PAMPA-BBB (*Pe* equals 9.4) and metabolic stability on HLMs (88.6% remained unchanged after 2 h of incubation). In terms of safety, **36** lacked significant interactions with CYP enzyme isoforms 3A4 (up to 10 μM), 2D6 (in none of the tested concentrations), and 2C9 (up to 25 μM), and displayed cytotoxicity with IC_50_ > 100 μM on HT-22 cells and 89.3 μM on BV-2 cells.

The crystal structure of compound **36** complexed with GSK-3β was solved by X-ray crystallography. Our computational analysis with quantum mechanical-based models allowed us to determine the molecular mechanism behind GSK-3β inhibition with this inhibitor, as well as the SAR of other compounds from the series described. Introducing an oxadiazole ring as an amide bioisoster brings advantages in terms of protein–ligand shape complementarity, allowing the simultaneous capture of the hinge region and the catalytic Lys85. The suggested dual binding mode for compound **36**, supported by in-depth quantum mechanical analysis, efficiently exploits the interaction space of the phosphate region of the binding pocket.

The study not only identifies a compelling candidate with potential for further development in Alzheimer’s disease treatment but also underscores the significance of the energy decomposition and deconvolution analysis (EDDA) algorithm. This tool proves useful for providing a rational explanation of structure–activity relationships, facilitating a more efficient design of new ligands.

## Methods

### General Chemistry Information

All reagents were purchased from commercial suppliers and were used without further purification unless stated otherwise. Tetrahydrofuran (THF) and dichloromethane (DCM) were distilled under argon immediately before use. The drying agent used for THF was sodium/benzophenone ketyl, and for DCM, calcium hydride. Reactions were monitored by thin-layer chromatography carried out on aluminum sheets precoated with silica gel 60 F254 (Merck). Compounds were visualized with UV light and by suitable visualization reagents (solution of ninhydrin). Compounds were purified with flash chromatography on Isolera Spectra (Biotage) with silica gel 60 (63–200 μm; Merck) as a stationary phase or using reverse-phase HPLC performed on LC-4000 Jasco with a Phenomenex Luna C8 (5 μm, 15 × 21.2 mm) column and water/acetonitrile gradient with 0.1% solution of formic acid (v/v) as a mobile phase. The UPLC-MS analyses were done on UPLC-MS/MS system comprising Waters ACQUITY UPLC (Waters Corporation, Milford, MA, USA) coupled with Waters TQD mass spectrometer (electrospray ionization mode ESI with tandem quadrupole). Chromatographic separations were carried out using the ACQUITY UPLC BEH (bridged ethyl hybrid) C18 column: 2.1 × 100 mm and 1.7 μm particle size. The column was maintained at 40 °C and eluted under gradient conditions using 95–0% of eluent A over 10 min, at a flow rate of 0.3 mL/min. Eluent A: 0.1% solution of formic acid in water (v/v); eluent B: 0.1% solution of formic acid in acetonitrile (v/v). A total of 10 μL of each sample was injected and chromatograms were recorded using Waters eλ PDA detector. The spectra were analyzed in the range of 200–700 nm with 1.2 nm resolution and at a sampling rate of 20 points/s. The UPLC/MS purity of all the test compounds was determined to be ≥95% and is given for each compound in the following description. ^1^H NMR and ^13^C NMR spectra were recorded on Varian Mercury 300 MHz (Varian, Inc., Palo Alto, CA) or Jeol 500 MHz (Jeol Inc., Peabody, MA). The chemical shifts are reported in ppm and were referenced to the residual solvent signals (CDCl_3_^1^H: 7.26 ppm, ^13^C: 77.16 ppm; CD_3_OD ^1^H: 3.31 ppm, ^13^C: 49.00 ppm; D_2_O ^1^H: 4.79 ppm; DMSO-*d*_6_^1^H: 2.50 ppm, ^13^C: 39.52 ppm), coupling constants are reported in hertz (Hz). HRMS analyses were performed on MALDI-TOF/TOF mass spectrometer UltrafleXtreme from Bruker Daltonics (Bremen, Germany) with α-cyano-4-hydroxycinnamic acid (CHCA) MALDI matrix after standard dried droplet preparation on ground steel target plate.

### Previously Reported Compounds

*N*-(4-Bromopyridin-2-yl)cyclopropanecarboxamide (**1**),^63^*N*-(4-(4,4,5,5-tetramethyl-1,3,2-dioxaborolan-2-yl)pyridin-2-yl)cyclopropanecarboxamide (**21**)^63^

### Chemical Synthesis

#### *N*-(4-Bromopyridin-2-yl)cyclopropanecarboxamide (**1**)

2-Amino-4-bromopyridine (2.00 g, 11.56 mmol, 1 equiv) was dissolved in 40 mL DCM, solution was cooled to 0 °C on an ice bath, then pyridine (1.87 mL, 23.12 mmol, 2 equiv) and cyclopropanecarbonyl chloride (1.21 mL, 13.29 mmol, 1.15 equiv) were added dropwise. The reaction mixture was then warmed up to rt and stirred overnight. After that time, the mixture was extracted with DCM, combined organic layer was dried over anhydrous Na_2_SO_4_, filtered and concentrated under vacuum. The product did not require further purification. Yield: 2.54 g (91%). ^1^H NMR (500 MHz, chloroform-*d*) δ ppm 0.91–0.96 (m, 2H), 1.08–1.14 (m, 2H), 1.63–1.70 (m, 1H), 7.23 (dd, *J* = 5.7, 1.7 Hz, 1H), 8.02 (d, *J* = 5.7 Hz, 1H), 8.58 (d, *J* = 1.7 Hz, 1H), 9.61 (br s, 1H). Formula: C_9_H_9_BrN_2_O. MW: 241.09.

#### *Tert*-Butyl 3-(2-(Cyclopropanecarboxamido)pyridin-4-yl)-2,5-dihydro-1*H*-pyrrole-1-carboxylate (**2**)

*Tert*-Butyl 3-(4,4,5,5-tetramethyl-1,3,2-dioxaborolan-2-yl)-2,5-dihydro-1*H*-pyrrole-1-carboxylate (1.02 g, 3,45 mmol, 1 equiv) and **1** (1.0 g, 3.47 mmol, 1 equiv) were dissolved in 20 mL anhydrous dioxane. Then Cs_2_CO_3_ (2.26 g, 6.94 mmol, 2 equiv) was added, and under Ar Pd(dppf)Cl_2_ (505 mg, 0.69 mmol, 0.2 equiv). The mixture was stirred at 90 °C for 4 h. After that time, reaction mixture was diluted with DCM, filter through Celite and evaporated under reduced pressure. Purification: flash chromatography (DCM/PE/EtOAc 5:2:3). Yield: 965 mg (71%). ^1^H NMR (500 MHz, CDCl_3_ δ ppm 0.99–1.04 (m, 2H), 1.14 (br dd, *J* = 7.3, 3.6 Hz, 2H), 1.23 (s, 9H), 1.89 (br s, 1H), 4.33–4.42 (m, 2H), 4.50 (dt, *J* = 18.7, 3.8 Hz, 2H), 6.67 (br s, 1H), 8.10 (d, *J* = 6.0 Hz, 1H), 8.43 (s, 1H), 8.57 (s, 1H), 11.27 (br s, 1H). Formula: C_18_H_23_N_3_O_3_. MW: 329.40

#### *N*-(4-(2,5-Dihydro-1*H*-pyrrol-3-yl)pyridin-2-yl)cyclopropanecarboxamide (**3**)

To a solution of **2** (425 mg, 1.29 mmol, 1 equiv) in 9 mL EtOAc 37% HCl (530 μL, 6.45 mmol, 5 equiv) was added and the mixture was stirred at rt for 1 h. When all of starting material was consumed up, the pH was adjusted to 8 by addition of saturated aqueous K_2_CO_3_ solution and the solvents were concentrated. The residue was purified by flash chromatography (DCM/MeOH/NH_3(aq)_ 95:5:0.5 then 92:8:0.8). Yield: 210 mg (71%). ^1^H NMR (500 MHz, DMSO-*d*_6_) δ ppm 0.71–0.82 (m, 4H), 1.92–2.01 (m, 1H), 3.13 (s, 1H), 3.72–3.80 (m, 2H), 3.88 (td, *J* = 4.4, 1.9 Hz, 2H), 6.58 (t, *J* = 2.0 Hz, 1H), 7.12 (dd, *J* = 5.3, 1.6 Hz, 1H), 8.00 (s, 1H), 8.21 (dd, *J* = 5.2, 0.6 Hz, 1H), 10.74 (s, 1H). Formula: C_13_H_15_N_3_O. MW: 229.28

#### *N*-(4-(1-Acetyl-2,5-dihydro-1*H*-pyrrol-3-yl)pyridin-2-yl)cyclopropanecarboxamide (**10**)

**3** (60 mg, 0.26 mmol, 1 equiv) was dissolved in 1.5 mL DCM, solution was cooled to 0 °C on an ice bath, then pyridine (42 μL, 0.52 mmol, 2 equiv) and acetyl chloride (21 μL, 0.3 mmol, 1.15 equiv) were added dropwise. The reaction mixture was then warmed up to rt and stirred overnight. After that time, the mixture was extracted with DCM, combined organic layer was dried over anhydrous Na_2_SO_4_, filtered and concentrated under vacuum. Purification: flash chromatography (DCM/EtOAc/MeOH 96:2:2 then 92:4:4), then preparative HPLC (5–50% MeCN gradient). Yield: 9 mg (13%), yellow oil. ^1^H NMR (500 MHz, CHLOROFORM-*d*) δ ppm 0.91–1.00 (m, 2H), 1.09–1.17 (m, 2H), 1.73 (td, *J* = 7.8, 3.9 Hz, 1H), 2.14 (d, *J* = 19.2 Hz, 3H), 4.43–4.52 (m, 2H), 4.57–4.67 (m, 2H), 6.56 (dt, *J* = 25.5, 2.0 Hz, 1H), 7.06 (ddd, *J* = 62.4, 5.4, 1.4 Hz, 1H), 8.20 (dd, *J* = 5.6, 2.1 Hz, 1H), 8.34 (d, *J* = 84.8 Hz, 1H), 9.73 (br s, 1H). Rotamer 1: ^13^C NMR (126 MHz, CHLOROFORM-*d*) δ ppm 9.34 (2C), 16.14, 21.92, 52.66, 53.96, 110.72, 115.72, 126.35, 127.90, 135.08, 144.52, 151.31, 169.25, 173.63. Rotamer 2: ^13^C NMR (126 MHz, CHLOROFORM-*d*) δ ppm 9.34 (2C), 16.17, 22.27, 53.67, 55.12, 110.93, 115.98, 126.35, 127.90, 136.12, 144.76, 151.67, 169.30, 173.77. Formula: C_15_H_17_N_3_O_2_. MW: 271.32. LC-MS: *m*/*z* 272 [M + H]^+^.

#### 3-(2-(Cyclopropanecarboxamido)pyridin-4-yl)-2,5-dihydro-1*H*-pyrrole-1-carboxamide (**11**)

To a stirred solution of **3** (60 mg, 0.26 mmol, 1 equiv) in 2 mL anhydrous THF TEA (360 μL, 2.6 mmol, 10 equiv) and (trimethylsilyl)isocyanate (352 μL, 2.6 mmol, 10 equiv) were added dropwise. The reaction mixture was stirred at rt for 8 h. After that time, solvent was evaporated under reduced pressure. Purification: flash chromatography (DCM/EtOAc/MeOH 95:2.5:2.5 then 9:5:5). Yield: 10 mg (14%), white solid. ^1^H NMR (500 MHz, DMSO-*d*_6_) δ ppm 0.75–0.88 (m, 4H), 1.96–2.06 (m, 1H), 4.15–4.26 (m, 2H), 4.31–4.39 (m, 2H), 5.93 (s, 2H), 6.64 (s, 1H), 7.22 (d, *J* = 4.3 Hz, 1H), 8.07 (s, 1H), 8.29 (d, *J* = 5.2 Hz, 1H), 10.84 (s, 1H). ^13^C NMR (126 MHz, DMSO-*d*_6_) δ ppm 7.69 (2C), 14.18, 52.53, 53.80, 109.43, 115.52, 135.55, 141.93, 148.16, 149.96, 152.88, 157.00, 172.76. Formula: C_14_H_16_N_4_O_2_. MW: 272.31. LC-MS: *m*/*z* 273 [M + H]^+^.

### General Procedure for the Synthesis of Compounds **16**, **17**, **18** (**GP1**)

To *N*-(4-(2,5-dihydro-1*H*-pyrrol-3-yl)pyridin-2-yl)cyclopropanecarboxamide (**3**) (1 equiv) dissolved in anhydrous DCM TEA (3 equiv) was added. Then solution was cooled to 0 °C on an ice bath and appropriate sulfonyl chloride (1–3 equiv) was added dropwise. The reaction mixture was warmed up to rt and stirred 1 h. After that time, solvent was evaporated and the crude product was purified by different methods described below.

#### *N*-(4-(1-(Methylsulfonyl)-2,5-dihydro-1*H*-pyrrol-3-yl)pyridin-2-yl)cyclopropanecarboxamide (**16**)

Following **GP1**, compound **16** was prepared using **3** (51 mg, 0.22 mmol), mesyl chloride (17 μL, 0.22 mmol), TEA (92 μL, 0.66 mmol) in 3 mL DCM. Purification: flash chromatography (DCM/MeOH 92:8), then the solid residue was washed with MeCN. Yield: 38 mg (56%), white solid. ^1^H NMR (500 MHz, DMSO-*d*_6_) δ ppm 0.78–0.85 (m, 4H), 1.96–2.05 (m, 1H), 2.99 (s, 3H), 4.27–4.32 (m, 2H), 4.43–4.47 (m, 2H), 6.60–6.64 (m, 1H), 7.25 (dd, *J* = 5.2, 1.7 Hz, 1H), 8.06 (s, 1H), 8.30 (d, *J* = 5.4 Hz, 1H), 10.86 (s, 1H). ^13^C NMR (126 MHz, DMSO-d6) δ ppm 7.71 (2C), 14.19, 33.31, 54.09, 55.59, 109.64, 115.67, 125.25, 135.22, 141.31, 148.19, 152.86, 172.81. Formula: C_14_H_17_N_3_O_3_S. MW: 307.37. LC-MS: *m*/*z* 308 [M + H]^+^.

#### *N*-(4-(1-(Propylsulfonyl)-2,5-dihydro-1*H*-pyrrol-3-yl)pyridin-2-yl)cyclopropanecarboxamide (**17**)

Following **GP1**, compound **17** was prepared using **3** (55 mg, 0.24 mmol), propane-1-sulfonyl chloride (27 μL, 0.24 mmol), TEA (100 μL, 0.72 mmol) in 2.5 mL DCM. Purification: flash chromatography (DCM/MeOH 92:8). Yield: 56 mg (70%), white solid. ^1^H NMR (500 MHz, CDCl_3_) δ ppm 0.93–1.01 (m, 2H), 1.08 (t, *J* = 7.4 Hz, 3H), 1.10–1.16 (m, 2H), 1.67–1.75 (m, 1H), 1.85–1.96 (m, 2H), 3.00–3.07 (m, 2H), 4.44 (td, *J* = 4.7, 2.3 Hz, 2H), 4.58 (td, *J* = 4.7, 1.7 Hz, 2H), 6.51 (t, *J* = 1.9 Hz, 1H), 7.04 (dd, *J* = 5.6, 1.3 Hz, 1H), 8.21 (d, *J* = 5.4 Hz, 1H), 8.31 (s, 1H), 9.60 (br s, 1H). ^13^C NMR (126 MHz, chloroform-d) δ ppm 9.16 (2C), 13.07, 16.03, 17.02, 51.85, 54.25, 55.71, 110.74, 115.70, 126.46, 135.57, 143.91, 144.99, 151.51, 173.37. Formula: C_16_H_21_N_3_O_3_S. MW: 335.42. LC-MS: *m*/*z* 336 [M + H]^+^.

#### *N*-(4-(1-(Cyclopentylsulfonyl)-2,5-dihydro-1*H*-pyrrol-3-yl)pyridin-2-yl)cyclopropanecarboxamide (**18**)

Following **GP1**, compound **18** was prepared using **3** (55 mg, 0.24 mmol), cyclopentanesulfonyl chloride (95 μL, 0.72 mmol), TEA (100 μL, 0.72 mmol) in 2.5 mL DCM. Purification: flash chromatography (DCM/MeOH 99:1), then preparative HPLC (5–50% MeCN gradient). Yield: 5 mg (6%), colorless oil. ^1^H NMR (500 MHz, CDCl_3_) δ ppm 0.97–1.03 (m, 2H), 1.12–1.17 (m, 2H), 1.60–1.69 (m, 2H), 1.78–1.88 (m, 3H), 1.99–2.13 (m, 4H), 3.56–3.64 (m, 1H), 4.47–4.53 (m, 2H), 4.60–4.66 (m, 2H), 6.59 (br s, 1H), 7.10 (br d, *J* = 5.2 Hz, 1H), 8.17 (d, *J* = 5.4 Hz, 1H), 8.39 (s, 1H), 10.49 (br s, 1H). ^13^C NMR (126 MHz, CHLOROFORM-d) δ ppm 9.64 (2C), 16.18, 25.67 (2C), 27.93 (2C), 54.53, 56.19, 61.49, 111.25, 115.58, 128.33, 135.15, 142.30, 145.65, 150.89, 173.99. Chemical Formula: C_18_H_23_N_3_O_3_S. MW: 361.46. LC-MS: *m*/*z* 362 [M + H]^+^.

### General Procedure for the Synthesis of Compounds **4** and **5** (**GP2**)

To a solution of carboxylic acid (1 equiv) in anhydrous DCM EDC hydrochloride (1.5 equiv) and DMAP (0.5 equiv) were added under Ar, and stirred in rt for 1 h. Then *N*-(4-(2,5-dihydro-1*H*-pyrrol-3-yl)pyridin-2-yl)cyclopropanecarboxamide (**3**) (1 equiv) was added and reaction mixture was stirred overnight. After that time, the mixture was extracted with DCM, combined organic layer was dried over anhydrous Na_2_SO_4_, filtered and concentrated under vacuum. The crude product was purified by flash chromatography.

#### *N*-(4-(1-(3-(1,3-Dioxoisoindolin-2-yl)propanoyl)-2,5-dihydro-1*H*-pyrrol-3-yl)pyridin-2-yl)cyclopropanecarboxamide (**4**)

Following **GP2**, compound **4** was prepared using 3-phthalimidopropionic acid (59 mg, 0.27 mmol), **3** (61 mg, 0.27 mmol), EDC hydrochloride (79 mg, 0.41 mmol) and DMAP (17 mg, 0.14 mmol) in 2 mL DCM. Purification: flash chromatography (DCM/MeOH 98:2). Yield: 29 mg (25%). ^1^H NMR (500 MHz, DMSO-*d*_6_) δ ppm 0.75–0.87 (m, 4H), 1.96–2.04 (m, 1H), 2.67–2.81 (m, 2H), 3.80–3.89 (m, 2H), 4.22–4.42 (m, 2H), 4.43–4.65 (m, 2H), 6.67 (br d, *J* = 2.0 Hz, 1H), 7.25 (ddd, *J* = 9.0, 5.3, 1.4 Hz, 1H), 7.82–7.89 (m, 4H), 8.07 (d, *J* = 12.9 Hz, 1H), 8.29 (dd, *J* = 5.3, 2.7 Hz, 1H), 10.84 (d, *J* = 5.4 Hz, 1H). Formula: C_24_H_22_N_4_O_4_. MW: 430.46.

#### *Tert*-Butyl (4-(3-(2-(Cyclopropanecarboxamido)pyridin-4-yl)-2,5-dihydro-1*H*-pyrrol-1-yl)-4-oxobutyl)carbamate (**5**)

Following **GP2**, compound **5** was prepared using 4-((*tert*-butoxycarbonyl)amino)butanoic acid (89 mg, 0.44 mmol), **3** (100 mg, 0.44 mmol), EDC hydrochloride (127 mg, 0.66 mmol) and DMAP (27 mg, 0.22 mmol) in 3 mL DCM. Purification: flash chromatography (DCM/MeOH 98:2 then 95:5). Yield: 120 mg (66%). ^1^H NMR (500 MHz, CDCl_3_) δ ppm 0.91–0.97 (m, 2H), 1.12 (td, *J* = 3.4, 2.3 Hz, 2H), 1.43 (s, 9H), 1.59–1.68 (m, 1H), 1.88–1.95 (m, 2H), 2.36 (br t, *J* = 7.0 Hz, 1H), 2.38–2.43 (m, 1H), 3.17–3.27 (m, 2H), 4.45 (br d, *J* = 2.0 Hz, 2H), 4.60 (br s, 2H), 4.86 (br s, 1H), 6.44–6.56 (m, 1H), 6.93–7.07 (m, 1H) 8.21 (s, 1H), 8.22–8.27 (m, 1H), 8.35 (s, 1H), 8.63–8.77 (m, 1H). Formula: C_22_H_30_N_4_O_4_. MW: 414.51.

### General Procedure for the Synthesis of Compounds **12**, **19**, and **20** (**GP3**)

To a solution of phthalimide-protected aliphatic amine derivative—**4**, **8** or **9** (1 equiv) dissolved in anhydrous EtOH, NH_2_NH_2_·H_2_O (3–6 equiv) was added and the mixture and stirred under reflux for 2 h. When all of starting material was consumed up, the solvent was evaporated under reduced pressure and crude product was purified by flash chromatography. After purification the compounds were dissolved in MeOH and transformed into solid hydrochloride salt by adding concentrated HCl_(aq)_ and removing the solvent in vacuo.

#### *N*-(4-(1-(3-Aminopropanoyl)-2,5-dihydro-1*H*-pyrrol-3-yl)pyridin-2-yl)cyclopropanecarboxamide Hydrochloride (**12**)

Following **GP3**, compound **12** was prepared using **4** (97 mg, 0.23 mmol), NH_2_NH_2_·H_2_O (33.5 μL, 0.69 mmol) in 2.5 mL EtOH. Purification: flash chromatography (DCM/MeOH/PE/NH_4_OH 7.84:1.35:0.54:0.27). Yield: 66 mg (87%), white solid. ^1^H NMR (500 MHz, deuterium oxide) δ ppm 1.02–1.15 (m, 4H), 1.78–1.95 (m, 1H), 2.82 (dt, *J* = 28.6, 6.0 Hz, 2H), 3.28 (br d, *J* = 5.2 Hz, 2H), 4.39–4.49 (m, 1H), 4.57 (br s, 2H), 4.70–4.72 (m, 1H), 6.94 (br d, *J* = 14.3 Hz, 1H), 7.24 (br s, 1H), 7.57 (t, *J* = 6.9 Hz, 1H), 8.19 (br d, *J* = 5.7 Hz, 1H). Protons of the −NH– and the −NH_3_^+^ groups were not detected. Rotamer 1: ^13^C NMR (126 MHz, deuterium oxide) δ ppm 10.05 (2C), 15.31, 30.26, 35.27, 52.03, 54.30, 111.08, 116.95, 132.20, 133.02, 137.04, 147.82, 149.63, 170.43, 178.35. Rotamer 2: ^13^C NMR (126 MHz, deuterium oxide) δ ppm 10.05 (2C), 15.31, 30.50, 35.27, 52.65, 54.90, 111.24, 117.01, 132.41, 133.09, 137.10, 147.82, 149.66, 170.43, 178.35. Formula: C_16_H_21_ClN_4_O_2_. MW: 336.82. LC-MS: *m*/*z* 301 [M + H]^+^.

### General Procedure for the Synthesis of Compounds **13**, **14**, **(*R*)-15**, and **(*S*)-15** (**GP4**)

To a solution of Boc-protected amine derivative—**5**, **6**, **(*S*)-7** or **(*R*)-7** (1 equiv) dissolved in MeOH, 37% HCl (5–10 equiv) was added and the mixture was stirred under reflux for 1 h. When all of starting material was consumed up, the pH was adjusted to 8 by addition of saturated aqueous K_2_CO_3_ solution and the solvents were evaporated under reduced pressure. The crude product was purified by different methods described below. After purification compounds were dissolved in MeOH and transformed into solid hydrochloride salt by adding concentrated HCl_(aq)_ and removing the solvent in vacuo.

#### *N*-(4-(1-(4-Aminobutanoyl)-2,5-dihydro-1*H*-pyrrol-3-yl)pyridin-2-yl)cyclopropanecarboxamide Hydrochloride (**13**)

Following **GP4**, compound **13** was prepared using **5** (160 mg, 0.39 mmol, 1 equiv), 37% HCl (320 μL, 3.9 mmol, 10 equiv) in 3 mL MeOH. Purification: to a dry residue Et_2_O and MeOH in a 1:1 ratio were added, then precipitated white solid was filtered and collected. Yield: 113 mg (84%), white solid. ^1^H NMR (500 MHz, methanol-*d*_4_) δ ppm 1.10–1.16 (m, 2H), 1.16–1.21 (m, 2H), 1.94–1.99 (m, 1H), 2.00–2.05 (m, 2H), 2.64 (dt, *J* = 42.8, 6.9 Hz, 2H), 3.06 (q, *J* = 8.0 Hz, 2H), 4.49–4.54 (m, 1H), 4.66 (s, 2H), 4.84 (dt, *J* = 3.7, 1.9 Hz, 1H), 7.11 (s, 1H), 7.40 (dd, *J* = 53.5, 1.7 Hz, 1H), 7.76 (dd, *J* = 6.7, 1.9 Hz, 1H), 8.28 (dd, *J* = 6.6, 2.0 Hz, 1H). Protons of the −NH– and the −NH_3_^+^ groups were not detected. Rotamer 1: ^13^C NMR (126 MHz, methanol-*d*_4_) δ ppm 10.81 (2C), 16.26, 23.55, 31.86, 40.57, 53.37, 55.63, 112.38, 117.95, 134.27, 135.15, 138.49, 150.10, 151.25, 172.82, 178.14. Rotamer 2: ^13^C NMR (126 MHz, methanol-*d*_4_) δ ppm 10.81 (2C), 16.26, 23.59, 32.22, 40.60, 53.94, 56.07, 112.38, 117.95, 134.27, 135.37, 138.55, 150.10, 151.34, 172.86, 178.16. Formula: C_17_H_23_ClN_4_O_2_. MW: 350.85. LC-MS: *m*/*z* 315 [M + H]^+^.

#### *Tert*-Butyl 2-(3-(2-(Cyclopropanecarboxamido)pyridin-4-yl)-2,5-dihydro-1*H*-pyrrole-1-carbonyl)pyrrolidine-1-carboxylate (**6**)

To a stirred solution of **3** (100 mg, 0.44 mmol, 1 equiv) in 3.5 mL anhydrous DCM, EDC hydrochloride (127 mg, 0.66 mmol, 1.5 equiv), DMAP (5 mg, 0.04 mmol, 0.1 equiv) and DIEA (158 μL, 0.88 mmol, 2 equiv) were added under Ar. After 5 min (*tert*-butoxycarbonyl)proline (95 mg, 0.44 mmol, 1 equiv) dissolved in a small amount of DCM was added dropwise. The reaction mixture was was stirred at rt overnight. After that time, solvent was evaporated under reduced pressure. Purification: flash chromatography (DCM/MeOH 96:4). Yield: 85 mg (46%). ^1^H NMR (500 MHz, CDCl_3_) δ ppm 0.87–0.96 (m, 2H), 1.06–1.13 (m, 2H), 1.30–1.46 (m, 9H), 1.60–1.74 (m, 1H), 1.79–1.99 (m, 2H), 2.02–2.26 (m, 2H), 2.90 (d, *J* = 37.5 Hz, 1H), 3.39–3.53 (m, 1H), 3.54–3.63 (m, 1H), 4.32–4.70 (m, 4H), 6.44–6.58 (m, 1H), 6.90–7.12 (m, 1H), 8.18 (s, 1H), 8.20–8.39 (m, 1H), 9.37 (br d, *J* = 152.6 Hz, 1H). Formula: C_23_H_30_N_4_O_4_. MW: 426.52.

### General Procedure for the Synthesis of Compounds **(*S*)-7** and **(*R*)-7** (**GP5**)

To a stirred solution of *N*-(4-(2,5-dihydro-1*H*-pyrrol-3-yl)pyridin-2-yl)cyclopropanecarboxamide (**3**) (1.1 equiv) dissolved in MeOH, aldehyde (1 equiv) and glacial acetic acid (catalytic amounts) were added. After 1 h the reaction mixture was cooled on an ice bath and NaBH_3_CN (3 equiv) was added. The reaction was stirred at r.t. overnight. After that time, solvent was evaporated under reduced pressure and residue was extracted with DCM. The combined organic layer was dried over anhydrous Na_2_SO_4_, filtered and concentrated under vacuum. The crude product was purified by different methods described below.

#### *Tert*-Butyl (*S*)-2-((3-(2-(Cyclopropanecarboxamido)pyridin-4-yl)-2,5-dihydro-1*H*-pyrrol-1-yl)methyl)pyrrolidine-1-carboxylate (**(*S*)-7**)

Following **GP5**, compound **(*S*)-7** was prepared using **3** (90 mg, 0.39 mmol), (*S*)-1-Boc-2-formylpyrrolidine (70 mg, 0.35 mmol), glacial acetic acid (6 μL), NaBH_3_CN (66 mg, 1.05 mmol) in 3 mL MeOH. Purification: flash chromatography (DCM/MeOH 92:8), then preparative HPLC (5–55% MeCN gradient). Yield: 36 mg (22%). ^1^H NMR (500 MHz, CDCl_3_) δ ppm 0.91 (dq, *J* = 7.6, 3.7 Hz, 2H), 1.06–1.12 (m, 2H), 1.45 (s, 9H), 1.71–1.77 (m, 1H), 1.83–1.94 (m, 2H), 1.97–2.22 (m, 2H), 2.95–3.10 (m, 1H), 3.24–3.44 (m, 3H), 3.88–4.16 (m, 2H), 4.20 (br d, *J* = 15.8 Hz, 1H), 4.30 (br d, *J* = 13.5 Hz, 1H), 4.47 (dd, *J* = 53.3, 13.2 Hz, 1H), 6.42 (s, 1H), 7.00 (dd, *J* = 5.4, 1.4 Hz, 1H), 8.12 (br d, *J* = 5.2 Hz, 1H), 8.26 (s, 1H), 8.29 (br s, 1H). Formula: C_23_H_32_N_4_O_3_. MW: 412.53.

#### *Tert*-Butyl (*R*)-2-((3-(2-(Cyclopropanecarboxamido)pyridin-4-yl)-2,5-dihydro-1*H*-pyrrol-1-yl)methyl)pyrrolidine-1-carboxylate (**(*R*)-7**)

Following **GP5**, compound **(*R*)-7** was prepared using **3** (177 mg, 0.77 mmol), (*R*)-1-Boc-2-formylpyrrolidine (132 μL, 0.7 mmol), glacial acetic acid (9 μL), NaBH_3_CN (132 mg, 2.1 mmol) in 4 mL MeOH. Purification: flash chromatography (DCM/PE/EtOAc/MeOH 50:20:27:3). Yield: 186 mg (58%). ^1^H NMR (500 MHz, CDCl_3_) δ ppm 0.90–0.94 (m, 2H), 1.08–1.12 (m, 2H), 1.46 (s, 9H), 1.60–1.67 (m, 1H), 1.91 (br d, *J* = 3.2 Hz, 2H), 2.08–2.26 (m, 2H), 2.39–2.54 (m, 1H), 3.06 (br s, 1H), 3.19–3.45 (m, 3H), 3.87–4.09 (m, 2H), 4.40–4.57 (m, 1H), 4.57–4.76 (m, 1H), 6.38 (s, 1H), 6.91–6.98 (m, 1H), 8.17 (br s, 1H), 8.20–8.27 (m, 1H), 8.76 (br s, 1H). Formula: C_23_H_32_N_4_O_3_. MW: 412.53.

#### *N*-(4-(1-Prolyl-2,5-dihydro-1*H*-pyrrol-3-yl)pyridin-2-yl)cyclopropanecarboxamide (**14**)

Following **GP4**, compound **14** was prepared using **6** (85 mg, 0.2 mmol), 37% HCl (82 μL, 1.0 mmol) in 2 mL MeOH. Purification: flash chromatography (DCM/MeOH/NH3_(aq)_ 96:4:0.4 then 92:8:0.8). Yield: 45 mg (69%), beige oil. ^1^H NMR (500 MHz, DMSO-*d*_6_) δ ppm 0.77–0.87 (m, 4H), 1.58–1.76 (m, 3H), 1.97–2.13 (m, 2H), 2.64–2.77 (m, 1H), 2.93–3.07 (m, 1H), 3.81 (ddd, *J* = 36.7, 8.0, 5.4 Hz, 1H), 4.23–4.37 (m, 1H), 4.37–4.84 (m, 3H), 6.68 (dt, *J* = 12.2, 1.9 Hz, 1H), 7.26 (ddd, *J* = 12.0, 5.3, 1.6 Hz, 1H), 8.10 (d, *J* = 6.9 Hz, 1H), 8.31 (dt, *J* = 5.7, 0.5 Hz, 1H), 10.85 (s, 1H). Rotamer 1: ^13^C NMR (126 MHz, DMSO-*d*_6_) δ ppm 7.69 (2C), 14.19, 26.09, 29.10, 46.96, 51.93, 53.41, 58.33, 109.36, 115.63, 124.96, 134.73, 141.56, 148.24, 152.82, 171.61, 172.78. Rotamer 2: ^13^C NMR (126 MHz, DMSO-*d*_6_) δ ppm 7.71 (2C), 14.19, 26.11, 29.13, 47.00, 52.72, 54.00, 58.50, 109.50, 115.75, 125.32, 135.40, 141.67, 148.27, 152.87, 171.69, 172.84. Formula: C_18_H_22_N_4_O_2_. MW: 326.40. LC-MS: *m*/*z* 327 [M + H]^+^.

#### (*R*)-*N*-(4-(1-(Pyrrolidin-2-ylmethyl)-2,5-dihydro-1*H*-pyrrol-3-yl)pyridin-2-yl)cyclopropanecarboxamide hydrochloride (**(*R*)-15**)

Following **GP4**, compound **(*R*)-15** was prepared using **(*R*)-7** (185 mg, 0.45 mmol), 37% HCl (369 μL, 4.5 mmol) in 4 mL MeOH. Purification: flash chromatography (DCM/MeOH/NH_3(aq)_ 95:5:0.5 then 9:1:0.1). Yield: 22 mg (14%), beige solid. ^1^H NMR (500 MHz, DMSO-*d*_6_) δ ppm 0.80–0.90 (m, 4H), 1.61–1.75 (m, 1H), 1.87 (dquin, *J* = 13.0, 8.2, 8.2, 8.2, 8.2 Hz, 1H), 1.93–2.01 (m, 1H), 2.01–2.07 (m, 1H), 2.18–2.28 (m, 1H), 3.21–3.32 (m, 2H), 3.69–3.77 (m, 1H), 3.80–3.91 (m, 1H), 3.96 (br s, 1H), 4.34–4.85 (m, 4H), 6.76 (s, 1H), 7.32 (br d, *J* = 4.9 Hz, 1H), 8.06 (s, 1H), 8.36 (d, *J* = 5.4 Hz, 1H), 9.52 (br s, 2H), 11.14 (s, 1H). ^13^C NMR (126 MHz, METHANOL-d4) δ ppm 10.83 (2C), 16.27, 24.36, 30.38, 47.43, 56.75, 57.83, 60.47, 62.96, 113.19, 118.15, 131.87, 134.69, 139.24, 149.32, 150.32, 178.12. Formula: C_18_H_25_ClN_4_O. MW: 348.88.

#### (*S*)-*N*-(4-(1-(Pyrrolidin-2-ylmethyl)-2,5-dihydro-1*H*-pyrrol-3-yl)pyridin-2-yl)cyclopropanecarboxamide Hydrochloride (**(*S*)-15**)

Following **GP4**, compound **(*S*)-15** was prepared using **(*S*)-7** (185 mg, 0.45 mmol), 37% HCl (369 μL, 4.5 mmol) in 4 mL MeOH. Purification: flash chromatography (DCM/MeOH/NH_3(aq)_ 95:5:0.5 then 9:1:0.1). Yield: 29 mg (19%), beige solid. ^1^H NMR (500 MHz, DMSO-d6) δ ppm 0.80–0.89 (m, 4H), 1.60–1.76 (m, 1H), 1.81–1.93 (m, 1H), 1.93–2.01 (m, 1H), 2.01–2.08 (m, 1H), 2.17–2.29 (m, 1H), 3.18–3.35 (m, 2H), 3.70–3.77 (m, 1H), 3.86 (br d, *J* = 12.0 Hz, 2H), 4.32 (br s, 1H), 4.53 (br s, 2H), 4.71 (br s, 1H), 6.76 (s, 1H), 7.32 (d, *J* = 4.6 Hz, 1H), 8.06 (s, 1H), 8.36 (d, *J* = 5.2 Hz, 1H), 9.52 (br s, 2H), 11.14 (s, 1H). ^13^C NMR (126 MHz, methanol-d4) δ ppm 10.77 (2C), 16.25, 24.36, 30.39, 47.42, 56.74, 57.88, 60.48, 62.98, 113.13, 118.16, 131.71, 134.73, 139.59, 149.07, 150.40, 178.03. Formula: C_18_H_25_ClN_4_O. MW: 348.88.

### General Procedure for the Synthesis of Compounds **8** and **9** (**GP6**)

*N*-(4-(2,5-Dihydro-1*H*-pyrrol-3-yl)pyridin-2-yl)cyclopropanecarboxamide (**3**) (1 equiv) was dissolved in anhydrous DCM, solution was cooled to 0 °C on an ice bath, then DIPEA (3 equiv) and appropriate sulfonyl chloride (1 equiv) were added. The reaction mixture was then warmed up to r.t. and stirred overnight. After that time, the mixture was extracted with DCM, combined organic layer was dried over anhydrous Na_2_SO_4_, filtered and concentrated under vacuum. The crude product was purified by flash chromatography.

#### *N*-(4-(1-((2-(1,3-Dioxoisoindolin-2-yl)ethyl)sulfonyl)-2,5-dihydro-1*H*-pyrrol-3-yl)pyridin-2-yl)cyclopropanecarboxamide (**8**)

Following **GP6**, compound **8** was prepared using 2-(1,3-dioxoisoindolin-2-yl)ethane-1-sulfonyl chloride (90 mg, 0.33 mmol), **3** (75 mg, 0.33 mmol), DIPEA (172 μL, 0.99 mmol) in 2 mL DCM. Purification: flash chromatography (DCM/MeOH 98:2). Yield: 57 mg (37%). ^1^H NMR (500 MHz, DMSO-*d*_6_) δ ppm 0.76–0.87 (m, 4H), 1.96–2.05 (m, 1H), 3.58 (t, *J* = 6.7 Hz, 2H), 4.00 (t, *J* = 6.7 Hz, 2H), 4.33 (br d, *J* = 2.0 Hz, 2H), 4.47 (br d, *J* = 3.2 Hz, 2H), 6.60 (t, *J* = 2.0 Hz, 1H), 7.22 (dd, *J* = 5.3, 1.6 Hz, 1H), 7.81–7.85 (m, 2H), 7.85–7.90 (m, 2H), 8.03 (s, 1H), 8.28 (d, *J* = 5.2 Hz, 1H), 10.85 (s, 1H). Formula: C_23_H_22_N_4_O_5_S. MW: 466.51.

#### *N*-(4-(1-((3-(1,3-Dioxoisoindolin-2-yl)propyl)sulfonyl)-2,5-dihydro-1*H*-pyrrol-3-yl)pyridin-2-yl)cyclopropanecarboxamide (**9**)

Following **GP6**, compound **9** was prepared using 3-(1,3-dioxoisoindolin-2-yl)propane-1-sulfonyl chloride (95 mg, 0.33 mmol), **3** (75 mg, 0.33 mmol), DIPEA (172 μL, 0.99 mmol) in 2 mL DCM. Purification: flash chromatography (DCM/MeOH 94:6) then MeOH was added to the residue and filtered, solid was collected. Yield: 51 mg (32.5%). ^1^H NMR (500 MHz, CDCl_3_) δ ppm 0.93–1.01 (m, 2H), 1.10–1.16 (m, 2H), 1.70 (br s, 1H), 2.21–2.31 (m, 2H), 3.14 (t, *J* = 7.6 Hz, 2H), 3.86 (t, *J* = 6.4 Hz, 2H), 4.46 (br s, 2H), 4.58 (br s, 2H), 6.50 (br s, 1H), 7.03 (br d, *J* = 4.9 Hz, 1H), 7.71–7.77 (m, 2H), 7.83–7.88 (m, 2H), 8.20 (br d, *J* = 5.2 Hz, 1H), 8.30 (s, 1H), 9.17–9.71 (m, 1H). Formula: C_24_H_24_N_4_O_5_S. MW: 480.54.

#### *N*-(4-(1-((2-Aminoethyl)sulfonyl)-2,5-dihydro-1*H*-pyrrol-3-yl)pyridin-2-yl)cyclopropanecarboxamide Hydrochloride (**19**)

Following **GP3**, compound **19** was prepared using **8** (52 mg, 0.11 mmol), NH_2_NH_2_·H_2_O (32 μL, 0.66 mmol) in 2 mL EtOH. Purification: flash chromatography (DCM/MeOH/NH_3(aq)_ 97:3:0.3 then 94:6:0.6), then the solid residue was washed with MeCN. Yield: 21 mg (51%), white solid. ^1^H NMR (500 MHz, DMSO-*d*_6_) δ ppm 0.83–0.92 (m, 4H), 2.01–2.08 (m, 1H), 3.17–3.25 (m, 2H), 3.59 (t, *J* = 7.3 Hz, 2H), 4.39 (br s, 2H), 4.53 (br d, *J* = 3.2 Hz, 2H), 6.76 (s, 1H), 7.38 (dd, *J* = 5.4, 1.1 Hz, 1H), 7.98 (s, 1H), 8.22 (br s, 3H), 8.31 (d, *J* = 5.4 Hz, 1H), 11.41 (br s, 1H). ^13^C NMR (126 MHz, DEUTERIUM OXIDE) δ ppm 10.07 (2C), 15.32, 33.94, 45.48, 53.68, 56.11, 111.18, 116.97, 132.22, 133.21, 137.51, 147.93, 149.05, 178.27. Formula: C_15_H_21_ClN_4_O_3_S. MW: 372.87. LC-MS: *m*/*z* 337 [M + H]^+^.

#### *N*-(4-(1-((3-Aminopropyl)sulfonyl)-2,5-dihydro-1*H*-pyrrol-3-yl)pyridin-2-yl)cyclopropanecarboxamide Hydrochloride (**20**)

Following **GP3**, compound **20** was prepared using **9** (51 mg, 0.11 mmol), NH_2_NH_2_·H_2_O (32 μL, 0.66 mmol) in 2 mL EtOH. Purification: flash chromatography (DCM/MeOH/NH_3(aq)_ 95:5:0.5 then 91:9:0.9), then the solid residue was washed with MeCN. Yield: 27 mg (66%), white solid. ^1^H NMR (500 MHz, deuterium oxide) δ ppm 1.09 (d, *J* = 6.3 Hz, 4H), 1.88 (quin, *J* = 6.2 Hz, 1H), 2.18 (quin, *J* = 7.6 Hz, 2H), 3.14 (br t, *J* = 7.7 Hz, 2H), 3.37 (t, *J* = 7.4 Hz, 2H), 4.43 (br d, *J* = 2.0 Hz, 2H), 4.51–4.61 (m, 2H), 6.91 (d, *J* = 2.0 Hz, 1H), 7.20 (d, *J* = 0.9 Hz, 1H), 7.55 (dd, *J* = 6.6, 1.4 Hz, 1H), 8.20 (d, *J* = 6.9 Hz, 1H). ^13^C NMR (126 MHz, deuterium oxide) δ ppm 10.17 (2C), 15.36, 20.95, 37.91, 45.38, 53.63, 56.10, 111.13, 116.99, 132.68, 133.23, 137.33, 147.86, 149.24, 178.32. Formula: C_16_H_23_ClN_4_O_3_S. MW: 372.87. LC-MS: *m*/*z* 351 [M + H]^+^.

#### *N*-(4-(4,4,5,5-Tetramethyl-1,3,2-dioxaborolan-2-yl)pyridin-2-yl)cyclopropanecarboxamide (**21**)

**1** (1.00 g, 4.15 mmol, 1 equiv) was dissolved in 10 mL anhydrous dioxane. Then bis(pinacolato)diboron (1.05 g, 4.15 mmol, 1 equiv) and potassium acetate (815 mg, 8.3 mmol, 2 equiv), were added, and under Ar Pd(dppf)Cl_2_ (307 mg, 0.42 mmol, 0.1 equiv). The mixture was stirred under reflux overnight. After that time, reaction mixture was cooled to room temperature, diluted with DCM, filter through Celite and evaporated under reduced pressure. The residue was dissolved in EtOAc, activated charcoal (2.5 g) was added and the mixture was stirred under reflux for 1 h. The mixture was then filtered again through Celite and evaporated under reduced pressure. Then, the solid residue was washed with hexane. Yield: 780 mg (65%). ^1^H NMR (500 MHz, CDCl_3_) δ ppm 0.83–0.90 (m, 2H), 1.07–1.12 (m, 2H), 1.30 (s, 12H), 1.55–1.64 (m, 1H), 7.34 (d, *J* = 4.6 Hz, 1H), 8.24 (d, *J* = 4.6 Hz, 1H), 8.55 (s, 1H), 8.95 (br s, 1H). Formula: C_15_H_21_BN_2_O_3_. MW: 288.15.

### General Procedure for the Synthesis of Compounds **22–26**, and **37** (**GP7**)

A mixture of *N*-(4-(4,4,5,5-tetramethyl-1,3,2-dioxaborolan-2-yl)pyridin-2-yl)cyclopropanecarboxamide (**21**) (1 equiv), appropriate aryl bromide (1 equiv) and K_2_CO_3_ (3 equiv) was dissolved in DMF, the resulting solution was flushed with argon for 5 min and then [1,1′-bis(diphenylphosphino)ferrocene]dichloropalladium(II) (0.2 equiv) was added. The reaction was stirred overnight at 80 °C. Then, it was cooled down to room temperature, diluted with ethyl acetate and filtered through Celite. The filtrate was concentrated in vacuo and purified by methods described below.

#### *N*-(4-(4-Cyanophenyl)pyridin-2-yl)cyclopropanecarboxamide (**22**)

Following **GP7**, compound **22** was prepared using *N*-(4-(4,4,5,5-tetramethyl-1,3,2-dioxaborolan-2-yl)pyridin-2-yl)cyclopropanecarboxamide (**21**) (104 mg, 0.36 mmol), 4-bromobenzonitrile (66 mg, 0.36 mmol), K_2_CO_3_ (149 mg, 1.08 mmol), Pd(dppf)Cl_2_ (50 mg, 0.07 mmol), DMF (3 mL). Purification: column chromatography (DCM/EtOAc 9:1). Yield: 72 mg (76%), white solid. Purity 100% (UPLC/MS). ^1^H NMR (500 MHz, CDCl_3_) δ ppm 9.71–9.99 (m, 1 H), 8.67 (s, 1 H), 8.33 (d, *J* = 5.4 Hz, 1 H), 7.76–7.82 (m, 4 H), 7.33 (d, *J* = 4.3 Hz, 1 H), 1.73–1.80 (m, 1 H), 1.13–1.18 (m, 2 H), 0.97–1.03 (m, 2 H). ^13^C NMR (CHLOROFORM-d, 126 MHz) δ 173.2, 152.1, 149.8, 146.7, 142.1, 132.8 (2C), 127.9 (2C), 118.3, 117.4, 113.1, 112.3, 15.9, 8.9 (2C). Formula: C_16_H_13_N_3_O; MS: *m*/*z* 264 (M+H^+^).

#### *N*-(4-(3-Cyanophenyl)pyridin-2-yl)cyclopropanecarboxamide (**23**)

Following **GP7**, compound **23** was prepared using *N*-(4-(4,4,5,5-tetramethyl-1,3,2-dioxaborolan-2-yl)pyridin-2-yl)cyclopropanecarboxamide (**21**) (144 mg, 0.50 mmol), 3-bromobenzonitrile (91 mg, 0.50 mmol), K_2_CO_3_ (207 mg, 1.50 mmol), Pd(dppf)Cl_2_ (69 mg, 0.09 mmol), DMF (4 mL). Purification: column chromatography (DCM/EtOAc 9:1). Yield: 93 mg (71%), light beige solid. Purity 99% (UPLC/MS). ^1^H NMR (500 MHz, CDCl_3_) δ ppm 9.40–9.78 (m, 1 H), 8.61 (s, 1 H), 8.34 (d, *J* = 5.2 Hz, 1 H), 7.87–7.99 (m, 2 H), 7.75 (d, *J* = 7.7 Hz, 1 H), 7.57–7.66 (m, 1 H), 7.30 (br d, *J* = 4.3 Hz, 1 H), 1.68–1.78 (m, 1 H), 1.13–1.18 (m, 2 H), 0.95–1.01 (m, 2 H). ^13^C NMR (CHLOROFORM-d, 126 MHz) δ 173.4, 151.7, 150.2, 145.7, 138.8, 133.0, 131.6, 130.7, 130.1, 118.1, 117.3, 113.5, 112.4, 16.0, 9.1 (2C). Formula: C_16_H_13_N_3_O; MS: *m*/*z* 264 (M+H^+^).

#### *N*-(6-Cyano-[3,4′-bipyridin]-2′-yl)cyclopropanecarboxamide (**24**)

Following **GP7**, compound **24** was prepared using *N*-(4-(4,4,5,5-tetramethyl-1,3,2-dioxaborolan-2-yl)pyridin-2-yl)cyclopropanecarboxamide (**21**) (200 mg, 0.69 mmol), 5-bromopicolinonitrile (126 mg, 0.69 mmol), K_2_CO_3_ (286 mg, 2.07 mmol), Pd(dppf)Cl_2_ (95 mg, 0.13 mmol), DMF (5 mL). Purification: column chromatography (DCM/EtOAc 8:2). Yield: 115 mg (63%), white solid. Purity 95% (UPLC/MS). ^1^H NMR (500 MHz, DMSO-*d*_6_) δ ppm 11.02 (s, 1 H), 9.09 (dd, *J* = 2.3, 0.9 Hz, 1 H), 8.47 (dd, *J* = 5.2, 0.9 Hz, 1 H), 8.44 (d, *J* = 0.9 Hz, 1 H), 8.37 (dd, *J* = 8.0, 2.3 Hz, 1 H), 8.19 (dd, *J* = 8.2, 0.7 Hz, 1 H), 7.54 (dd, *J* = 5.2, 1.7 Hz, 1 H), 2.00–2.08 (m, 1 H), 0.80–0.88 (m, 4 H). ^13^C NMR (DMSO-*d*_6_, 126 MHz) δ 173.0, 153.0, 149.4, 149.0, 144.6, 136.9, 136.1, 132.7, 129.3, 117.4, 117.3, 111.2, 14.2, 7.8 (2C). Formula: C_15_H_12_N_4_O; MS: *m*/*z* 265 (M+H^+^).

#### *N*-(2′-Cyano-[4,4′-bipyridin]-2-yl)cyclopropanecarboxamide (**25**)

Following **GP7**, compound **25** was prepared using *N*-(4-(4,4,5,5-tetramethyl-1,3,2-dioxaborolan-2-yl)pyridin-2-yl)cyclopropanecarboxamide (**21**) (200 mg, 0.69 mmol), 4-bromopyridine-2-carbonitrile (126 mg, 0.69 mmol), K_2_CO_3_ (286 mg, 2.07 mmol), Pd(dppf)Cl_2_ (95 mg, 0.13 mmol), DMF (5 mL). Purification: column chromatography (DCM/EtOAc 8:2). Yield: 130 mg (71%), pinkish solid. Purity 100% (UPLC/MS). ^1^H NMR (500 MHz, DMSO-*d*_6_) δ ppm 11.01 (s, 1 H), 8.84 (d, *J* = 5.2 Hz, 1 H), 8.43–8.48 (m, 2 H), 8.39 (d, *J* = 0.9 Hz, 1 H), 8.02 (dd, *J* = 5.2, 1.7 Hz, 1 H), 7.54 (dd, *J* = 5.2, 1.7 Hz, 1 H), 1.97–2.05 (m, 1 H), 0.77–0.85 (m, 4 H). ^13^C NMR (DMSO-*d*_6_, 126 MHz) δ 173.0, 153.1, 152.1, 149.1, 146.6, 144.6, 133.7, 126.7, 125.2, 117.4, 117.1, 111.0, 14.2, 7.8 (2C). Formula: C_15_H_12_N_4_O; MS: *m*/*z* 265 (M+H^+^).

#### *N*-(4-(5-Cyanothiophen-2-yl)pyridin-2-yl)cyclopropanecarboxamide (**26**)

Following **GP7**, compound **26** was prepared using *N*-(4-(4,4,5,5-tetramethyl-1,3,2-dioxaborolan-2-yl)pyridin-2-yl)cyclopropanecarboxamide (**21**) (144 mg, 0.50 mmol), 5-bromotiophene-2-carbonitrile (94 mg, 0.50 mmol), K_2_CO_3_ (207 mg, 1.50 mmol), Pd(dppf)Cl_2_ (69 mg, 0.09 mmol), DMF (4 mL). Purification: column chromatography (PE/EtOAc 6:4). Yield: 47 mg (35%), beige solid. Purity 98% (UPLC/MS). ^1^H NMR (500 MHz, DMSO-*d*_6_) δ ppm 11.02 (s, 1 H), 8.38–8.44 (m, 2 H), 8.06 (d, *J* = 4.0 Hz, 1 H), 7.86 (d, *J* = 4.0 Hz, 1 H), 7.48–7.52 (m, 1 H), 2.00–2.06 (m, 1 H), 0.81–0.88 (m, 4 H). ^13^C NMR (DMSO-*d*_6_, 126 MHz) δ ppm 173.1, 153.1, 149.2, 148.1, 140.4, 140.4, 126.9, 115.8, 114.0, 109.3, 108.9, 14.3, 7.9 (2C). Formula: C_14_H_11_N_3_OS; MS: *m*/*z* 270 (M+H^+^).

#### *N*-(4-(5-Phenylthiophen-2-yl)pyridin-2-yl)cyclopropanecarboxamide (**37**)

Following **GP7**, compound **37** was prepared using *N*-(4-(4,4,5,5-tetramethyl-1,3,2-dioxaborolan-2-yl)pyridin-2-yl)cyclopropanecarboxamide (**21**) (104 mg, 0.36 mmol), 2-bromo-5-phenylthiophene (86 mg, 0.36 mmol), K_2_CO_3_ (149 mg, 1.08 mmol), Pd(dppf)Cl_2_ (50 mg, 0.07 mmol), DMF (3 mL). Purification: column chromatography (DCM/MeOH 95:5). Yield: 87 mg (75%), white solid. Purity 100% (UPLC/MS). ^1^H NMR (500 MHz, DMSO-*d*_6_) δ ppm 10.91 (s, 1 H), 8.41 (s, 1 H), 8.32 (d, *J* = 5.2 Hz, 1 H), 7.72–7.77 (m, 3 H), 7.62 (d, *J* = 3.7 Hz, 1 H), 7.41–7.48 (m, 3 H), 7.33–7.39 (m, 1 H), 1.99–2.09 (m, 1 H), 0.78–0.91 (m, 4 H). ^13^C NMR (DMSO-*d*_6_, 126 MHz) δ 172.9, 153.0, 148.7, 144.9, 142.1, 139.7, 133.0, 129.3, 128.3, 127.6, 125.5, 125.3, 114.8, 108.5, 14.2, 7.8. Formula: C_19_H_16_N_2_OS; MS: *m*/*z* 321 (M+H^+^).

### General Procedure for the Synthesis of Compounds **27–31** (**GP8**)

To a stirred suspension of hydroxylamine hydrochloride (1.5 equiv) and corresponding nitrile in EtOH a NaHCO_3_ (1.5 equiv) was added. The reaction mixture was stirred under reflux for 6 h. After the reaction had completed, the reaction mixture was concentrated under reduced pressure, and the residue was purified using flash chromatography (5% MeOH in DCM).

#### *N-*(4-(4-(*N*′-Hydroxycarbamimidoyl)phenyl)pyridin-2-yl)cyclopropanecarboxamide (**27**)

Following **GP8**, compound **27** was prepared using *N*-(4-(4-cyanophenyl)pyridin-2-yl)cyclopropanecarboxamide (**22**) (65 mg, 0.25 mmol), hydroxylamine hydrochloride (26 mg, 0.38 mmol), NaHCO_3_ (31 mg, 0.38 mmol), EtOH (2 mL). Yield: 43 mg (58%), white solid. ^1^H NMR (500 MHz, DMSO-*d*_6_) δ ppm 10.82–10.94 (m, 1 H), 9.79 (s, 1 H), 8.33–8.45 (m, 2 H), 7.97–8.03 (m, 1 H), 7.76–7.83 (m, 2 H), 7.69–7.74 (m, 1 H), 7.40–7.48 (m, 1 H), 5.88 (s, 2 H), 1.97–2.06 (m, 1 H), 0.82–0.86 (m, 3 H), 0.63–1.03 (m, 1 H). Formula: C_16_H_16_N_4_O_2_; MS: *m*/*z* 297 (M+H^+^).

#### *N*-(4-(3-(*N*′-Hydroxycarbamimidoyl)phenyl)pyridin-2-yl)cyclopropanecarboxamide (**28**)

Following **GP8**, compound **28** was prepared using *N*-(4-(3-cyanophenyl)pyridin-2-yl)cyclopropanecarboxamide (**23**) (80 mg, 0.30 mmol), hydroxylamine hydrochloride (31 mg, 0.45 mmol), NaHCO_3_ (38 mg, 0.45 mmol), EtOH (2 mL). Yield: 39 mg (44%), white solid. ^1^H NMR (500 MHz, DMSO-*d*_6_) δ ppm 10.87–10.96 (m, 1 H), 9.75 (s, 1 H), 8.41–8.45 (m, 1 H), 8.37–8.42 (m, 1 H), 8.02 (t, *J* = 1.7 Hz, 1 H), 7.77 (dt, *J* = 8.0, 1.3 Hz, 1 H), 7.69–7.74 (m, 1 H), 7.52 (t, *J* = 7.7 Hz, 1 H), 7.43–7.48 (m, 1 H), 5.96 (s, 1 H), 2.00–2.08 (m, 1 H), 0.78–0.88 (m, 4 H). Formula: C_16_H_16_N_4_O_2_; MS: *m*/*z* 297 (M+H^+^).

#### *N*-(6-(*N′*-Hydroxycarbamimidoyl)-[3,4′-bipyridin]-2′-yl)cyclopropanecarboxamide (**29**)

Following **GP8**, compound **29** was prepared using *N*-(6-cyano-[3,4′-bipyridin]-2′-yl)cyclopropanecarboxamide (**24**) (88 mg, 0.33 mmol), hydroxylamine hydrochloride (35 mg, 0.50 mmol), NaHCO_3_ (42 mg, 0.50 mmol), EtOH (2.5 mL). Yield: 78 mg (79%), white solid. ^1^H NMR (500 MHz, DMSO-*d*_6_) δ ppm 10.93 (s, 1 H), 10.05 (s, 1 H), 8.83–8.86 (m, 1 H), 8.37–8.39 (m, 2 H), 8.07–8.09 (m, 1 H), 7.93 (dd, *J* = 8.3, 0.9 Hz, 1 H), 7.45–7.47 (m, 1 H), 5.86 (br s, 2 H), 1.99–2.06 (m, 1 H), 0.77–0.81 (m, 4 H). Formula: C_15_H_15_N_5_O_2_; MS: *m*/*z* 298 (M+H^+^).

#### *N*-(2′-(*N′*-Hydroxycarbamimidoyl)-[4,4′-bipyridin]-2-yl)cyclopropanecarboxamide (**30**)

Following **GP8**, compound **30** was prepared using *N*-(2′-cyano-[4,4′-bipyridin]-2-yl)cyclopropanecarboxamide (**25**) (100 mg, 0.38 mmol), hydroxylamine hydrochloride (40 mg, 0.57 mmol), NaHCO_3_ (48 mg, 0.57 mmol), EtOH (3 mL). Yield: 90 mg (80%), white solid. ^1^H NMR (500 MHz, DMSO-*d*_6_) δ ppm 11.02 (s, 1 H), 10.07 (s, 1 H), 8.70 (d, *J* = 5.2 Hz, 1 H), 8.51 (d, *J* = 0.9 Hz, 1 H), 8.46 (d, *J* = 5.2 Hz, 1 H), 8.15 (d, *J* = 1.1 Hz, 1 H), 7.78 (dd, *J* = 5.3, 1.9 Hz, 1 H), 7.52 (dd, *J* = 5.2, 1.7 Hz, 1 H), 5.93 (s, 2 H), 2.00–2.08 (m, 1 H), 0.77–0.90 (m, 4 H). Formula: C_15_H_15_N_5_O_2_; MS: *m*/*z* 298 (M+H^+^).

#### *N*-(4-(5-(*N*′-Hydroxycarbamimidoyl)thiophen-2-yl)pyridin-2-yl)cyclopropanecarboxamide (**31**)

Following **GP8**, compound **31** was prepared using *N*-(4-(5-cyanothiophen-2-yl)pyridin-2-yl)cyclopropanecarboxamide (**26**) (77 mg, 0.29 mmol), hydroxylamine hydrochloride (31 mg, 0.44 mmol), NaHCO_3_ (37 mg, 0.44 mmol), EtOH (2 mL). Yield: 66 mg (75%), white solid. ^1^H NMR (500 MHz, DMSO-*d*_6_) δ ppm 10.88 (s, 1 H), 9.80 (s, 1 H), 8.33–8.36 (m, 1 H), 8.30 (d, *J* = 5.7 Hz, 1 H), 7.63 (d, *J* = 3.7 Hz, 1 H), 7.52 (d, *J* = 4.0 Hz, 1 H), 7.37 (dd, *J* = 5.4, 1.7 Hz, 1 H), 6.03 (s, 2 H), 2.02 (tt, *J* = 7.6, 4.9 Hz, 1 H), 0.78–0.87 (m, 4 H). Formula: C_14_H_14_N_4_O_2_S; MS: *m*/*z* 303 (M + H^+^).

### General Procedure for the Synthesis of Compounds **32–36** (**GP9**)

The appropriate hydroxycarbamimidoyl derivative was dissolved in trimethyl orthoformate containing BF_3_·Et_2_O and the resulting solution was heated to 55 °C for 30 min. After that time the mixture was concentrated under reduced pressure and the solid residue was purified using flash chromatography (0–5% MeOH in DCM).

#### *N*-(4-(4-(1,2,4-Oxadiazol-3-yl)phenyl)pyridin-2-yl)cyclopropanecarboxamide (**32**)

Following **GP9**, compound **32** was prepared using *N-*(4-(4-(*N’*-hydroxycarbamimidoyl)phenyl)pyridin-2-yl)cyclopropanecarboxamide (**27**) (40 mg, 0.13 mmol), BF_3_·Et_2_O (14 μL), trimethyl orthoformate (1 mL). Yield: 17 mg (43%), white solid. Purity 100% (UPLC/MS). ^1^H NMR (500 MHz, DMSO-*d*_6_) δ ppm 10.92 (s, 1 H), 9.73 (s, 1 H), 8.44 (d, *J* = 0.9 Hz, 1 H), 8.38 (d, *J* = 5.2 Hz, 1 H), 8.12–8.18 (m, 2 H), 7.86–7.92 (m, 2 H), 7.44 (dd, *J* = 5.3, 1.6 Hz, 1 H), 1.95–2.06 (m, 1 H), 0.75–0.84 (m, 4 H). ^13^C NMR (DMSO-*d*_6_, 126 MHz) δ 172.9, 167.6, 166.4, 153.0, 148.8, 148.0, 140.6, 128.0 (2C), 127.7 (2C), 126.5, 116.9, 110.7, 14.2, 7.8 (2C). Formula: C_17_H_14_N_4_O_2_; MS: *m*/*z* 307 (M+H^+^).

#### *N*-(4-(3-(1,2,4-Oxadiazol-3-yl)phenyl)pyridin-2-yl)cyclopropanecarboxamide (**33**)

Following **GP9**, compound **33** was prepared using *N-*(4-(4-(*N’*-hydroxycarbamimidoyl)phenyl)pyridin-2-yl)cyclopropanecarboxamide (**28**) (35 mg, 0.12 mmol), BF_3_·Et_2_O (14 μL), trimethyl orthoformate (1 mL). Yield: 18 mg (49%), white solid. Purity 98% (UPLC/MS). ^1^H NMR (500 MHz, DMSO-*d*_6_) δ ppm 10.93 (s, 1 H), 9.74 (s, 1 H), 8.44 (d, *J* = 0.9 Hz, 1 H), 8.39 (d, *J* = 5.7 Hz, 1 H), 8.27 (t, *J* = 1.7 Hz, 1 H), 8.08–8.14 (m, 1 H), 7.90–7.95 (m, 1 H), 7.71 (t, *J* = 7.7 Hz, 1 H), 7.45 (dd, *J* = 5.4, 1.7 Hz, 1 H), 1.96–2.06 (m, 1 H), 0.76–0.85 (m, 4 H). ^13^C NMR (DMSO-*d*_6_, 126 MHz) δ 173.0, 167.7, 166.5, 153.0, 148.8, 148.1, 138.7, 130.4, 130.0, 127.8, 126.8, 125.1, 117.0, 110.6, 14.3, 7.8 (2C). Formula: C_17_H_14_N_4_O_2_; MS: *m*/*z* 307 (M+H^+^).

#### *N*-(6-(1,2,4-Oxadiazol-3-yl)-[3,4′-bipyridin]-2′-yl)cyclopropanecarboxamide (**34**)

Following **GP9**, compound **34** was prepared using *N*-(6-(*N′*-hydroxycarbamimidoyl)-[3,4′-bipyridin]-2′-yl)cyclopropanecarboxamide (**29**) (98 mg, 0.33 mmol), BF_3_·Et_2_O (38 μL), trimethyl orthoformate (2.75 mL). Yield: 27 mg (27%), white solid. Purity 99% (UPLC/MS). ^1^H NMR (500 MHz, DMSO-*d*_6_) δ ppm 10.97 (s, 1 H), 9.79 (s, 1 H), 9.08 (dd, *J* = 2.3, 0.9 Hz, 1 H), 8.41–8.47 (m, 2 H), 8.29–8.34 (m, 1 H), 8.19–8.24 (m, 1 H), 7.53 (dd, *J* = 5.2, 1.7 Hz, 1 H), 1.97–2.06 (m, 1 H), 0.77–0.86 (m, 4 H). ^13^C NMR (DMSO-*d*_6_, 126 MHz) δ 173.0, 167.9, 166.6, 153.0, 148.9, 148.5, 145.8, 145.3, 135.9, 135.3, 123.7, 117.1, 110.9, 14.3, 7.8 (2C). Formula: C_16_H_13_N_5_O_2_; MS: *m*/*z* 308 (M+H^+^).

#### *N*-(2′-(1,2,4-Oxadiazol-3-yl)-[4,4′-bipyridin]-2-yl)cyclopropanecarboxamide (**35**)

Following **GP9**, compound **35** was prepared using *N*-(2′-(*N′*-hydroxycarbamimidoyl)-[4,4′-bipyridin]-2-yl)cyclopropanecarboxamide (**30**) (85 mg, 0.29 mmol), BF_3_·Et_2_O (34 μL), trimethyl orthoformate (2.5 mL). Yield: 35 mg (39%), white solid. Purity 100% (UPLC/MS). ^1^H NMR (500 MHz, DMSO-*d*_6_) δ ppm 11.05 (s, 1 H), 9.84 (s, 1 H), 8.90–8.93 (m, 1 H), 8.53–8.56 (m, 1 H), 8.48–8.51 (m, 1 H), 8.33 (dd, *J* = 1.7, 0.9 Hz, 1 H), 7.97 (dd, *J* = 5.0, 1.9 Hz, 1 H), 7.59 (dd, *J* = 5.2, 1.7 Hz, 1 H), 2.02–2.09 (m, 1 H), 0.80–0.90 (m, 4 H). ^13^C NMR (DMSO-*d*_6_, 126 MHz) δ 173.6, 168.4, 167.3, 153.7, 152.0, 149.7, 147.1, 146.9, 146.1, 124.1, 121.1, 117.5, 111.2, 14.8, 8.4 (2C). Formula: C_16_H_13_N_5_O_2_; MS: *m*/*z* 308 (M+H^+^).

#### *N*-(4-(5-(1,2,4-Oxadiazol-3-yl)thiophen-2-yl)pyridin-2-yl)cyclopropanecarboxamide (**36**)

Following **GP9**, compound **36** was prepared using *N*-(4-(5-(*N*′-hydroxycarbamimidoyl)thiophen-2-yl)pyridin-2-yl)cyclopropanecarboxamide (**31**) (52 mg, 0.17 mmol), BF_3_·Et_2_O (14 μL), trimethyl orthoformate (1 mL). Yield: 14 mg (26%), white solid. Purity 100% (UPLC/MS). ^1^H NMR (500 MHz, DMSO-*d*_6_) δ ppm 10.94 (s, 1 H), 9.72 (s, 1 H), 8.41 (d, *J* = 0.9 Hz, 1 H), 8.34 (d, *J* = 6.0 Hz, 1 H), 7.85 (d, *J* = 4.0 Hz, 1 H), 7.81 (d, *J* = 3.7 Hz, 1 H), 7.46 (dd, *J* = 5.2, 1.7 Hz, 1 H), 1.96–2.04 (m, 1 H), 0.75–0.87 (m, 4 H). ^13^C NMR (DMSO-*d*_6_, 126 MHz) δ 173.1, 167.7, 162.5, 153.1, 149.0, 144.5, 141.2, 131.4, 128.0, 127.4, 115.4, 109.0, 14.3, 7.8 (2C). Formula: C_15_H_12_N_4_O_2_S; MS: *m*/*z* 313 (M+H^+^).

### Crystallography

#### Plasmid Construction

For bacterial expression, the fragment of the gene encoding kinase domain of GSK-3β (26–383) was codon optimized and synthesized by Genscript, and then the gene was cloned into a pET24a expression plasmid. The kinase domain of GSK-3β was expressed with a C-terminal hexahistidine tag and proceeded with the tobacco etch virus protease cleavage site (ENLYFQ*GHHHHHH).

#### Protein Expression and Purification

GSK-3β was expressed in *E. coli* LOBSTR strain (Kerafast) in media for autoinduction ZYM-5052^64^ (1% tryptone, 0.5% yeast extract, 25 mM Na_2_HPO_4_, 25 mM KH_2_PO_4,_ 50 mM NH_4_Cl, 5 mM Na_2_SO_4,_ 0.5% glycerol, 0.05% glucose, 0.2% α-lactose, 2 mM MgSO_4_) supplemented with kanamycin (50 μg/mL) at 17 °C for 16 h. The pellet was resuspended in cold lysis buffer (20 mM HEPES pH 7.2, 500 mM NaCl, 5% glycerol, 15 mM imidazole, and 5 mM 2-mercaptoethanol) and the cells were disintegrated by sonication. Clarified lysate was passed through HisPur Cobalt resin (Thermo Fisher Scientific, Waltham, MA, United States), and the protein of interest was eluted with stepwise increments of imidazole concentration (50–300 mM). The fraction corresponding to GSK-3β was pulled and dialyzed against 20 mM HEPES, pH 7.2, containing 300 mM NaCl, 10 mM MgCl_2_, and 5 mM 2-mercaptoethanol. His tag was removed by TEV protease cleavage during dialysis. Further purification was obtained by size exclusion chromatography on a HiLoad 16/600 Superdex 75 pg column (Cytiva) in 20 mM HEPES, pH 7.2, containing 300 mM NaCl and 5 mM 2-mercaptoethanol. Purified GSK-3β kinase was flash-frozen in liquid nitrogen and stored at −80 °C for further analysis.

#### Protein Crystallization, Data Collection, and Structure Determination

For crystallization, GSK-3β was concentrated to 6–8 mg/mL. The protein was incubated with 5–10 molar excess of **36** at 20 °C. The preparation was mixed 1:1 (v/v) with the crystallization solutions. Crystallization experiments were carried out at 4 and 20 °C. Crystals appeared within 2–4 days at room temperature. The GSK-3β/**36** complex (PDB ID: 8QJI) was obtained in 0.1 M MES, pH 6.5, 12% w/v PEG 20000. Crystals were cryoprotected with mother liquor containing 25% glycerol and cryocooled in liquid nitrogen. The diffraction data were collected at ESRF (Grenoble).^65^

The diffraction data was indexed and integrated in XDS.^66^ Data was scaled in AIMLESS^67^ from the CCP4 software package.^68^ The following steps were performed in Phenix.^69^ The protein crystallized with 1 copy in the asymmetric unit. The structure of GSK-3β was solved by molecular replacement using PHASER^70^ and 6Y9S as a search model. Models were refined by interchanging cycles of automated refinement using phenix.refine^71^ and manual building in Coot.^72^ Data collection and refinement statistics are summarized in Table S1 in the SI.

#### Dye-Based Thermal Shift Assay

GSK-3β kinase stability in the presence of **36** and **11** was analyzed by the proteins’ melting temperatures determination using Thermal Shift Assay (TSA) as described previously.^73^ The protein (1.5 mg/mL) was incubated with 1:200 diluted Sypro Orange dye in 20 mM HEPES, 100 mM KCl, 10 mM MgCl_2_, 1 mM 2-mercaptoethanol, pH 8.0, and compound (10 μM) or DMSO. The fluorescence signal of Sypro Orange was determined as a function of temperature between 5 and 95 °C in increments of 0.5 °C min^–1^ (λ_ex_ 492, λ_em_ 610 nm). The melting temperature was calculated as the inflection point of the fluorescence as a function of temperature. The experiment was carried out in triplicates.

### GSK-3β Kinase Activity Assay

The inhibitory activities of the tested compounds against the GSK-3β human recombinant kinase were measured using Promega’s GSK-3β Kinase Enzyme System (Promega; Madison, WI, USA), according to the provided manufacturer’s instruction, using the low-volume white polystyrene 384-well plates. The ADP-Glo Assay (Promega; Madison, WI, USA) was used for bioluminescent detection of the kinase activity. Tested compounds were prepared as 1 mM stock solutions in DMSO and diluted with the Kinase Assay Buffer before use (40 mM Tris, pH 7.5, enriched with 50 μM dithiothreitol; DTT) to obtain the desired compounds’ concentrations. The kinase enzyme, GSK-3β-substrate (derived from human muscle glycogen synthase 1), and ATP were also diluted in the assay buffer before use. At first, GSK-3β (10 ng per well) was incubated with the tested sample (10 μM in well) for 5 min. In the case of blank wells, the DMSO solution (1% in well) was used instead of the target samples’ solutions. After the incubation period, ATP (25 μM in well) and GSK-3β-substrate (0.2 μg/μL in well) were added to start the enzymatic reaction. The reaction mixture was kept at room temperature for 1 h, followed by the addition of the ADP-Glo reagent, to terminate the kinase reaction and deplete any remaining ATP. After the following 40 min—a second reagent (Kinase Detection Reagent) was applied to convert the obtained ADP to ATP and to generate light from the newly synthesized ATP using a luciferase/luciferin reaction. The mixture was kept for another 30 min at room temperature, then the luminescence was measured. Based on equation 100 – (*S*/*B*) × 100 (where *S* and *B* were the respective enzyme activities with and without the tested sample, respectively) the percent of inhibition of GSK-3β for each compound was calculated. Compounds with enzyme inhibitory activities at 10 μM better than 50% were further evaluated to obtain IC_50_ values. The IC_50_ values were determined based on the kinase’s inhibitory activities in the six to seven different concentrations of each compound, resulting in inhibition between 5% and 95%. Calculations were made using nonlinear regression (GraphPad Prism 9; GraphPad Software, San Diego, CA, USA) by plotting the residual enzyme activities against the applied inhibitor concentration. Staurosporine (Biokom, Janki, Poland) was used as the reference compound. Each data point was collected in triplicate.

### Kinetics of GSK-3β Inhibition by **36**

The GSK-3β Kinase Enzyme System (Promega; Madison, WI, USA) and ADP-Glo bioluminescent assay (Promega; Madison, WI, USA) were used in kinetic studies. The assay procedures were followed according to the provided manufacturer’s instructions. The general workflow is described in Section GSK-3β kinase activity assay. The luminescence was measured using the EnSpire multimode microplate reader (PerkinElmer, Waltham, MA, USA). Five diverse inhibitor concentrations were tested, giving the enzyme inhibition between 10 and 90%. For each concentration of the inhibitor, ATP was added at concentrations of 100, 50, 25, and 10 μM in the wells. Each data point was collected in triplicate. Vmax and Km values of the Michaelis–Menten kinetics were calculated using nonlinear regression from substrate–velocity curves. Lineweaver–Burk and Cornish–Bowden plots were obtained by linear regression in GraphPad Prism (GraphPad Prism 9; GraphPad Software, San Diego, CA, USA). The *K*_i_ value of inhibitor 36 was obtained from a replot of the Lineweaver–Burk plots data (*K*_m_ versus [*I*]).

### Computational Studies

The GSK-3β/**36** protein crystal structure has been prepared in the Maestro suite, Schrödinger Release 2023-2: Maestro, Schrödinger, LLC, New York, NY, 2023. The structure has been protonated with Epik and propka and then minimized with the OPLS4 force field. Missing residues were added with Prime.^51^ The crystal structure 8QJI served as a protein model. To recover possible crystal flaws, compound **36** was redocked to the 8QJI protein structure using the Induced Fit Docking procedure and distinct oxadiazole orientation has been obtained. Ligands were docked with Glide, with two constraints applied on hydrogen bond formation with the Val135 main chain in the hinge region. Poses generated by this protocol were used for further quantum mechanical analysis of SAR data. The pockets were cut manually and prepared to ensure their chemical consistency to the full protein systems, based on prior experience.^45^ The quantum mechanical analysis of the SAR data was performed with ULYSSES.^74^ We made use of GFN2-xTB^75^ together with ALPB solvation.^76^ The GFN2-xTB method was benchmarked against GSK-3β in a previous study and proved effective.^45^ Ligand-residue pairs were prepared with in-pocket optimization^45^ to relax the positions of hydrogen atoms. In the case of the **36/**GSK-3β complex, our analysis was performed using residues Asp133, Tyr134, Val135, and Pro136 of the hinge region/adenine binding region; Cys199 and Val70; Phe67, which forms the hydrophobic region of the binding pocket; Asp200 from the DFG motif; and Lys85 and Glu97 of the phosphate binding region. The energy decomposition and deconvolution analysis were performed with our algorithm, implemented in ULYSSES.^45^ Molecular graphics and analyses were performed with UCSF ChimeraX 1.7.1, developed by the Resource for Biocomputing, Visualization, and Informatics at the University of California, San Francisco, with support from National Institutes of Health R01-GM129325 and the Office of Cyber Infrastructure and Computational Biology, National Institute of Allergy and Infectious Diseases.^77,78^

To bridge the results of the quantum chemical calculations with experimental data we made use of a simple statistical mechanical model which is summarized in the equation below:^79^
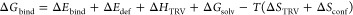
1Here, Δ*E*_bind_ is the gas phase binding energy for the system, Δ*E*_def_ contains the protein and ligand deformation energies (though one assumes that only the ligand pays deformation penalties), Δ*H*_TRV_ is the translation–rotation–vibrational contribution to enthalpy, Δ*G*_solv_ contains the solvation terms for all species involved, Δ*S*_TRV_ is the translation-rotation-vibrational entropy penalty (or entropy of binding) and Δ*S*_conf_ accounts for the loss of conformational freedom of the ligand and protein upon binding. Together, Δ*E*_bind_ + Δ*E*_def_ + Δ*H*_TRV_ make the gas phase enthalpy of binding. The EDDA calculations account for the contributions of binding energy and solvation Gibbs free energy (Δ*E*_bind_ + Δ*G*_solv_). Note that these are the main factors typically leading to the stabilization of the protein–ligand complex, motivating the basis for the analysis.

From a formal perspective, the accurate determination of the binding Gibbs energies (Δ*G*_bind_) requires extensive sampling. This is particularly critical in evaluating entropies, as these are not a simple average over all available conformers.^74^ This problem is mitigated by the calculation of conformational entropies, which was performed with CREST.^80^ However, the multiconformer model underlying eq 1 estimates all enthalpic terms as Boltzmann averages over all conformers of the ligand, protein, and ligand–protein complex. Consequently, for the EDDA analysis to be meaningful it is sufficient to use representative conformations of the bound complex. Note that in this model the conversion of free molecules to their binding modes is accomplished by the deformation energy term. Also note that the expression used does not lose validity if the protein undertakes significant conformational changes upon binding, since these terms are also accounted in the deformation energy of the protein.

Since several of the terms in eq 1 are omitted, the calculation of absolute Gibbs free energies is not meaningful. Avoiding the calculation of absolute Gibbs energies brings additional advantages regarding the use of single binding poses, as additional errors are potentially canceled. Furthermore, an exact comparison between experimental and calculated affinities is only possible if *K*_d_ values are available (which is not the case in the present work). Comparison with experimental data is consequently best performed using relative data. Here we use the standard text-book expression to convert between calculated and experimental data.
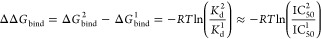
2DLPNO-CCSD(T) and DFT calculations (B3LYP, PBE, r2SCAN-3C, wB97X) on a reduced structural model were run with ORCA 5.0.4.^81−83^ All calculations made use of the def2-TZVP basis^84^ set along with the resolution of the identity approximation. Calculations in solution were run with the CPCM implicit solvation model.^85,86^ Grimme’s D3 dispersion correction was used along with the B3LYP and PBE methods.

### BV-2 and HT-22 Cell Lines-Based Assays

#### Cells Preparation

Mouse microglial cells (BV-2) were a generous gift from Professor Bozena Kaminska-Kaczmarek of the Laboratory of Molecular Neurobiology, Neurobiology Center, Nencki Institute of Experimental Biology, Polish Academy of Sciences, Warsaw, Poland. Cells were cultured in Dulbecco’s modified Eagle’s Medium-high glucose (DMEM, Glutamax Thermo Fisher) supplemented with 10% heat-inactivated fetal bovine serum (Thermo Fisher), 100 IU/mL penicillin (Merck) and 100 μg/mL streptomycin (Merck). Cells were cultured in flasks (area 175 cm^2^, Nunc), and incubated at 37 °C, 5% CO_2_. To evaluate the level of NO, IL-6, and TNF-α and the effectiveness of the compounds tested, BV-2 microglia cells were cultured in a 96-well culture plate (5 × 10^4^ cells per well, Falcon). For the measurement of cell viability and cell membrane damage, cells were placed in a 96-well culture plate (2 × 10^4^ cells per well, Falcon). Before the tests, cells were grown for 24 h in the incubator (37 °C, 5% CO_2_).

Mouse Hippocampal Neuronal Cell Line (HT-22) was a generous gift from Dr Bartosz Pomierny of the Department of Biochemical Toxicology, Jagiellonian University Medical College, Krakow, Poland. Cells were cultured in Dulbecco’s modified Eagle’s Medium—high glucose (DMEM, Glutamax Thermo Fisher) supplemented with 10% heat-inactivated fetal bovine serum (Thermo Fisher), 100 IU/mL penicillin (Merck) and 100 μg/mL streptomycin (Merck). Cells were cultured in flasks (area 175 cm^2^, Nunc), and incubated at 37 °C, 5% CO_2_. For the measurement of cell viability and neuroprotective effect against okadaic acid cells were placed in a 96-well culture plate (2 × 10^4^ cells per well, Falcon). Before the tests, cells were grown for 24 h in the incubator (37 °C, 5% CO_2_).

#### Preparation of Stock Solutions of Tested Compounds

Stock solutions were prepared at a concentration of 10 mM for the test and reference compounds. A minimum of 1 mg of each tested compound was weighed and dissolved in the appropriate volume of dimethyl sulfoxide. Serial dilutions were prepared in DMSO and then the diluted compounds were transferred to PBS. Before assays eventual precipitation or opalescence was checked.

#### Cell Viability Assay

Cell viability was evaluated using the PrestoBlue reagent (ThermoFisher), according to the manufacturer’s procedures.^87^ Following 24 h of incubation with the tested molecule, PrestoBlue reagent was added to a microplate well in an amount equal to one-tenth of the remaining medium volume. The resulting mixture was incubated for 15 min at 37 °C, and the fluorescence intensity (EX 530 nm; EM 580 nm) was measured in the plate reader POLARstar Omega, (BMG Labtech). The results (viability values) are provided as a percentage of live cells with respect to DMSO (control sample).

#### Okadaic Acid Treated HT-22 Cells

HT-22 cells were treated with okadaic acid (Merck): 400 nM for 3 h. After this time, the 10, 1, and 0.1 μM of tested compounds or DMSO were added and incubated in the aseptic condition (37 °C, 5% CO_2_). Cell viability was determined by Presto Blue assay after 24 h.

#### LPS-Treated BV-2 Cells

The cells were pretreated with tested compounds for 1 h. After this time lipopolysaccharide (100 ng/mL) was added and the resulting mixture was incubated for 18h. Next, the culture supernatant was acquired to measure the levels of nitric oxide (NO), IL-6 and TNF-α according to the following procedures.

#### NO Release Measurement

The NO level in the culture supernatants was measured using 2,3-diaminonaphthalene (DAN) reagent according to the method of Nussler et al.^88^ After 15 min of incubation at room temperature, the fluorescence intensity (EX 360; EM 440 nm) was measured using a microplate reader POLARstar Omega, (BMG Labtech). The values of nitric oxide were calculated as a percentage of control (maximal response of LPS).

#### Measurement of Cytokine Levels

The IL-6 and TNF-α levels in the culture supernatants were measured using LANCE Ultra TR-FRET Detection Kit (PerkinElmer), according to manufacturer protocol. Each cytokine detection was performed separately in a 384-well plate following the kit instructions. Samples were added at 15 μL/well to a 384-well plate and then premixed antibody solution was added at 5 μL/well. After 1 h of incubation of IL-6 and 3h for incubation of TNF-α in the dark, at 22 °C, the plates were read with an EnVision plate reader (PerkinElmer) with the excitation wavelength at 320 nm, the donor emission at 615 nm, and the acceptor emission at 660 nm. The values of IL-6 and TNF-α were calculated as a percentage of control (maximal response of LPS).

#### Statistical Analysis

Statistical analysis was performed using the program GraphPad Prism 9.0.0. All values are expressed as mean with SD. Differences among groups were evaluated by one-way ANOVA followed by posthoc analysis (Dunnett’s multiple comparison tests) and were considered statistically significant if *p* < 0.05 (*p < 0.05, **p < 0.01, ***p < 0.001, ****p < 0.0001).

### In Vitro ADME-Tox Studies

All protocols used for the evaluation of drug-like properties (ADME-To parameters) were described in our previous works.^58,89^ Precoated PAMPA Plate System Gentest was obtained from Corning, (Tewksbury, MA, USA). The metabolic stability assay was performed on human liver microsomes (HLMs, Sigma-Aldrich, St. Louis, MO, USA). The assays with microsomes were supported by MetaSite 6.0.1 software (Molecular Discovery Ltd. Hertfordshire, UK). To predict potential drug–drug interactions (DDIs) the influence on CYP3A4, CYP2D6, and CYP2C9 were carried with use of respective P450-Glo kit (Promega, Madison, WI, USA). The luminescence signal and absorbance were measured by using a microplate reader EnSpire PerkinElmer (Waltham, MA, USA). The LC/MS/MS analyses used in PAMPA and the assays with use of HLMs were obtained on Waters ACQUITY TQD system (Waters, Milford, CT, USA). The reference drugs (caffeine, ketoconazole, quinidine, and sulfaphenazole) were purchased from Sigma-Aldrich (St. Louis, MO, USA). Statistical significances and IC_50_ values were calculated by Graph Pad Prism 9 software.

## Supporting Information Available

X-ray crystallography data of GSK-3β in complex with compound **36**; thermal denaturation curves (first derivative) of GSK-3β after treatment with **36**; quantum mechanical SAR analysis; metabolic stability of compounds **11** and **36** in human liver microsomes—MS analysis and MetaSite predictions; interactions with CYP3A4, CYP2D6, and CYP2C9; kinetics of GSK-3β inhibition by compound **36**; selectivity studies for compound **36**; and ^1^H and ^13^C NMR spectra and LCMS chromatograms (PDF)
