# Supplementary material for: Exploring Novel GSK-3β Inhibitors for Anti-Neuroinflammatory and Neuroprotective Effects: Synthesis, Crystallography, Computational Analysis, and Biological Evaluation
Source: ACS Chem Neurosci. 2024 Aug 19;15(17):3181–201. doi: 10.1021/acschemneuro.4c00365 (PMC11378298; doi:10.1021/acschemneuro.4c00365)
Supplement: Supplementary file 1 — cn4c00365_si_001.pdf [file cn4c00365_si_001.pdf]

Supporting Information

for

Exploring Novel GSK-3β Inhibitors for Anti-Neuroinflammatory and Neuroprotective Effects: Synthesis, Crystallography, Computational Analysis, and Biological Evaluation

Izabella Góral[a,b,‡], Tomasz Wichur[a,‡], Emilia Sługocka[a,b,c], Przemysław Grygier[c,d], Monika Głuch-Lutwin[e], Barbara Mordyl[e], Ewelina Honkisz-Orzechowska[f], Natalia Szałaj[a], Justyna Godyń[a], Dawid Panek[a], Paula Zaręba[a], Anna Sarka[f], Paweł Żmudzki[g], Gniewomir Latacz[f], Katarzyna Pustelny[h], Adam Bucki[g], Anna Czarna[c], Filipe Menezes[i,*], Anna Więckowska[a,*]

[a] Department of Physicochemical Drug Analysis, Faculty of Pharmacy, Jagiellonian University Medical College, 9 Medyczna St., 30-688 Krakow, Poland

[b] Doctoral School of Medical and Health Sciences, Jagiellonian University Medical College, 16 Lazarza St., 31-530 Krakow, Poland

[c] Malopolska Centre of Biotechnology, Jagiellonian University, Gronostajowa 7a, 30-387 Krakow, Poland

[d] Doctoral School of Exact and Natural Sciences, Jagiellonian University, Lojasiewicza 11, 30-348 Krakow, Poland

[e] Department of Pharmacobiology, Faculty of Pharmacy, Jagiellonian University Medical College, 9 Medyczna St., 30-688 Krakow, Poland

[f] Department of Technology and Biotechnology of Drugs, Faculty of Pharmacy, Jagiellonian University Medical College, 9 Medyczna St., 30-688 Krakow, Poland

[g] Department of Medicinal Chemistry, Faculty of Pharmacy, Jagiellonian University Medical College, 9 Medyczna St., 30-688 Krakow, Poland

[h] Department of Physical Biochemistry, Faculty of Biochemistry, Biophysics and Biotechnology, Jagiellonian University, Gronostajowa 7 St., 30-387 Krakow, Poland

[i] Helmholtz Munich, Molecular Targets and Therapeutics Center, Institute of Structural Biology, Ingolstädter Landstr. 1, 85764 Neuherberg, Germany

*Corresponding Authors:

e-mail addresses: filipe.menezes@helmholtz-munich.de (Filipe Menezes), anna.wieckowska@uj.edu.pl (Anna Więckowska)

‡ Izabella Góral and Tomasz Wichur contributed equally to the synthesis. Góral, Wichur and Sługocka contributed equally to the work as a whole.

Contents:

X-RAY CRYSTALLOGRAPHY OF GSK-3B IN COMPLEX WITH COMPOUND 36 4

BIOLOGICAL EVALUATION AND SAR ANALYSIS 6

METABOLIC STABILITY OF COMPOUNDS 11 AND 36 IN HUMAN LIVER MICROSOMES (HLMS) 28

INFLUENCE ON CYP3A4, CYP2D6 AND CYP2C9 ACTIVITY 31

KINETICS OF GSK-3B INHIBITION BY COMPOUND 36 32

SELECTIVITY STUDIES FOR COMPOUND 36 33

LCMS CHROMATOGRAMS AND ^{1}H AND ^{13}C NMR SPECTRA OF FINAL COMPOUNDS 35

REFERENCES: 80

X-ray crystallography of GSK-3β in complex with compound 36

Table S1. Data collection and refinement statistics. Data for the highest resolution shell are shown in parentheses.

	**GSK-3β/36**
**PDB ID**	8QJI
**Wavelength**	0.9677
**Resolution range**	66.20 – 3.02
**Space group**	C 2 2 21
**Unit cell**	86.19 103.38 94.70 (Å)<br>90 90 90 (°)
**Total reflections**	47466
**Unique reflections**	8530 (1385)
**Multiplicity**	5.6 (5.8)
**Completeness (%)**	98.9 (66.20-3.02)
**Mean I/sigma(I)**	8.2 (1.4)
**Wilson B-factor**	86.8
**R-merge**	0.137 (1.317)
**R-meas**	0.151 (1.447)
**CC1/2**	0.997
**Reflections used in refinement**	55222 (5322)
**Reflections used for R-free**	868 (10.14%)
**R-work**	0.250
**R-free**	0.288
**Overall number of atoms**	2457
**In macromolecules**	2412
**In ligands**	22
**In waters**	23
**Protein residues**	340
**RMS (bonds)**	0.002
**RMS(angles)**	0.483
**Ramachandran favored (%)**	300 (90%)

**Ramachandran allowed (%)**	31 (9%)
**Ramachandran outliers (%)**	2 (1%)
**Rotamer outliers (%)**	5 (2%)
**Clashscore**	5
**Average B-factor (all atoms)**	101 Å^2

Biological evaluation and SAR analysis

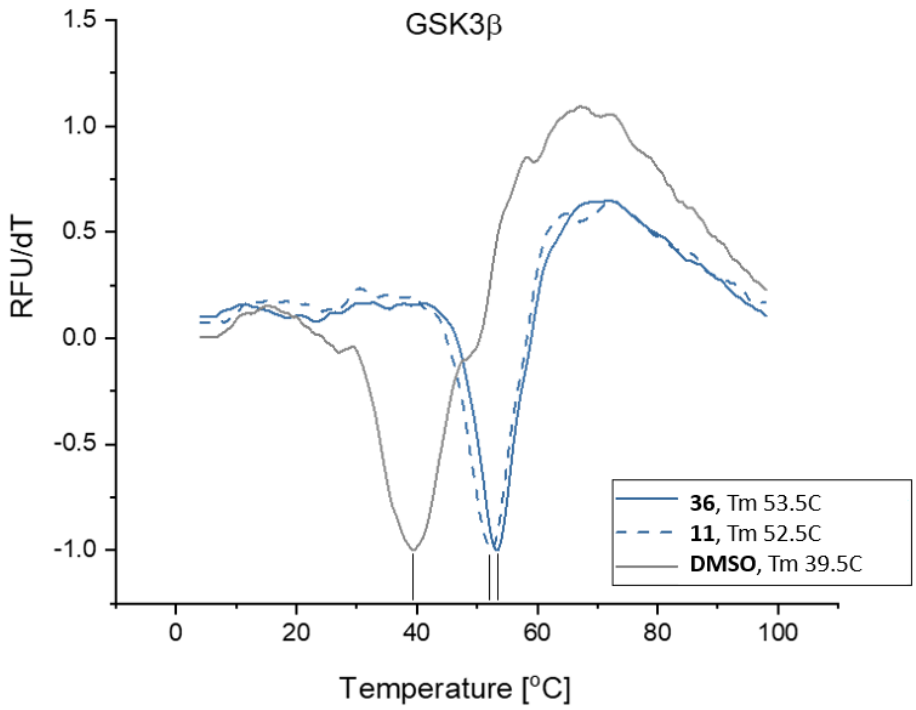

Figure S1. Thermal denaturation curves (first derivative) of GSK-3β after treatment with **36**, **11** or DMSO as determined in Sypro Orange dye monitored thermal shift assay.

Quantum Mechanical SAR

SC1. Energy Decomposition and Deconvolution Analysis.

The main interactions probed by the EDDA calculations are:

1) electrostatic interactions (ES), resulting from the interplay of protein and ligand as a collection of point-charges,
2) polarization (POL) resulting from the deformations of the electronic densities of each molecule as a response to the presence of its binding partner,
3) charge transfer (CT) accounting for the unidirectional intermolecular flow of electronic density, or exchange, if the flow is occurring synchronously; note that the method used for the calculations does not allow the separation of polarization from an exchange,
4) dispersion (DISP) - inter-particle repulsion generating a correlation in the electronic motions, which yields a stabilizing effect due to instantaneous asymmetries of the atomic densities). Note that this has a 1:1 correlation with lipophilicity,
5) repulsive effect (REP), which corresponds to steric repulsion caused by the overlap of electronic densities,
6) solvation contribution (SOLV) - accounting for the role of solvent for binding,
7) interaction energy (INT), the sum of all contributions, which correlates with the enthalpy of binding.

To facilitate analysis and explore the locality of interactions, our EDDA algorithm further partitions each contribution in a pairwise fashion. This allows us to build interaction maps (imaps), which offer an immediate overview of the landscape of interactions.

SC2. Amide Vs. Urea: Analysing the SAR data of compounds **10** and **11**. **Figure S2** summarizes the key findings regarding the comparison of compounds **10** (terminal amide) and **11** (terminal urea). The calculations indicate that the binding of compound **11** to GSK-3β is favored by approximately 1 kcal/mol (**Table S2**), which reflects the experimental relative IC_{50} (calc. 5.0; exp. 4.2, see differential energy decomposition graph in **Figure S2A**). This preference results from a fine balance of several factors. The total interaction maps for the two compounds show anchoring points between the ligand's aminopyridine and the main chain of the hinge region residue Val135 (**Figures S2D**, **E**). The major component contributing to distinguishing inhibitors relates indeed to the amide/urea termini, which adopt different binding motifs around the catalytic Lys85 (**Figures S2B**, **C**). This brings an additional stabilization in the case of compound **11**, as it facilitates a weak H-bond between the urea and Glu97, which further strengthens the H-bond of this group with Lys85. This is reflected in the interaction maps (**Figures S2D**, **E**). In addition to this effect, the slight change in binding pose brings an asymmetry in the H-bond network between the aminopyridine and Val135, which is absent for compound **10**. This is also reflected in the weights of the partitioned energies recorded by the calculations.

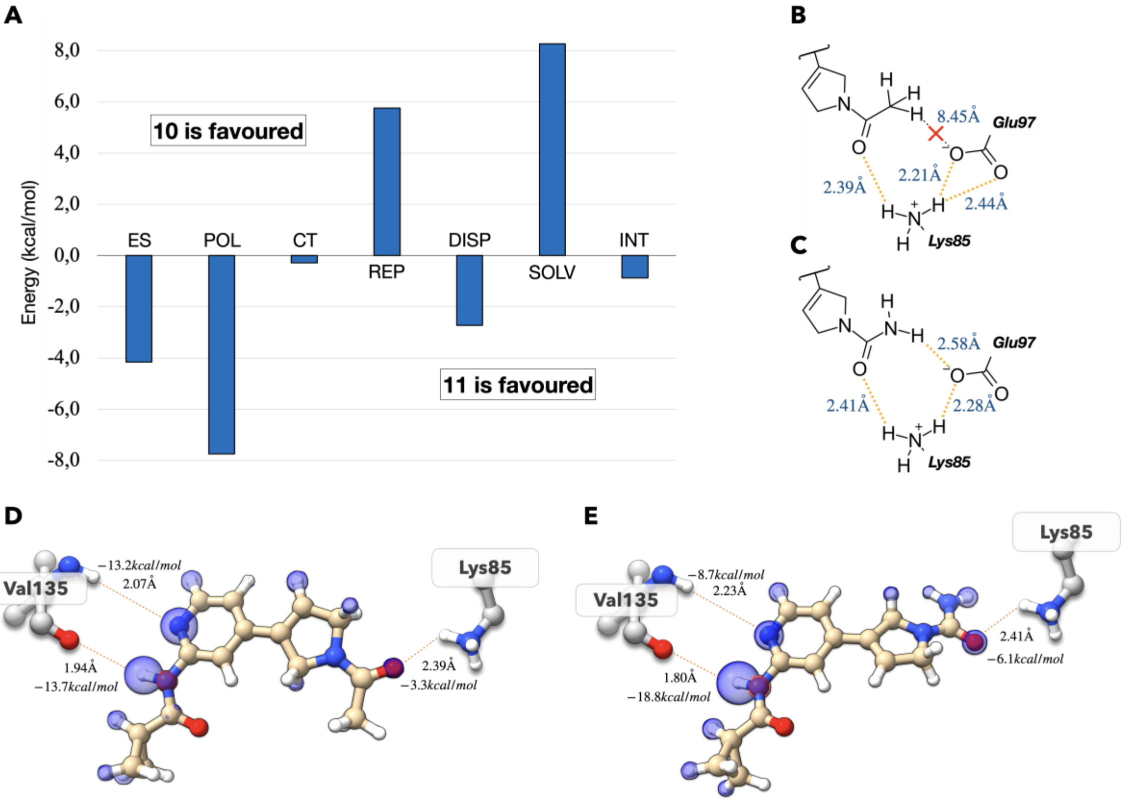

Figure S2. (**A**) Differential EDDA results for the comparison between compounds **11** and **10**. Abbreviations stand for ES, electrostatics; POL, polarization; CT, charge transfer; REP, repulsion; DISP, dispersion; SOLV, solvation; INT, total interaction energy. For a more

detailed description refer to section SC2. (**B**), (**C**) Schematic representation of interactions captured by each compound around the catalytic lysine. Atom-specific contributions to the total interaction energy and some of the key distances for compounds **10** (**D**) and **11** (**E**).

The repulsion energy, which corresponds to the overlap of electronic densities between protein and ligand, is larger in compound **11** by almost 6 kcal/mol (**Table S2**). The respective maps (see **Figures S3** and **S4**) reveal the asymmetry mentioned above in the binding motif around the hinge for the two compounds, resulting from a different accommodation of the urea in the pocket. More than just affecting how the ligand interacts with the hinge and the catalytic lysin, the change in binding pose brings a strong contact with the hydrogens of the 2,5-dihydro-1*H*-pyrrole group. Repulsion is largely compensated by the exchange-polarization terms. Note that this is meaningful since electronic exchange contributions are most predominant when the atoms are closer. Similarly, electrostatics and solvation contributions cancel to a great degree. Dispersion forces, or lipophilicity, further contribute to favor the relative binding energy towards the urea. This is brought by the sp^2 hybridization of the terminal nitrogen, which is active in the π system of this functional group (see **Figure S5**).

Altogether, the key elements making compound **11** more active than **10** are i) electrostatics and the ability to efficiently capture hydrogen bonds from the hinge and Lys85; ii) better fit to the pocket; iii) extended lipophilic contacts due to a terminal π system.

Table S2. Summary of different Energy Decomposition and Deconvolution Analysis contributions for analyzed pair of complexes of GSK-3β with compounds **11**, **10**; all values given in kcal/mol.

	**11**	**10**
E_{INT}	**-29.18**	**-28.31**
E_{ES}	**-32.18**	**-28.03**
E_{POL}	**-53.37**	**-45.63**
E_{CT}	**0.13**	**0.41**
E_{DISP}	**-32.74**	**-30.01**

E_{SOLV}	**28.98**	**20.70**
E_{REP}	**60.01**	**54.25**

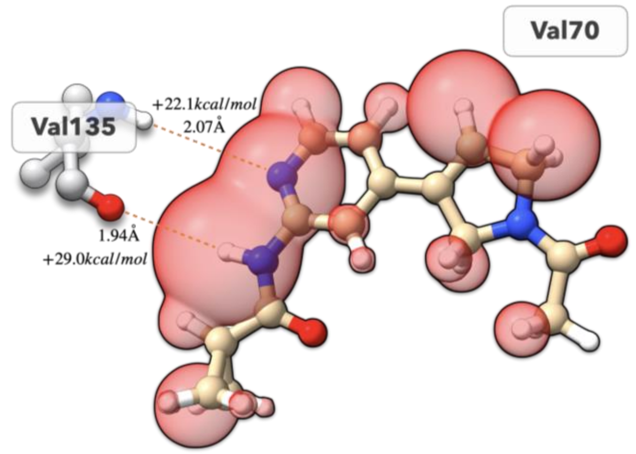

Figure S3. Repulsion maps for compound **10**.

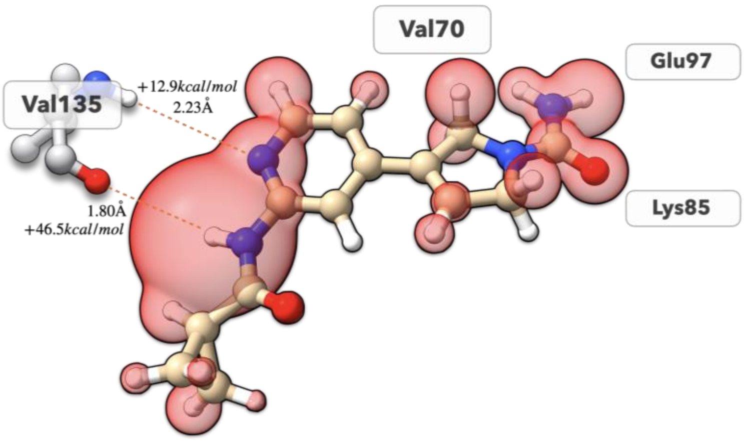

Figure S4. Repulsion maps for compound **11**.

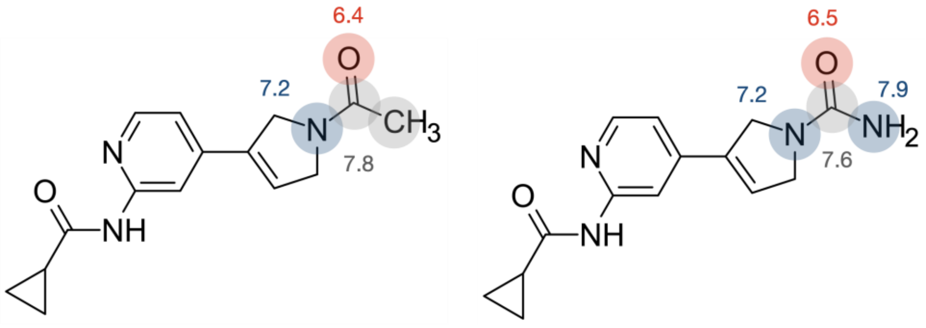

Figure S5. Atomic polarizabilities in a_0^3 for selected atoms of the amide and urea ligands **10** and **11**.

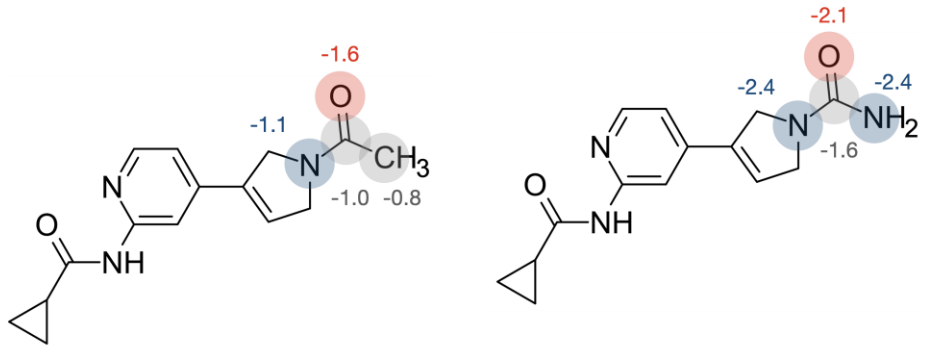

Figure S6. Selected contributions to the dispersion interaction energy for atoms of the amide and urea ligands **10** and **11**. Values in kcal/mol.

SC3. The QM-SAR data of compounds **10** and **12**. Further, we have analyzed the effect of the introduction of alkylamino substituents on the GSK-3β inhibitory activity, as in compounds **12–15**. Preliminary docking studies indicated that the introduction of such substituents should enhance the activity through additional interactions with Asn186 or Asp200. **Figure S7** summarizes the main findings, comparing compounds **10** and **12**.

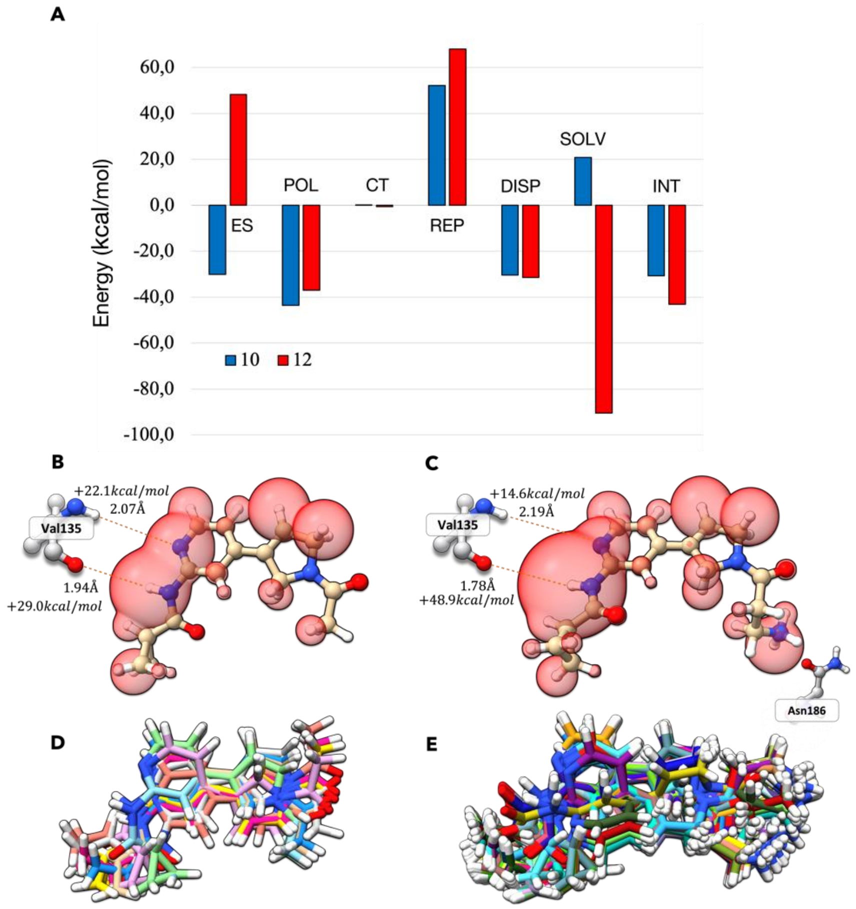

Figure S7. (**A**) EDDA results comparing compounds **10** and **12**. For the description of abbreviations refer to **Figure S2**. Repulsion maps, key contributions to the total interaction energy, and some of the key distances for compounds (**B**) **10** and (**C**) **12**, respectively. Representation of the main conformers for compounds (**D**) **10** and (**E**) **12** used for estimating the conformational entropy values.

Expectedly, compound **12** shows an interaction energy that is significantly lower than that of compound **10** (**Figure S7A**). This results from the ethylaminium extension in compound **12**, granting the interaction with Asn186. The fact that compound **12** has a larger interaction surface with the protein than compound **10** generates an imbalance in the binding energies, hampering any direct comparison between these quantities without consideration of the respective entropy penalties, which are completely absent from binding energies [1]. Consequently, the EDDA results can only be analyzed to retrieve positive cues leading to the binding of the two compounds (see section **Table S3**).

EDDA calculations show that the H-bond between the ethylaminium tail and Asn186 is a critical element for binding compound **12**. For this to take place, the ethylene linkage of the tail is forced to interact with the ATP binding pocket, which may be followed in the repulsion maps (**Figures S7B**, **C**). That contact increases the interaction surface between molecules with no pronounced gain in enthalpy, meaning that additional entropy penalties are expected. Fitting the ethylaminium tail in the pocket also requires a tighter binding mode of the ligand to the pocket. A natural consequence is that the protein must significantly deform the ligand for binding to take place, introducing a penalty for bringing the ligand to its binding pose. A comparison of the free and bound modes of compounds **10** and **12** reveals that the latter pays an additional 5.7 kcal/mol in deformation energy. Conformational entropy calculations show an increase of 33% for compound **12**, which must furthermore be lost upon binding (5.1 $cal.K^{-1}.mol^{-1}$ for compound **10**; 7.2 $cal.K^{-1}.mol^{-1}$ for compound **12**; representation of ensembles of the conformers on **Figures S7D**, **E**). These entropic penalties shift the binding energies by an additional 0.6 kcal/mol, contributing towards the preferencial binding of compound **10**. In summary, though the EDDA calculations could not reflect the relative orders of activity for the two compounds, we could nonetheless use it to extract information to infer the reasons behind the loss of binding. These are i) additional deformation energy penalties which are requested from the ligand; ii) unfavorable entropy penalties. Together, these factors make **12** a poor candidate for further development.

Further details on the comparison between compounds **10** and **12**. The repulsion maps for the two ligands in the pocket (see **Figures S7B**, **C**) show a sharp increase in the overlap of electronic densities in the hinge region. This affects primarily the amide group, for which repulsion increases by 20 kcal/mol. Much like the case of ligands **10** and **11**, there is a slight decrease in repulsion for nitrogen 2 (for atom naming convention refer to **Figure S8**) due to small differences in the binding pose, which furthermore impacts the 2,5-dihydro-1*H*-pyrrole moiety contact with Val70. This allows us to build the hypothesis where **12**, in order to fit the pocket and to accommodate the ethyl ammonium tail, brings all the other groups too close to the pocket of the kinase. This means that also the aminopyridine group comes closer to the hinge on the carboxamide moiety, the 2,5-dihydro-1H-pyrrole moiety is closer to Val70, and the cyclopropyl terminus comes closer to Pro136. All of these may be visually followed on the maps presented in **Figures S7B**, **C**. Consequently, to accommodate the extended tail in the binding pocket, **12** is forced to remain tighter in the kinase pocket. The question, however, is, since the ethyl ammonium tail does not capture a hydrogen bond with Asp200, what is the reason behind the attempt to accommodate the whole ligand in the pocket? The repulsion (see **Figure S7C**) and the total interaction map (see below, **Figure S10**) bring the answer to that question indicating that the terminal ammonium participates in a hydrogen bond with Asn186.

Table S3. Summary of different Energy Decomposition and Deconvolution Analysis contributions for analyzed pair of complexes of GSK-3β with compounds **12**, **10**; all values given in kcal/mol.

	**12**	**10**
E_{INT}	**-43.04**	**-28.31**
E_{ES}	**48.35**	**-28.03**
E_{POL}	**-36.97**	**-45.63**
E_{CT}	**-0.60**	**0.41**
E_{DISP}	**-31.42**	**-30.01**
E_{SOLV}	**-90.45**	**20.70**
E_{REP}	**68.04**	**54.25**

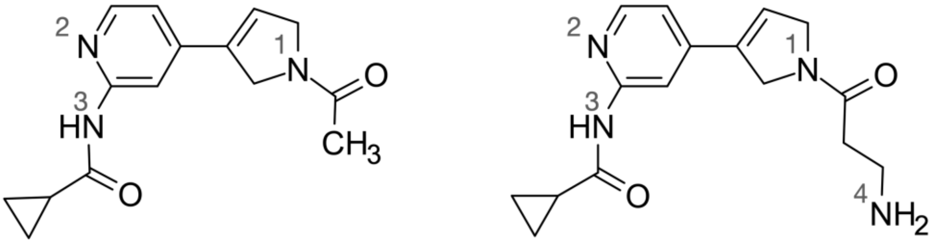

Figure S8. Atom naming convention for ligands **10** (left) and **12** (right) used in the discussion above.

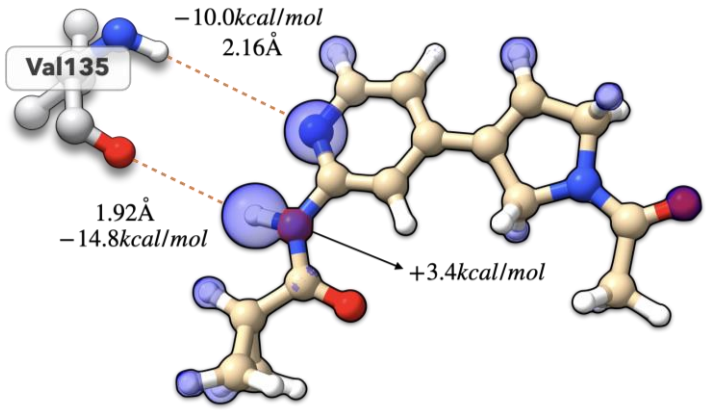

Figure S9. Total interaction maps for compound **10**.

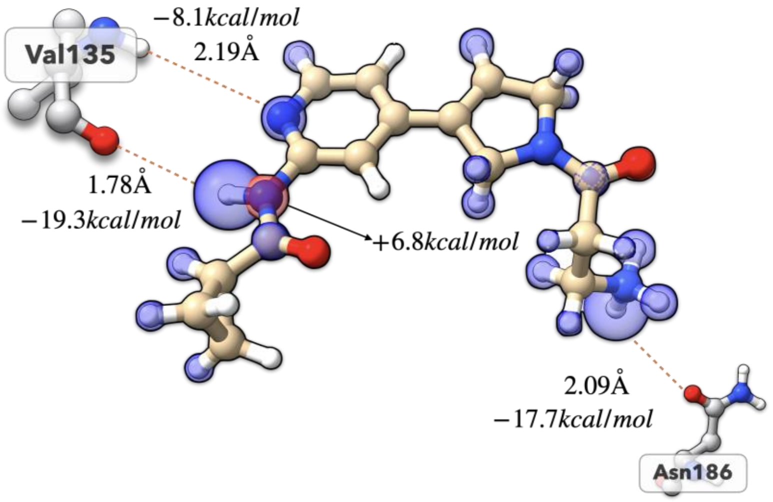

Figure S10. Total interaction maps for compound **12**.

SC4. Comparing amides and sulphonamides, the case of compounds **10** and **16**.

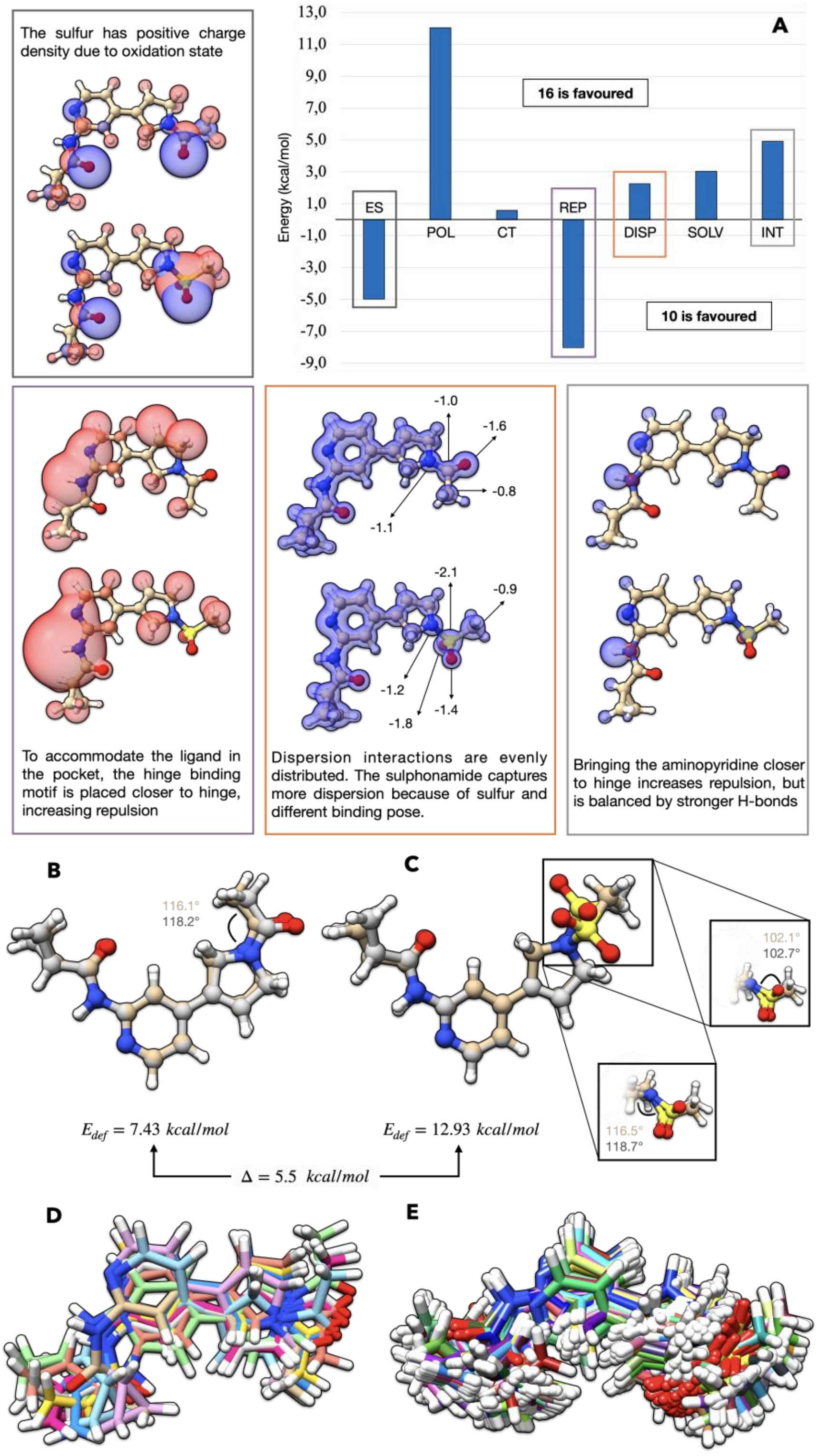

Figure S11. (**A**) Differential EDDA results for the comparison between compounds **10** and **16** with comments addressing selected energy contributions. For the description of abbreviations refer to **Figure S2**. Deformation energy values for (**B**) **10** and (**C**) **16** together with an aligned representation of their binding pose (silver) and free ligand (beige) geometries, with the emphasis on altered angle measures of the sulfonamide derivative. Representation of the main conformers for compounds (**D**) **10** and (**E**) **16** used for estimating the conformational entropy values.

Sulfonamides were reported in a number of projects as amides' bioisosteres [2]. Replacing the terminal amide group of **10** with the sulfonamide of **16** was therefore a natural substitution to undertake. The experimental data shows, however, that this substitution leads to a significant decrease in activity, which is observed for all sulfonamide-substituted compounds (compounds **16**, **17**, **18**, **19**, **20**). **Figure S11** compares partitioned energies for compounds **10** and **16**. Replacing the amide with a sulfonamide lowered the binding energy by 5 kcal/mol (**Table S4**), again, in contrast to experimental data. A closer look at the structures revealed that the angles of the sulfonamide group in the bound ligand were flattened when compared to the freely optimized molecule (**Figures S11B**, **C**). Note that like other *N*, *N*-dialkyl sulfonamides (see for instance the CSD entry AFAJOJ [3,4]), our simulations indicate a strong sp^3 character for compound **16**'s sulfonamide's nitrogen (see **Figure S12**). Consequently, the sulfonamide warps the 2,5-dihydro-1*H*-pyrrole conformation and distorts the aminopyridine hinge binding motif, sacrificing the planarity of the latter to maintain the H-bond with Val135 which was not observed for compound **10** (see **Figure S13**). This hinted at the potential role of deformation energy in decreasing the inhibitory potency, which was once more confirmed by calculations (difference of 5.5 kcal/mol). However, the lack of planarity of sulfonamides like the ones we synthesized brings additional conformational entropy penalties. Our calculations indicate that the sulfonamide has almost twice as many conformers as the amide, leading to conformational entropies of 5.1 $cal.K^{-1}.mol^{-1}$ for **10** and 8.4 $cal.K^{-1}.mol^{-1}$ for **16** (**Figures S11D**, **E**). When combined, deformation energy and loss of conformational freedom shift the balance towards the amide, leading to a difference in binding energy of approximately 1.5 kcal/mol. The primary conclusion drawn from our calculations is that, while sulfonamide **16** exhibits a greater potential for tightly binding with GSK-3β (as indicated by its lower binding energy), the binding process to the protein and the necessary conformational changes incur substantial energy penalties due to the loss of conformational freedom and the structural rearrangement.

Table S4. Summary of different Energy Decomposition and Deconvolution Analysis contributions for analyzed pair of complexes of GSK-3β with compounds **10**, **16**; all values given in kcal/mol.

	**16**	**10**
E_{INT}	**-33.22**	**-28.31**
E_{ES}	**-23.04**	**-28.03**
E_{POL}	**-57.67**	**-45.63**
E_{CT}	**-0.18**	**0.41**
E_{DISP}	**-32.37**	**-30.01**
E_{SOLV}	**17.66**	**20.70**
E_{REP}	**62.28**	**54.25**

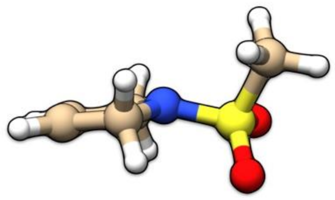

Figure S12. CAM-B3LYP/6-31G** optimized structure of the 1-methanesulfonyl-2,5-dihydro-1H-pyrrole moiety of compound **16** in CPCM water. Figure focusing on visualizing the bent shape of the sulphonamide with the sp^3 character of the nitrogen atom.

Figure S12 shows the CAM-B3LYP/6-31G** optimized geometry for the moiety of compound **16** containing the sulfonamide group. We chose CAM-B3LYP to optimize the molecule because GFN2-XTB was not parametrized using data with the functional, while also leading to good geometries. Calculations were performed using ORCA 5.0.4. [5–7].

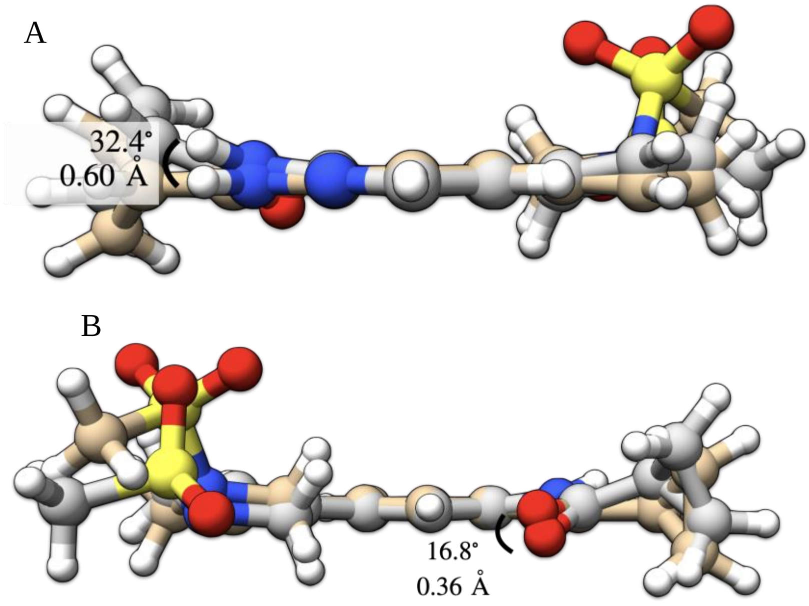

Figure S13. Horizontal representations of the binding pose geometry (silver) and the GFN2-xTB optimized geometry (beige) of compound **16**. Alignments with emphasis on the angle measures and distances between amide nitrogen atoms (**A**, upper) and carbonyl oxygen (**B**, lower) of the hinge binding motif in both representations of the molecule.

SC5. Additional maps comparing the binding modes of compound **36**.

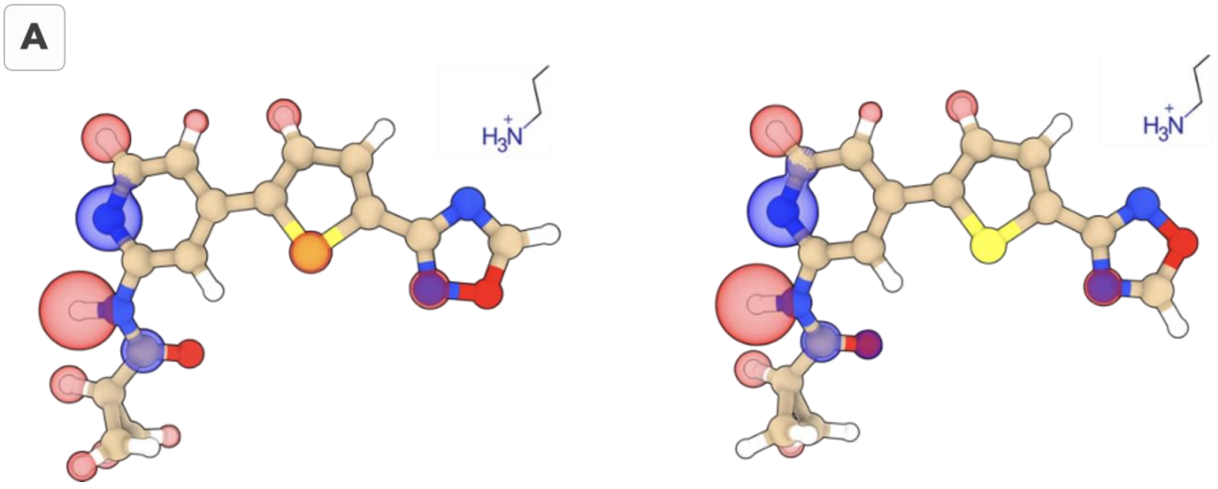

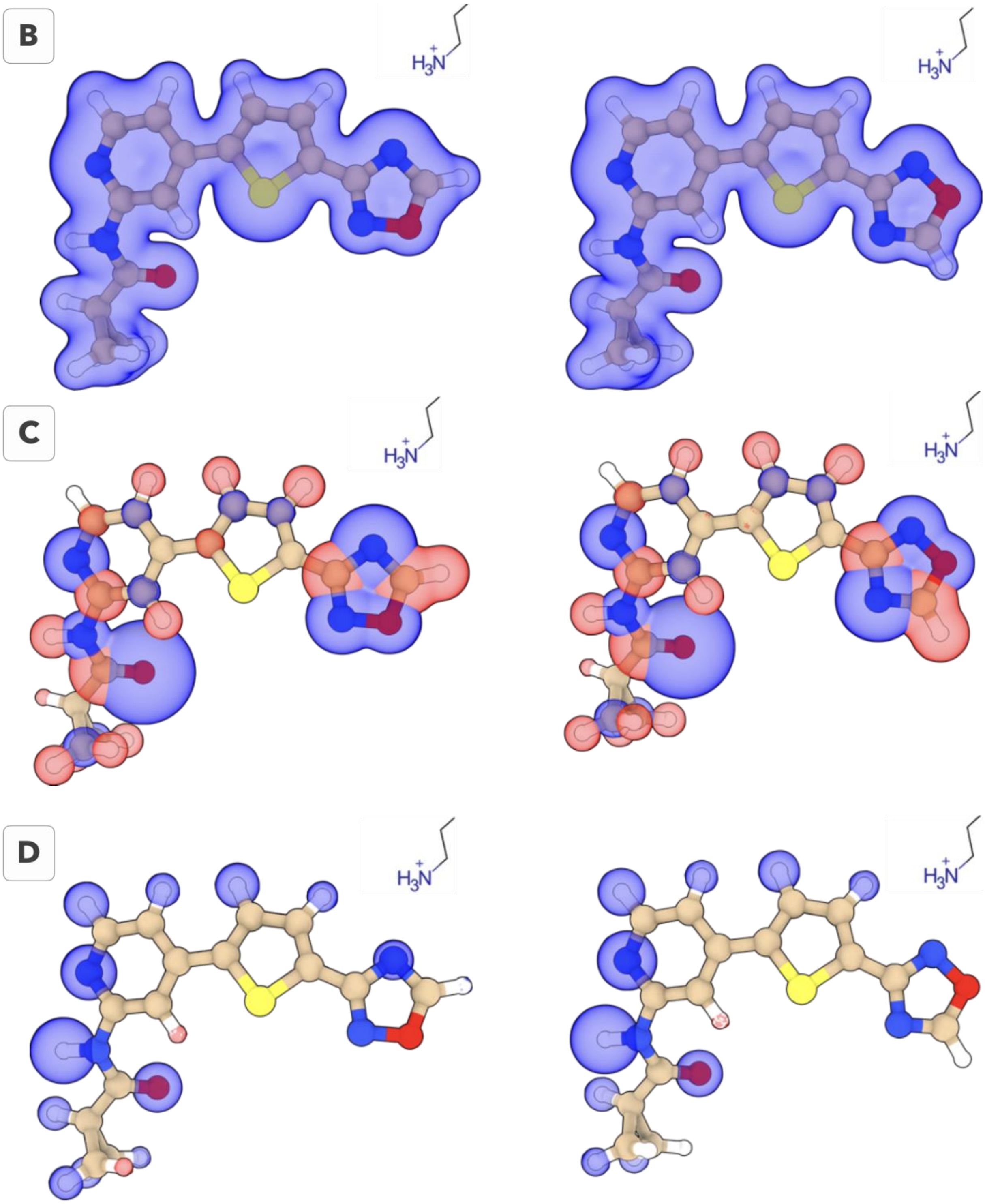
B
C
D
H3N+

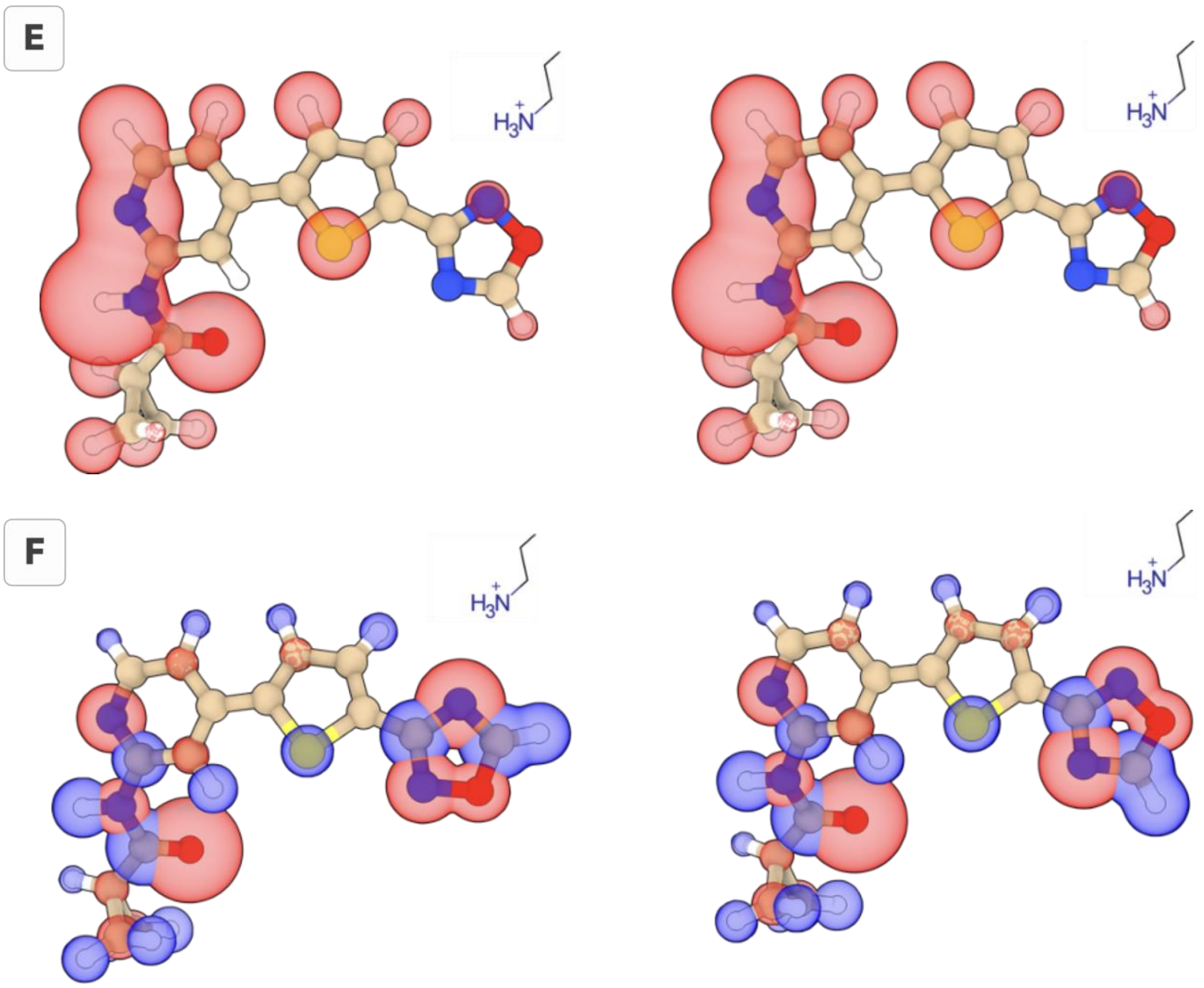

Figure S14. (**A**) The CT maps. (**B**) The DISP maps. (**C**) The ES maps. (**D**) The POL maps. (**E**) The REP maps. (**F**) SOLV maps.

Table S5. Summary of EDDA contributions for the N-C and N-O binding modes of compound **36**; all values given in kcal/mol.

	**36 N-C**	**36 N-O**
E_{INT}	**-32.16**	**-32.68**
E_{ES}	**-25.88**	**-23.81**
E_{POL}	**-59.39**	**-53.44**
E_{CT}	**0.12**	**0.32**
E_{DISP}	**-35.92**	**-35.18**
E_{SOLV}	**19.68**	**15.00**
E_{REP}	**69.24**	**64.43**

SC6. EDDA analysis of the binding modes of compound **34**.

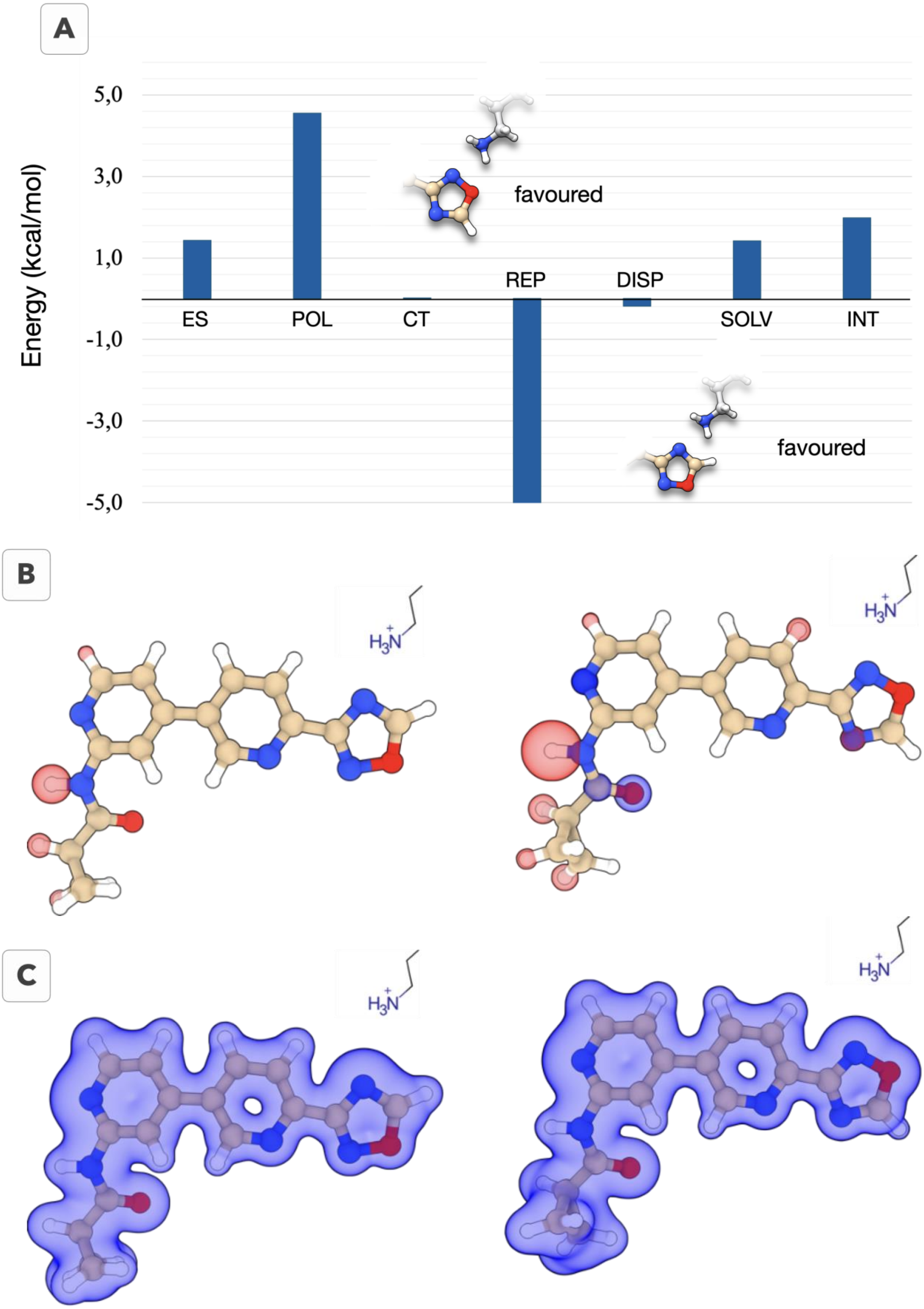

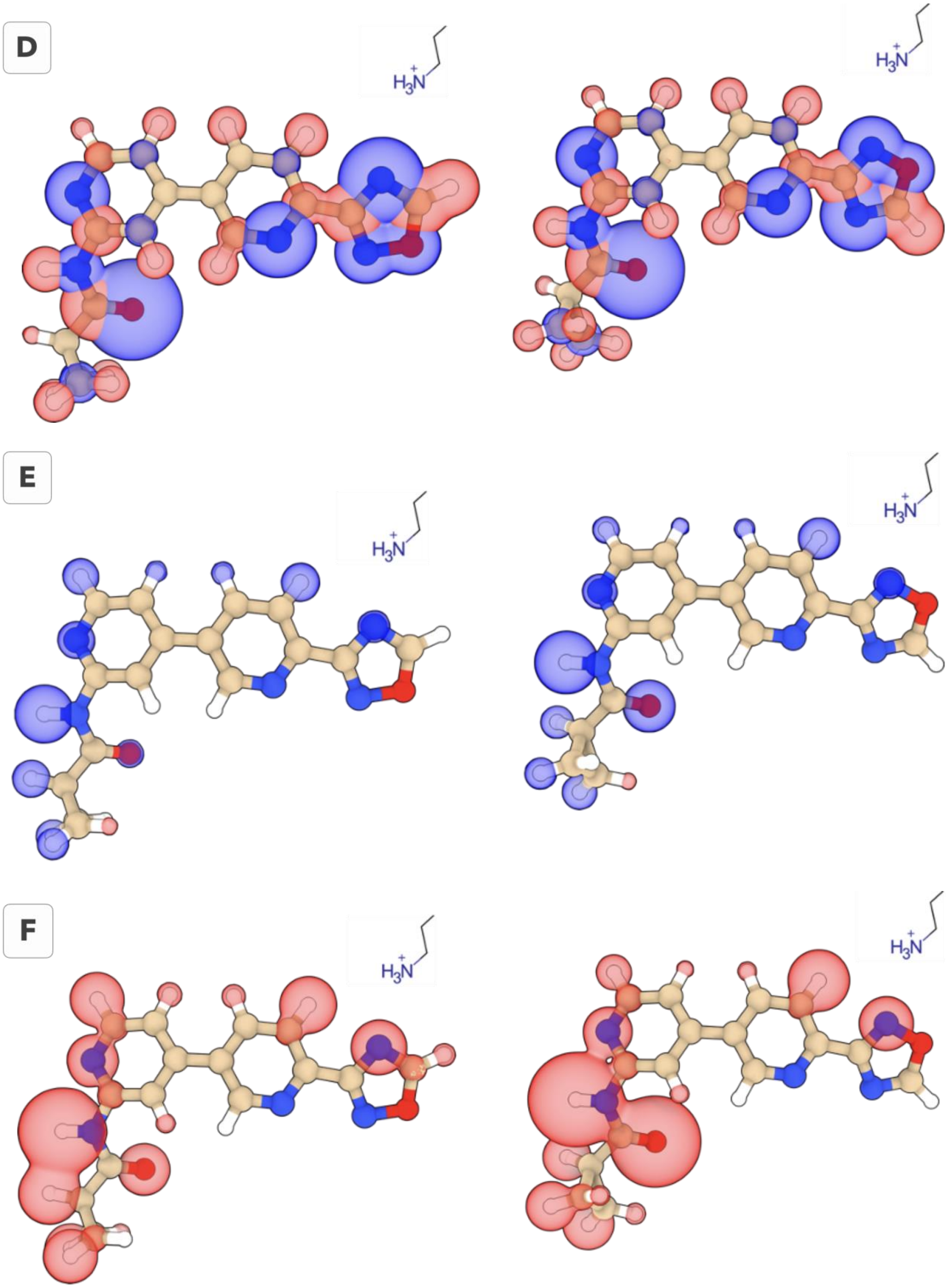
D
H_3N^+
H_3N^+
E
H_3N^+
H_3N^+
F
H_3N^+
H_3N^+

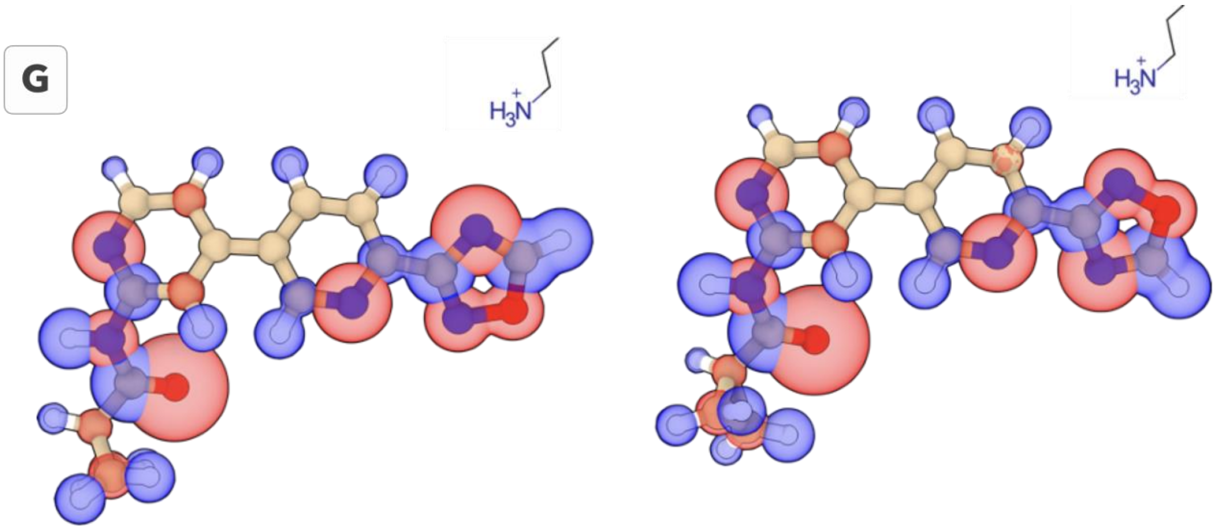

Figure S15. (**A**) Distribution of relative energies for the two binding modes of compound **34**. (**B**) The CT maps. (**C**) The DISP maps. (**D**) The ES maps. (**E**) The POL maps. (**F**) The REP maps. (**G**) SOLV maps.

Table S6. Summary of EDDA contributions for the N-C and N-O binding modes of compound **34**; all values given in kcal/mol.

	**34 N-C**	**34 N-O**
E_{INT}	**-27.63**	**-29.61**
E_{ES}	**-23.30**	**-24.73**
E_{POL}	**-42.94**	**-47.49**
E_{CT}	**0.08**	**0.07**
E_{DISP}	**-34.21**	**-34.03**
E_{SOLV}	**22.15**	**20.73**
E_{REP}	**50.58**	**55.83**

SC7. EDDA analysis of the N-O binding mode of compounds **36** and **34**.

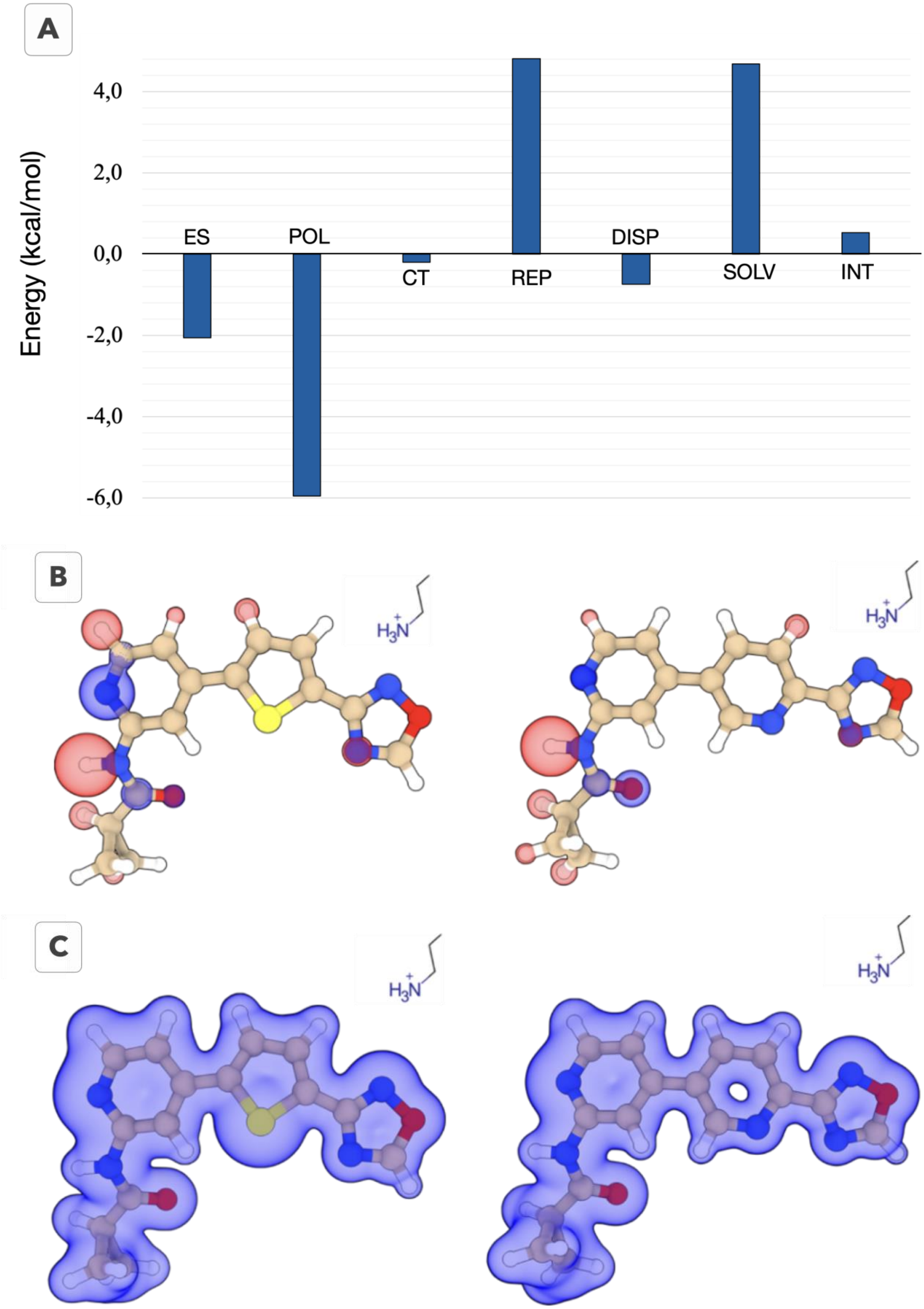

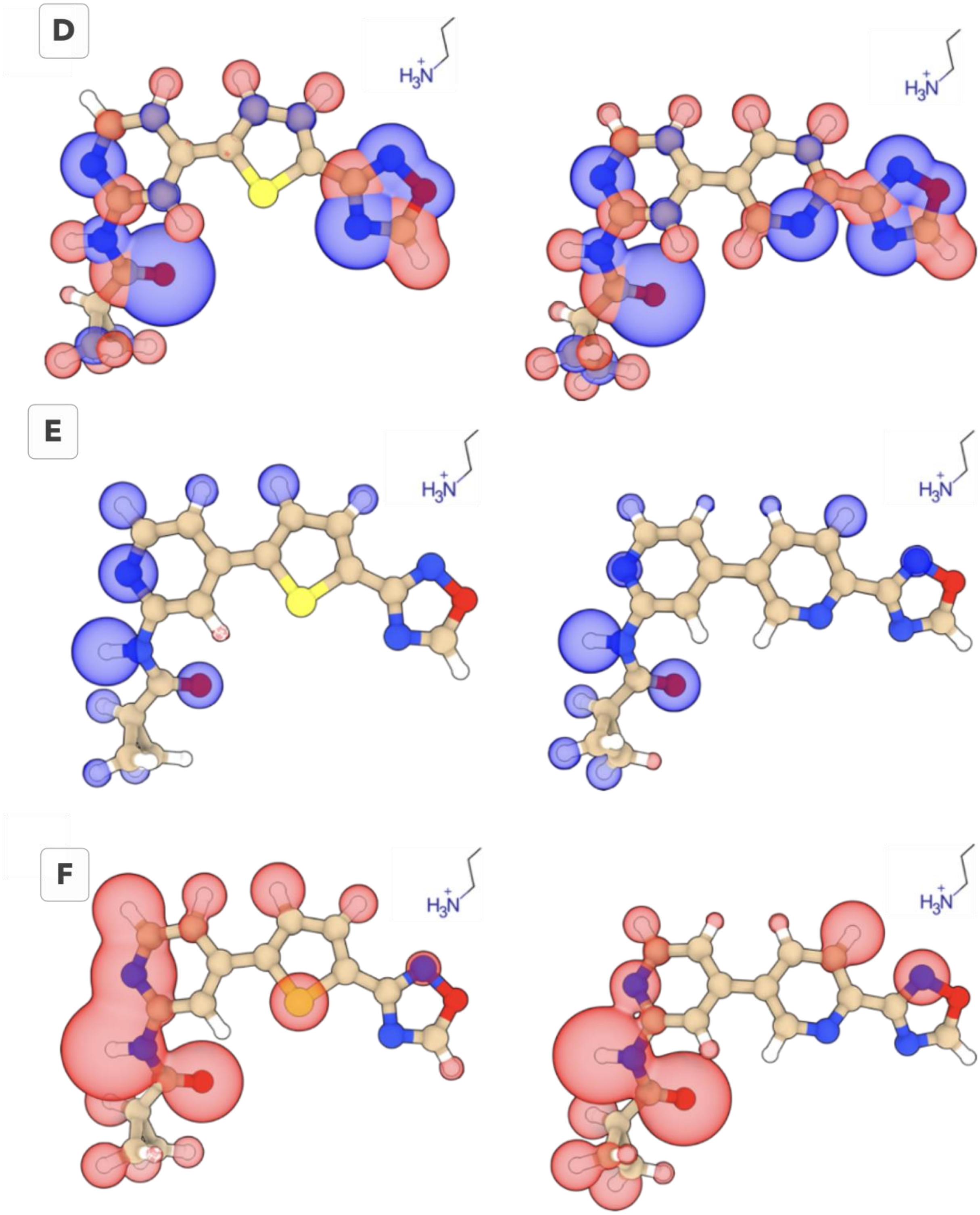
D
E
F

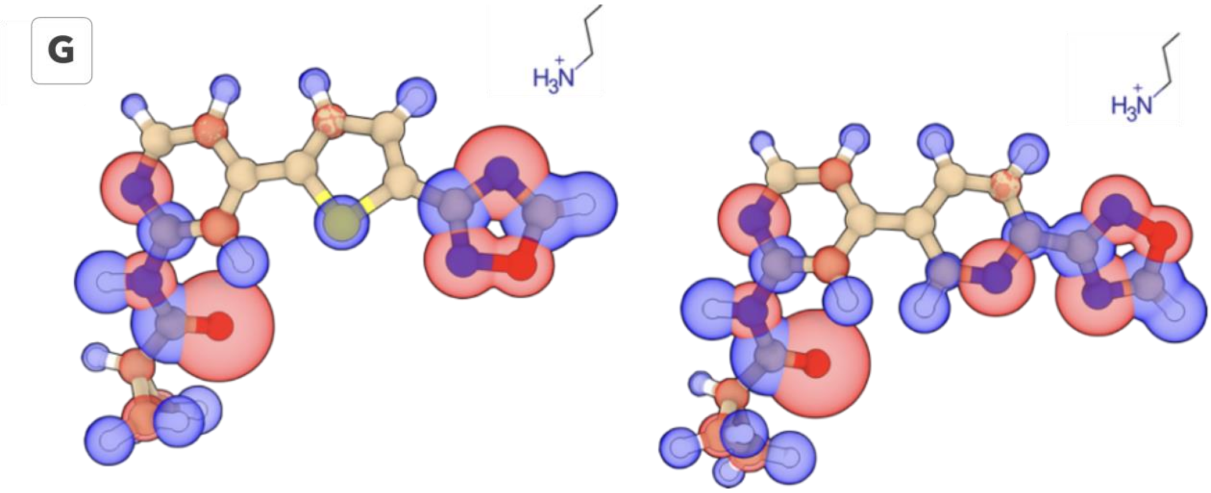

Figure S16. (**A**) Differential distribution of relative energies for the NO- binding mode of compounds **36** (left) and **34** (right). (**B**) The CT maps. (**C**) The DISP maps. (**D**) The ES maps. (**E**) The POL maps. (**F**) The REP maps. (**G**) SOLV maps.

Table S7. Summary of EDDA contributions for the N-O binding modes of compounds **36** and **34**; all values given in kcal/mol.

	36 N-O	34 N-O
E_{INT}	**-32.68**	**-29.61**
E_{ES}	**-23.81**	**-24.73**
E_{POL}	**-53.44**	**-47.49**
E_{CT}	**0.32**	**0.07**
E_{DISP}	**-35.18**	**-34.03**
E_{SOLV}	**15.00**	**20.73**
E_{REP}	**64.43**	**55.83**

SC8. *Ab initio* and DFT calculations to further evaluate the dual binding mode of the oxadiazole ring.

Table S8. Energy values in Hartree (E_h) for L (ligand), P (protein residues), PL (protein-ligand complex) calculated with different methods in water (H_2O) or gas environments. Binding energies (ΔE) given in kcal/mol.

Method	env	mode	L (E_h)	P (E_h)	PL (E_h)	ΔE (E_h)	ΔE (kcal/mol)
GFN2-xTB	gas	NO	-27,984811	-198,4009738	-226,4077345	-0,021950	-13,77
		NC	-27,984104	-198,4009738	-226,4142465	-0,029168	-18,30
	H_2O	NO	-27,990878	-198,5739093	-226,5822969	-0,017509	-10,99
		NC	-27,990293	-198,5739093	-226,5842787	-0,020076	-12,60
r^2SCAN-3C	gas	NO	-813,828215	-3170,525241	-3984,371632	-0,018176	-11,41
		NC	-813,827460	-3170,525241	-3984,379362	-0,026661	-16,73
	H_2O	NO	-813,838611	-3170,696255	-3984,549526	-0,014660	-9,20
		NC	-813,837847	-3170,696255	-3984,548614	-0,014512	-9,11
PBE-D3	gas	NO	-813,453763	-3168,648818	-3982,125642	-0,023062	-14,47
		NC	-813,453036	-3168,648818	-3982,132989	-0,031136	-19,54
	H_2O	NO	-813,46355	-3168,814977	-3982,297518	-0,018990	-11,92
		NC	-813,4628	-3168,814977	-3982,296959	-0,019186	-12,04
B3LYP-D3	gas	NO	-813,82752	-3170,718743	-3984,569138	-0,022878	-14,36
		NC	-813,82665	-3170,718743	-3984,578001	-0,032605	-20,46
	H_2O	NO	-813,83796	-3170,891027	-3984,748646	-0,019663	-12,34
		NC	-813,83708	-3170,891027	-3984,748572	-0,020465	-12,84
wB97X	gas	NO	-813,95794	-3171,497901	-3985,469576	-0,013736	-8,62
		NC	-813,95682	-3171,497901	-3985,478058	-0,023339	-14,65
	H_2O	NO	-813,96913	-3171,674591	-3985,654412	-0,010686	-6,71
		NC	-813,96801	-3171,674591	-3985,653393	-0,010789	-6,77
DLPNO-CCSD(T)	gas	NO	-812,74281	-3165,781599	-3978,545071	-0,020662	-12,97
		NC	-812,74232	-3165,781599	-3978,553567	-0,029650	-18,61
	H_2O	NO	-812,75291	-3165,953368	-3978,723719	-0,017442	-10,94
		NC	-812,7523	-3165,953368	-3978,723173	-0,017505	-10,98

Table S9. Differences in binding energies given for methods in gas and water environment.

Method	gas [kcal/mol]	water [kcal/mol]
GFN2-xTB	-4,53	-1,61
r^2SCAN-3C	-5,32	0,09
PBE-D3	-5,07	-0,12
B3LYP-D3	-6,10	-0,50
wB97X	-6,03	-0,06
DLPNO-CSSD(T)	-5,64	-0,04

<u>Metabolic stability of compounds 11 and 36 in human liver microsomes (HLMs)</u>

Table S10. The molecular masses and metabolic pathways of compounds **11** and **36** determined using human liver microsomes.

**Substrate**	**Molecular mass (*m/z*)**	**% remaining**	**Molecular mass of the metabolite (*m/z*)**	**Metabolic pathway***
**11**	407.26	100	–	*no metabolites found*
**36**	413.24	88.61	329.10 (M1)	*hydroxylation or N-oxidation*
			245.08 (M2)	*amide hydrolysis*

*most probable metabolic pathways were estimated by MS analyses supported by *in silico* prediction using MetaSite 6.0.1 and ADMET Predictor tools

11

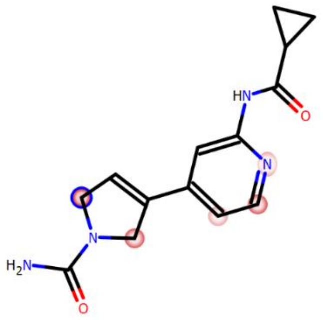

36

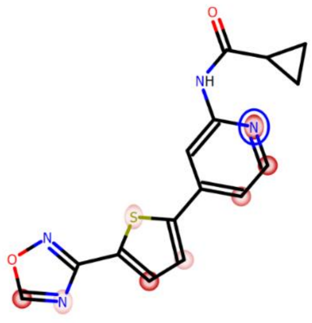

Figure S17. The MetaSite 6.0.1. software prediction of the most probable sites of compounds' **11** and **36** metabolism. The darker the red color - the higher the probability of being involved

in the metabolism pathway. The blue circle marked the site of the compound with the highest probability of metabolic bioconversion.

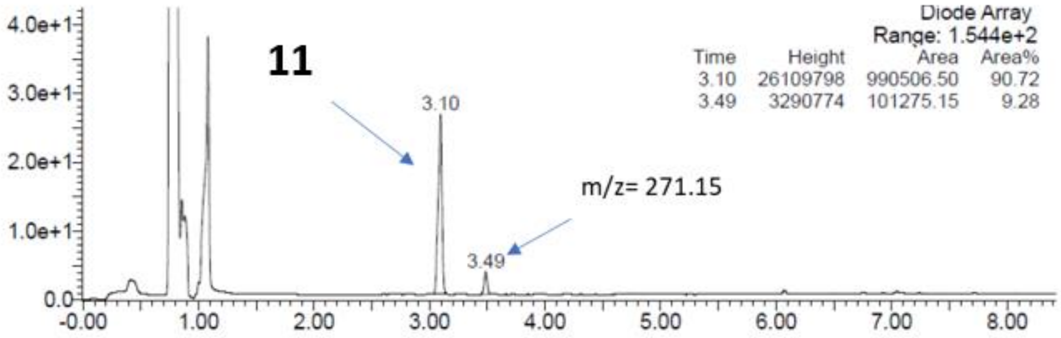

Figure S18. UPLC spectra after 120 min incubation of compound **11** with human liver microsomes in TRIS buffer pH=7.4 at 37 ºC. No metabolites were found. The contamination (m/z=271.15) was observed (also in the control reaction without microsomes – see Figure **S19**).

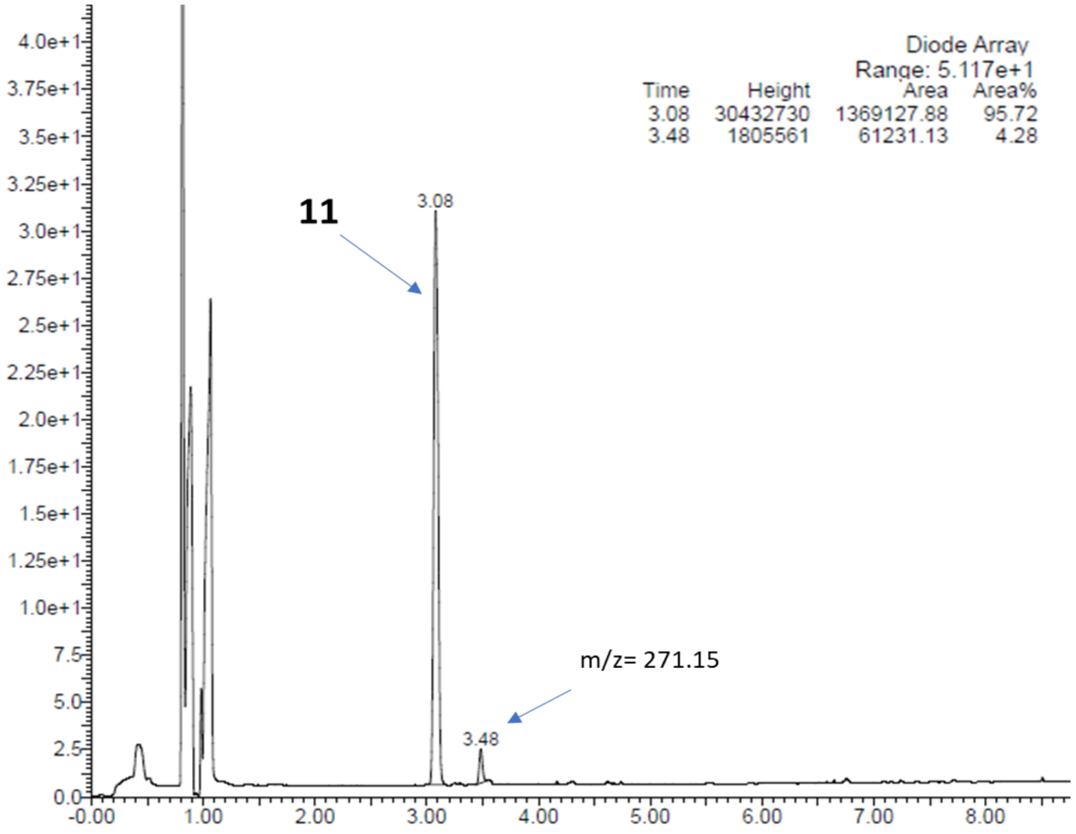

Figure S19. UPLC spectra after 120 min incubation of compound **11** in TRIS buffer pH=7.4 at 37 ºC (without microsomes). The contamination (m/z=271.15) was observed.

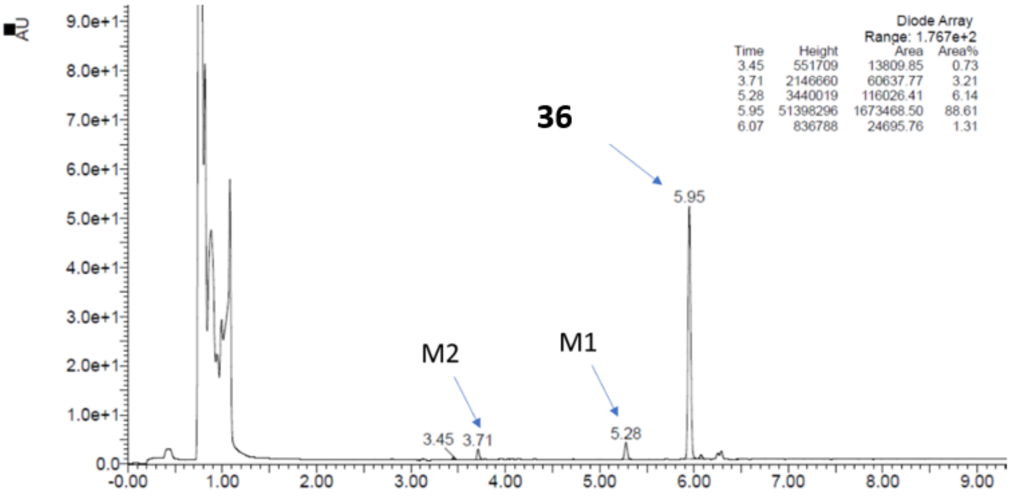

Figure S20. UPLC spectra after 120 min incubation of compound **36** with human liver microsomes in TRIS buffer pH=7.4 at 37 ºC. 88.61% of the parent compound remained in the reaction mixture. Two metabolites **M1** and **M2** were identified.

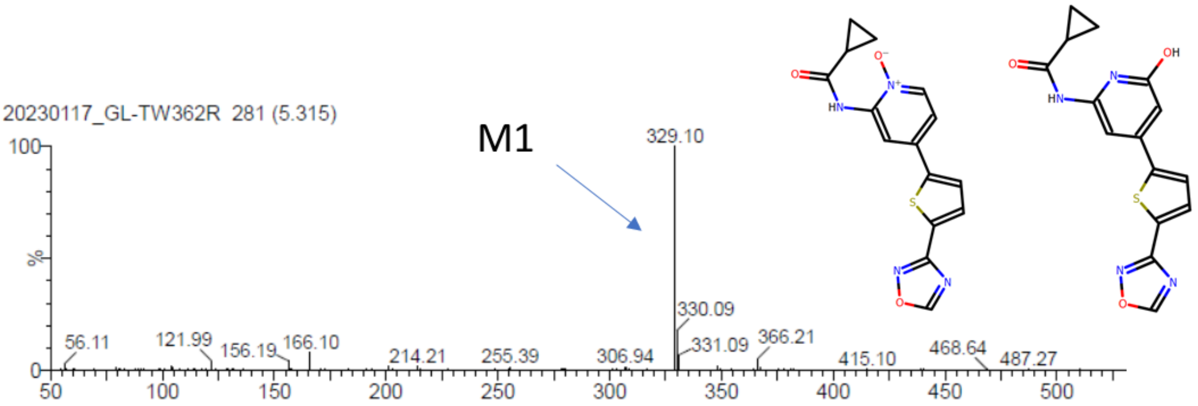

Figure S21. MS analysis and the proposed by MetaSite software most probable structures of **36** hydroxylated/oxidized metabolite **M1**.

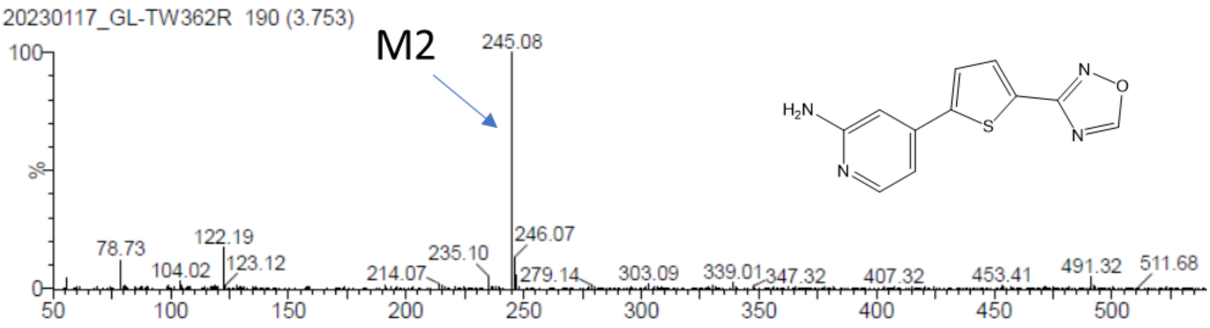

Figure S22. MS analysis and the proposed structure of **36** metabolite **M2** obtained after decomposition of the substrate.

Influence on CYP3A4, CYP2D6 and CYP2C9 activity

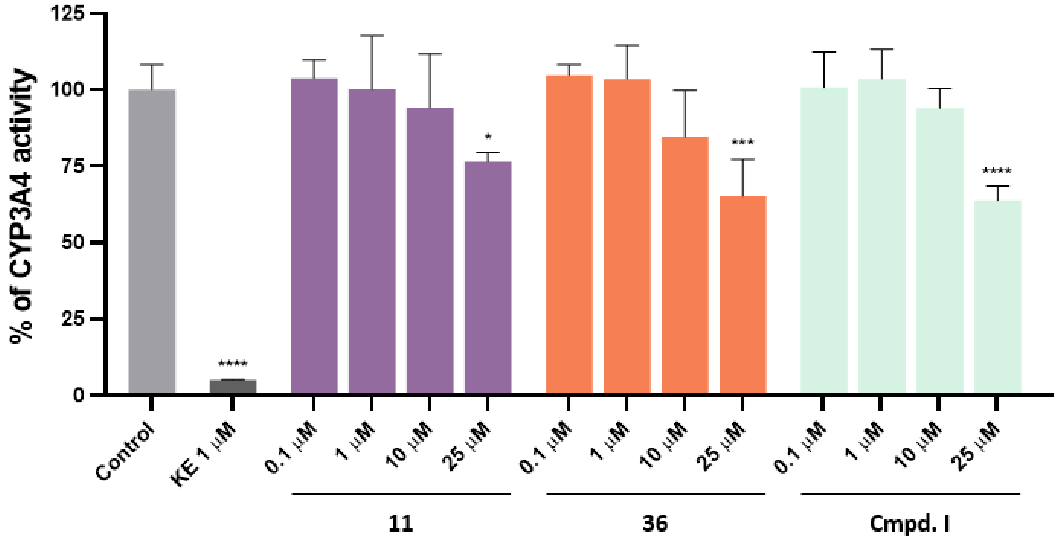

Figure S23. Effect of ketoconazole (KE), compounds **11**, **36**, and reference compound **I** on CYP3A4 activity.

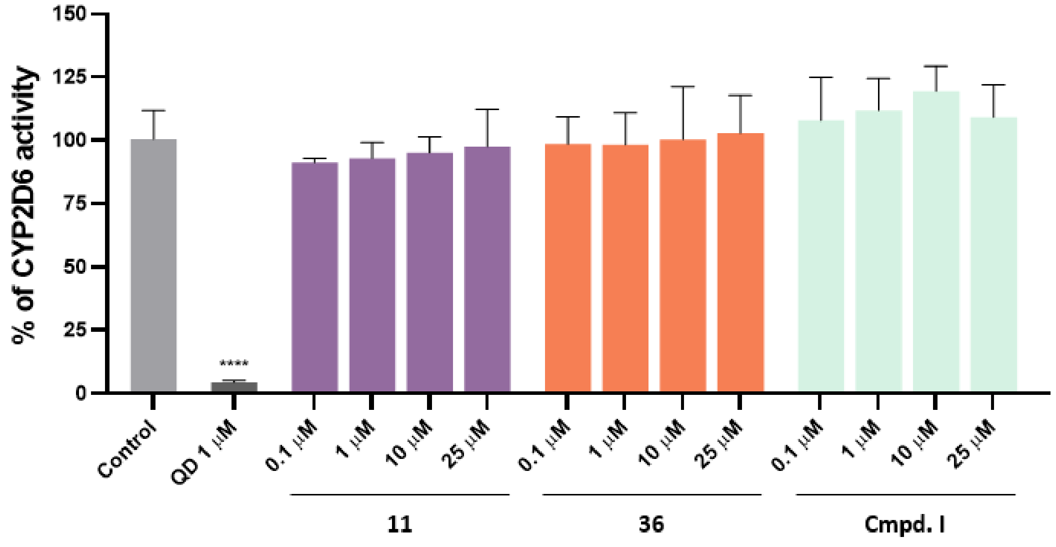

Figure S24. Effect of quinidine (QD), compounds **11**, **36**, and reference compound **I** on CYP2D6 activity.

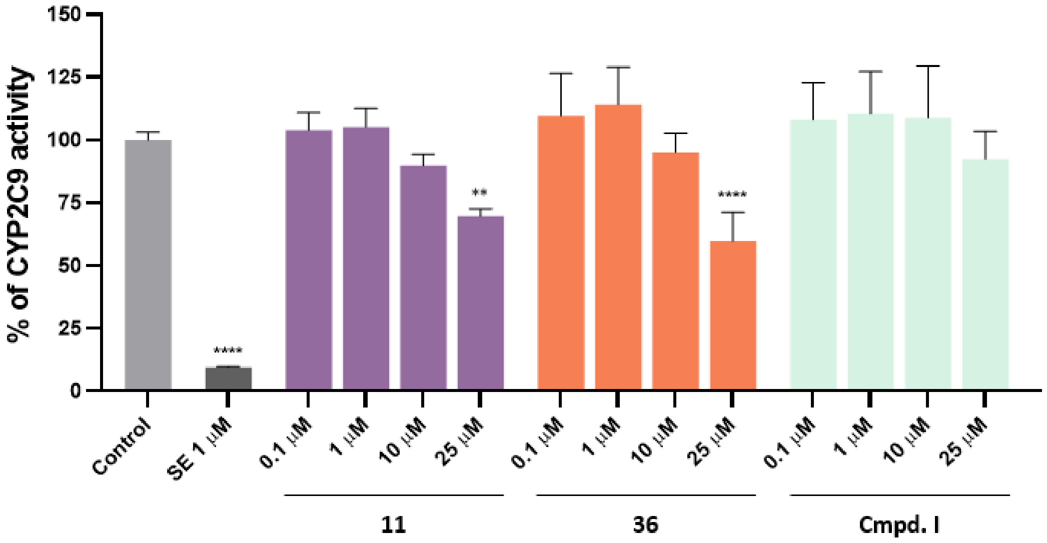

Figure S25. Effect of sulfaphenazole (SE), compounds **11**, **36**, and reference compound **I** on CYP2C9 activity.

Table S11. CYPs safety profile for **11**, **36**, and compound **I**.

Influence on CYPs P450 cytochromes (% of control ± SD)[a]	**11**	**36**	**cmpd. I**
CYP3A4 activity at 0.1 µM	103.8 ± 5.9	104.8 ± 3.4	100.8 ± 11.6
1 µM	100.1 ± 17.6	103.6 ± 11.0	103.6 ± 9.6
10 µM	94.2 ± 17.5	84.6 ± 15.1	94.0 ± 6.5
25 µM	76.4 ± 2.9	65.3 ± 11.9	63.8 ± 4.6
CYP2D6 activity at 0.1 µM	91.2 ± 1.7	98.4 ± 10.8	107.8 ± 17.1
1 µM	93.0 ± 6.0	98.1 ± 12.7	111.8 ± 12.6
10 µM	95.2 ± 6.2	100.4 ± 20.8	119.3 ± 9.9
25 µM	97.4 ± 14.8	102.8 ± 15.0	109.1 ± 12.9
CYP2C9 activity at 0.1 µM	103.9 ± 7.0	109.5 ± 17.0	108.1 ± 14.8
1 µM	105.2 ± 7.4	114.1 ± 14.9	110.4 ± 16.7
10 µM	89.8 ± 4.7	95.2 ± 7.5	108.8 ± 20.8
25 µM	69.7 ±3.04	59.8 ± 11.5	92.4 ± 11.2

[a]CYPs safety profile (P450-Glo™ kit (Promega, Madison, WI, USA).

Kinetics of GSK-3β inhibition by compound 36

Kinetic Studies of GSK-3β inhibition were performed with the most potent inhibitor **36** and diverse ATP concentrations to determine the mechanism of enzyme - inhibitor interaction. The obtained graphical representation of the Lineweaver-Burk equation (**Figure S26A**) displayed a

series of converging lines on the same point on the y-axis (1/V) confirming the unchanged V_{max} value with the increasing inhibitor concentration (increasing slopes). In addition, as more inhibitor was added to the enzymatic reaction, the x-axis intercepts were affected indicating the increasing K_m values. Based on obtained results the ATP-competitive type of GSK-3β inhibition by **36** was revealed - compound **36** binds only to the free enzyme and prevents ATP from binding. The type of enzyme inhibition was confirmed by the Cornish−Bowden plots obtained for **36** (ATP/V versus [I]). The parallel lines depicted in **Figure S26B** also indicate the ATP-competitive mechanism of GSK-3β inhibition. To obtain the K_i value of **36**, the data from Lineweaver-Burk plots were replotted as K_m versus [I] (**Figure S27**). The negative K_i value was read directly from the plot at the x-axis intersection.

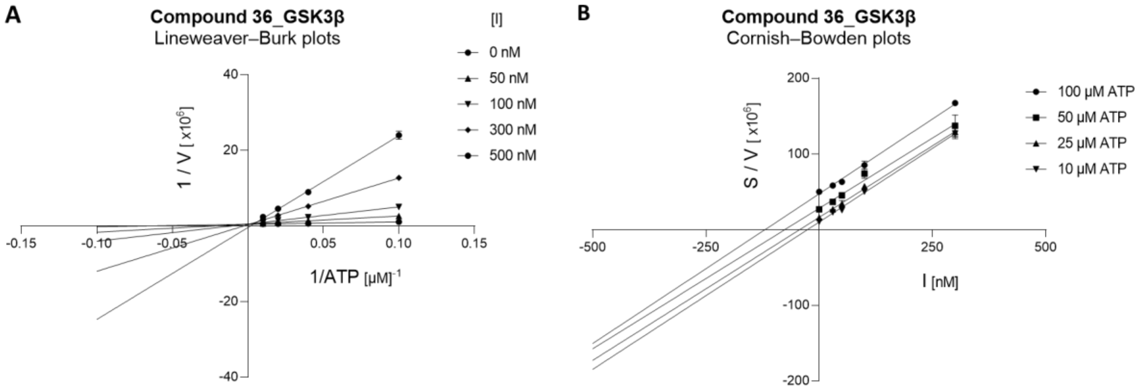

Figure S26. Lineweaver−Burk **(A)** and Cornish−Bowden **(B)** plots illustrating **ATP-competitive type** of GSK-3β inhibition by compound **36**; V = initial velocity rate, S = ATP concentration, I = inhibitor concentration

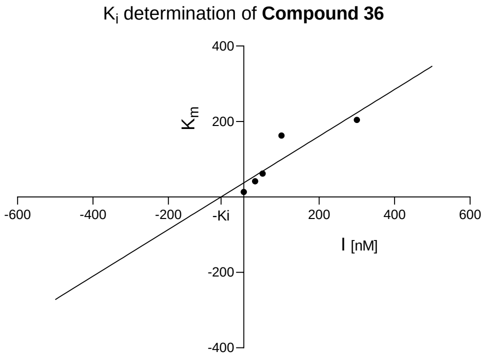

Figure S27. Replot from data from Lineveaver-Burk plots for determination of K_i value for **ATP-competitive** inhibitor **36**. Determined K_i = 60.3 nM, pK_i = 7.22; I = inhibitor concentration, K_m = Michaelis-Menten constant

Selectivity studies for compound 36

Selectivity studies were performed at Eurofins in Kinase Enzymatic Radiometric [Km ATP] Kinase Profiler Lead Hunter Assay. The percentage of enzymatic activity was measured at the concentration of 1 μM of compound **36** in duplicate for 17 selected kinases.

Table S12. Inhibitory activity of compound **36** against selected kinases.

**kinase**	% of **enzyme activity** at 1μM of **36 ±SD***
CDK1/cyclinB	42±4
CDK2/cyclinA	37±1
CDK4/cyclinD3	97±9
CDK6/cyclinD3	90±1
CDK7/cyclinH/ MAT1	93±7
CDK9/cyclin T1	16±2
DYRK1A	11±1
DYRK1B	26±6
GSK-3α	2±1
GSK-3β	5±1
JNK2α2	100±7
JNK3	73±6
MAPK1	100±1
MAPK2	98±6
SAPK2a(T106M)	95±7
SAPK2b	100±2
SAPK3	50±3

*Where n = 2, the value reported here is actually range / √ 2.

LCMS chromatograms and ^{1}H and ^{13}C NMR spectra of final compounds

N-(4-(1-Acetyl-2,5-dihydro-1*H*-pyrrol-3-yl)pyridin-2-yl)cyclopropanecarboxamide (**10**)

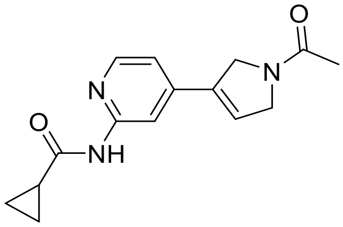

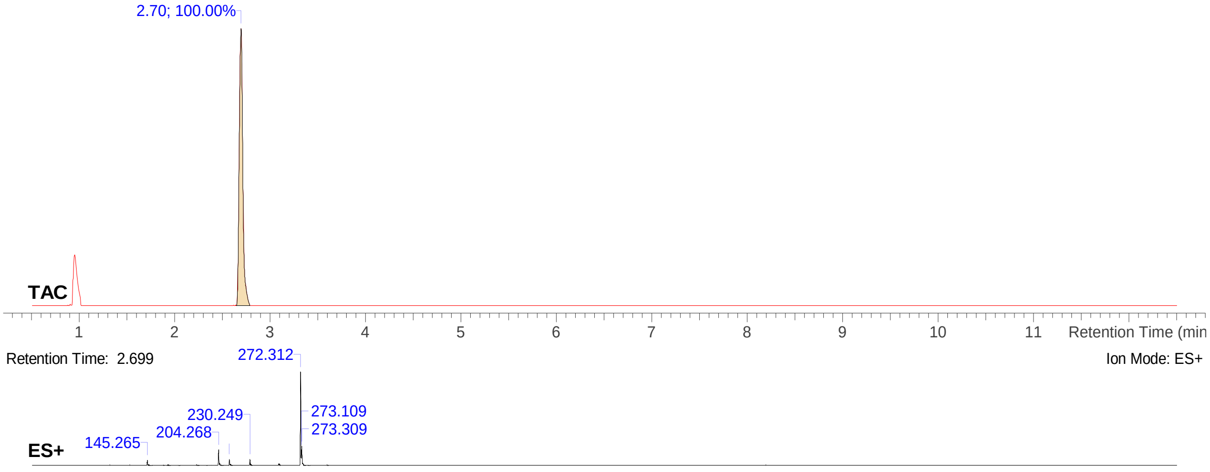

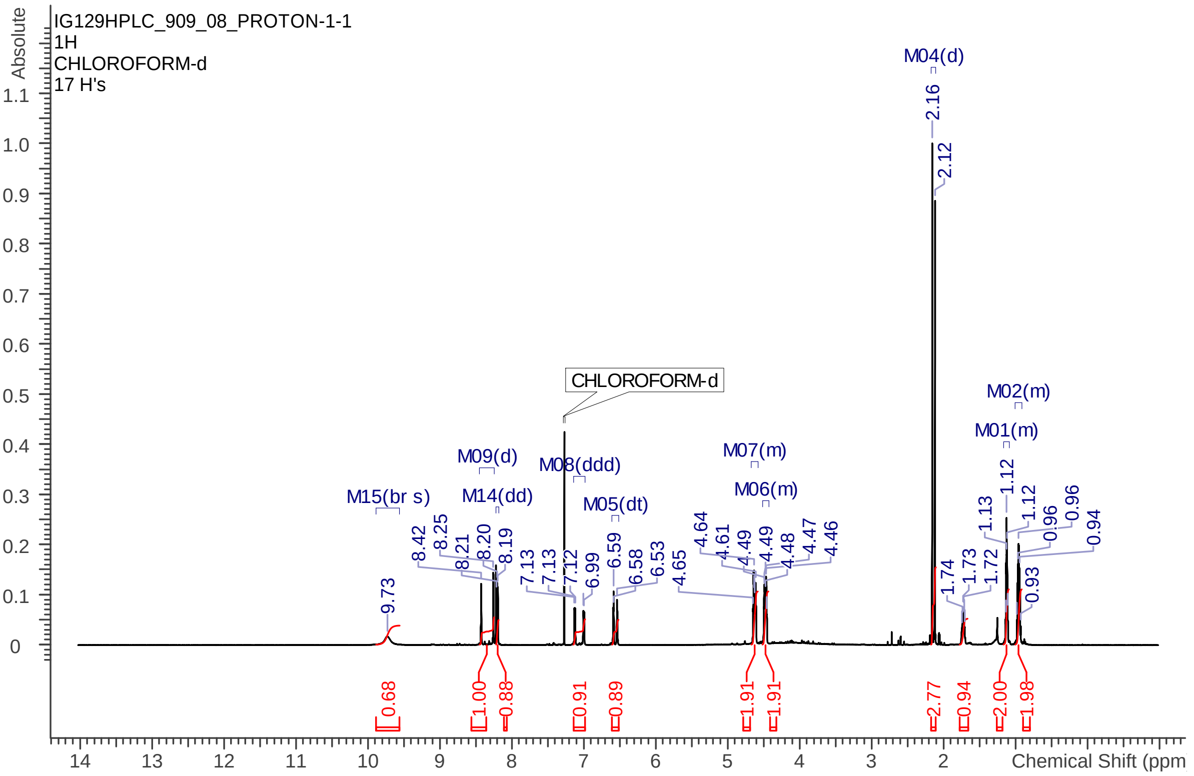

Rotamer 1:

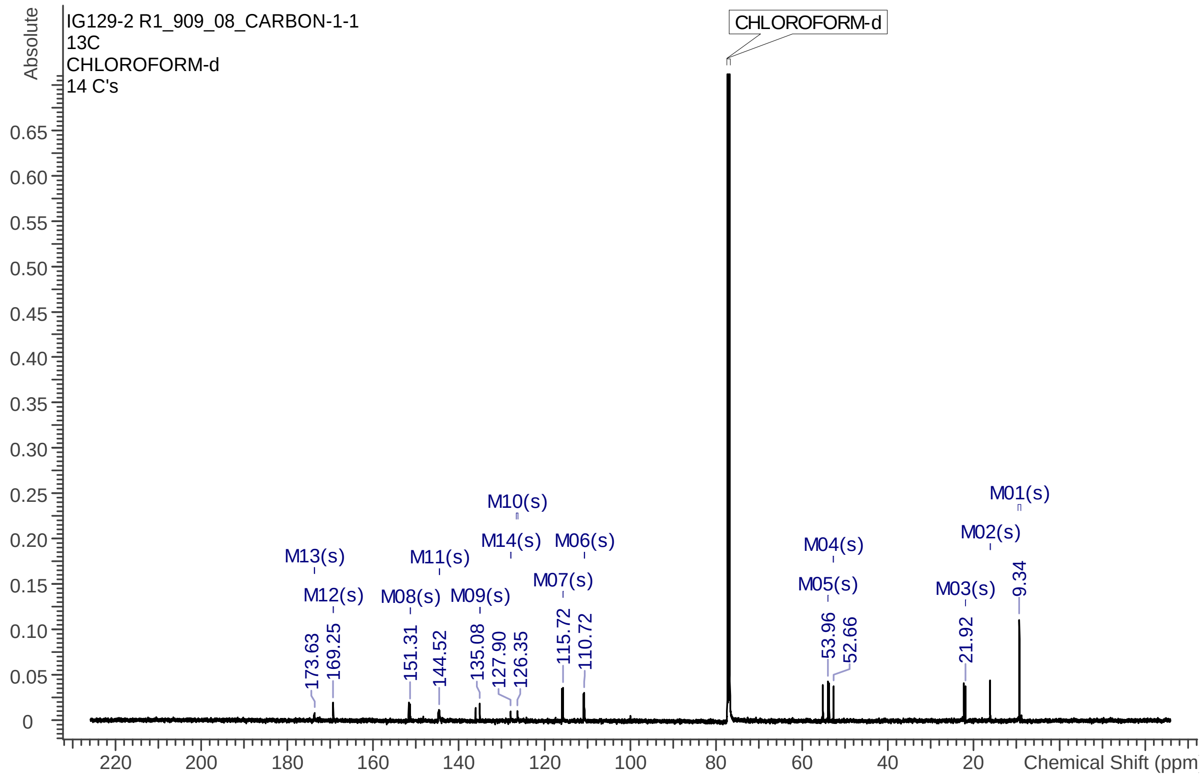

Rotamer 2:

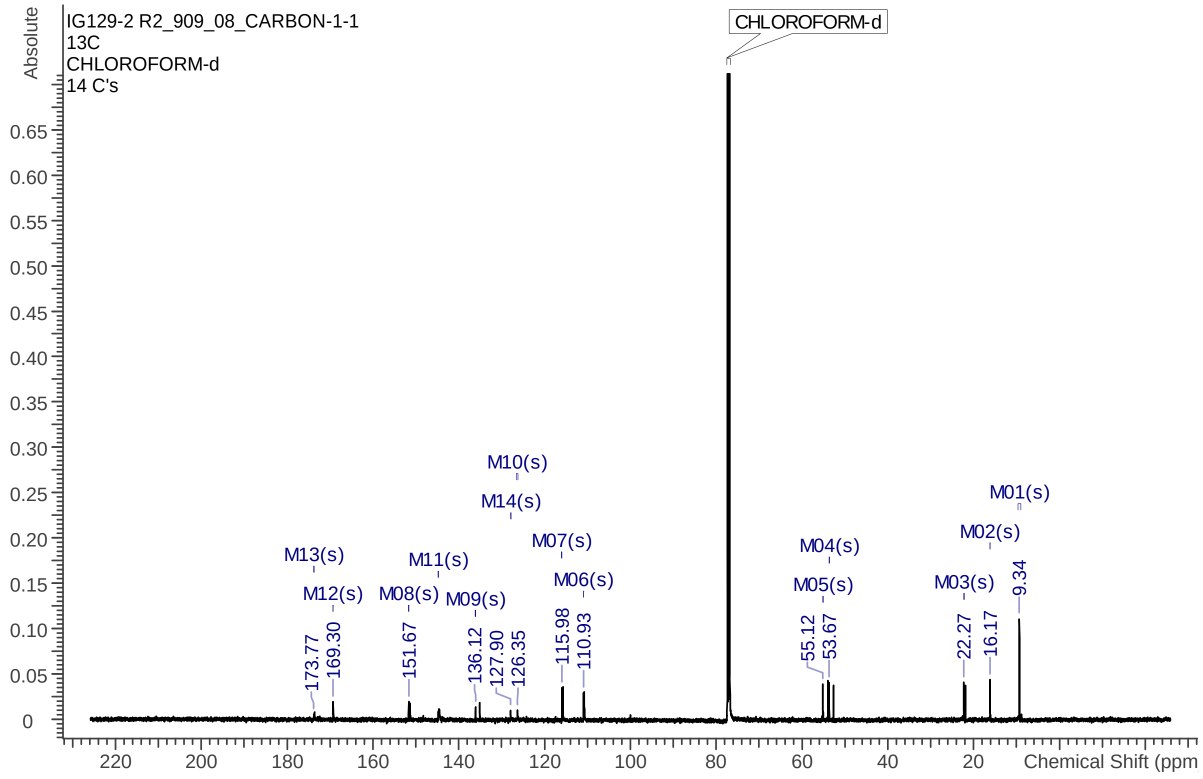

3-(2-(Cyclopropanecarboxamido)pyridin-4-yl)-2,5-dihydro-1*H*-pyrrole-1-carboxamide (**11**)

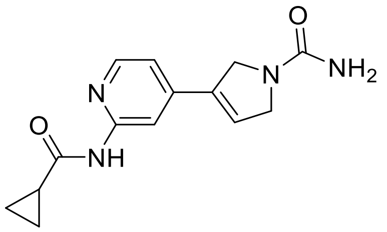

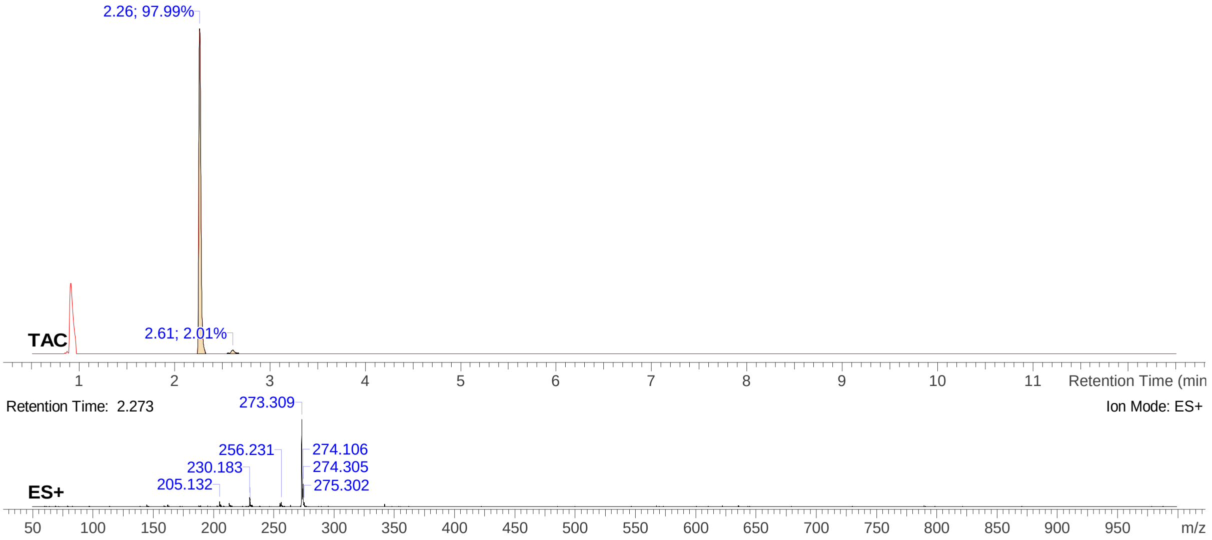

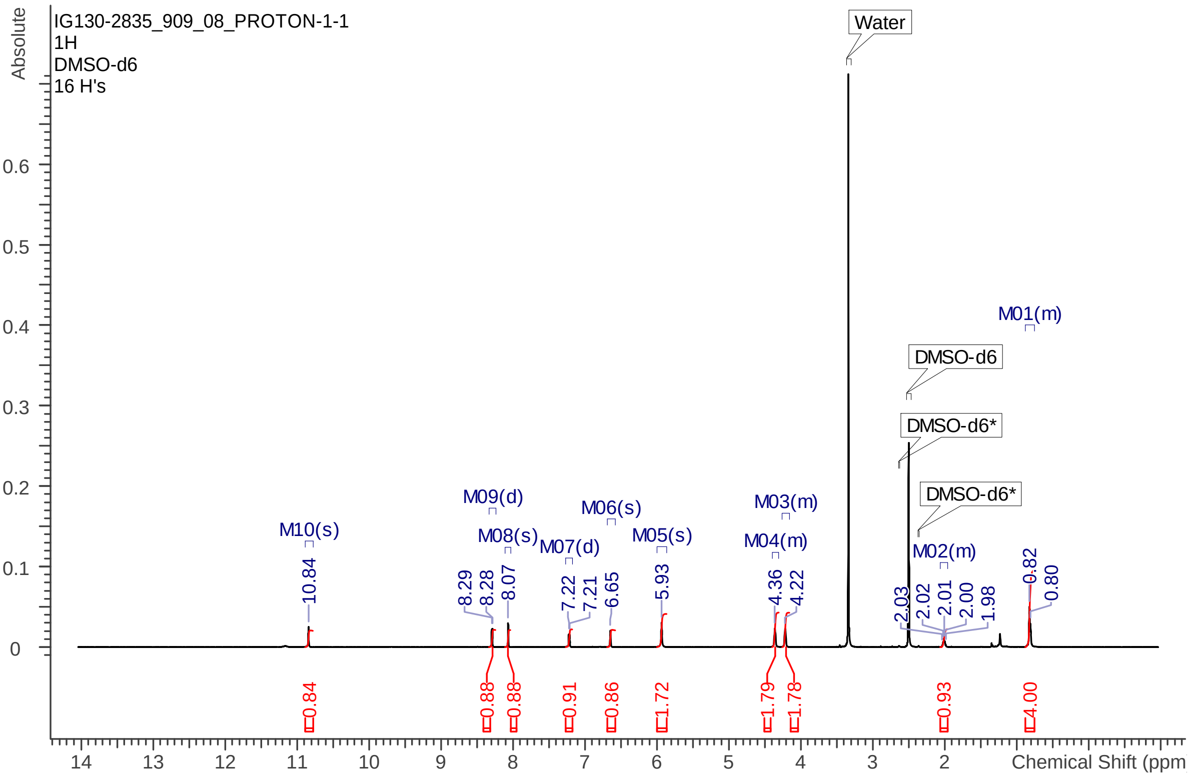

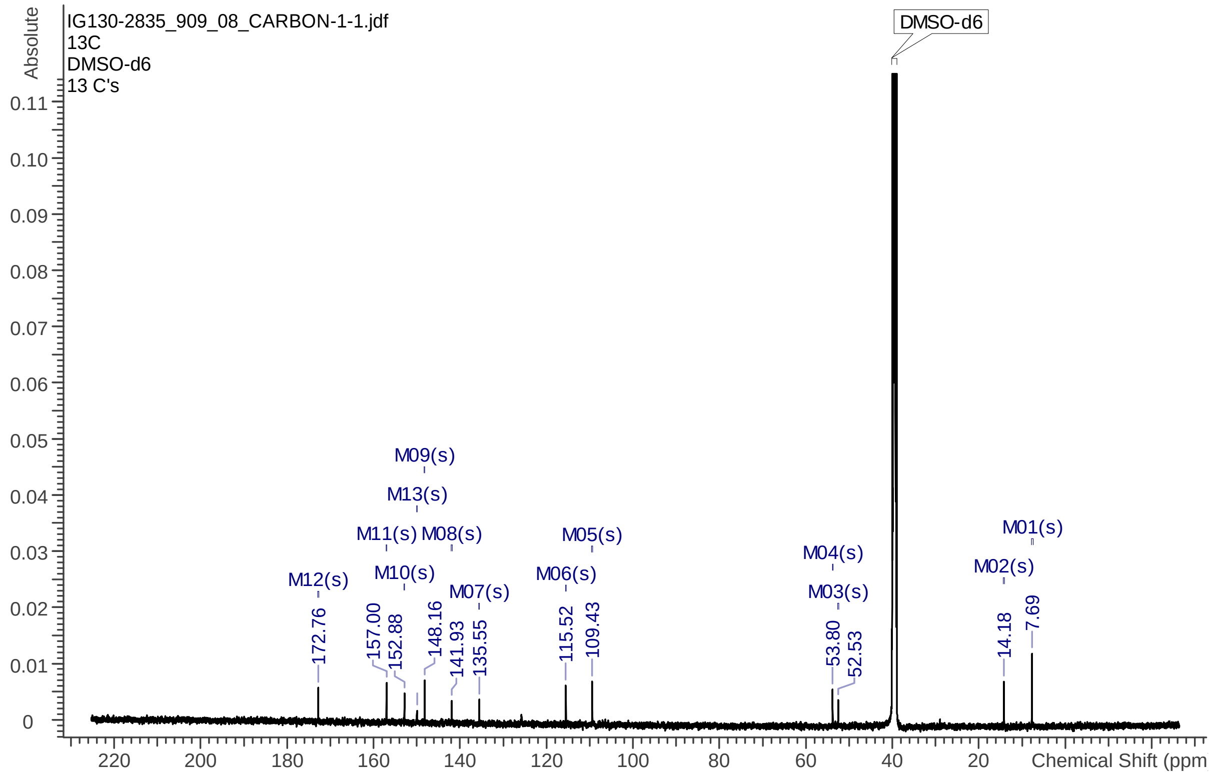

N-(4-(1-(Methylsulfonyl)-2,5-dihydro-1*H*-pyrrol-3-yl)pyridin-2-yl)cyclopropanecarboxamide (**16**)

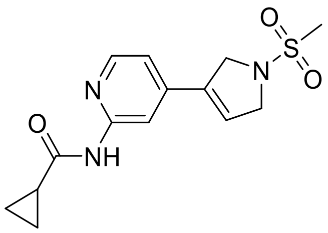

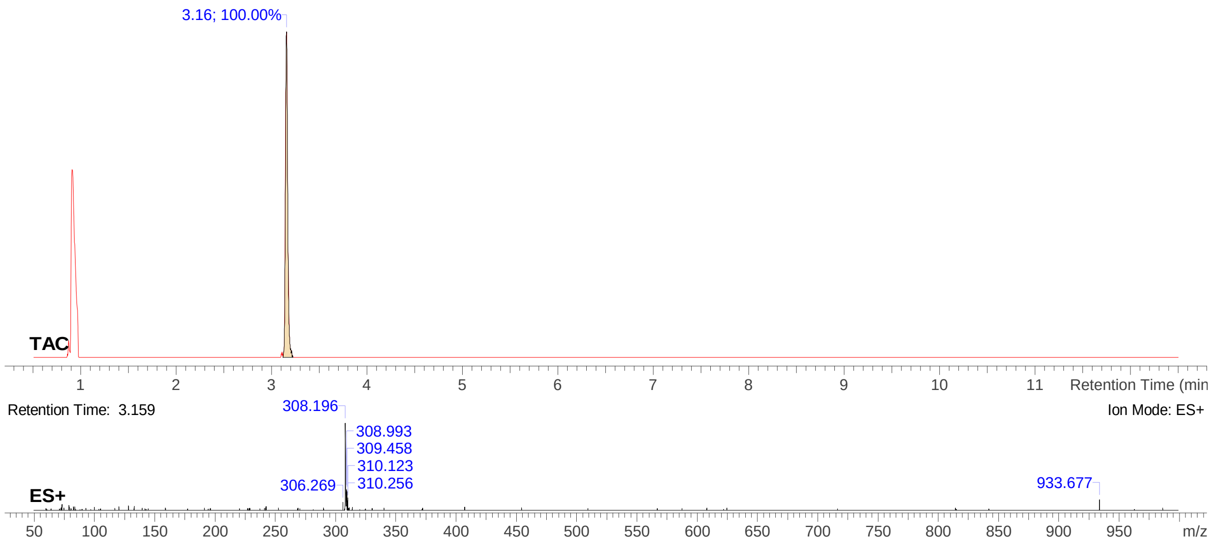

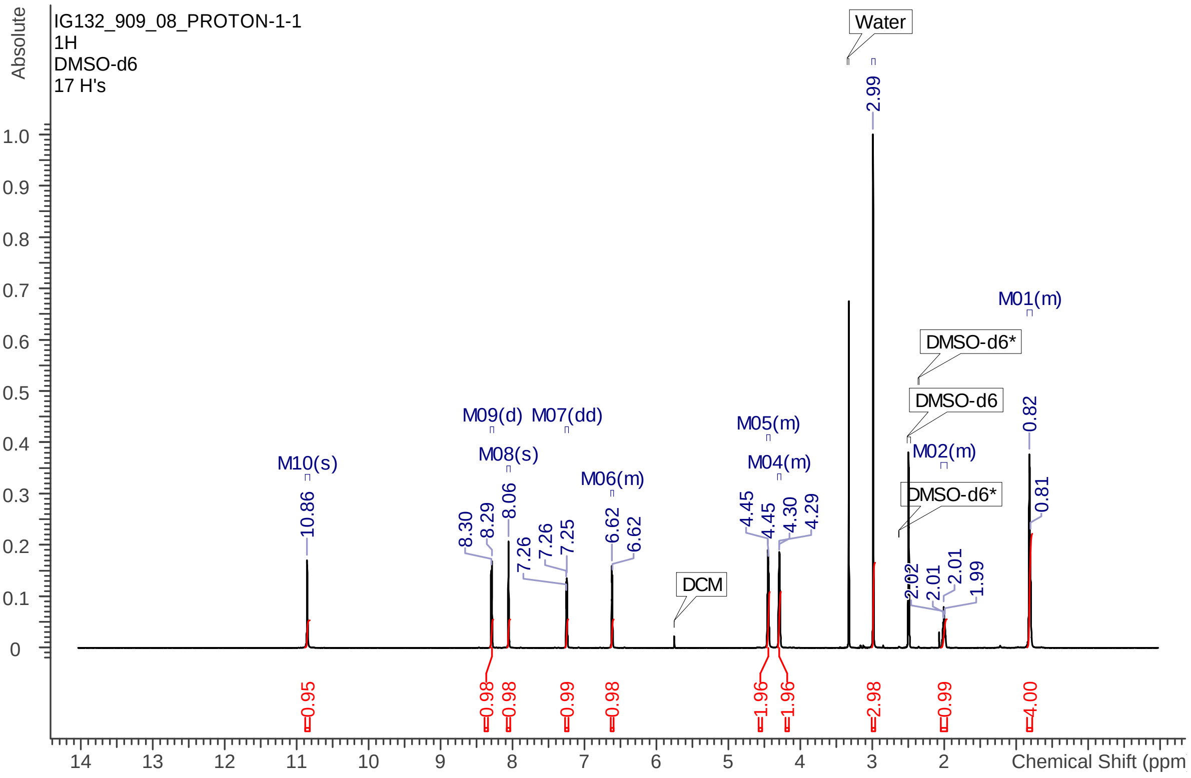
IG132_909_08_PROTON-1-1
1H
DMSO-d6
17 H's
Water
DMSO-d6*
DMSO-d6
DCM
M01(m)
M02(m)
M04(m)
M05(m)
M06(m)
M07(dd)
M08(s)
M09(d)
M10(s)
Chemical Shift (ppm

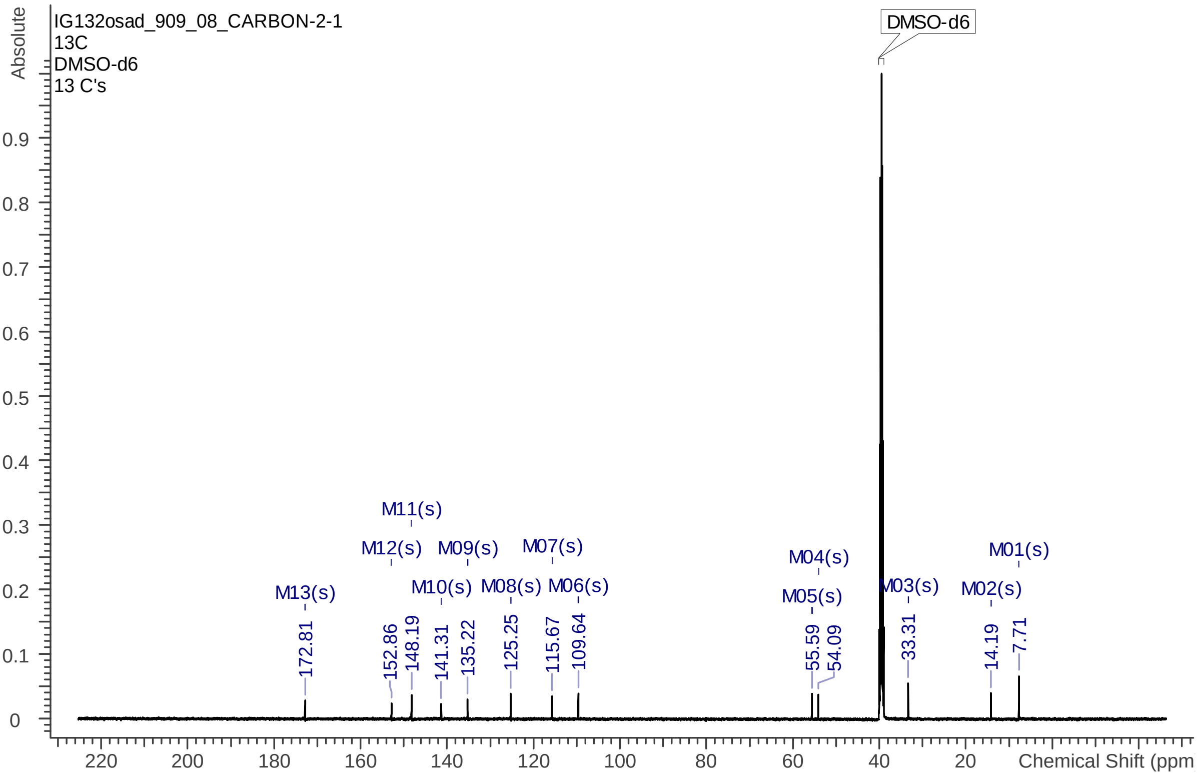
IG132osad_909_08_CARBON-2-1
13C
DMSO-d6
13 C's
DMSO-d6
M13(s) 172.81
M12(s) 152.86
M11(s) 148.19
M10(s) 141.31
M09(s) 135.22
M08(s) 125.25
M07(s) 115.67
M06(s) 109.64
M05(s) 55.59
M04(s) 54.09
M03(s) 33.31
M02(s) 14.19
M01(s) 7.71
Chemical Shift (ppm

N-(4-(1-(3-Aminopropanoyl)-2,5-dihydro-1*H*-pyrrol-3-yl)pyridin-2-yl)cyclopropanecarboxamide hydrochloride (**12**)

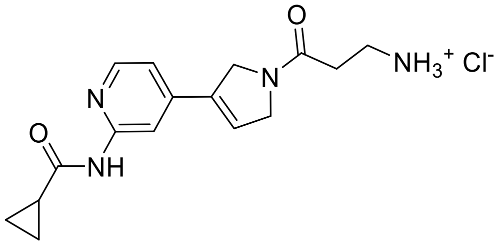

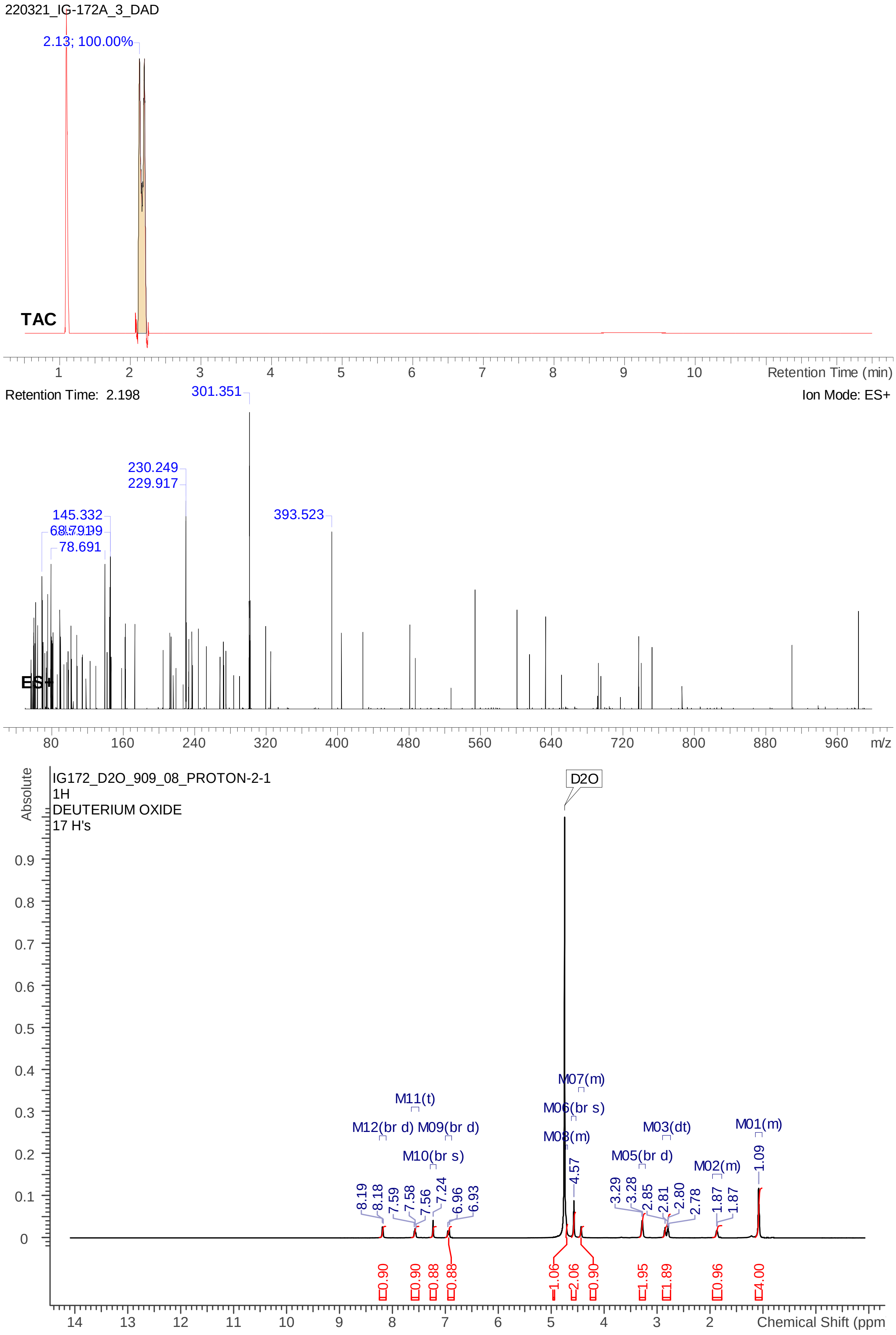
220321_IG-172A_3_DAD
2.13; 100.00%
TAC
Retention Time (min)
Retention Time: 2.198
Ion Mode: ES+
301.351
230.249
229.917
145.332
393.523
78.691
ES+
m/z
IG172_D2O_909_08_PROTON-2-1
1H
DEUTERIUM OXIDE
17 H's
D2O
Absolute
M11(t)
M12(br d) M09(br d)
M10(br s)
M07(m)
M06(br s)
M08(m)
M05(br d)
M03(dt)
M02(m)
M01(m)
Chemical Shift (ppm

Rotamer 1:

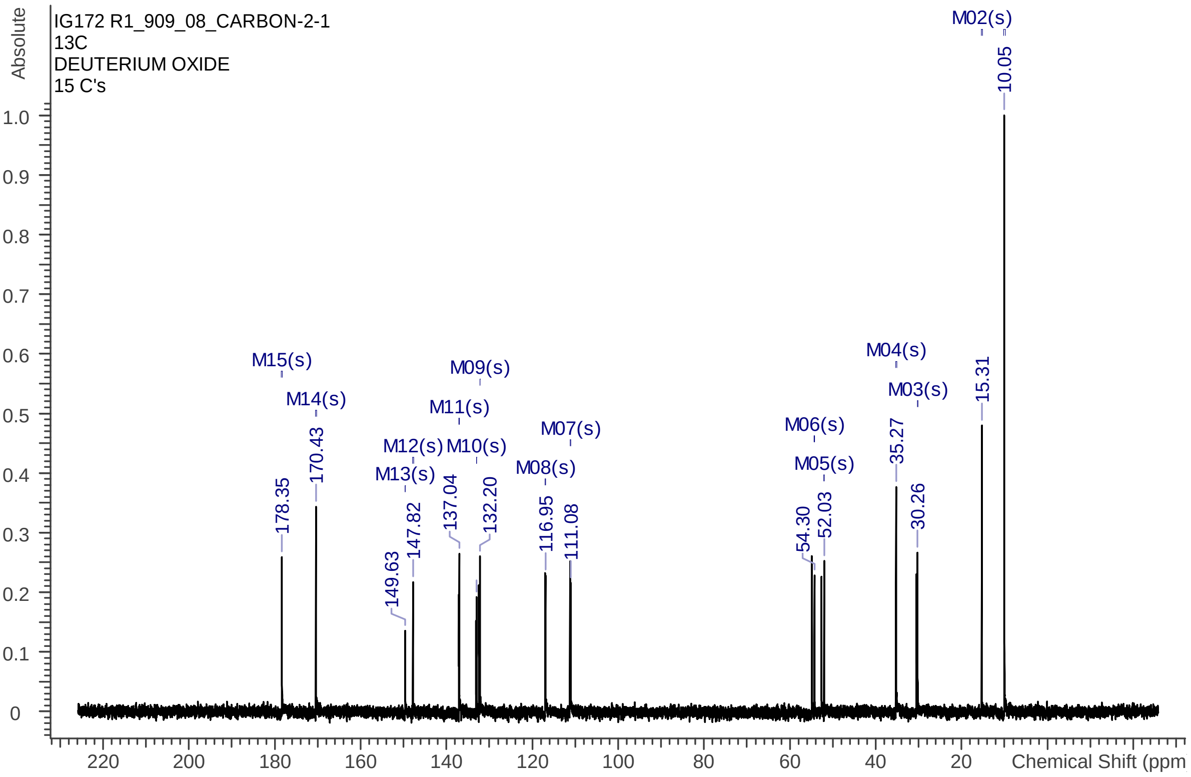

Rotamer 2:

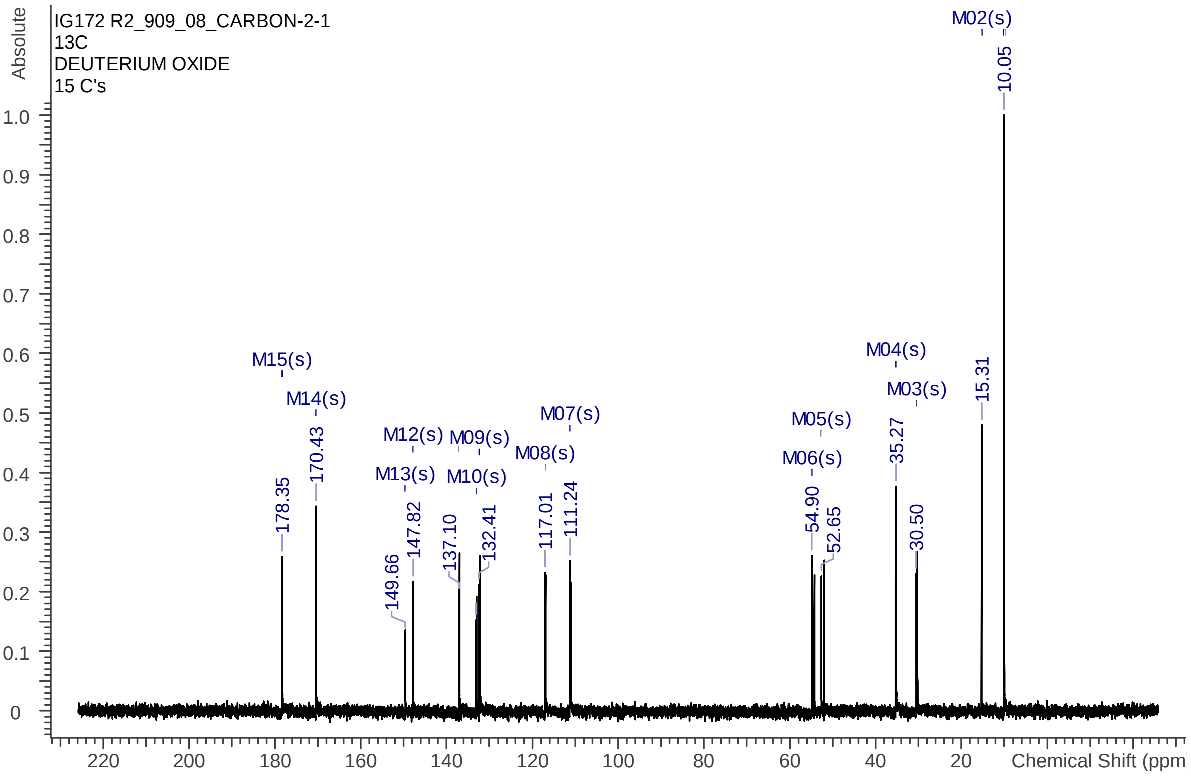

N-(4-(1-(4-Aminobutanoyl)-2,5-dihydro-1*H*-pyrrol-3-yl)pyridin-2-yl)cyclopropanecarboxamide hydrochloride (**13**)

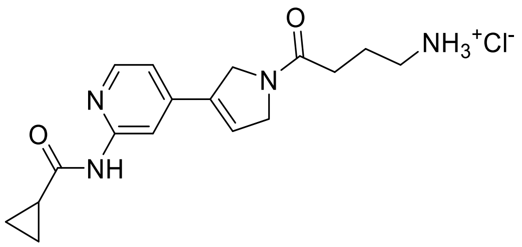

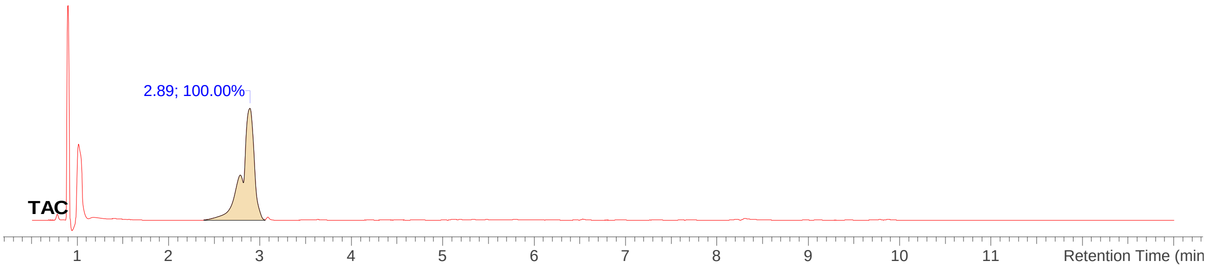

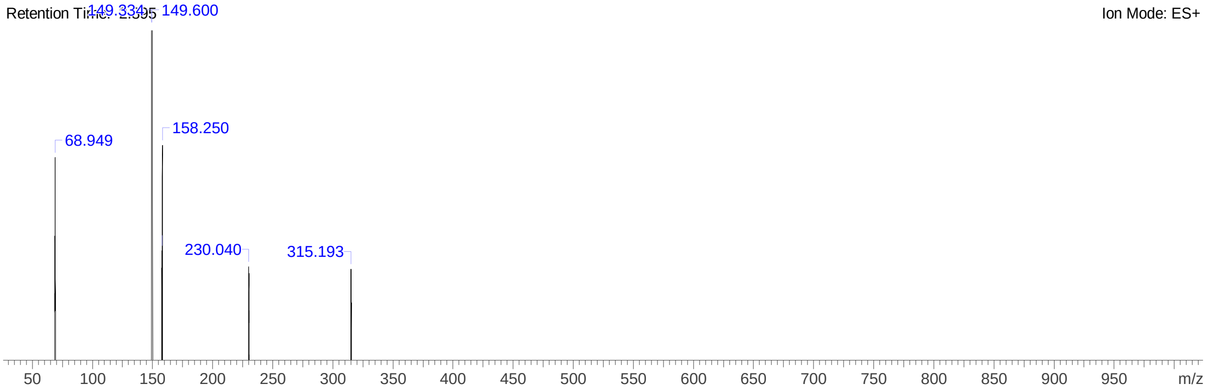

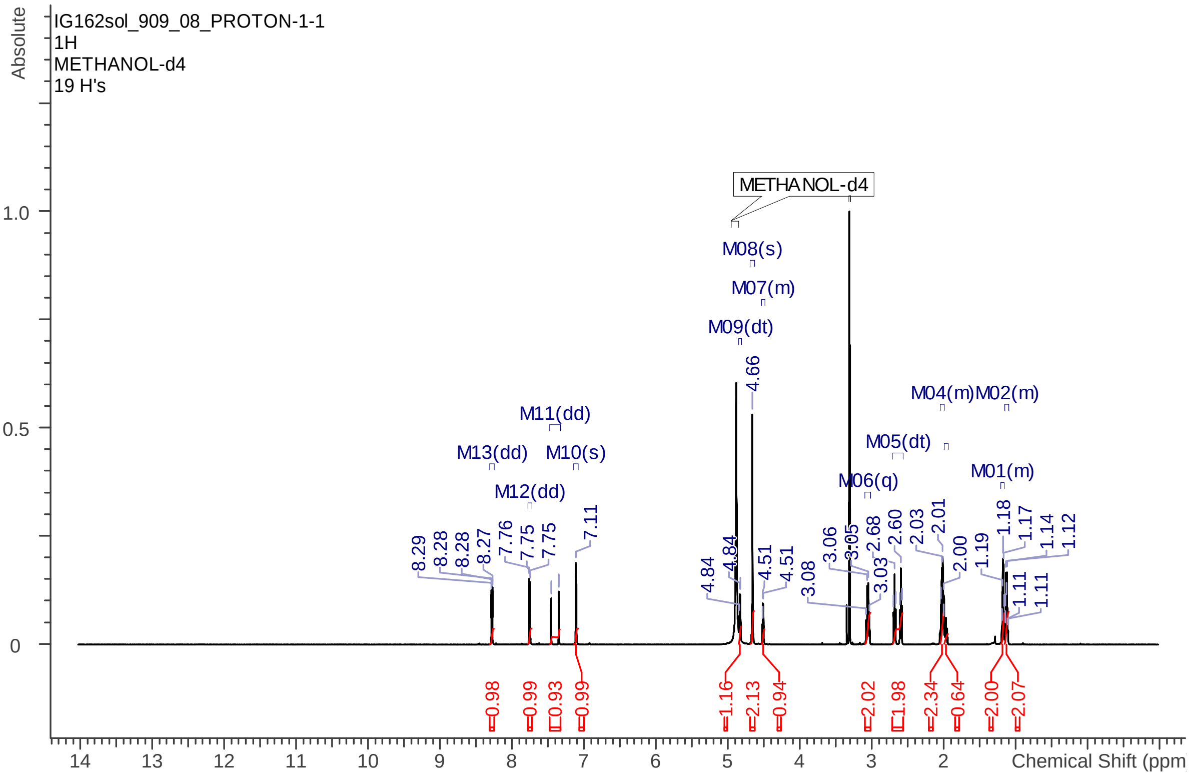

IG162sol_909_08_PROTON-1-1
1H
METHANOL-d4
19 H's
METHANOL-d4
Absolute
Chemical Shift (ppm

Rotamer 1:

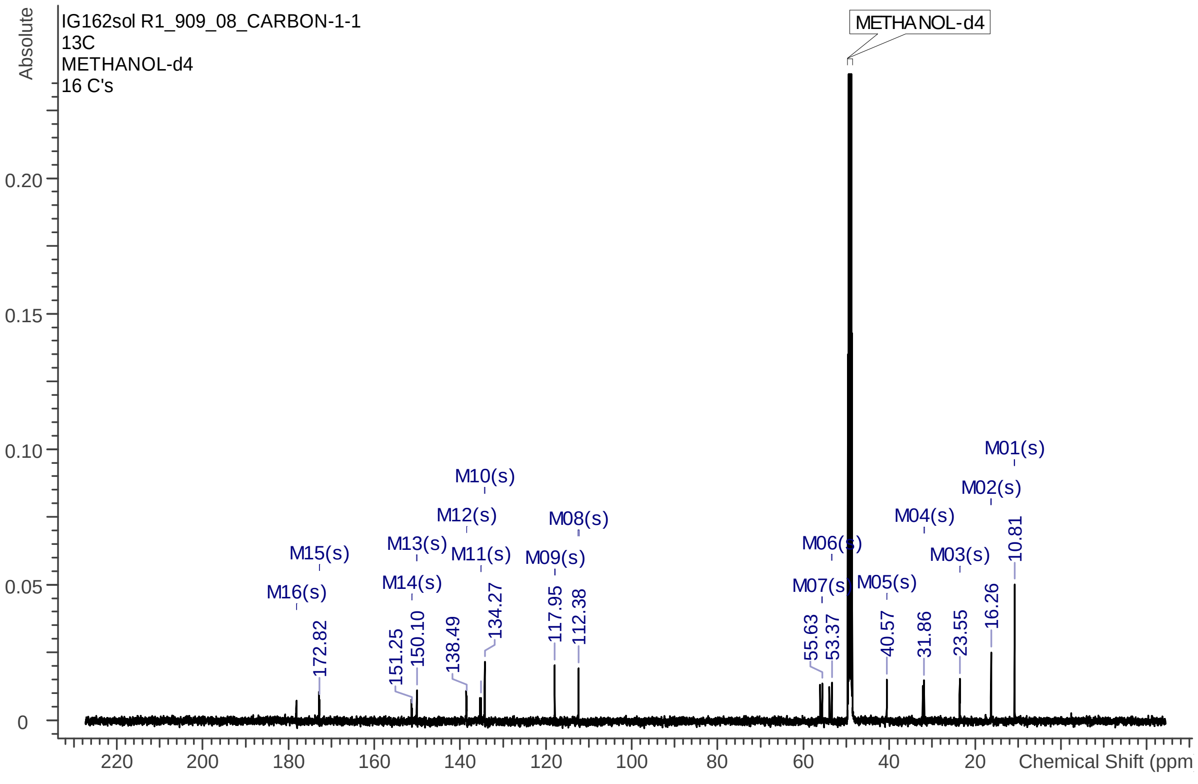

IG162sol R1_909_08_CARBON-1-1
13C
METHANOL-d4
16 C's
METHANOL-d4
Absolute
Chemical Shift (ppm

Rotamer 2:

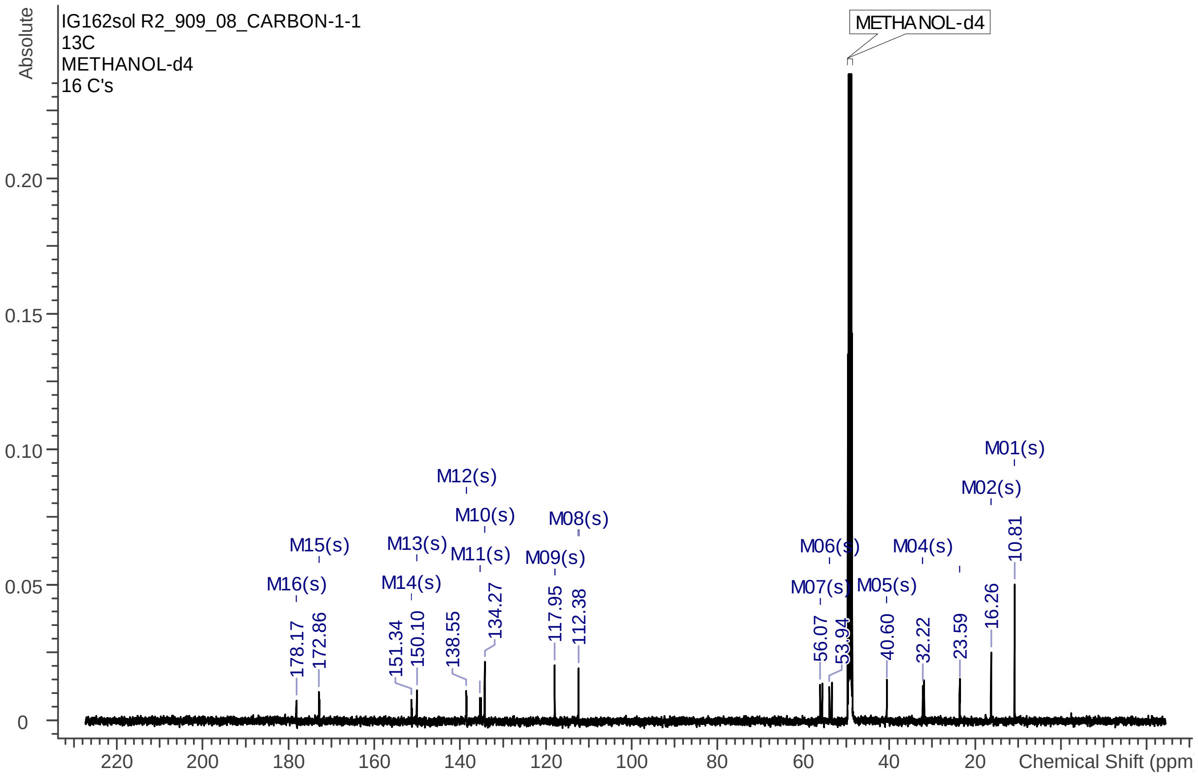

N-(4-(1-Prolyl-2,5-dihydro-1*H*-pyrrol-3-yl)pyridin-2-yl)cyclopropanecarboxamide (**14**)

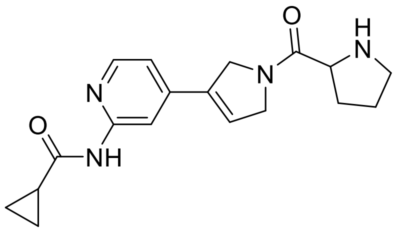

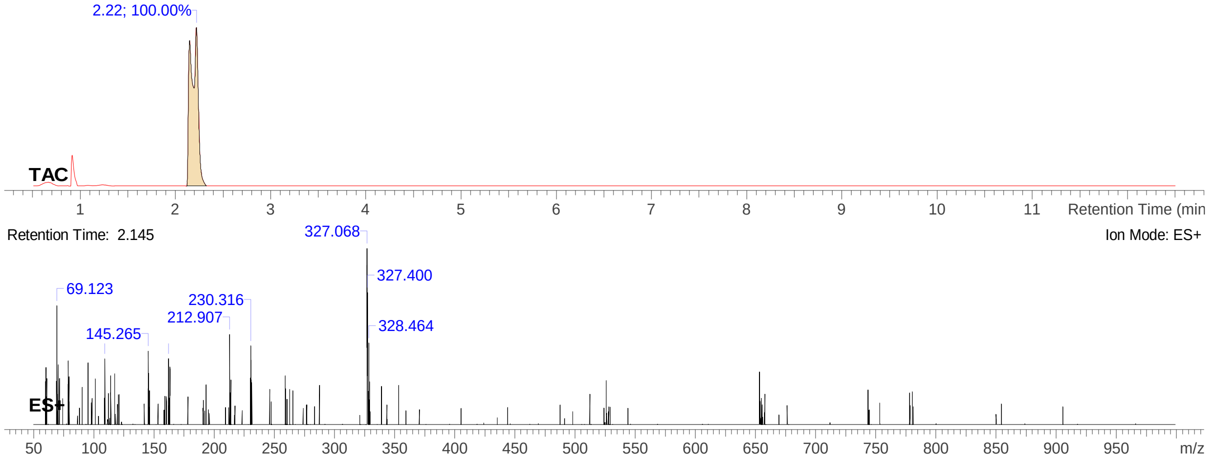

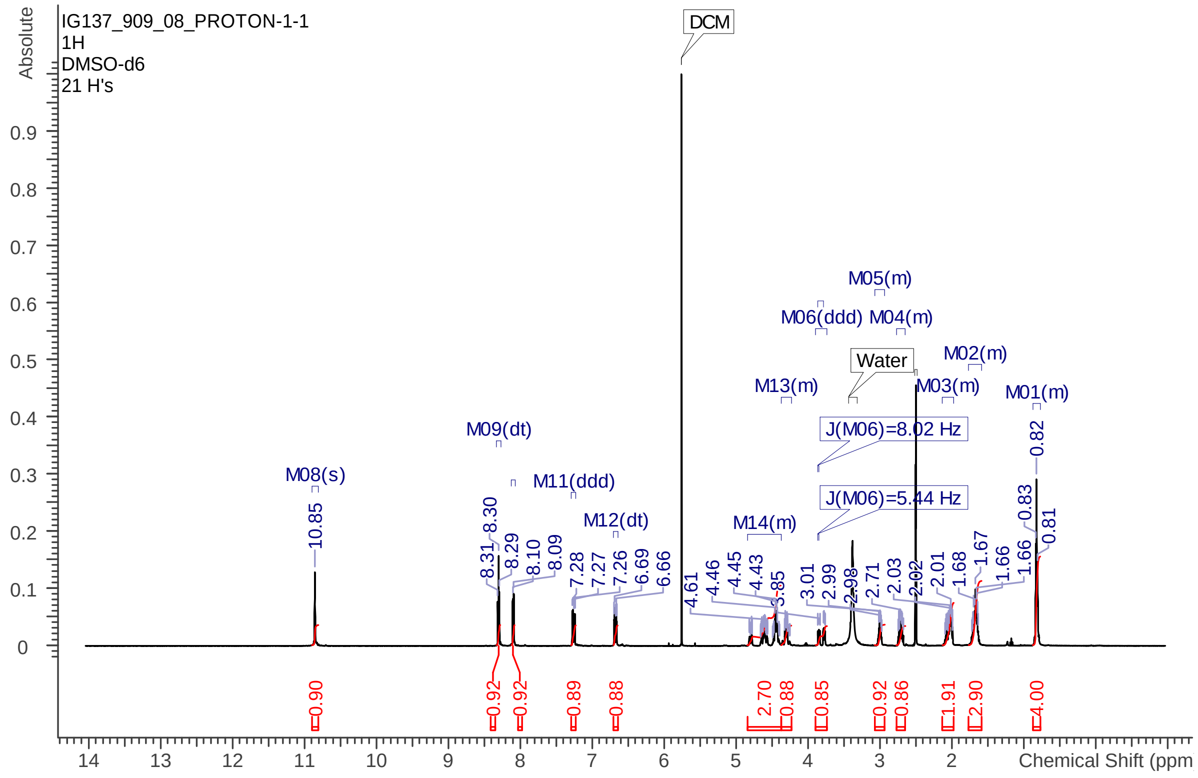
IG137_909_08_PROTON-1-1
1H
DMSO-d6
21 H's
Absolute
DCM
Water
M01(m)
M02(m)
M03(m)
M04(m)
M05(m)
M06(ddd)
M08(s)
M09(dt)
M11(ddd)
M12(dt)
M13(m)
M14(m)
J(M06)=8.02 Hz
J(M06)=5.44 Hz
Chemical Shift (ppm

Rotamer 1:

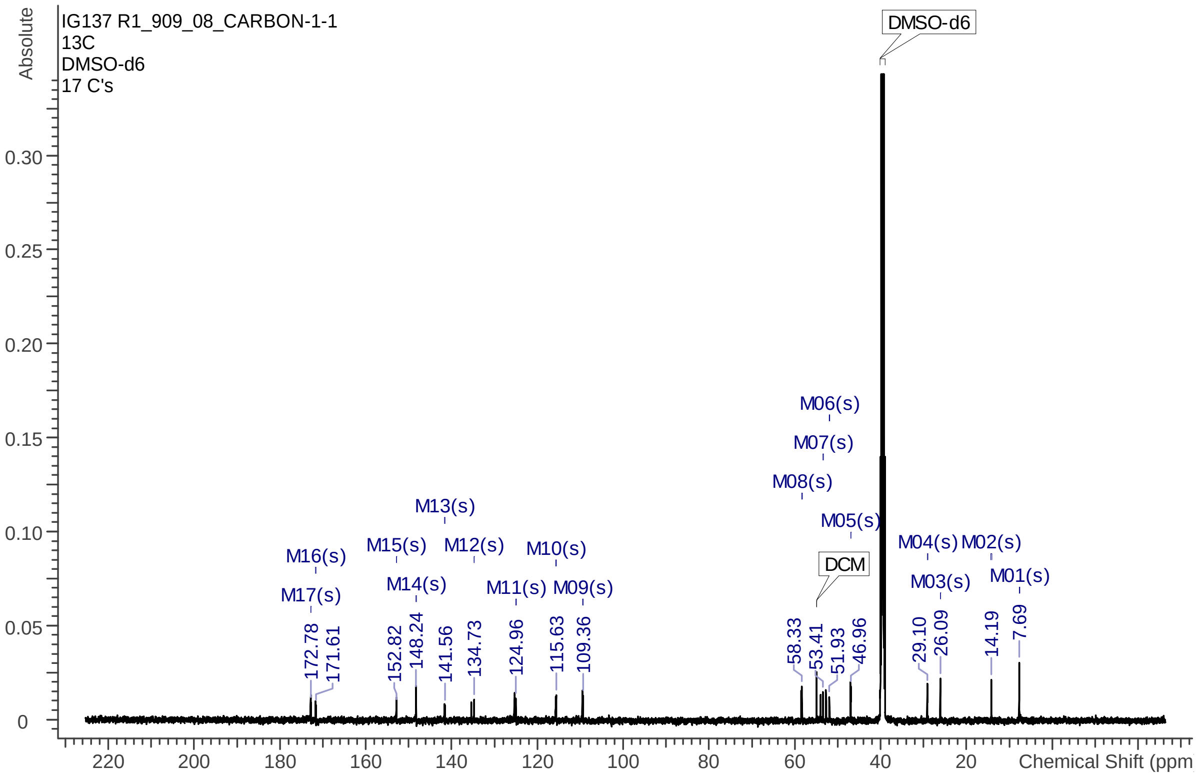
IG137 R1_909_08_CARBON-1-1
13C
DMSO-d6
17 C's
Absolute
DMSO-d6
DCM
172.78
171.61
152.82
148.24
141.56
134.73
124.96
115.63
109.36
58.33
53.41
51.93
46.96
29.10
26.09
14.19
7.69
Chemical Shift (ppm

Rotamer 2:

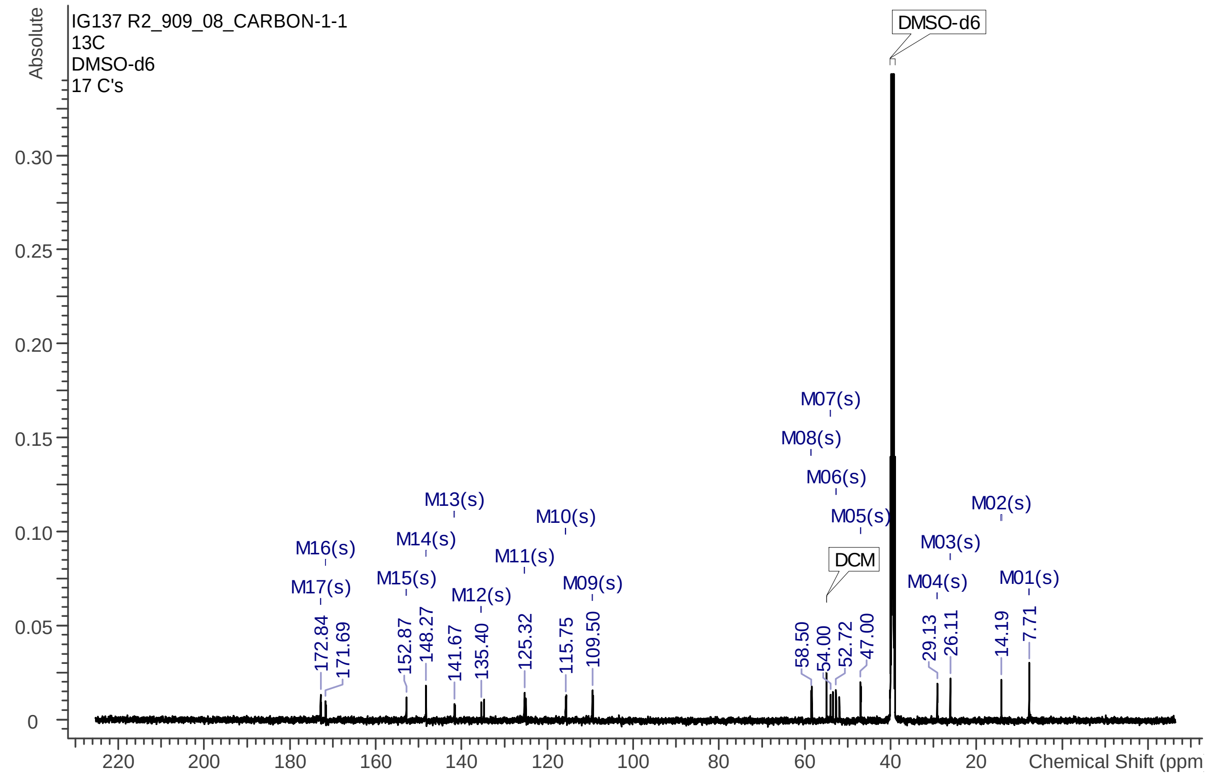

(*R*)-*N*-(4-(1-(Pyrrolidin-2-ylmethyl)-2,5-dihydro-1*H*-pyrrol-3-yl)pyridin-2-yl)cyclopropanecarboxamide hydrochloride (**(*R*)-15**)

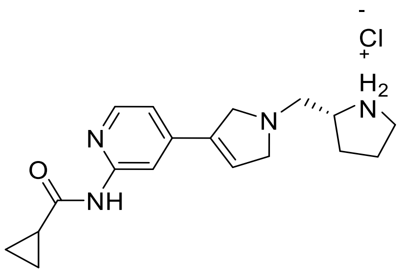

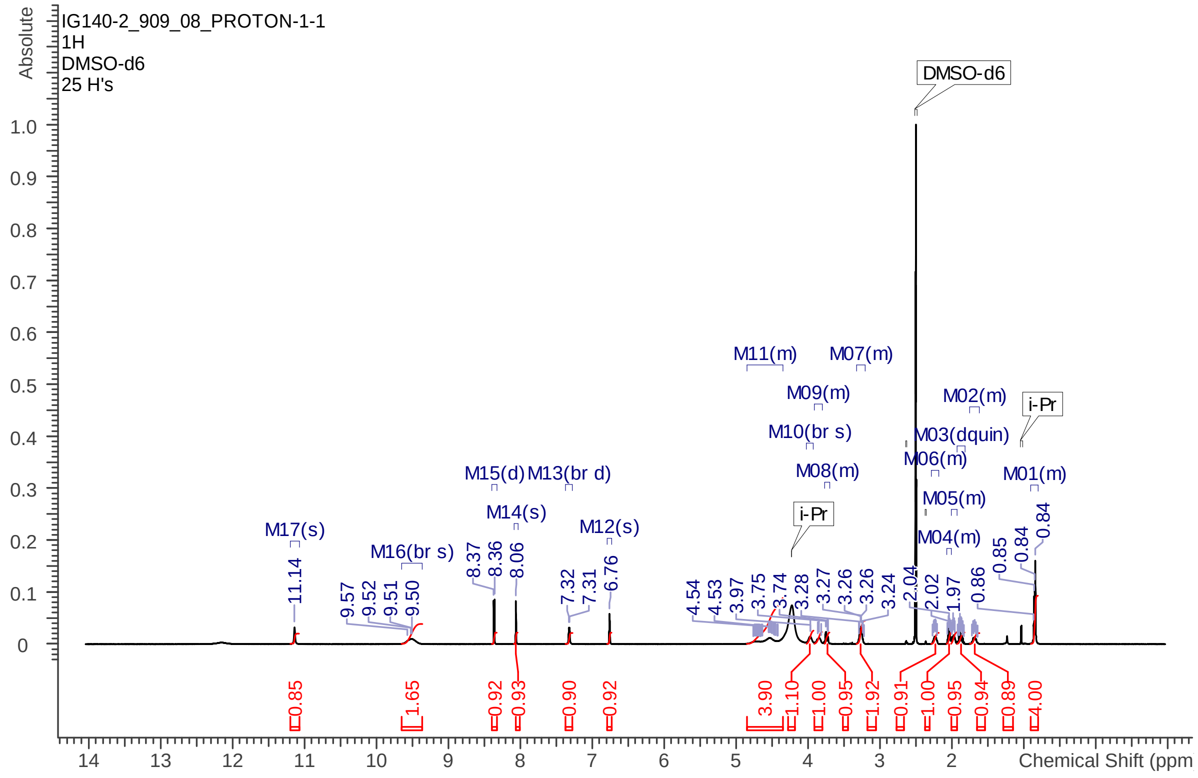
IG140-2_909_08_PROTON-1-1
1H
DMSO-d6
25 H's
DMSO-d6
i-Pr
Chemical Shift (ppm)
Absolute

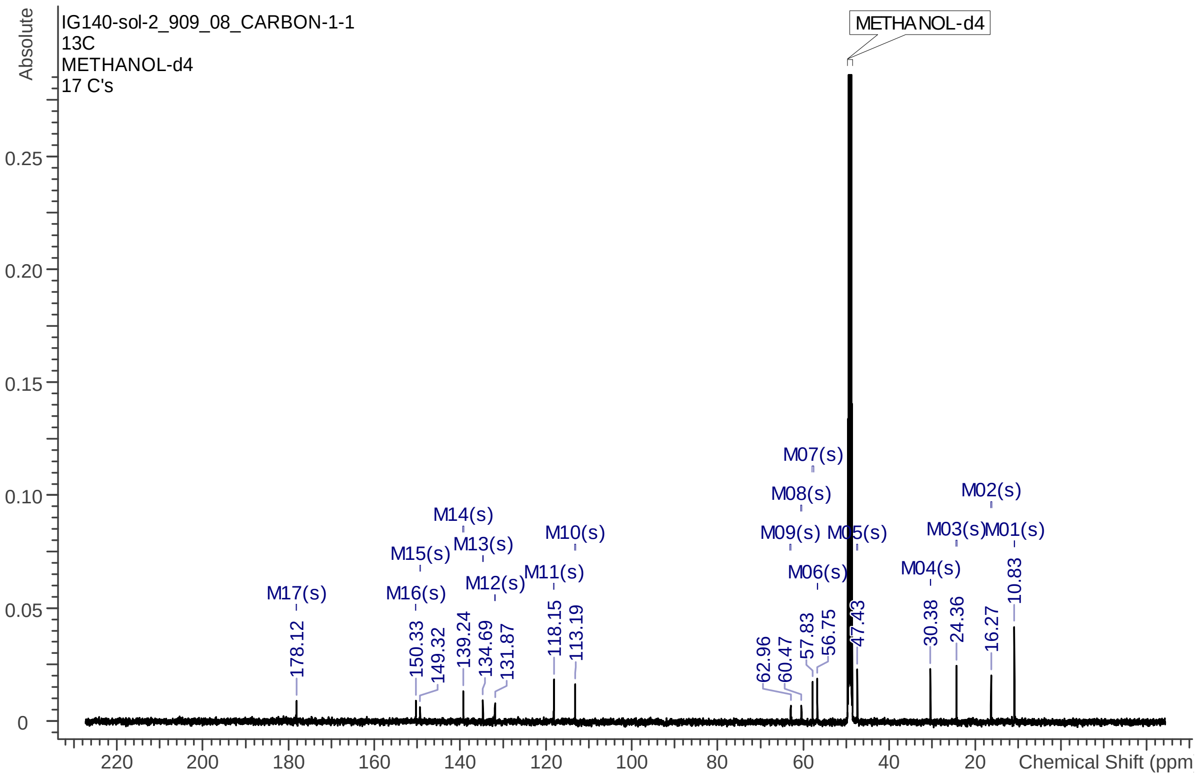
IG140-sol-2_909_08_CARBON-1-1
13C
METHANOL-d4
17 C's
METHANOL-d4
Chemical Shift (ppm)
Absolute

(*S*)-*N*-(4-(1-(Pyrrolidin-2-ylmethyl)-2,5-dihydro-1*H*-pyrrol-3-yl)pyridin-2-yl)cyclopropanecarboxamide hydrochloride ((***S***)-**15**)

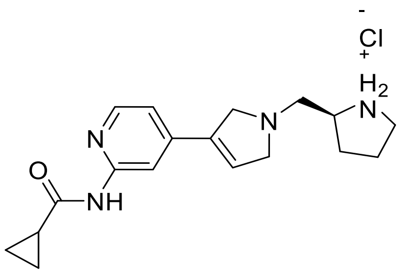

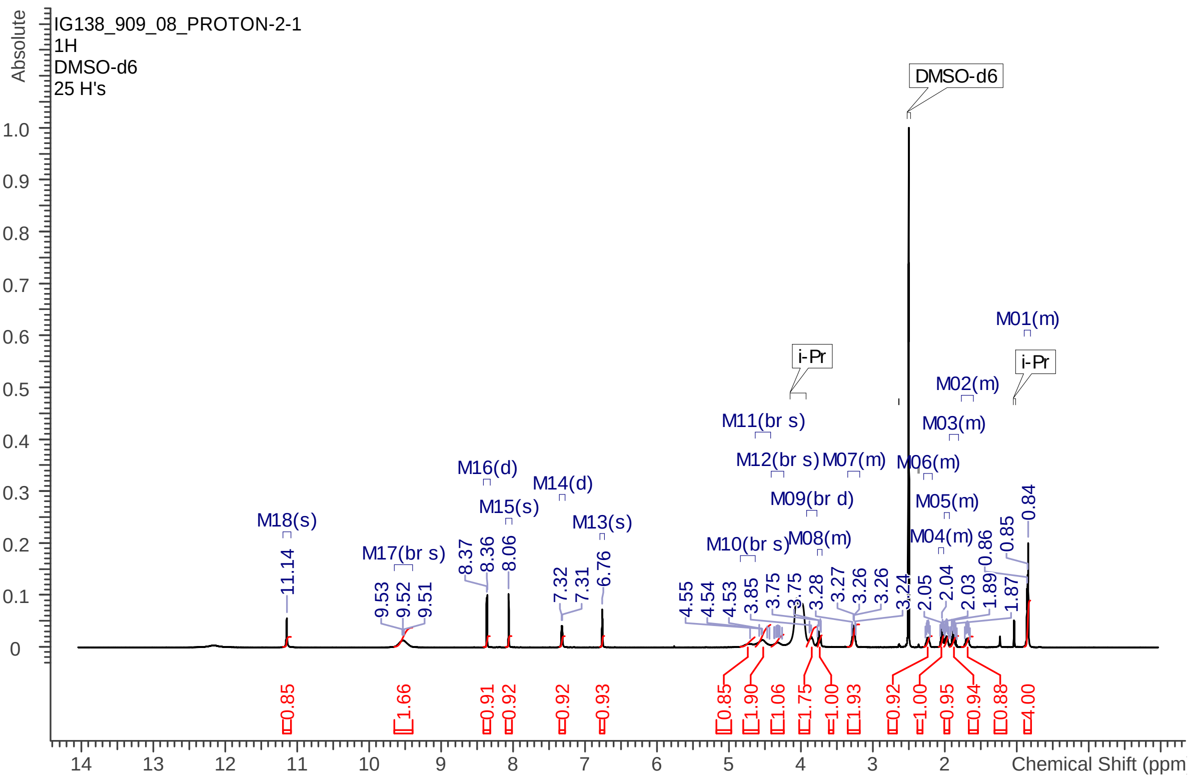

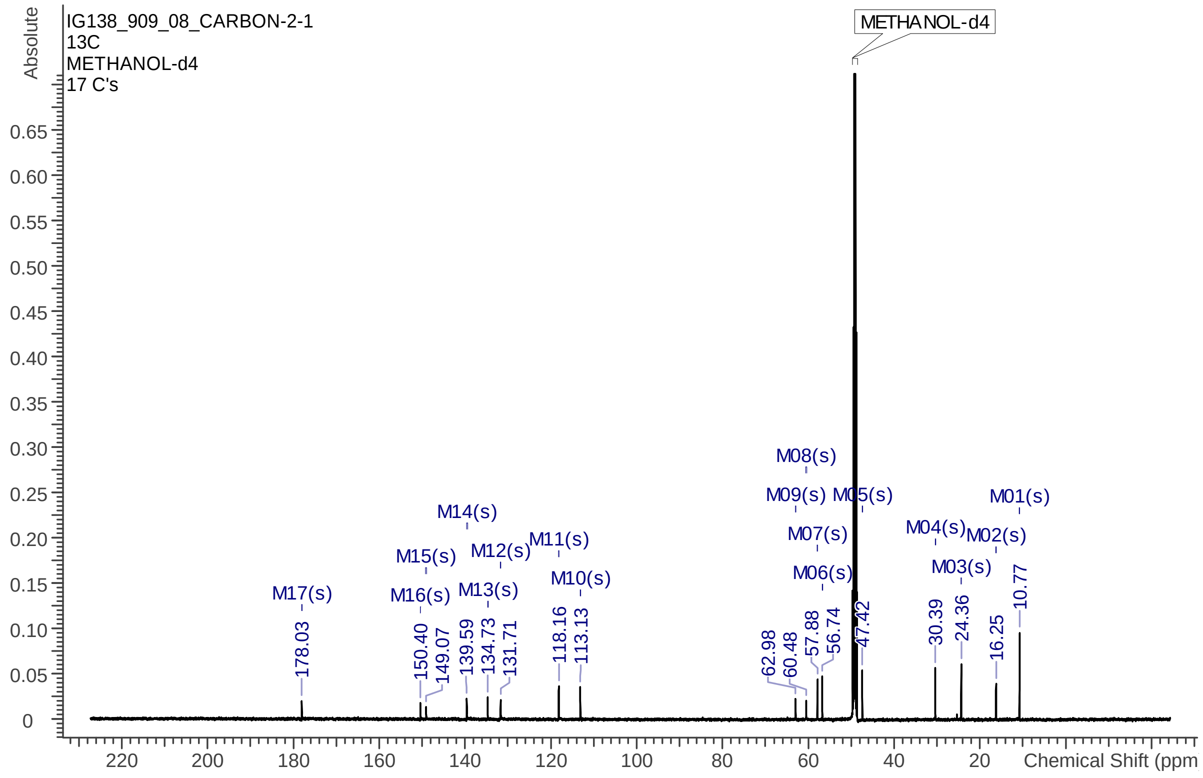

N-(4-(1-(Propylsulfonyl)-2,5-dihydro-1*H*-pyrrol-3-yl)pyridin-2-yl)cyclopropanecarboxamide (**17**)

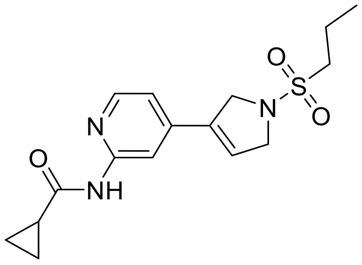

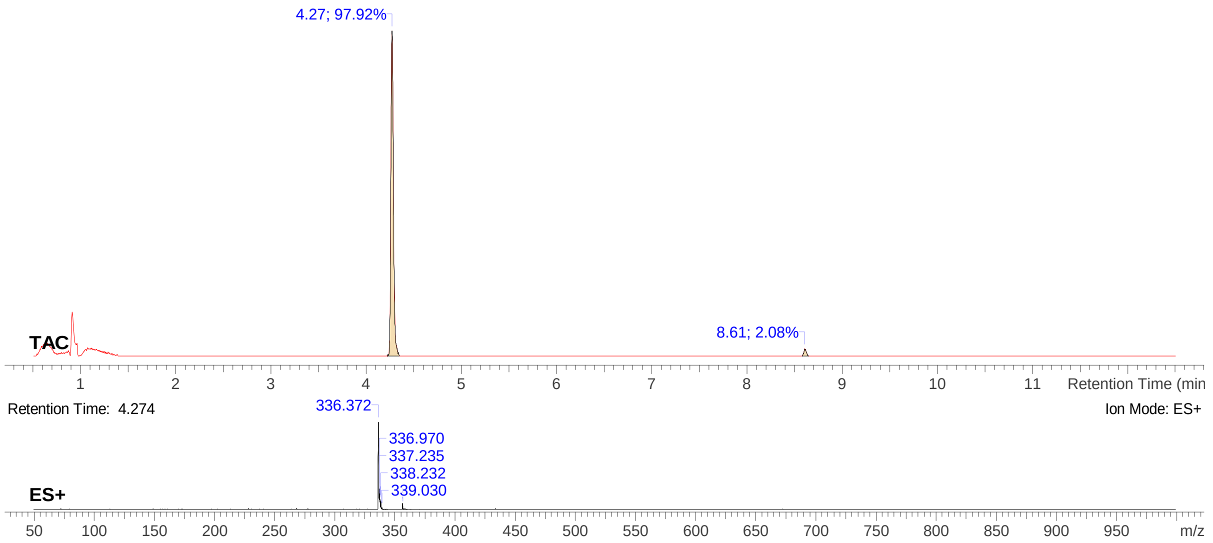
4.27; 97.92%
8.61; 2.08%
TAC
Retention Time (min
Retention Time: 4.274
Ion Mode: ES+
336.372
336.970
337.235
338.232
339.030
ES+
m/z

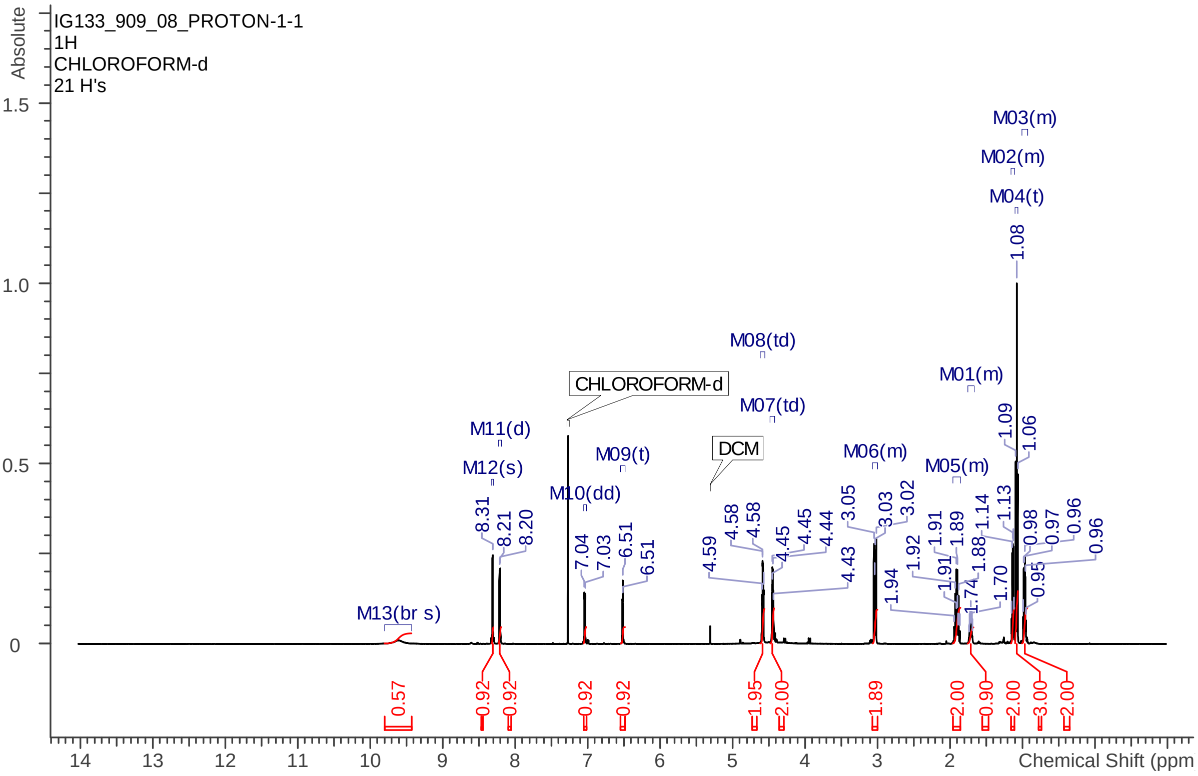
IG133_909_08_PROTON-1-1
1H
CHLOROFORM-d
21 H's
Absolute
CHLOROFORM-d
DCM
M01(m)
M02(m)
M03(m)
M04(t)
M05(m)
M06(m)
M07(td)
M08(td)
M09(t)
M10(dd)
M11(d)
M12(s)
M13(br s)
Chemical Shift (ppm

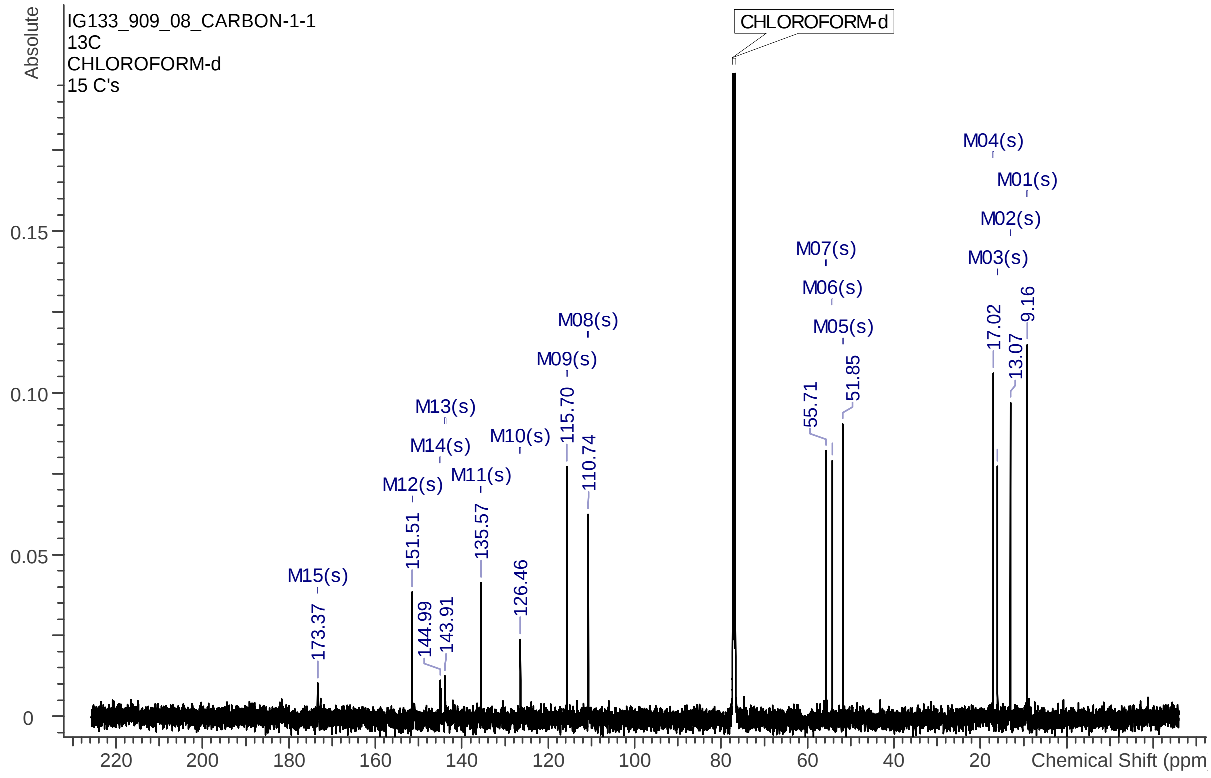

N-(4-(1-(Cyclopentylsulfonyl)-2,5-dihydro-1*H*-pyrrol-3-yl)pyridin-2-yl)cyclopropanecarboxamide (**18**)

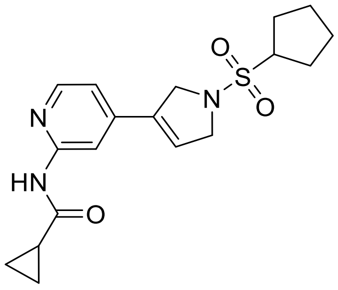

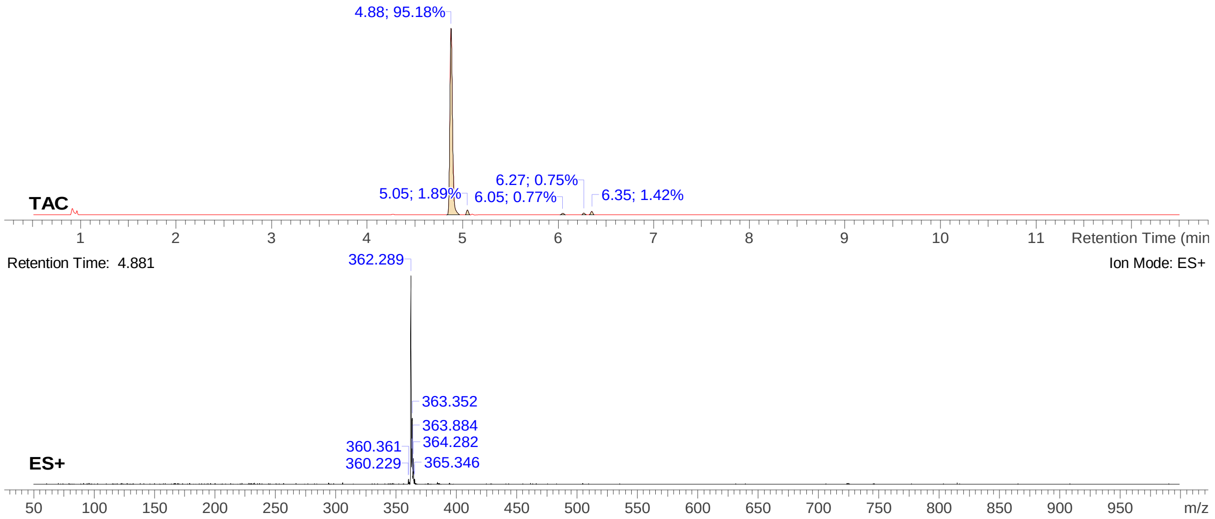

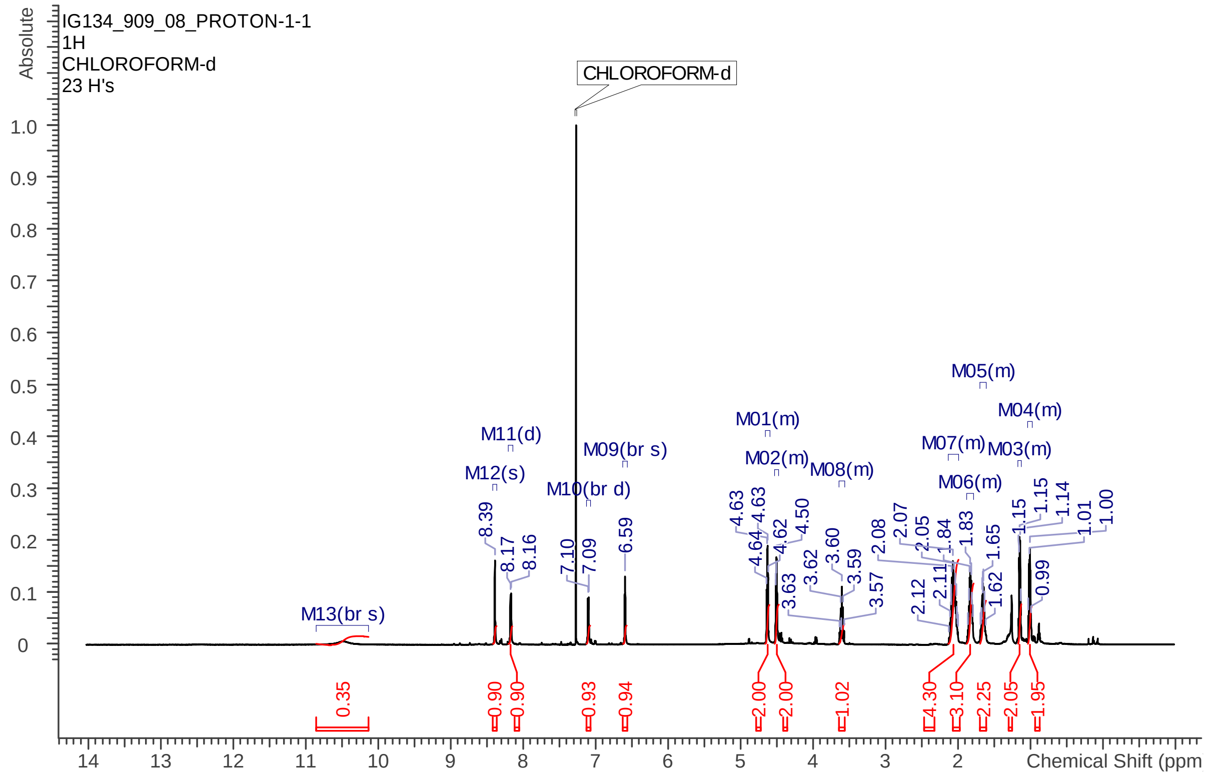
IG134_909_08_PROTON-1-1
1H
CHLOROFORM-d
23 H's
CHLOROFORM-d
M13(br s)
M12(s)
M11(d)
M10(br d)
M09(br s)
M01(m)
M02(m)
M08(m)
M07(m)
M06(m)
M05(m)
M04(m)
M03(m)
Chemical Shift (ppm)

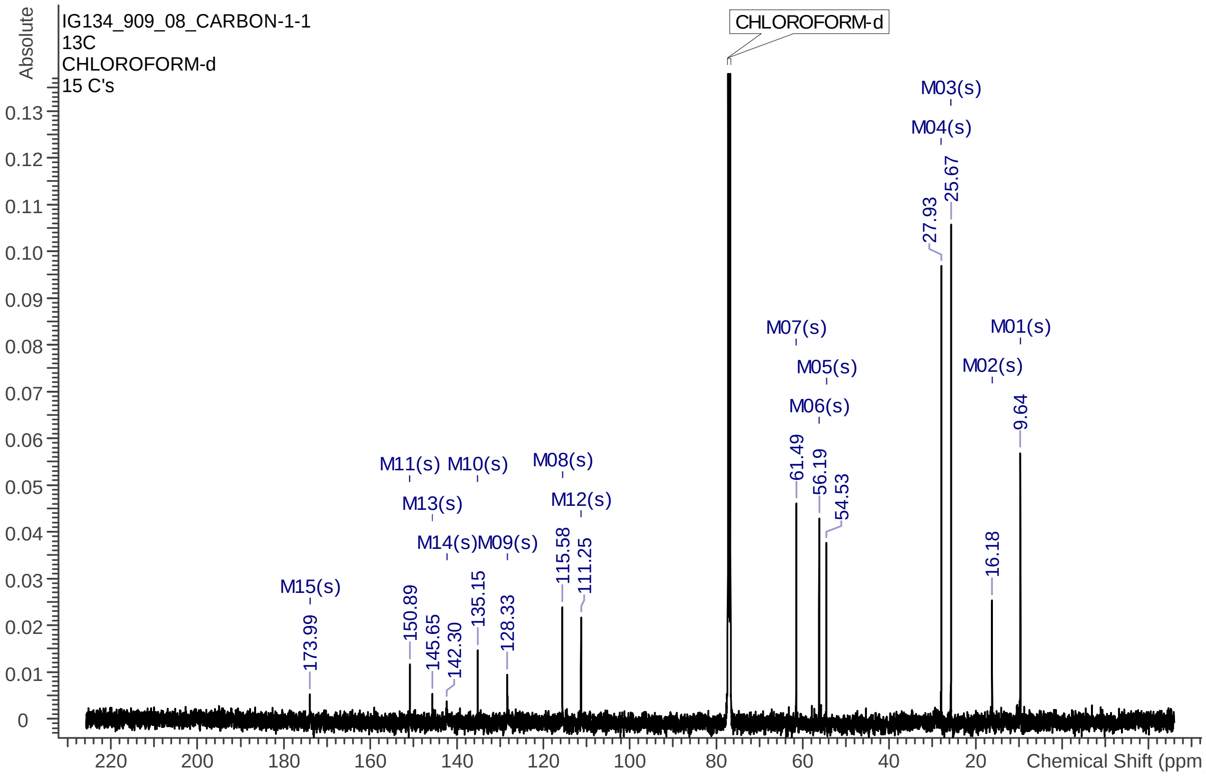
IG134_909_08_CARBON-1-1
13C
CHLOROFORM-d
15 C's
CHLOROFORM-d
M15(s)
M11(s) M10(s)
M13(s)
M14(s)M09(s)
M08(s)
M12(s)
M07(s)
M05(s)
M06(s)
M03(s)
M04(s)
M01(s)
M02(s)
173.99
150.89
145.65
142.30
135.15
128.33
115.58
111.25
61.49
56.19
54.53
27.93
25.67
16.18
9.64
Chemical Shift (ppm)

N-(4-(1-((2-Aminoethyl)sulfonyl)-2,5-dihydro-1*H*-pyrrol-3-yl)pyridin-2-yl)cyclopropanecarboxamide hydrochloride (**19**)

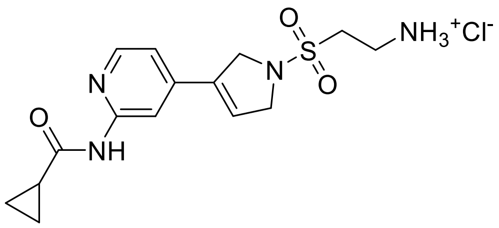

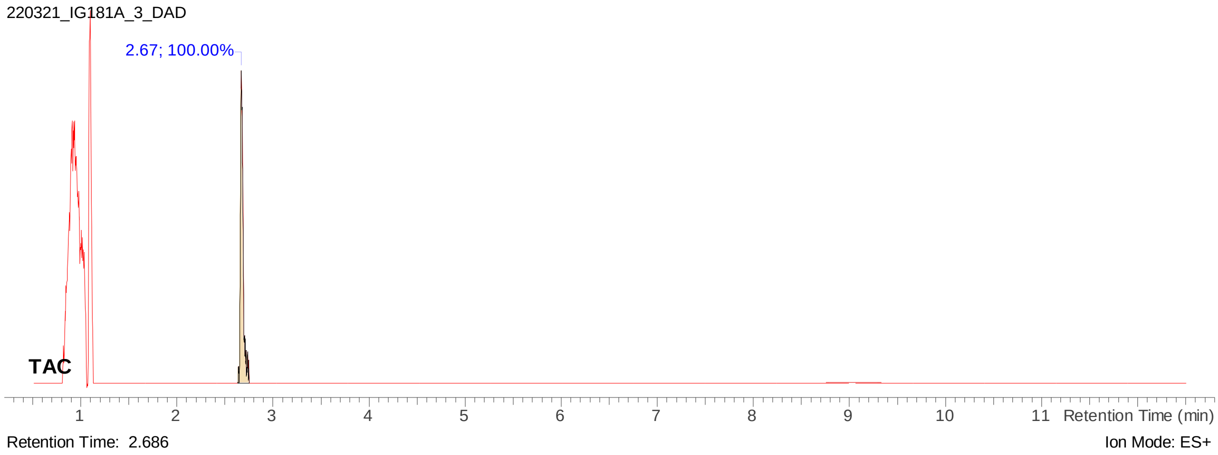

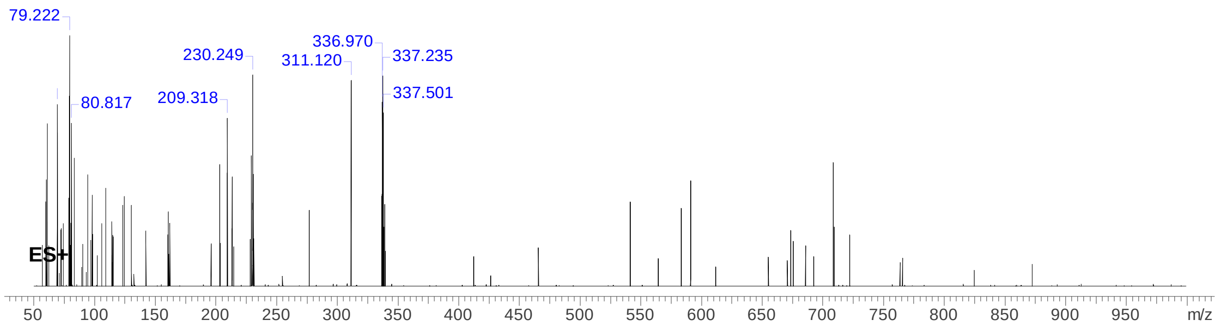

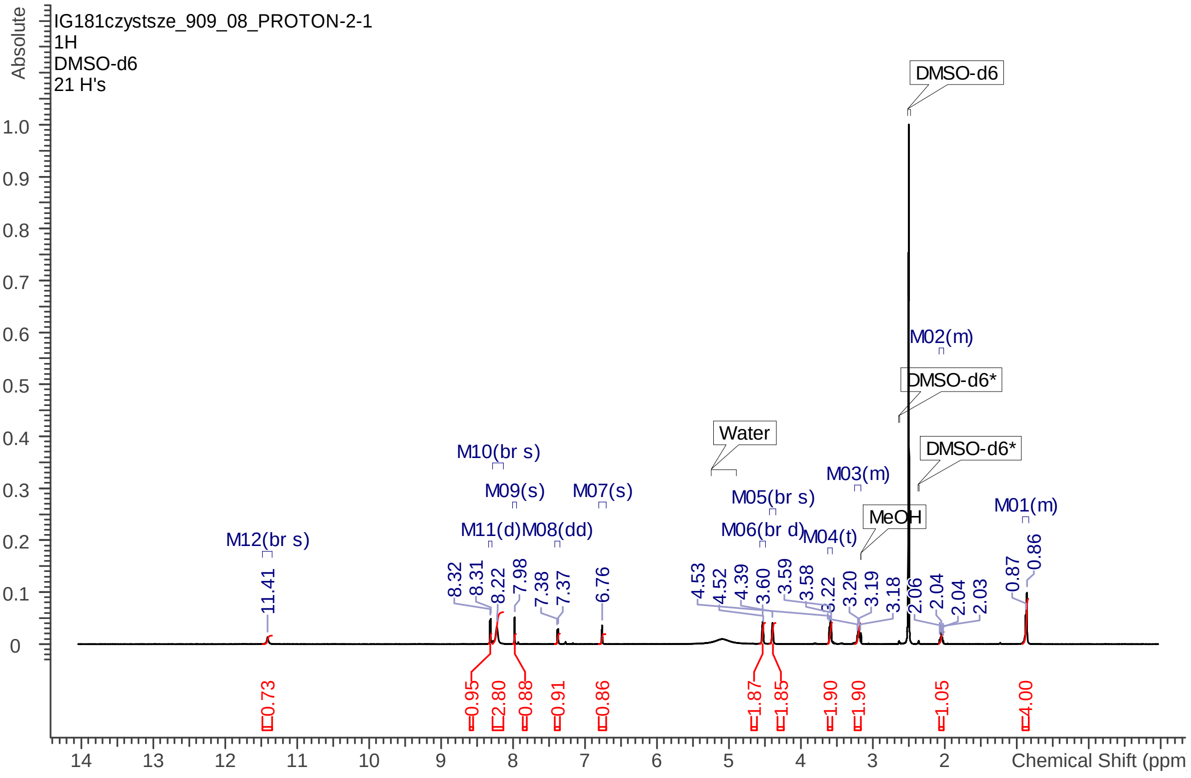
IG181czystsze_909_08_PROTON-2-1
1H
DMSO-d6
21 H's
Absolute
DMSO-d6
M02(m)
DMSO-d6*
DMSO-d6*
Water
M03(m)
M10(br s)
M09(s)
M07(s)
M05(br s)
MeOH
M01(m)
M12(br s)
M11(d)
M08(dd)
M06(br d)
M04(t)
Chemical Shift (ppm)

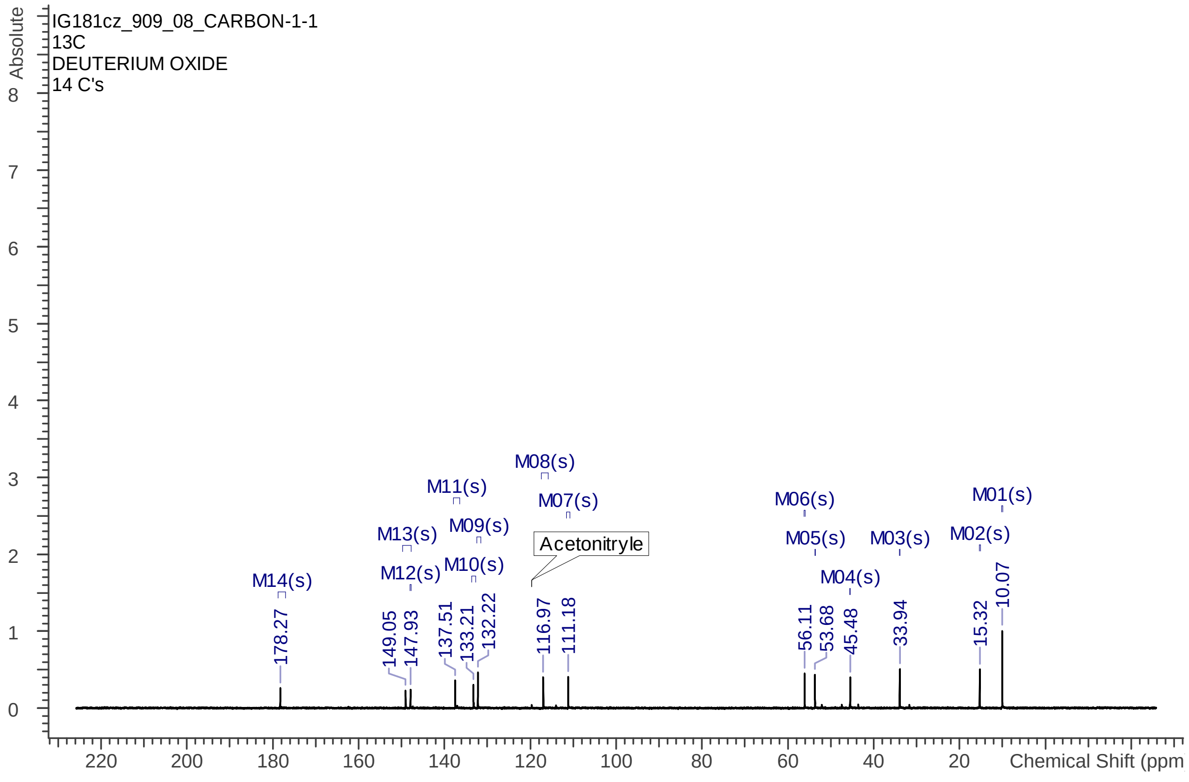
IG181cz_909_08_CARBON-1-1
13C
DEUTERIUM OXIDE
14 C's
Absolute
M08(s)
M11(s)
M07(s)
M06(s)
M01(s)
M13(s)
M09(s)
Acetonitryle
M05(s)
M03(s)
M02(s)
M12(s)
M10(s)
M14(s)
M04(s)
Chemical Shift (ppm)

N-(4-(1-((3-Aminopropyl)sulfonyl)-2,5-dihydro-1*H*-pyrrol-3-yl)pyridin-2-yl)cyclopropanecarboxamide hydrochloride (**20**)

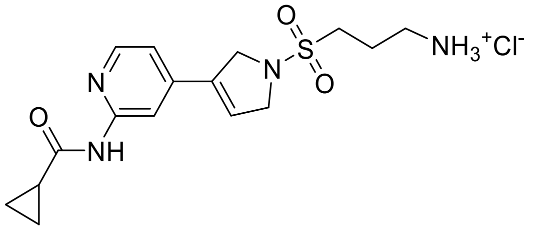

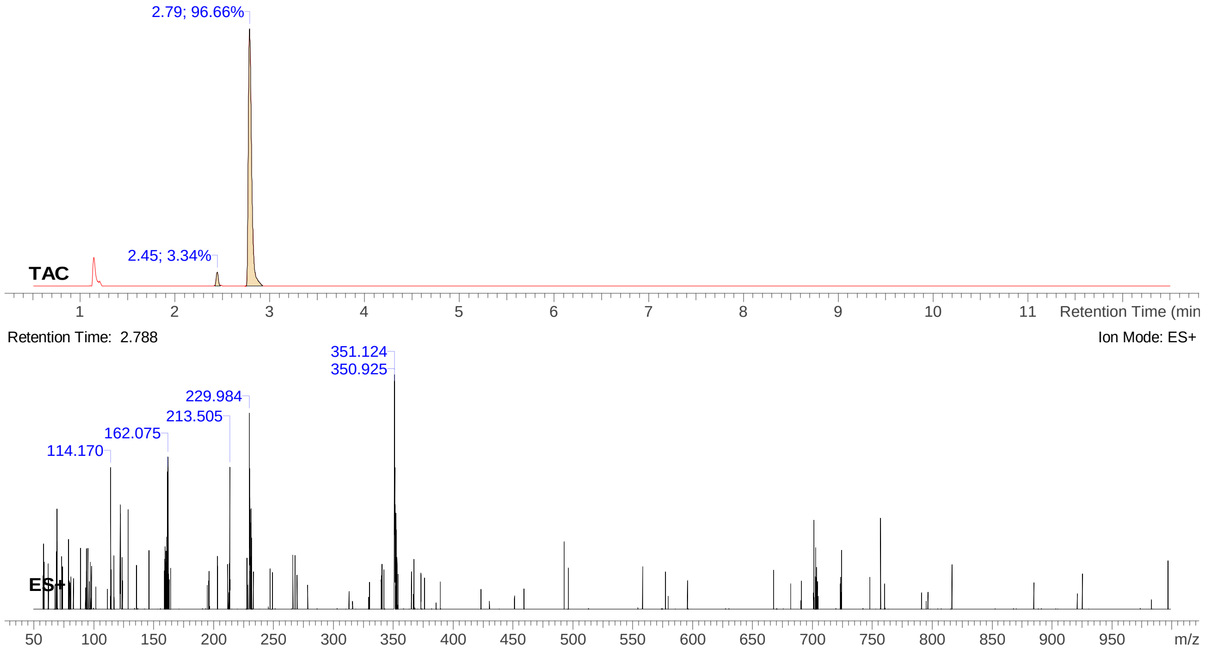

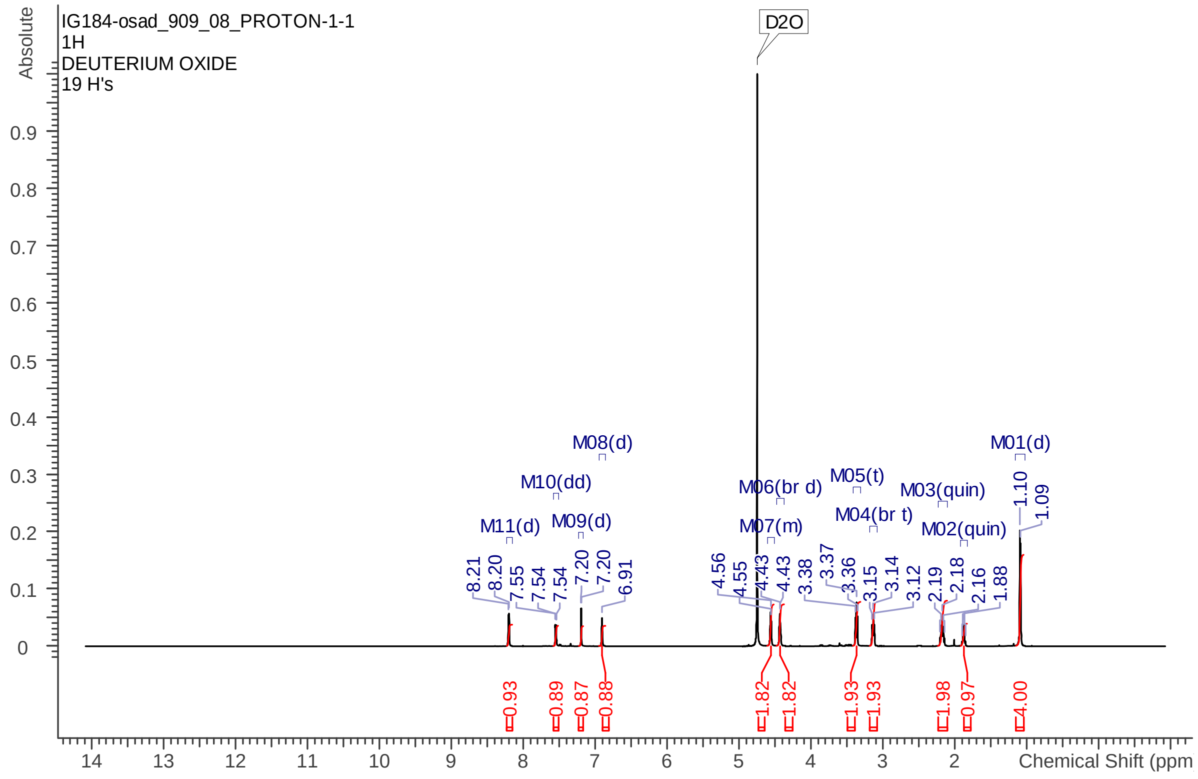

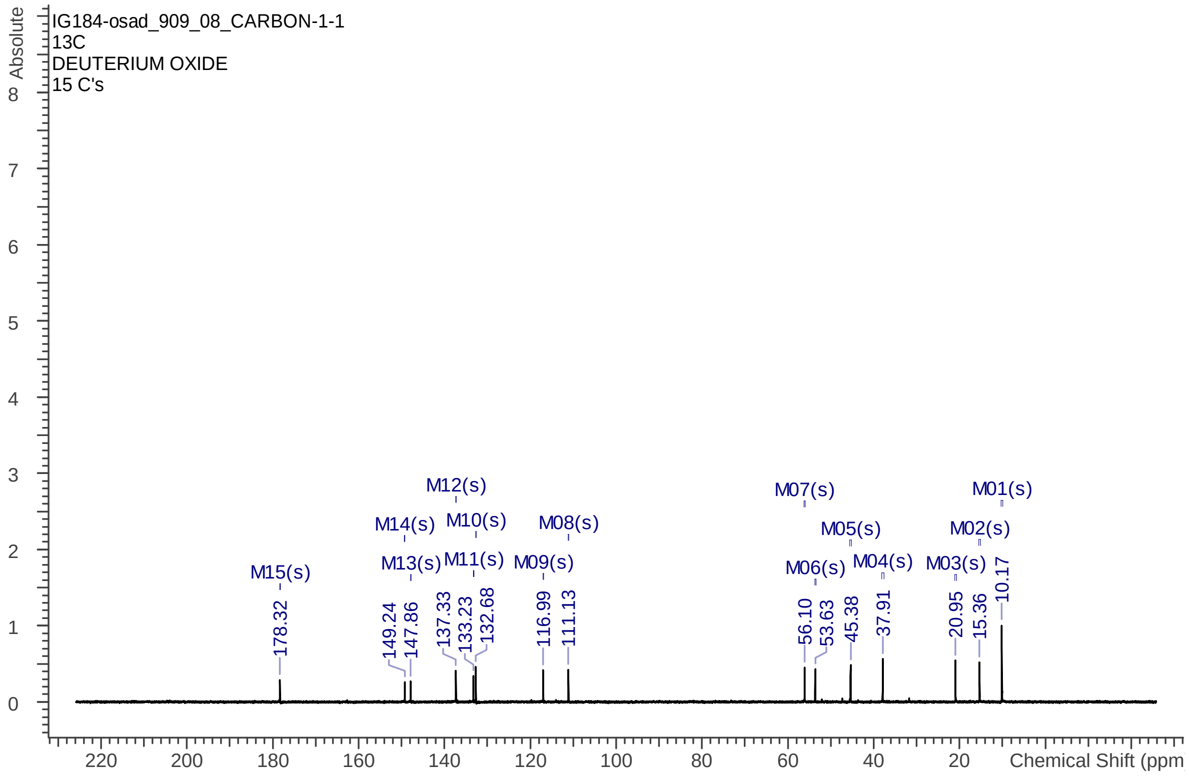

N-(4-(4-Cyanophenyl)pyridin-2-yl)cyclopropanecarboxamide (**22**)

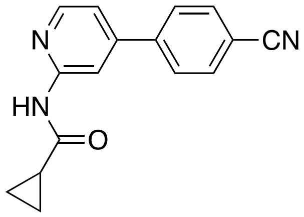

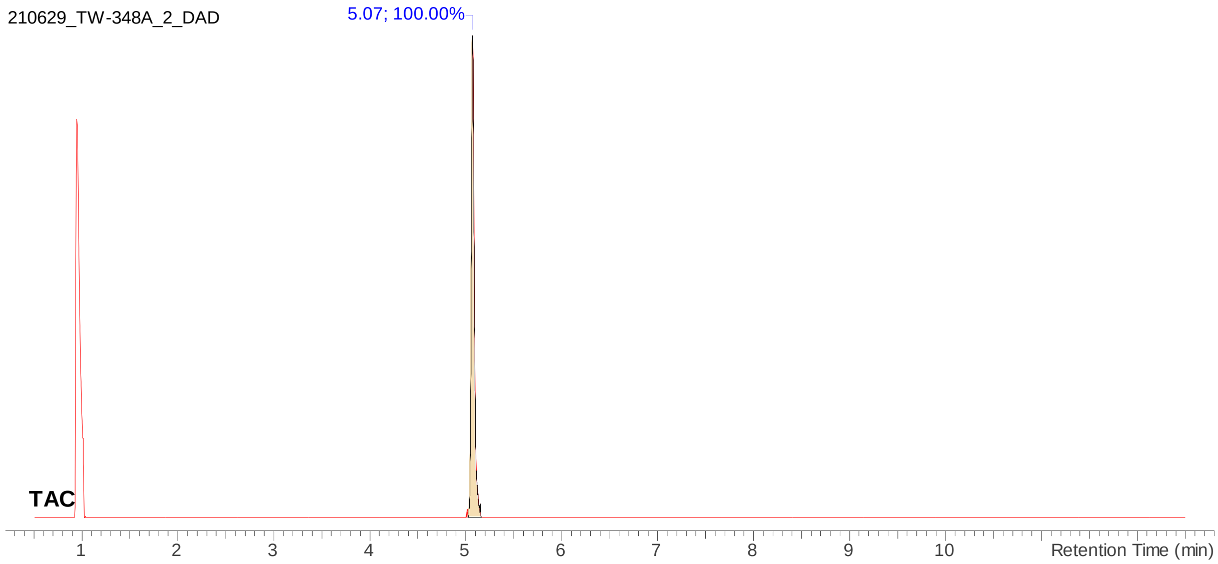
210629_TW-348A_2_DAD
5.07; 100.00%
TAC
Retention Time (min)

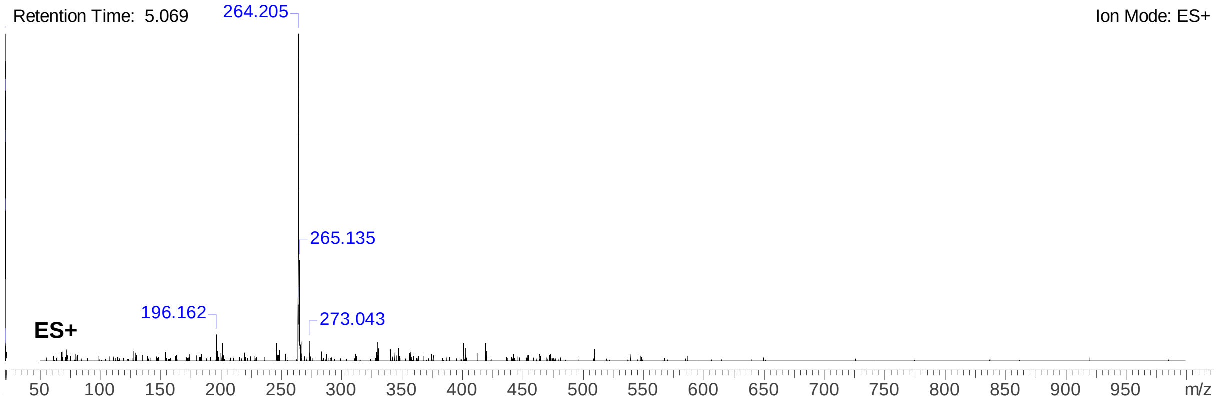
Retention Time: 5.069
264.205
Ion Mode: ES+
265.135
196.162
273.043
ES+
m/z

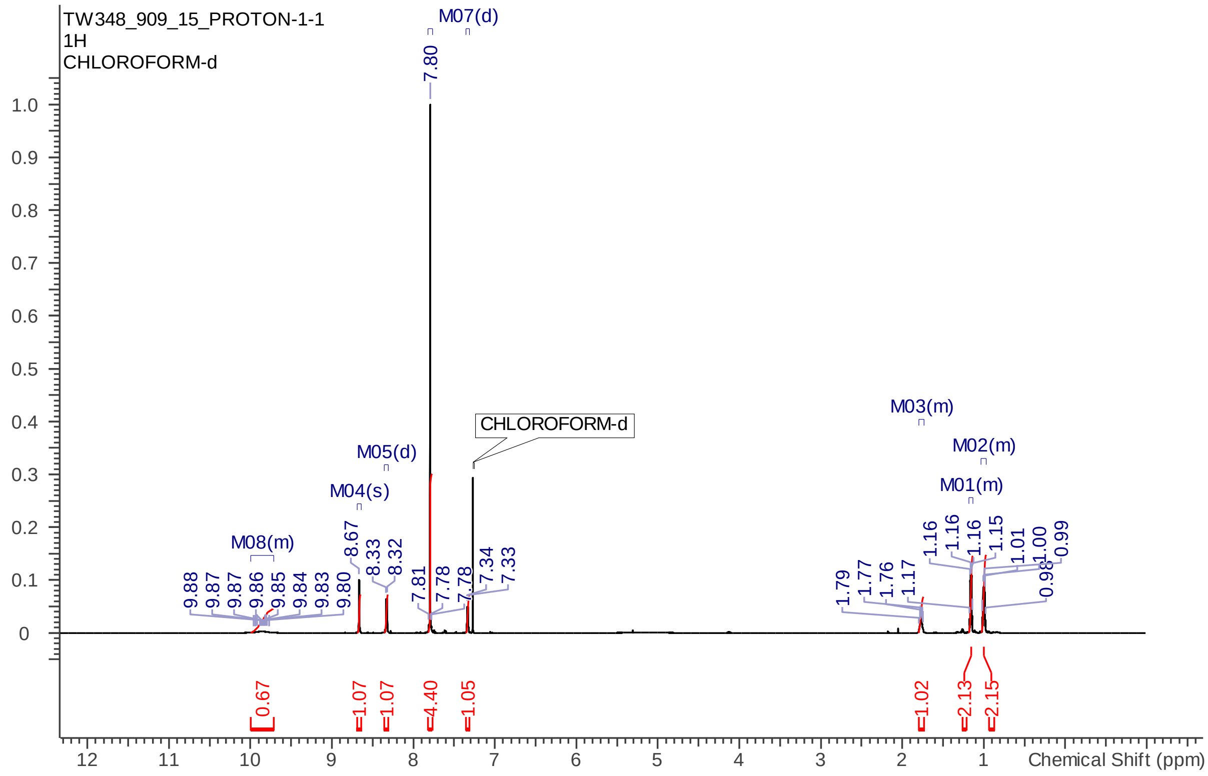
TW348_909_15_PROTON-1-1
1H
CHLOROFORM-d
M07(d)
7.80
CHLOROFORM-d
M03(m)
M02(m)
M01(m)
M05(d)
M04(s)
M08(m)
9.88 9.87 9.87 9.86 9.85 9.84 9.83 9.80
8.67 8.33 8.32
7.81 7.78 7.78 7.34 7.33
1.79 1.77 1.76 1.17 1.16 1.16 1.16 1.15 1.01 1.00 0.99 0.98
0.67 1.07 1.07 4.40 1.05 1.02 2.13 2.15
Chemical Shift (ppm)

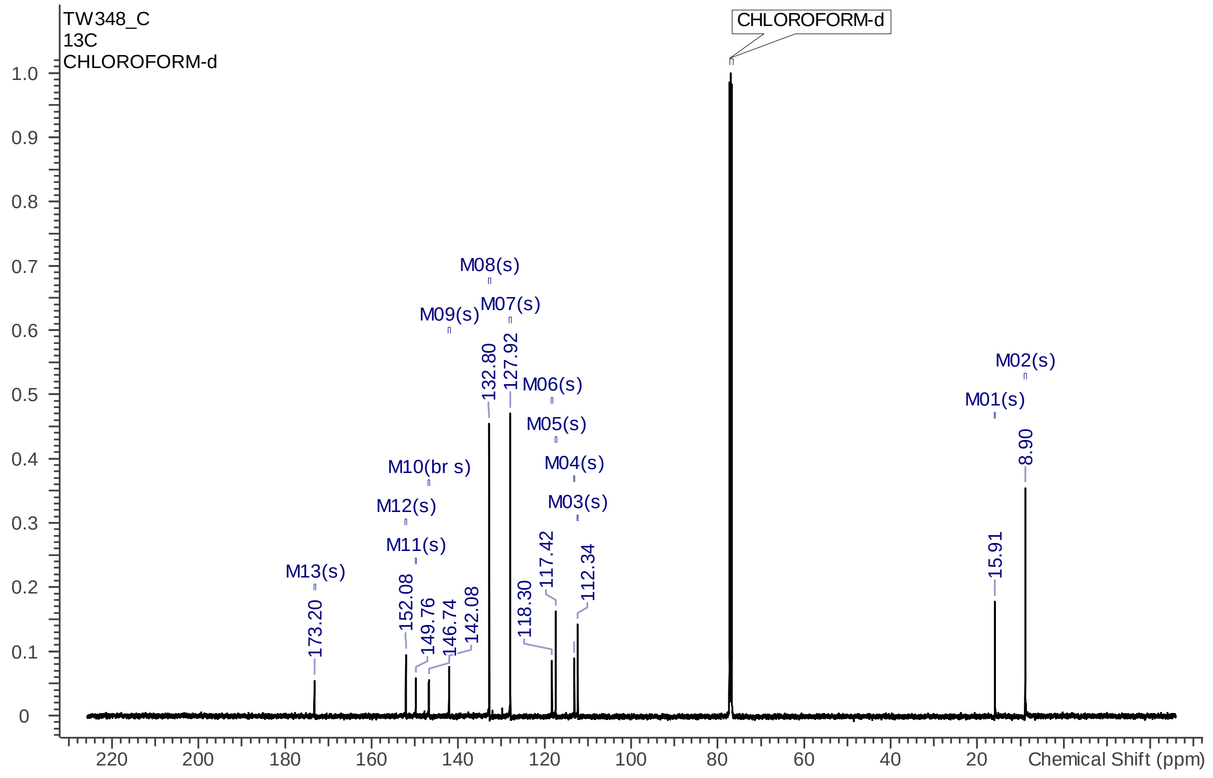

TW348_C
13C
CHLOROFORM-d
CHLOROFORM-d
M13(s)
173.20
M12(s)
152.08
M11(s)
149.76
M10(br s)
146.74
M09(s)
142.08
M08(s)
132.80
M07(s)
127.92
M06(s)
118.30
M05(s)
117.42
M04(s)
M03(s)
112.34
M01(s)
15.91
M02(s)
8.90
Chemical Shift (ppm)

N-(4-(3-Cyanophenyl)pyridin-2-yl)cyclopropanecarboxamide (**23**)

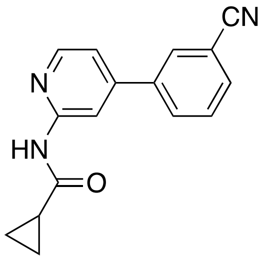

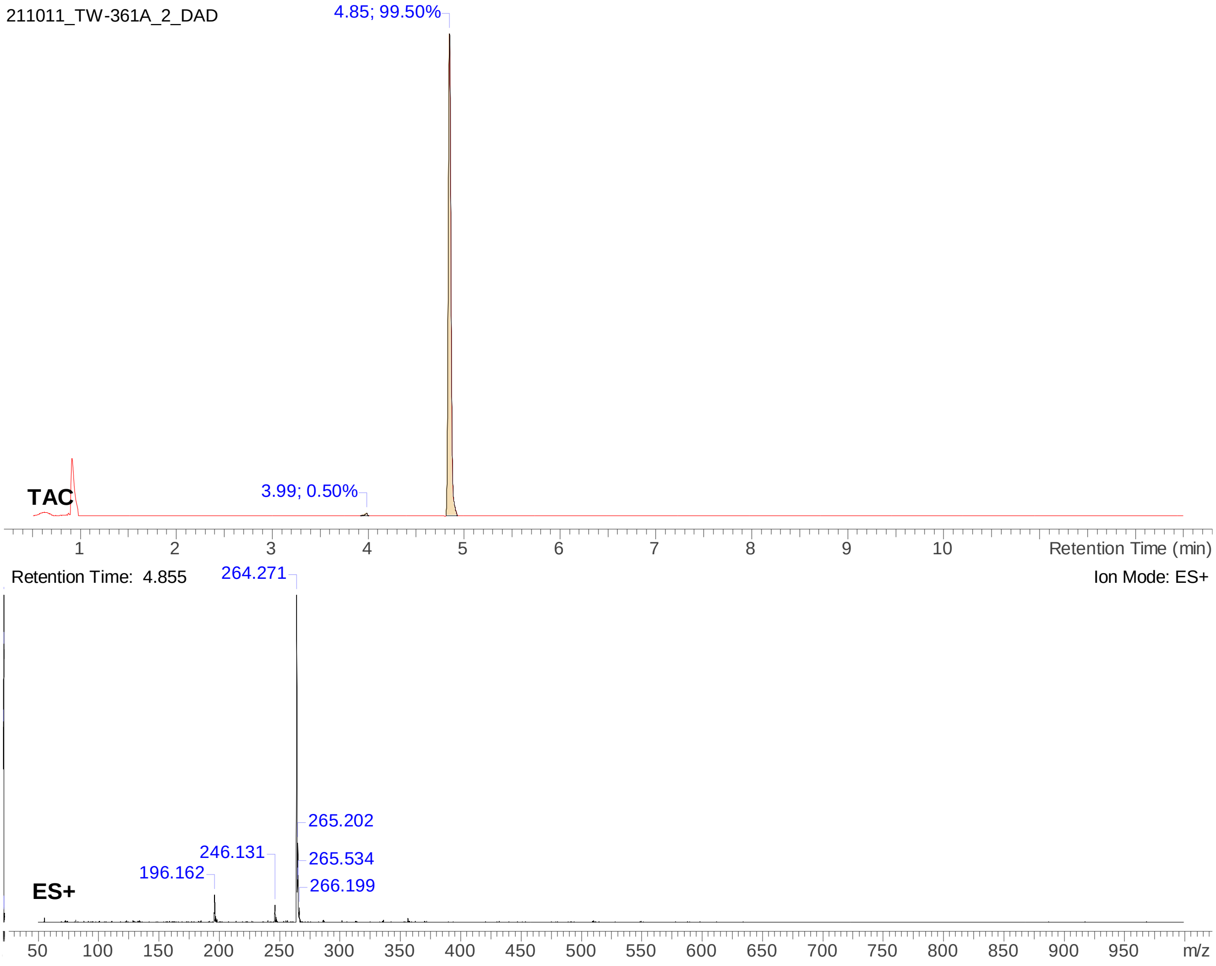

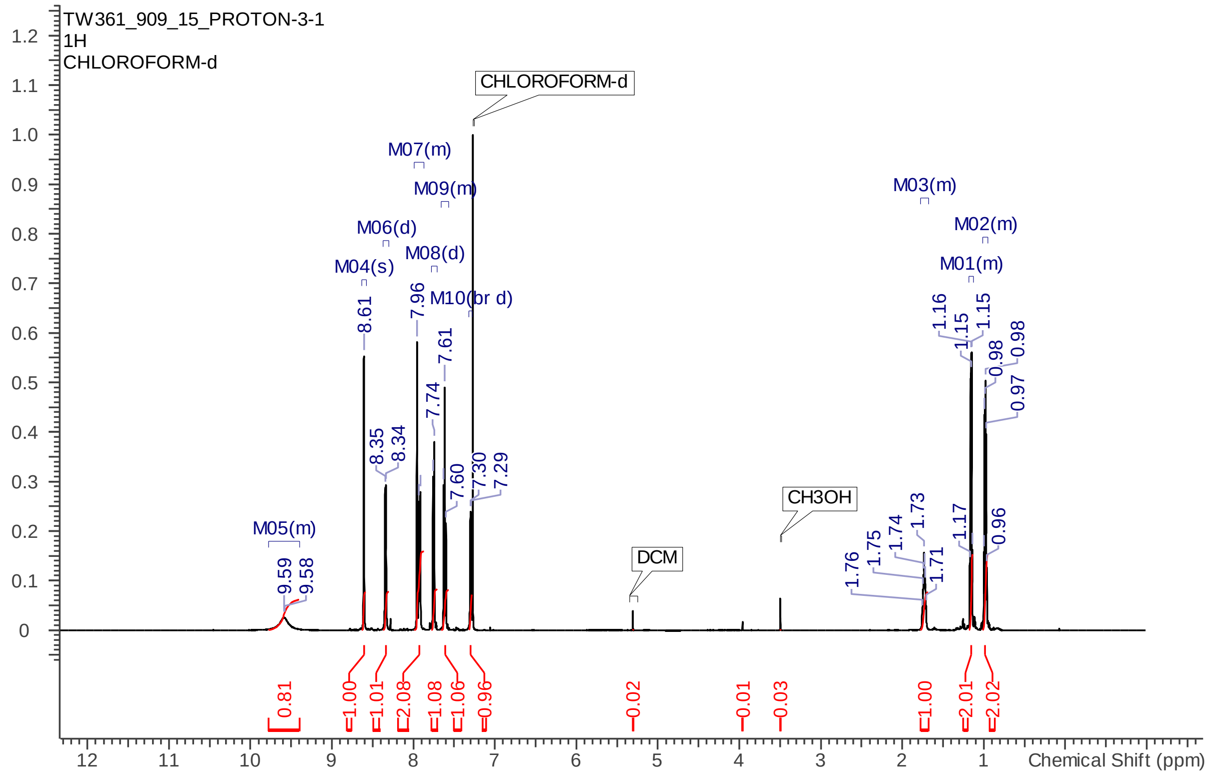
TW361_909_15_PROTON-3-1
1H
CHLOROFORM-d
CHLOROFORM-d
M07(m)
M09(m)
M06(d)
M08(d)
M04(s)
M10(br d)
M05(m)
M03(m)
M02(m)
M01(m)
DCM
CH3OH
Chemical Shift (ppm)

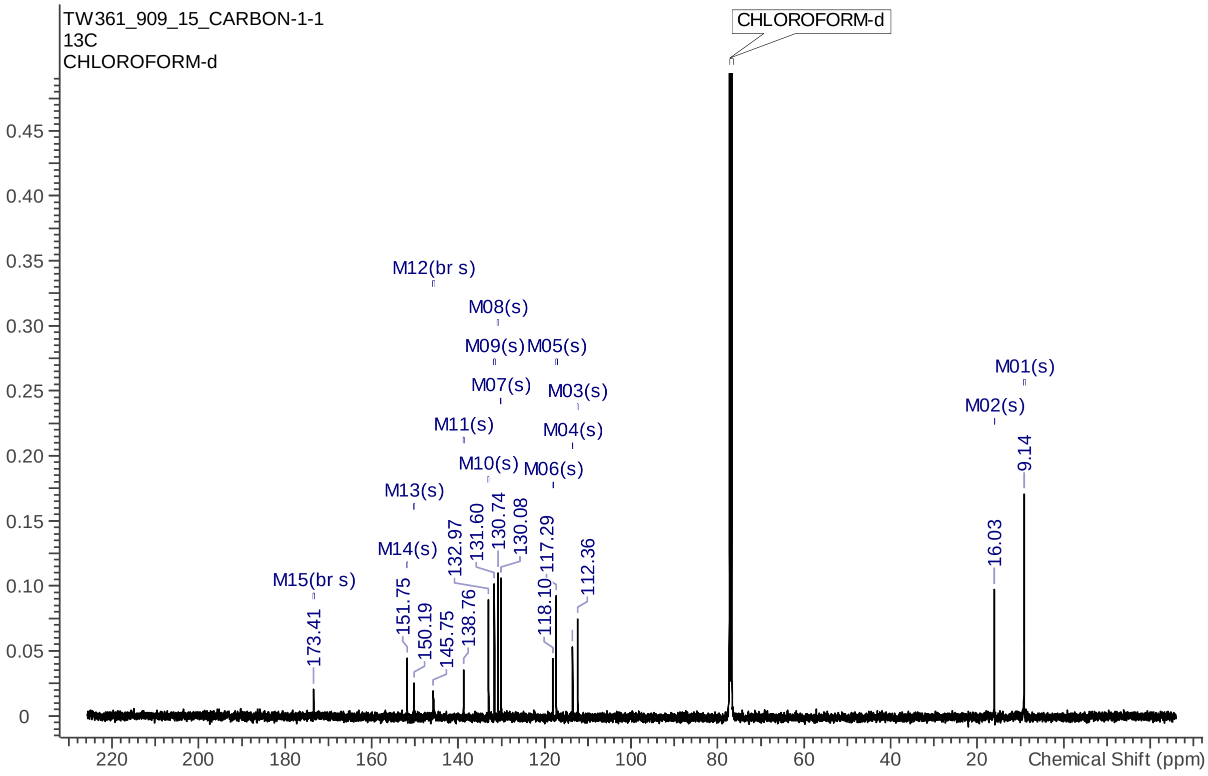
TW361_909_15_CARBON-1-1
13C
CHLOROFORM-d
CHLOROFORM-d
M12(br s)
M08(s)
M09(s) M05(s)
M07(s)
M03(s)
M11(s)
M04(s)
M10(s)
M06(s)
M13(s)
M14(s)
M15(br s)
M01(s)
M02(s)
Chemical Shift (ppm)

N-(6-Cyano-[3,4'-bipyridin]-2'-yl)cyclopropanecarboxamide (**24**)

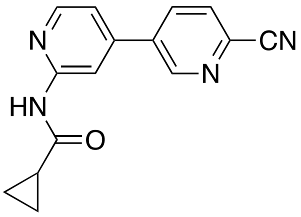

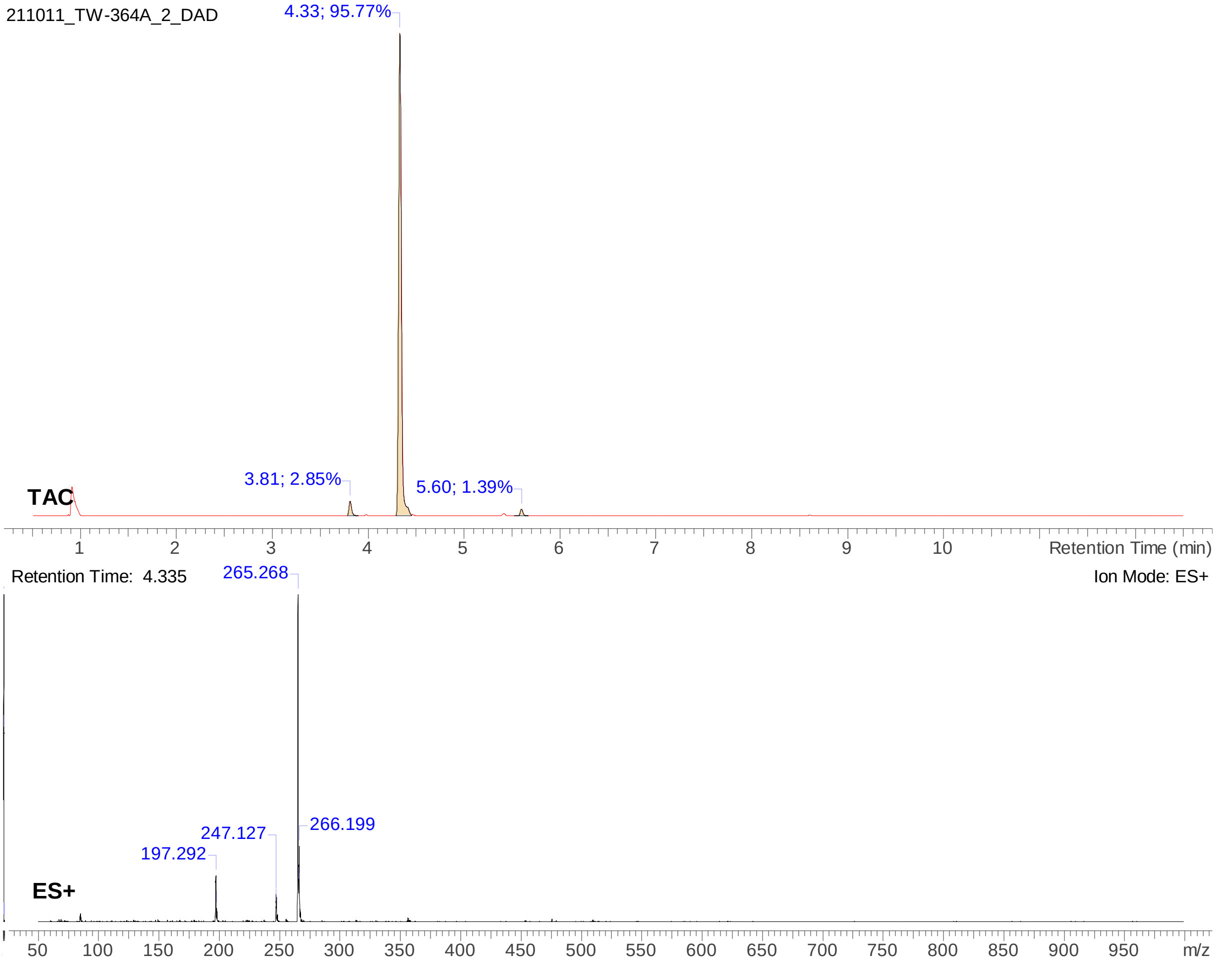

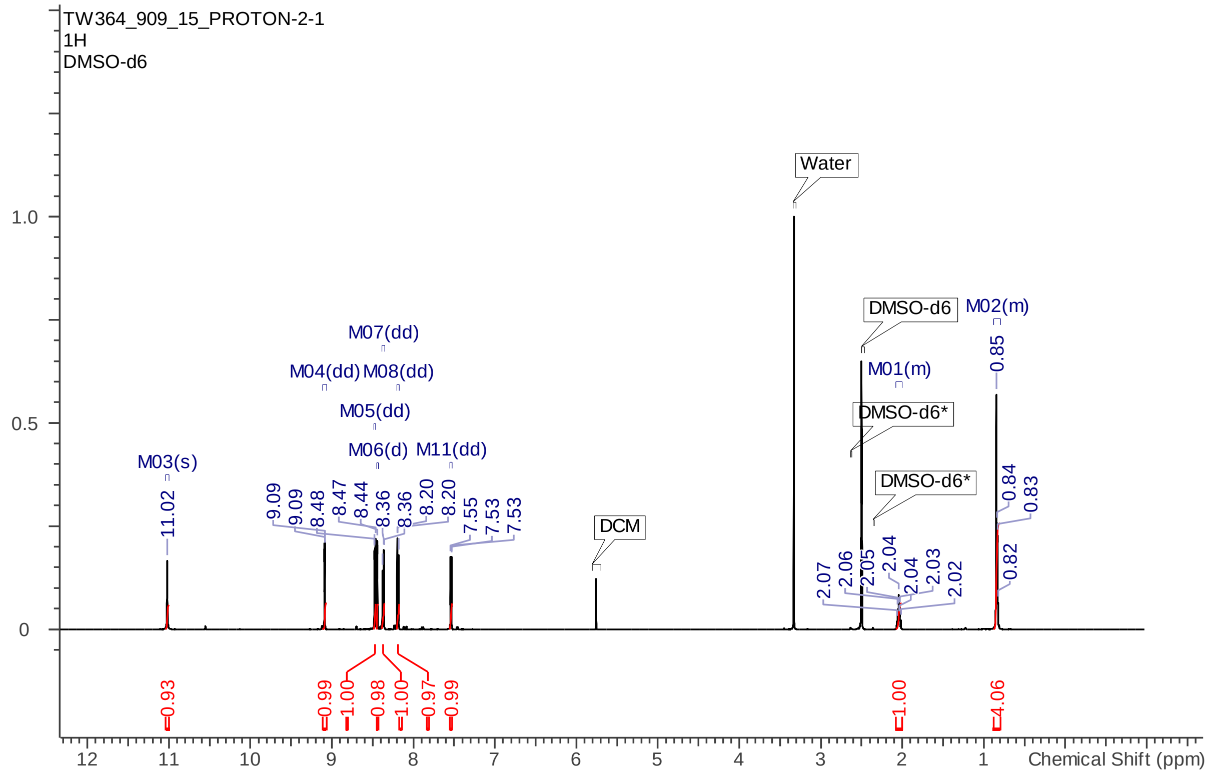
TW364_909_15_PROTON-2-1
1H
DMSO-d6
Water
DMSO-d6
M02(m)
M07(dd)
M04(dd)
M08(dd)
M01(m)
DMSO-d6*
M05(dd)
M06(d)
M11(dd)
M03(s)
DMSO-d6*
DCM
11.02
9.09
9.09
8.48
8.47
8.44
8.36
8.36
8.20
8.20
7.55
7.53
7.53
2.07
2.06
2.05
2.04
2.04
2.03
2.02
0.85
0.84
0.83
0.82
0.93
0.99
1.00
0.98
1.00
0.97
0.99
1.00
4.06
Chemical Shift (ppm)

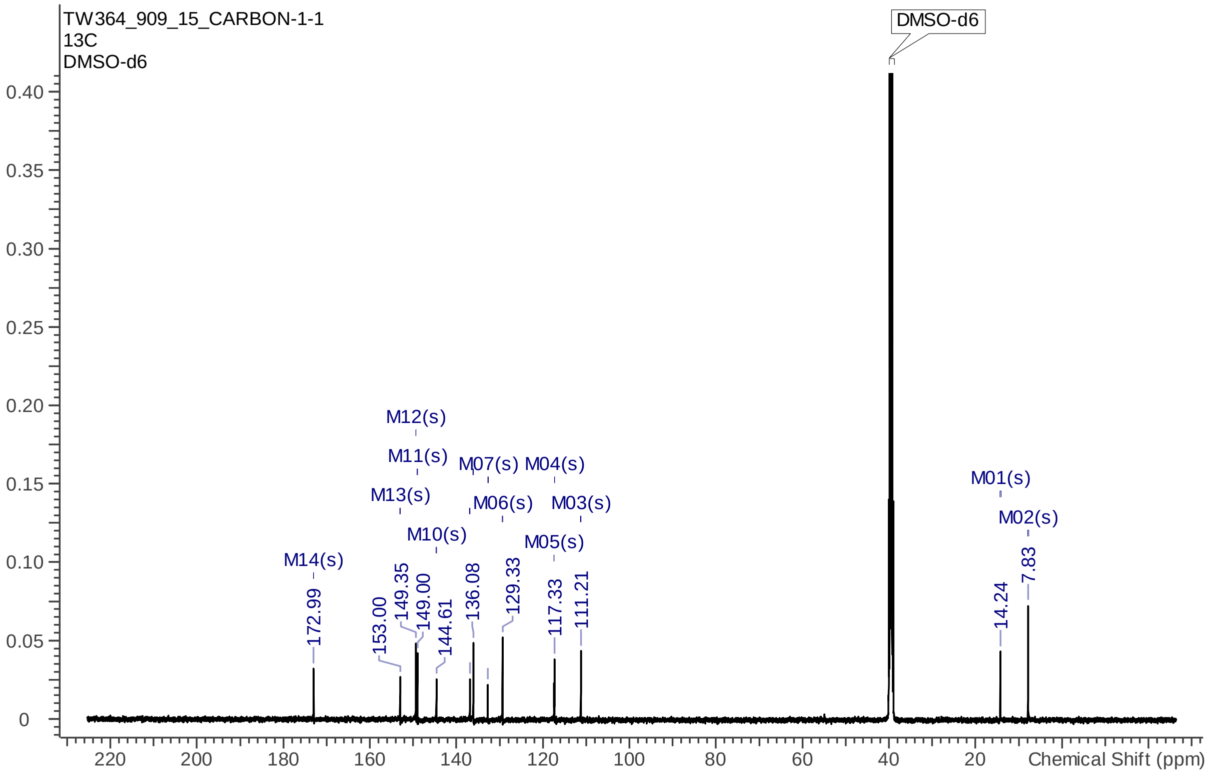
TW364_909_15_CARBON-1-1
13C
DMSO-d6
DMSO-d6
M12(s)
M11(s)
M07(s)
M04(s)
M13(s)
M06(s)
M03(s)
M01(s)
M10(s)
M02(s)
M05(s)
M14(s)
172.99
153.00
149.35
149.00
144.61
136.08
129.33
117.33
111.21
14.24
7.83
Chemical Shift (ppm)

N-(2'-Cyano-[4,4'-bipyridin]-2-yl)cyclopropanecarboxamide (**25**)

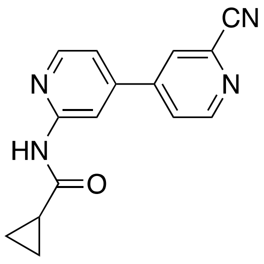

210915_TW369A_2_DAD

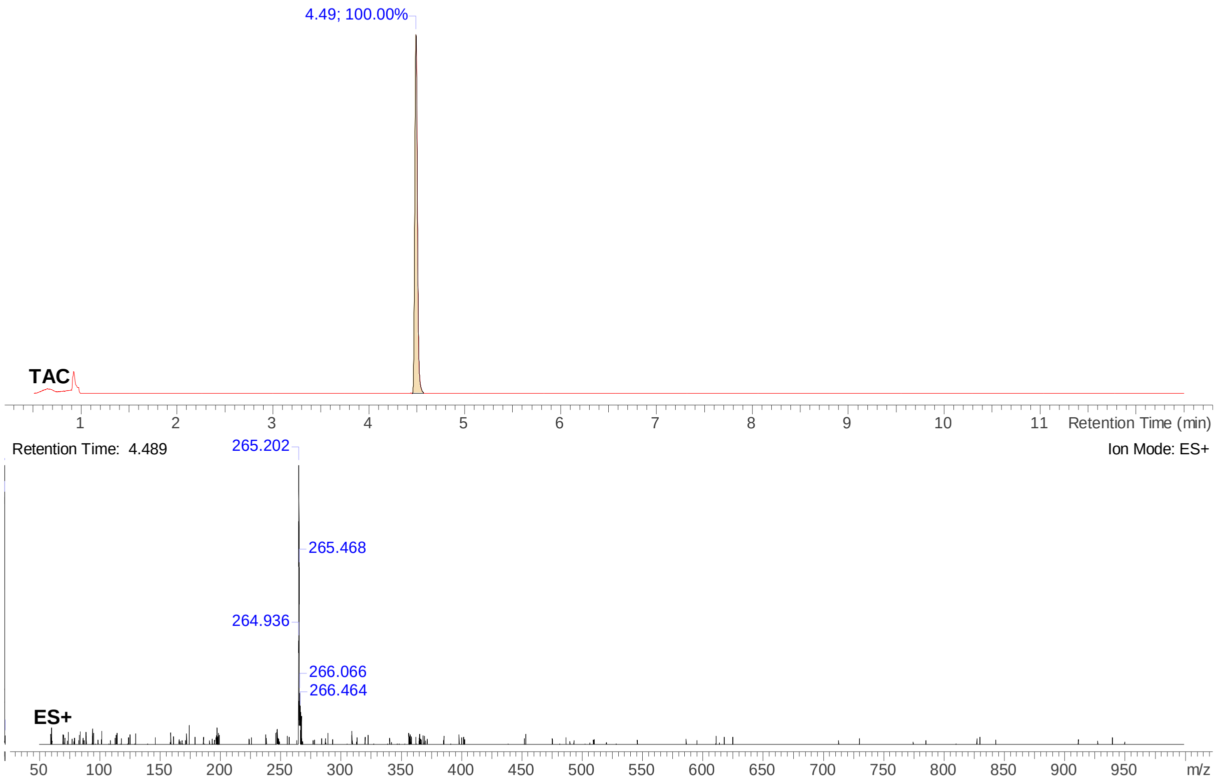

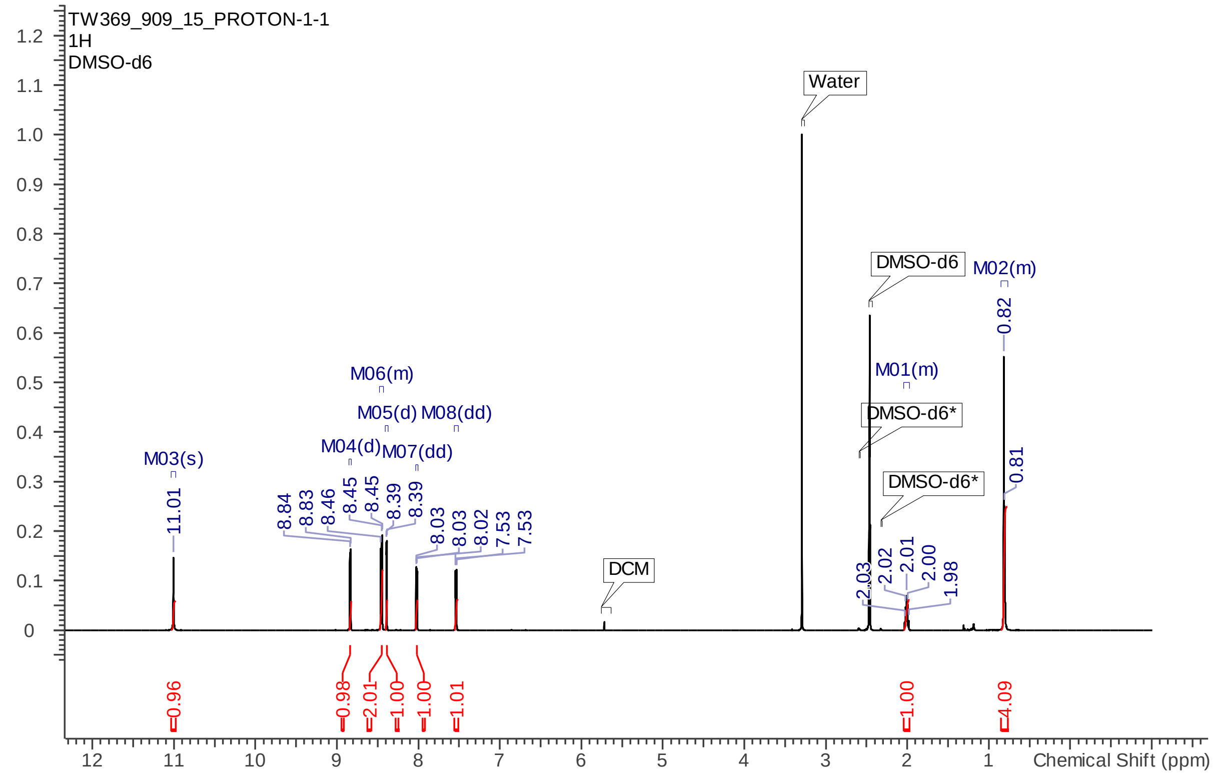
TW369_909_15_PROTON-1-1
1H
DMSO-d6
Water
DMSO-d6
M02(m)
M01(m)
DMSO-d6*
DMSO-d6*
M06(m)
M05(d) M08(dd)
M04(d) M07(dd)
M03(s)
DCM
11.01
8.84 8.83 8.46 8.45 8.45 8.39 8.39 8.03 8.03 8.02 7.53 7.53
2.03 2.02 2.01 2.00 1.98
0.82 0.81
0.96 0.98 2.01 1.00 1.00 1.01 1.00 4.09
Chemical Shift (ppm)

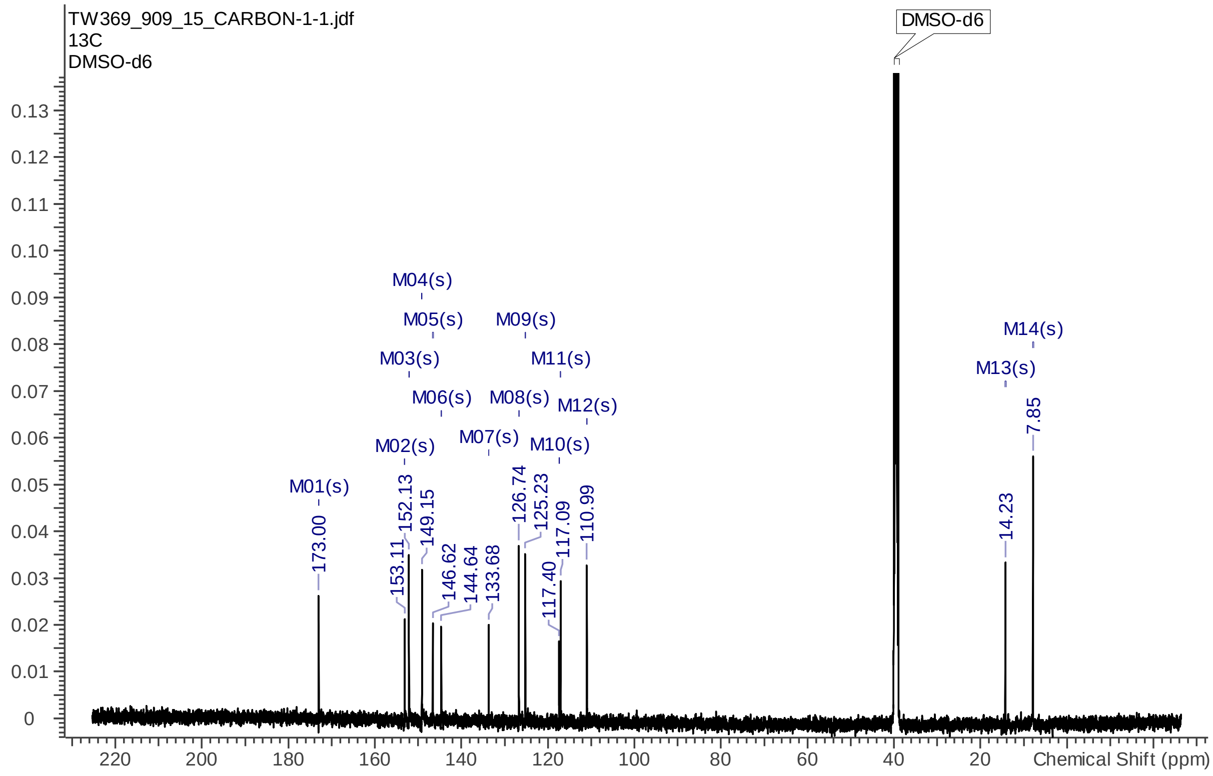
TW369_909_15_CARBON-1-1.jdf
13C
DMSO-d6
DMSO-d6
M01(s) M02(s) M03(s) M04(s) M05(s) M06(s) M07(s) M08(s) M09(s) M10(s) M11(s) M12(s) M13(s) M14(s)
173.00 153.11 152.13 149.15 146.62 144.64 133.68 126.74 125.23 117.40 117.09 110.99 14.23 7.85
Chemical Shift (ppm)

N-(4-(5-Cyanothiophen-2-yl)pyridin-2-yl)cyclopropanecarboxamide (**26**)

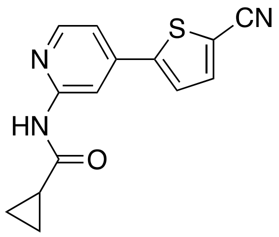

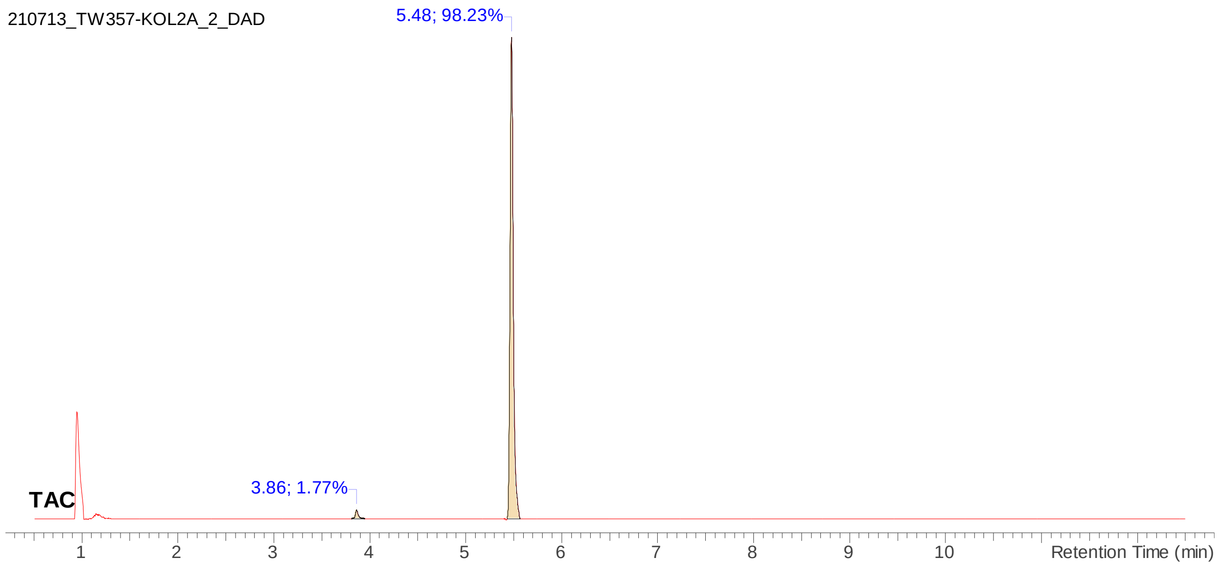

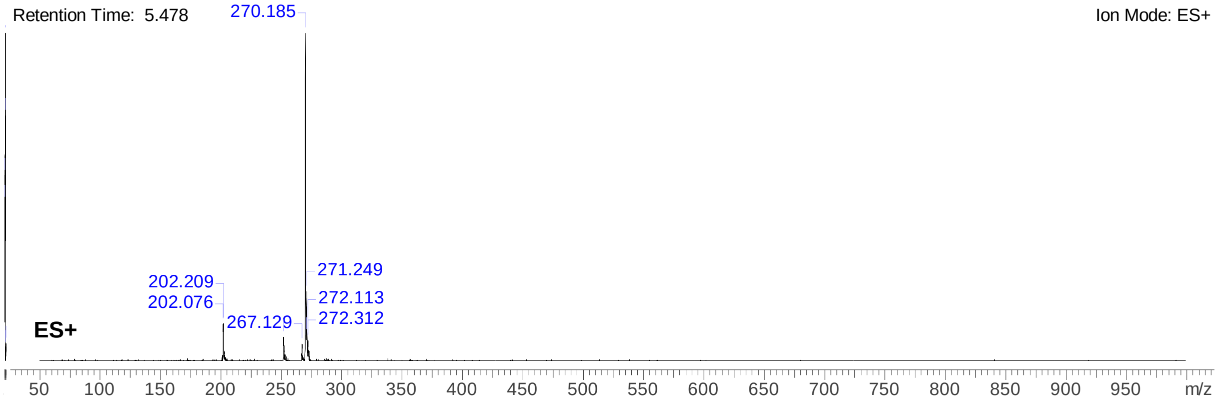

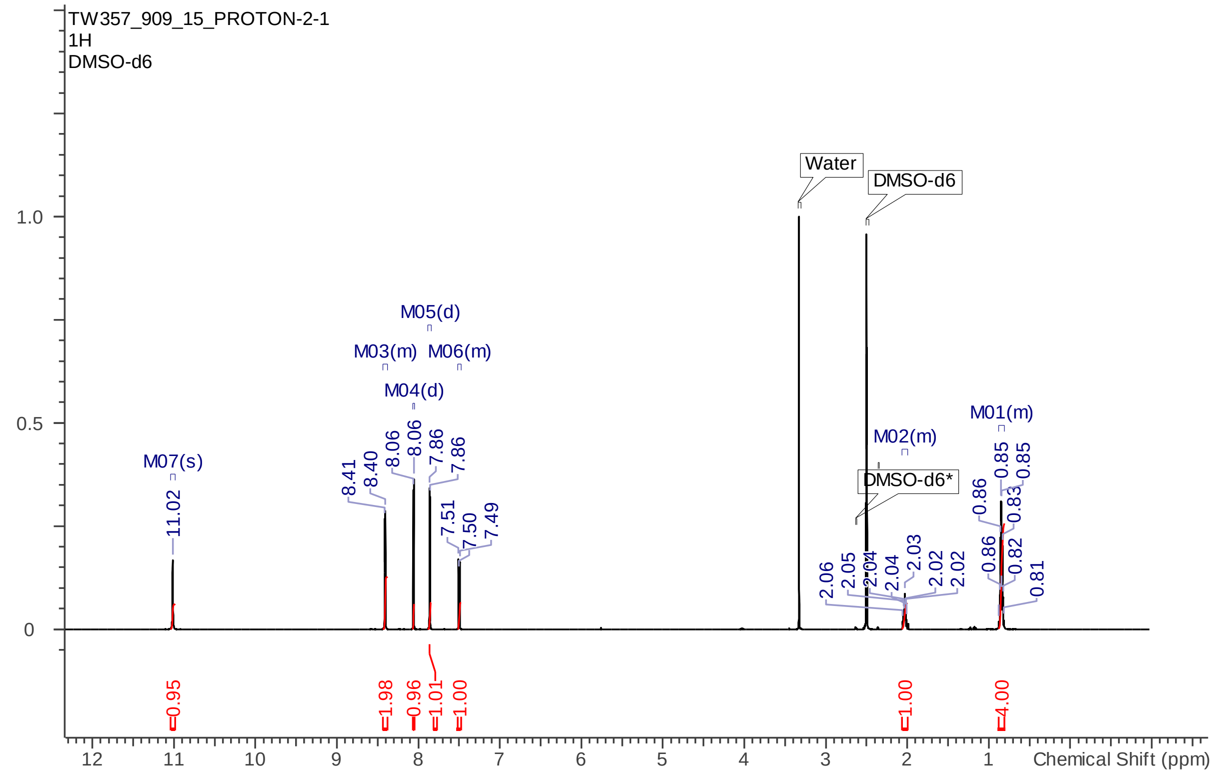
TW357_909_15_PROTON-2-1
1H
DMSO-d6
Water
DMSO-d6
DMSO-d6*
M07(s)
M03(m)
M04(d)
M05(d)
M06(m)
M02(m)
M01(m)
11.02
8.41
8.40
8.06
8.06
7.86
7.86
7.51
7.50
7.49
2.06
2.05
2.04
2.04
2.03
2.02
2.02
0.86
0.86
0.85
0.85
0.83
0.82
0.81
0.95
1.98
0.96
1.01
1.00
1.00
4.00
Chemical Shift (ppm)

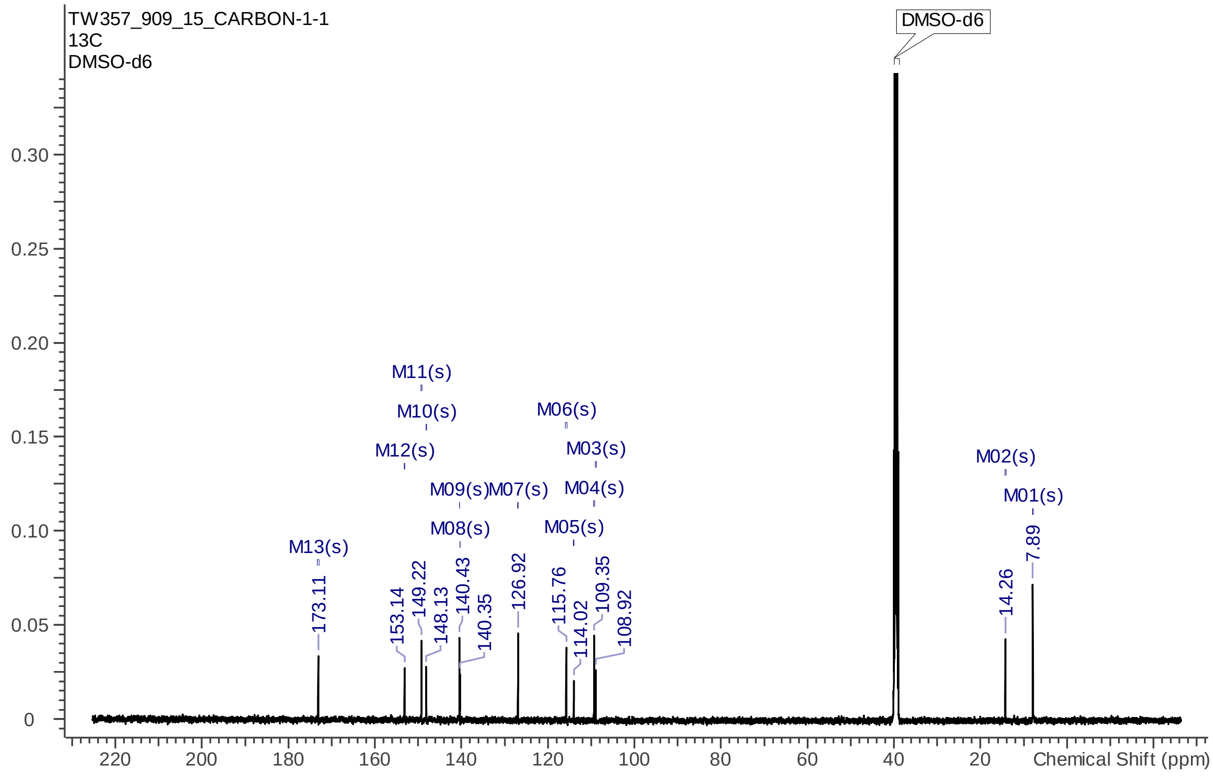
TW357_909_15_CARBON-1-1
13C
DMSO-d6
DMSO-d6
M13(s)
M12(s)
M11(s)
M10(s)
M09(s)
M08(s)
M07(s)
M06(s)
M03(s)
M04(s)
M05(s)
M02(s)
M01(s)
173.11
153.14
149.22
148.13
140.43
140.35
126.92
115.76
114.02
109.35
108.92
14.26
7.89
Chemical Shift (ppm)

N-(4-(4-(1,2,4-Oxadiazol-3-yl)phenyl)pyridin-2-yl)cyclopropanecarboxamide (**32**)

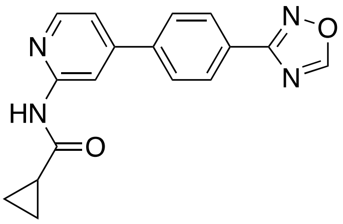

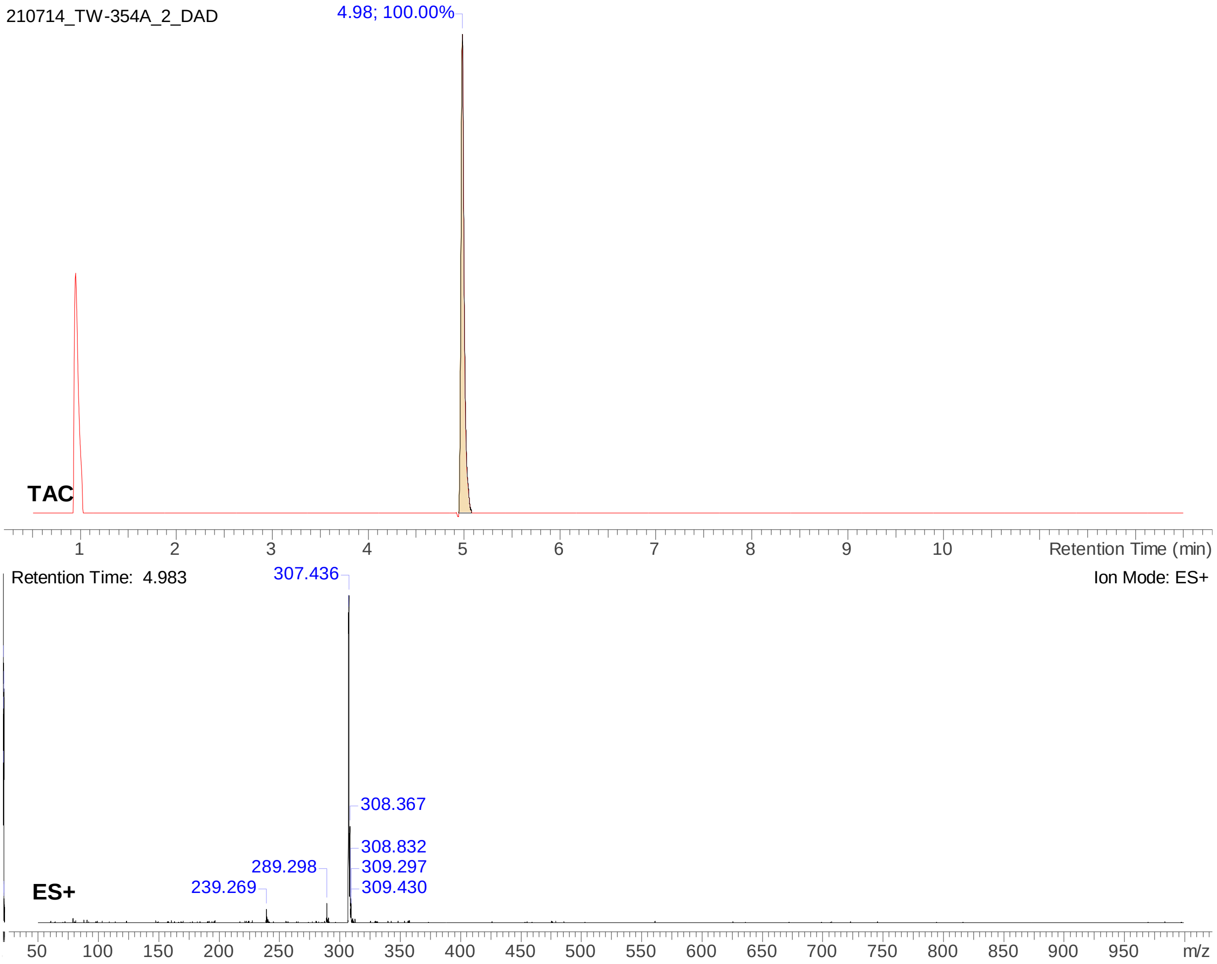

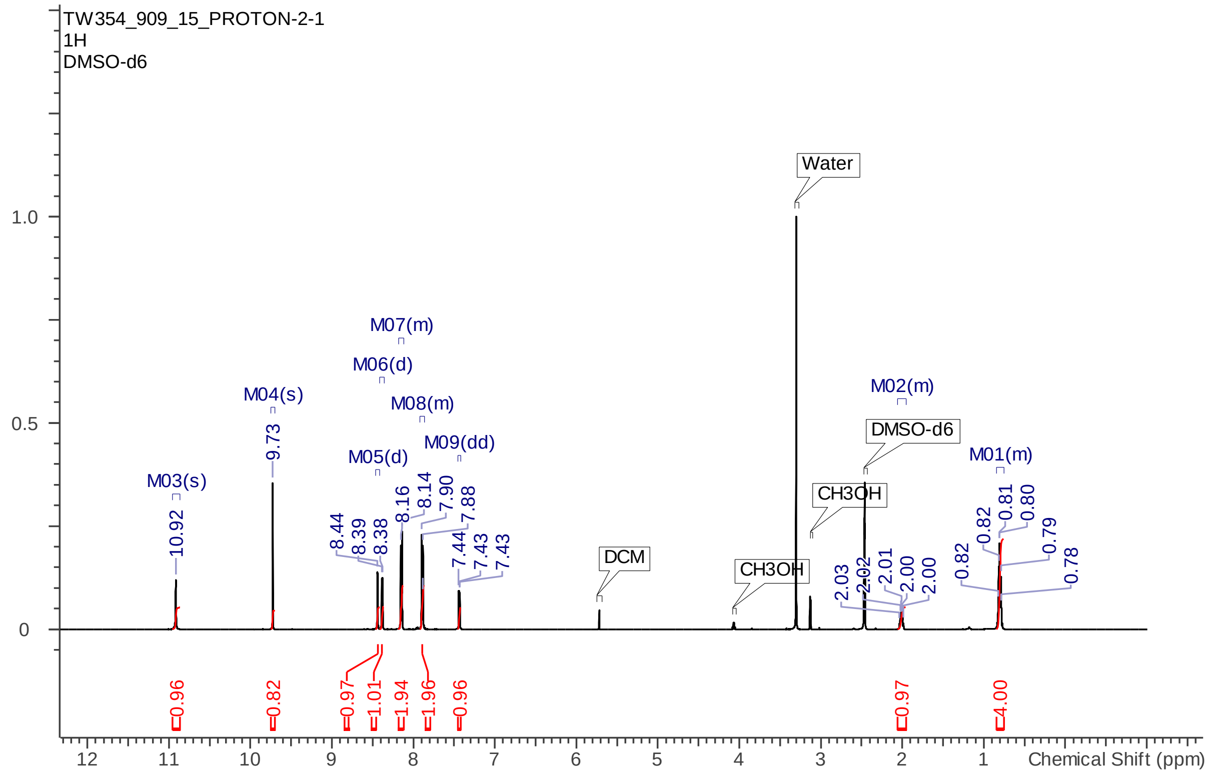
TW354_909_15_PROTON-2-1
1H
DMSO-d6
Water
DMSO-d6
CH3OH
CH3OH
DCM
M01(m)
M02(m)
M03(s)
M04(s)
M05(d)
M06(d)
M07(m)
M08(m)
M09(dd)
10.92
9.73
8.44
8.39
8.38
8.16
8.14
7.90
7.88
7.44
7.43
7.43
2.03
2.02
2.01
2.00
2.00
0.82
0.82
0.81
0.80
0.79
0.78
0.96
0.82
0.97
1.01
1.94
1.96
0.96
0.97
4.00
Chemical Shift (ppm)

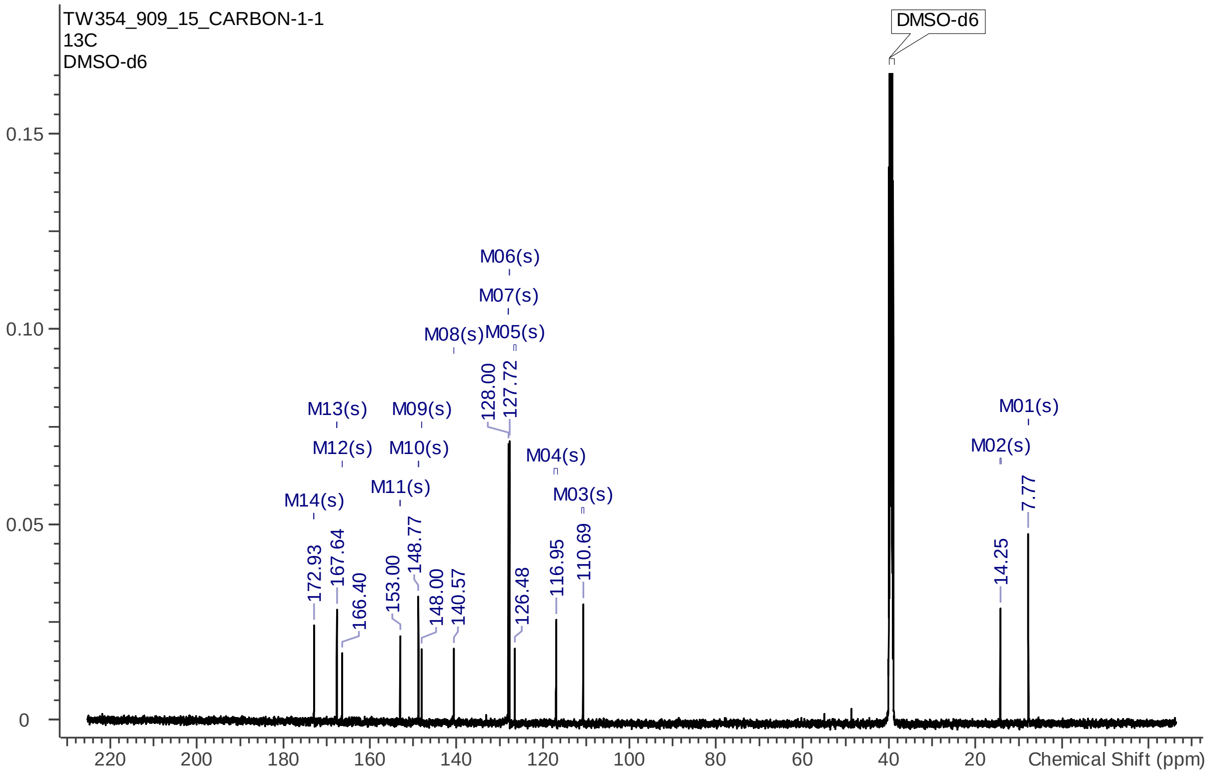
TW354_909_15_CARBON-1-1
13C
DMSO-d6
DMSO-d6
M01(s)
M02(s)
M03(s)
M04(s)
M05(s)
M06(s)
M07(s)
M08(s)
M09(s)
M10(s)
M11(s)
M12(s)
M13(s)
M14(s)
172.93
167.64
166.40
153.00
148.77
148.00
140.57
128.00
127.72
126.48
116.95
110.69
14.25
7.77
Chemical Shift (ppm)

N-(4-(3-(1,2,4-Oxadiazol-3-yl)phenyl)pyridin-2-yl)cyclopropanecarboxamide (**33**)

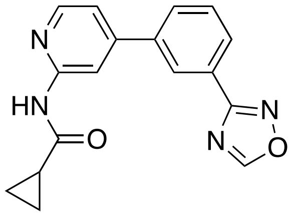

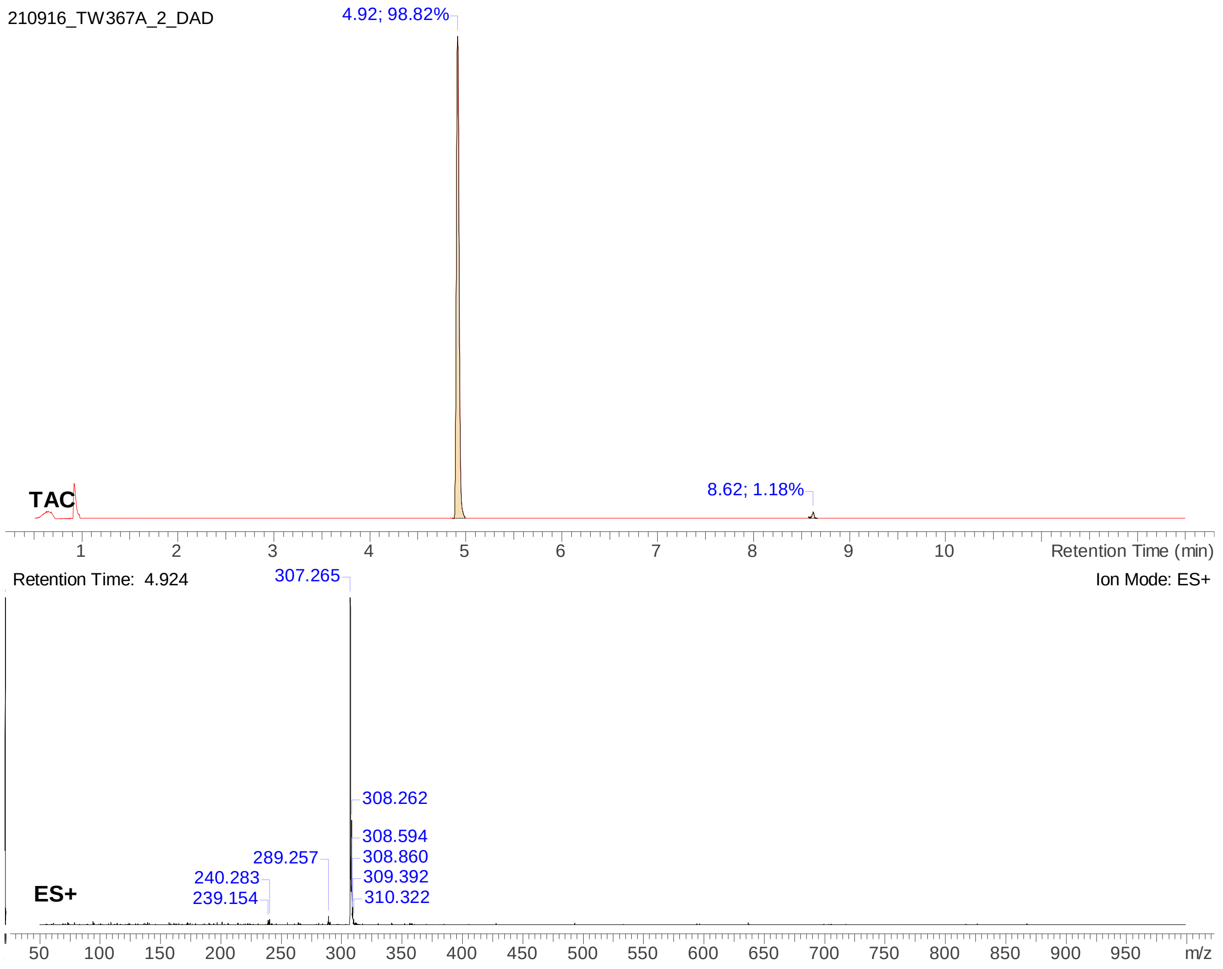

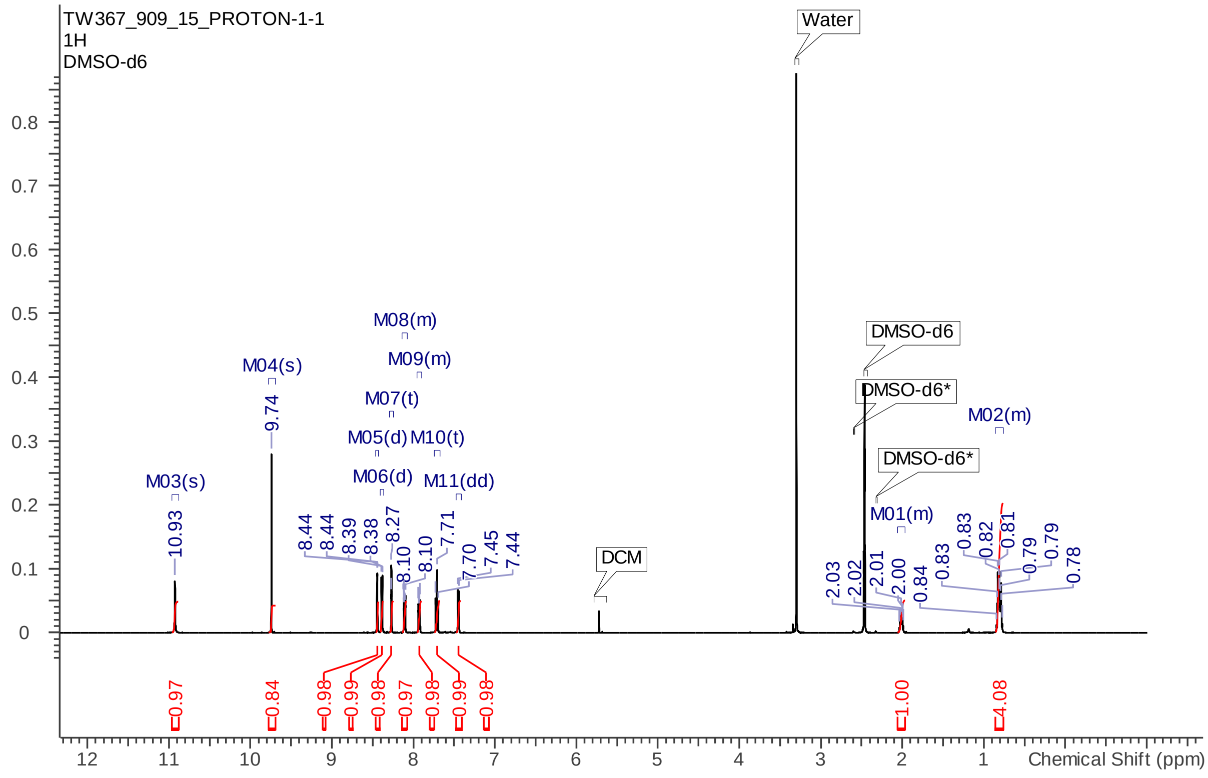
TW367_909_15_PROTON-1-1
1H
DMSO-d6
Water
DMSO-d6
DMSO-d6*
DCM
M01(m) M02(m) M03(s) M04(s) M05(d) M06(d) M07(t) M08(m) M09(m) M10(t) M11(dd)
10.93 9.74 8.44 8.44 8.39 8.38 8.27 8.10 8.10 7.71 7.70 7.45 7.44 2.03 2.02 2.01 2.00 0.84 0.83 0.83 0.82 0.81 0.79 0.79 0.78
0.97 0.84 0.98 0.99 0.98 0.97 0.98 0.99 0.98 1.00 4.08
Chemical Shift (ppm)

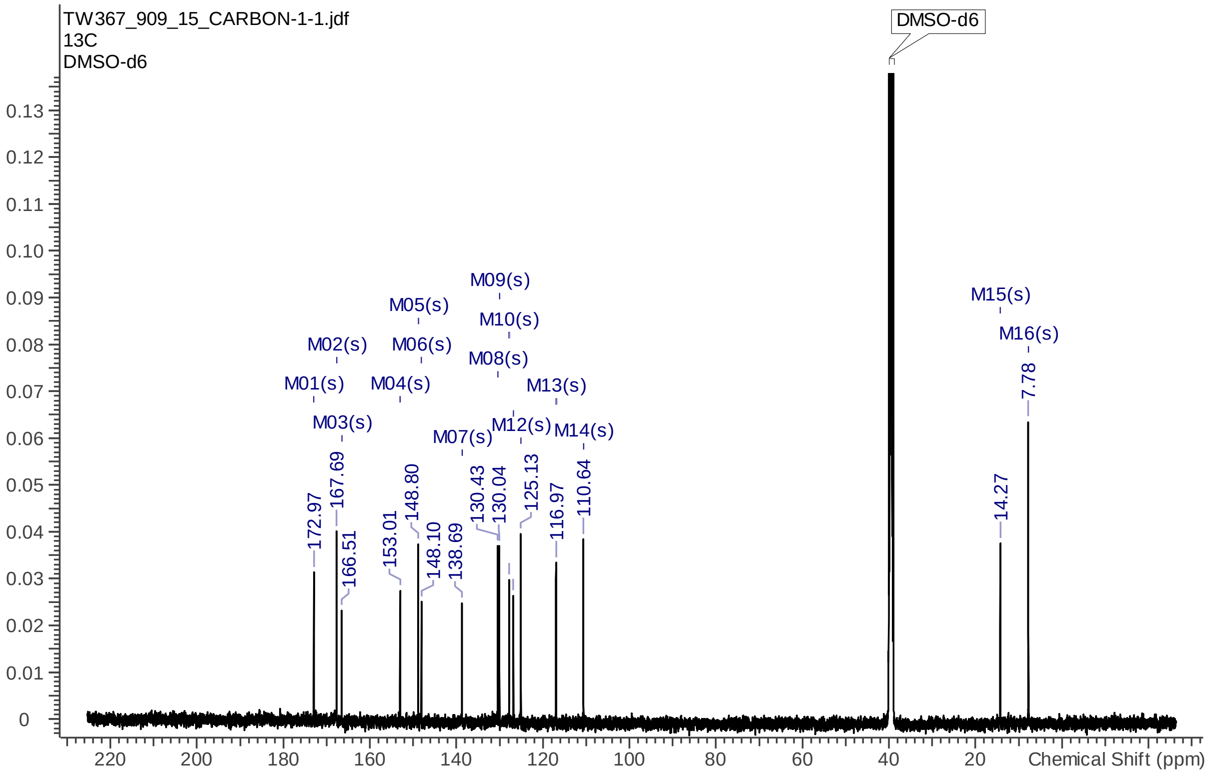
TW367_909_15_CARBON-1-1.jdf
13C
DMSO-d6
DMSO-d6
M01(s) M02(s) M03(s) M04(s) M05(s) M06(s) M07(s) M08(s) M09(s) M10(s) M12(s) M13(s) M14(s) M15(s) M16(s)
172.97 167.69 166.51 153.01 148.80 148.10 138.69 130.43 130.04 125.13 116.97 110.64 14.27 7.78
Chemical Shift (ppm)

N-(6-(1,2,4-Oxadiazol-3-yl)-[3,4'-bipyridin]-2'-yl)cyclopropanecarboxamide (**34**)

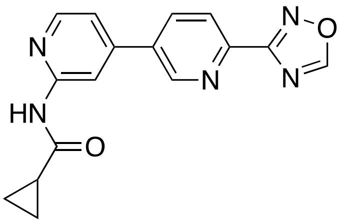

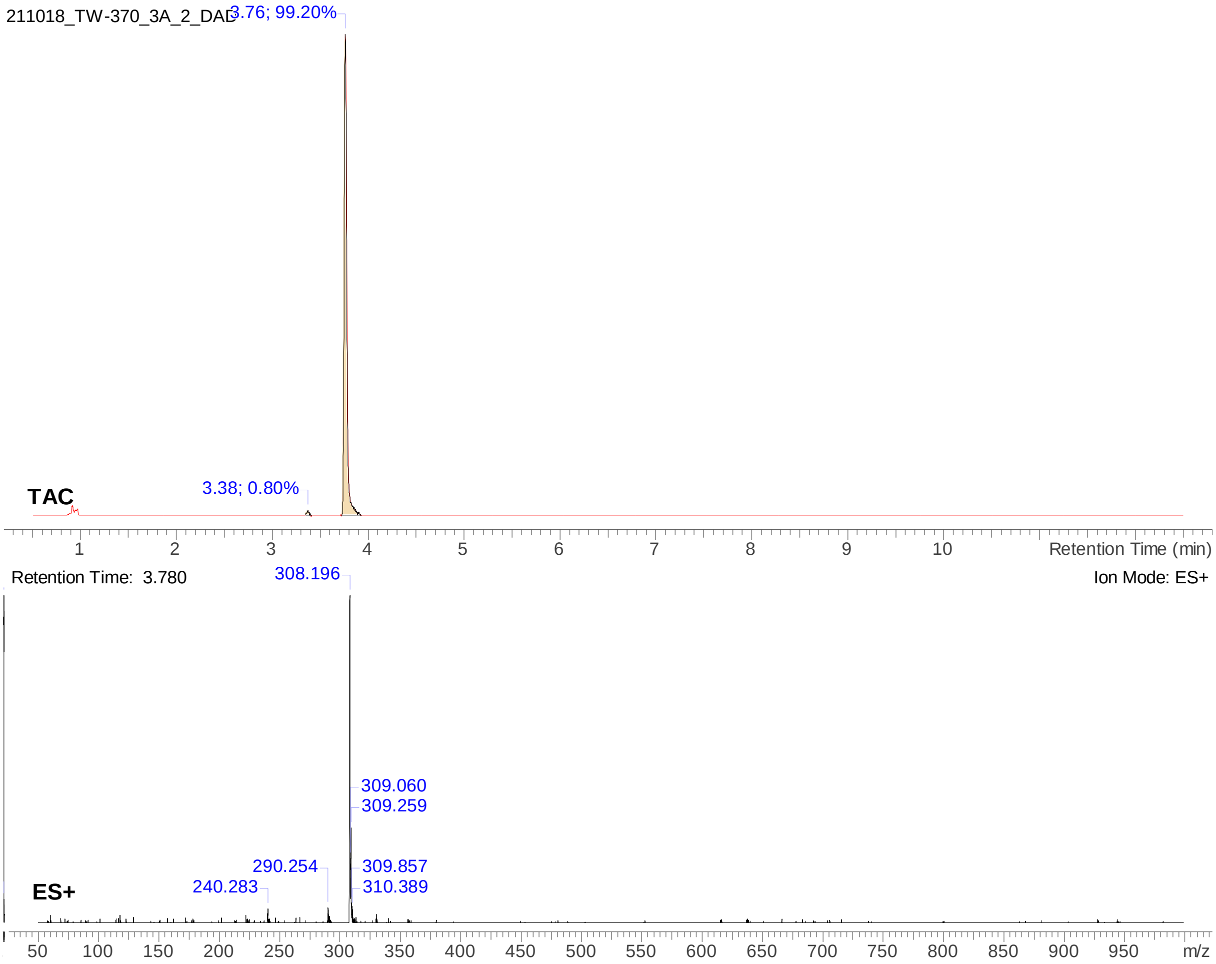

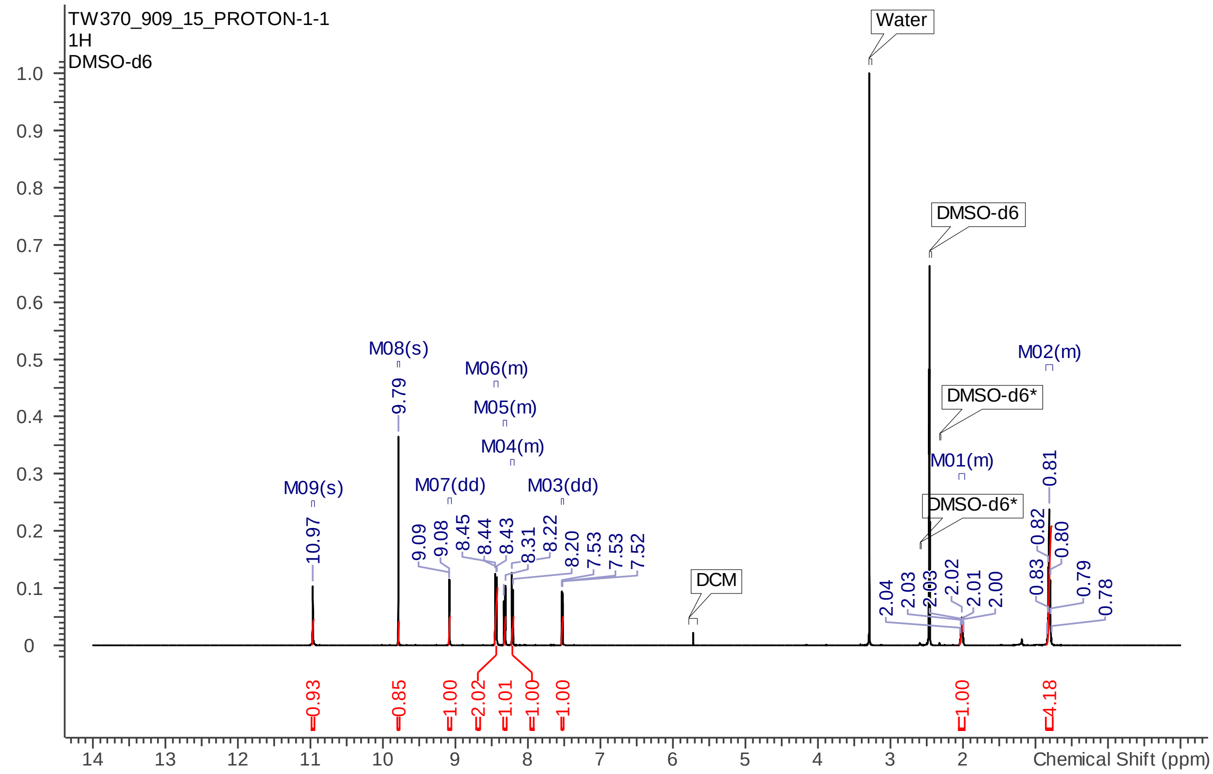
TW370_909_15_PROTON-1-1
1H
DMSO-d6
Water
DMSO-d6
DMSO-d6*
DMSO-d6*
DCM
M09(s)
M08(s)
M07(dd)
M06(m)
M05(m)
M04(m)
M03(dd)
M02(m)
M01(m)
Chemical Shift (ppm)

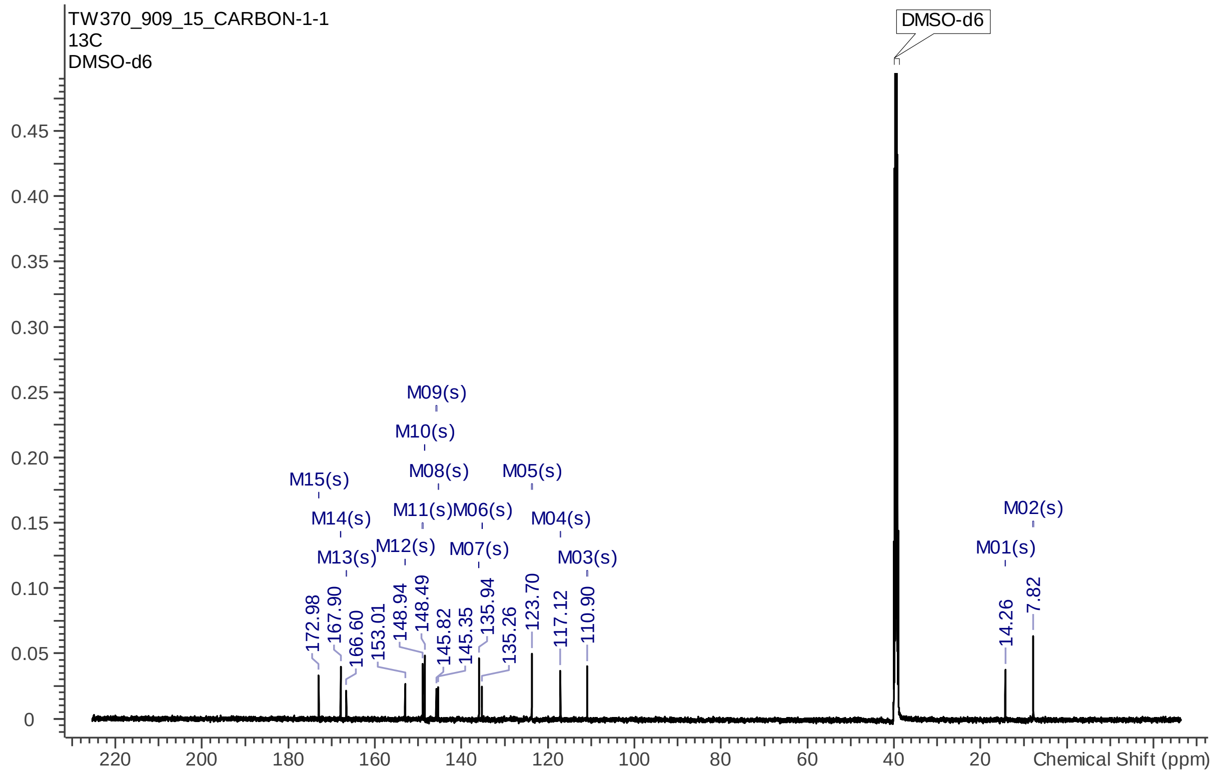
TW370_909_15_CARBON-1-1
13C
DMSO-d6
DMSO-d6
Chemical Shift (ppm)

N-(2'-(1,2,4-Oxadiazol-3-yl)-[4,4'-bipyridin]-2-yl)cyclopropanecarboxamide (**35**)

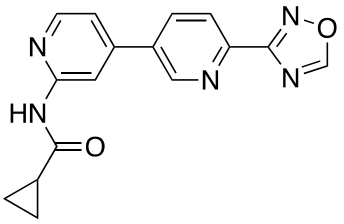

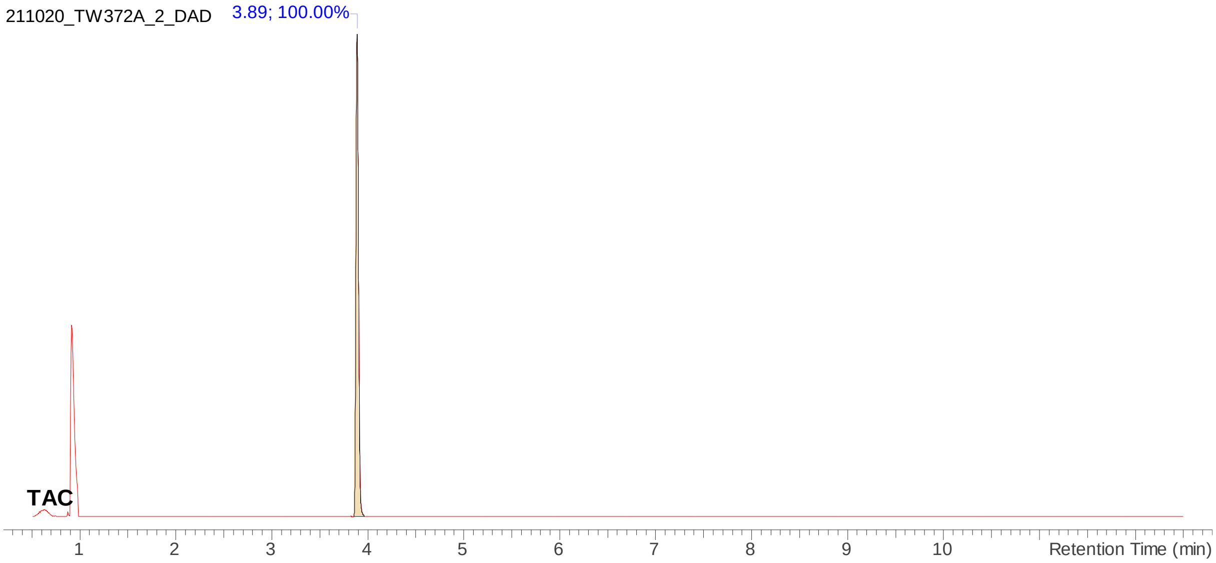

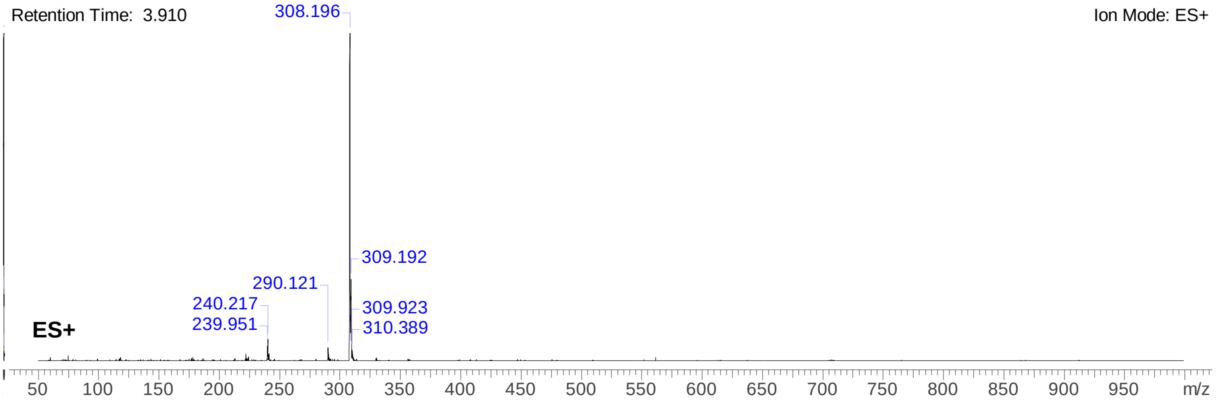

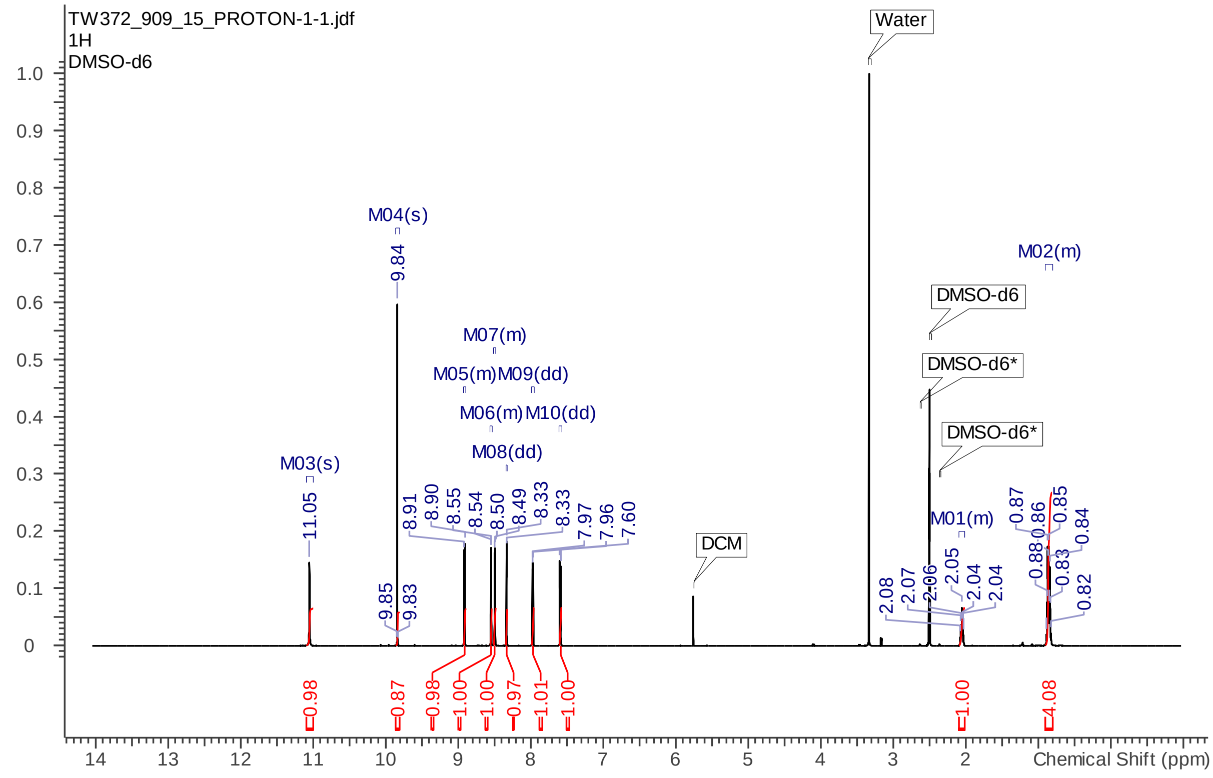
TW372_909_15_PROTON-1-1.jdf
1H
DMSO-d6
Water
DMSO-d6
DMSO-d6*
DMSO-d6*
DCM
M03(s)
M04(s)
M07(m)
M05(m)M09(dd)
M06(m)M10(dd)
M08(dd)
M01(m)
M02(m)
11.05
9.85
9.84
9.83
8.91
8.90
8.55
8.54
8.50
8.49
8.33
8.33
7.97
7.96
7.60
2.08
2.07
2.06
2.05
2.04
2.04
0.88
0.87
0.86
0.85
0.84
0.83
0.82
0.98
0.87
0.98
1.00
1.00
0.97
1.01
1.00
1.00
4.08
Chemical Shift (ppm)

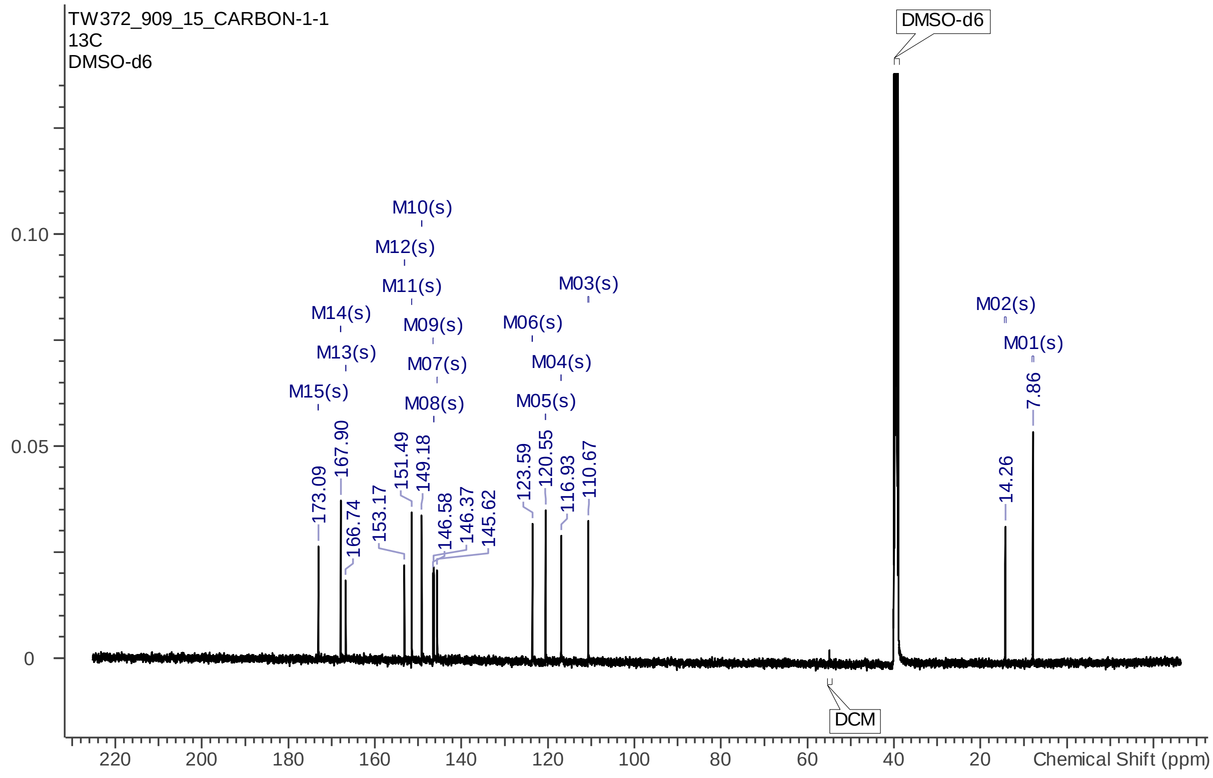
TW372_909_15_CARBON-1-1
13C
DMSO-d6
DMSO-d6
DCM
M10(s)
M12(s)
M11(s)
M14(s)
M09(s)
M13(s)
M07(s)
M15(s)
M08(s)
M06(s)
M03(s)
M04(s)
M05(s)
M02(s)
M01(s)
173.09
167.90
166.74
153.17
151.49
149.18
146.58
146.37
145.62
123.59
120.55
116.93
110.67
14.26
7.86
Chemical Shift (ppm)

N-(4-(5-(1,2,4-Oxadiazol-3-yl)thiophen-2-yl)pyridin-2-yl)cyclopropanecarboxamide (**36**)

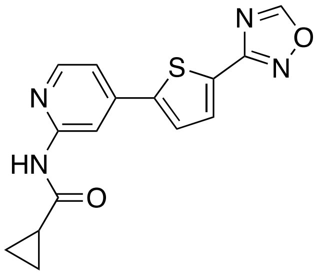

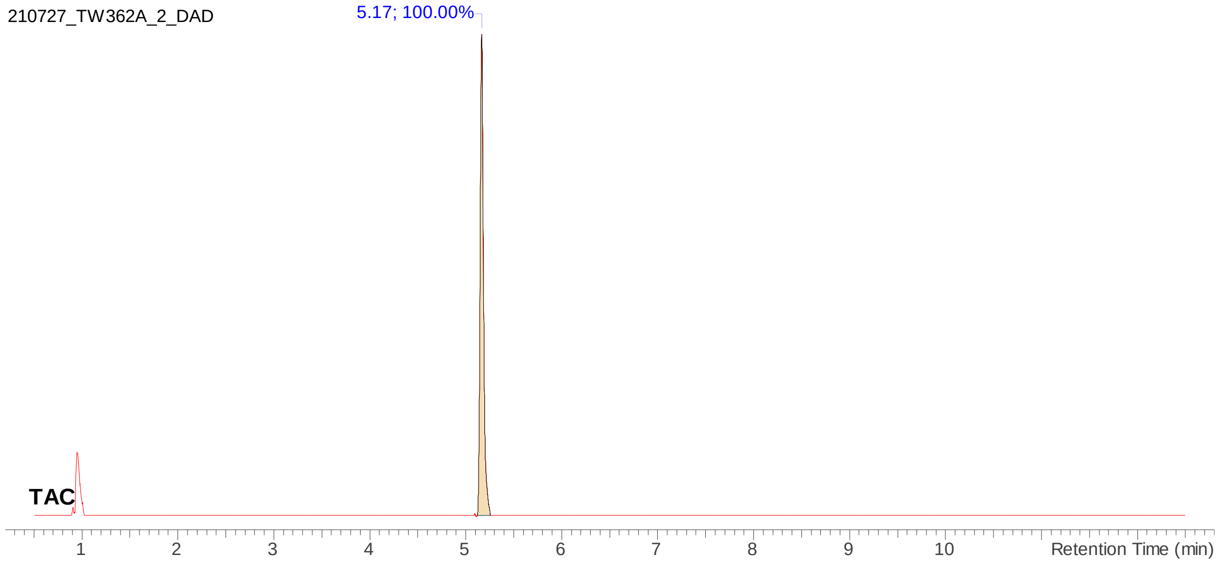

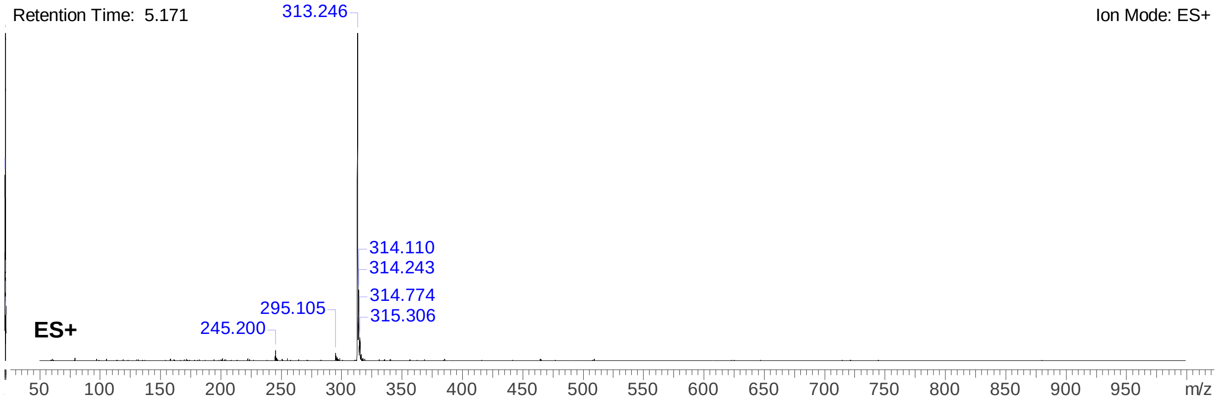

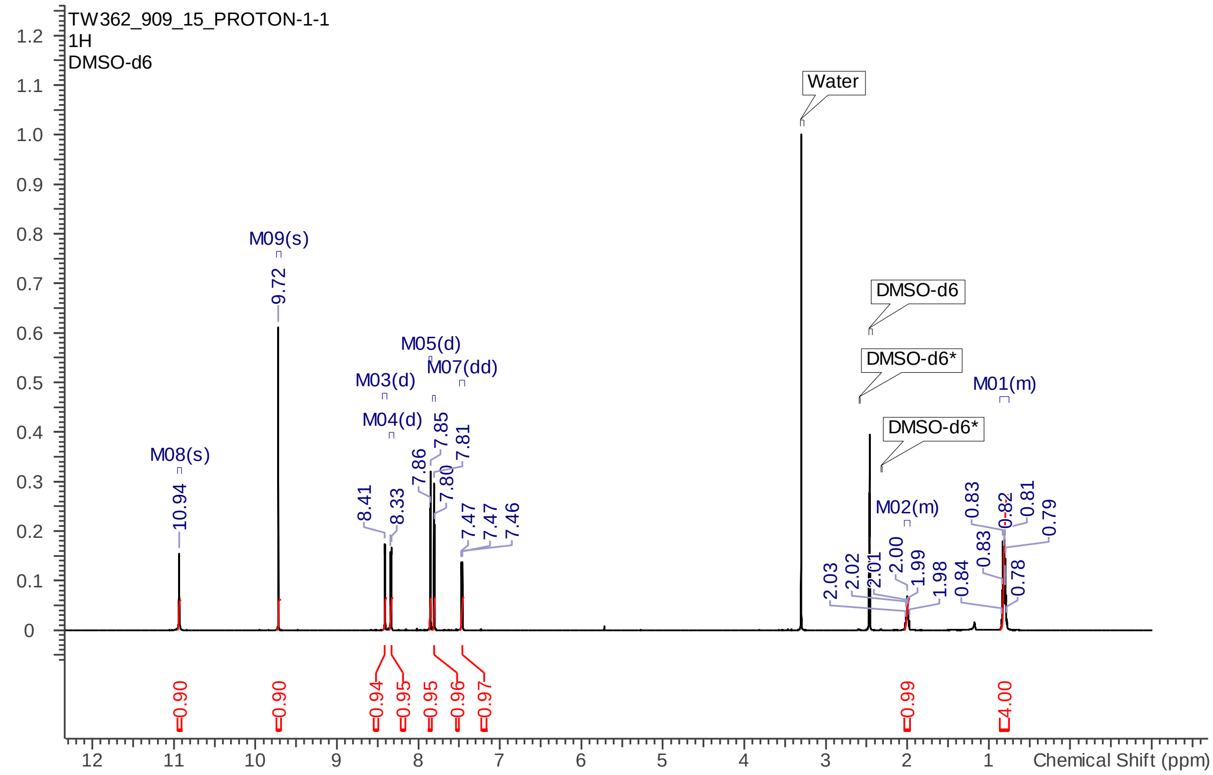
TW362_909_15_PROTON-1-1
1H
DMSO-d6
Water
DMSO-d6
DMSO-d6*
DMSO-d6*
M08(s)
10.94
M09(s)
9.72
M03(d)
M04(d)
M05(d)
M07(dd)
8.41
8.33
7.86
7.85
7.81
7.80
7.47
7.47
7.46
M02(m)
2.03
2.02
2.01
2.00
1.99
1.98
M01(m)
0.84
0.83
0.83
0.82
0.81
0.79
0.78
0.90
0.90
0.94
0.95
0.95
0.96
0.97
0.99
4.00
Chemical Shift (ppm)

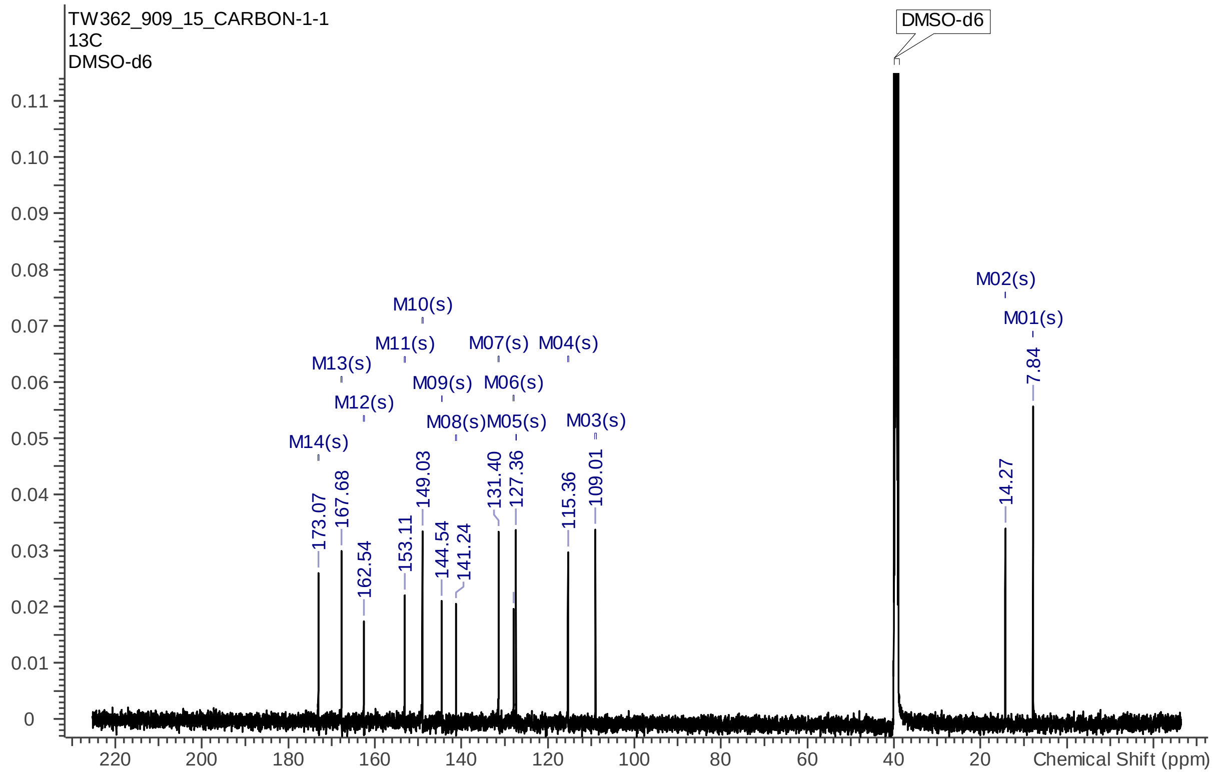
TW362_909_15_CARBON-1-1
13C
DMSO-d6
DMSO-d6
M14(s)
M13(s)
M12(s)
M11(s)
M10(s)
M09(s)
M08(s)
M07(s)
M06(s)
M05(s)
M04(s)
M03(s)
M02(s)
M01(s)
173.07
167.68
162.54
153.11
149.03
144.54
141.24
131.40
127.36
115.36
109.01
14.27
7.84
Chemical Shift (ppm)

N-(4-(5-Phenylthiophen-2-yl)pyridin-2-yl)cyclopropanecarboxamide (**37**)

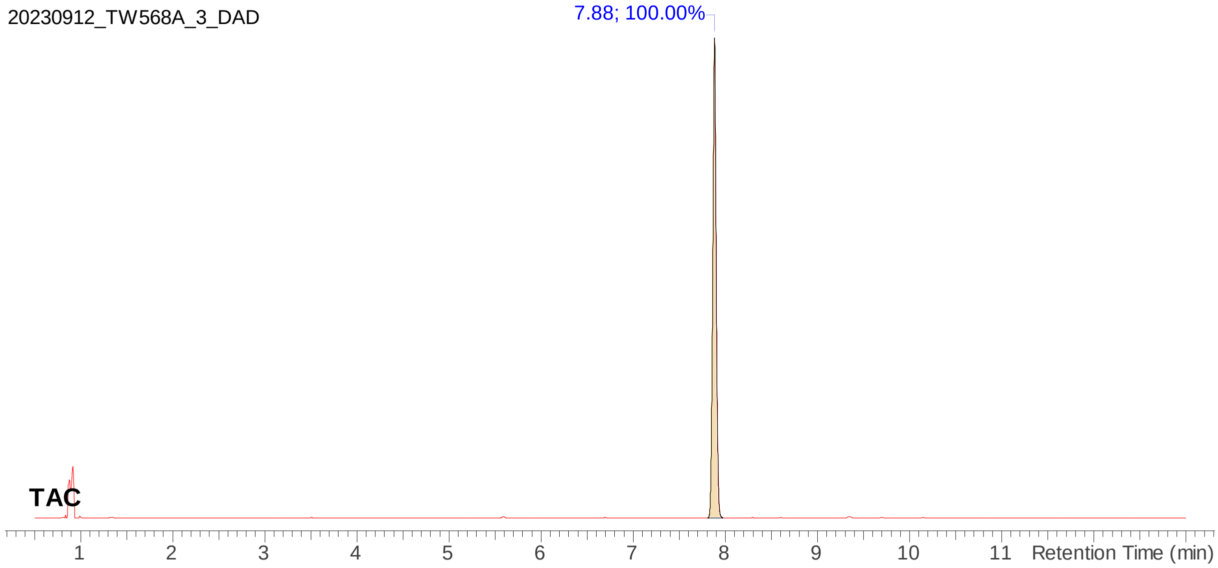

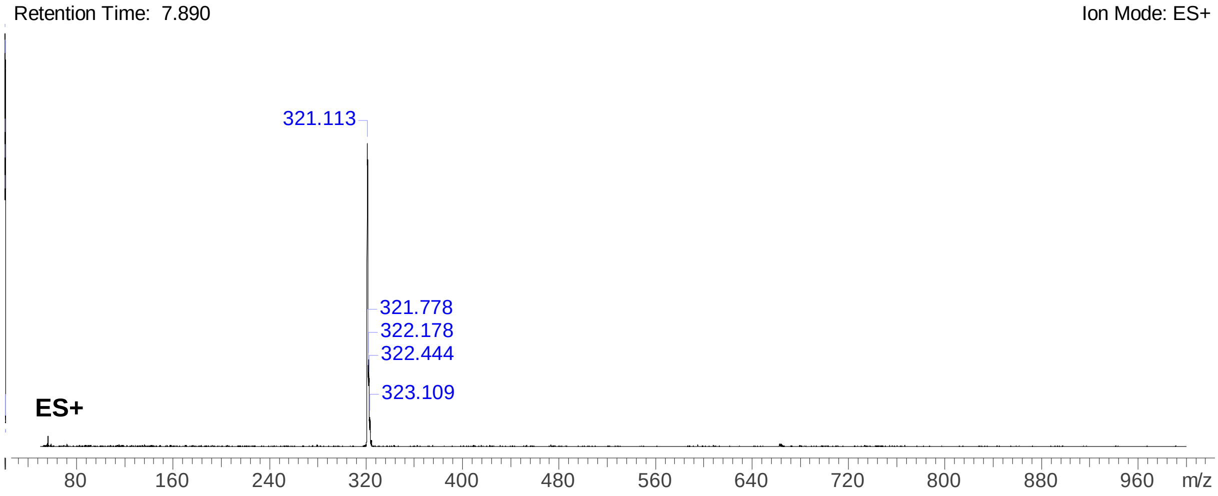

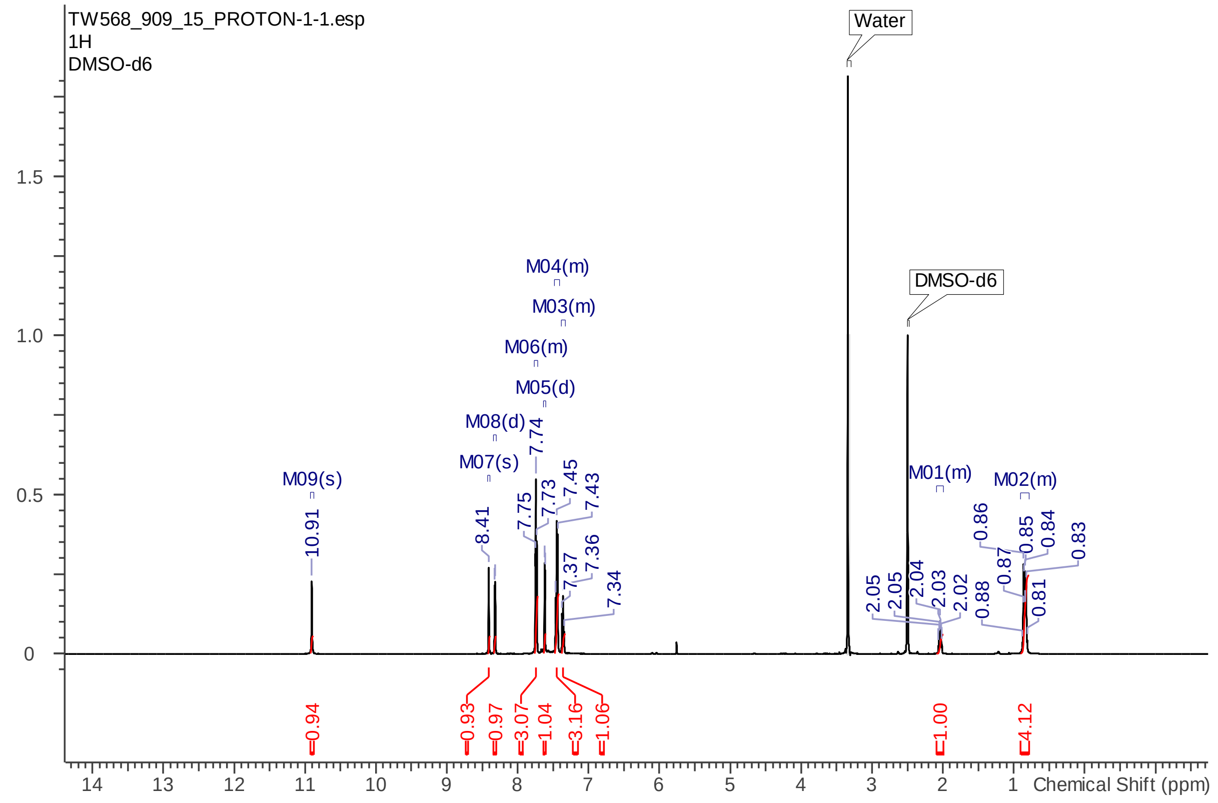
TW568_909_15_PROTON-1-1.esp
1H
DMSO-d6
Water
DMSO-d6
M09(s)
10.91
M08(d)
M07(s)
8.41
M04(m)
M03(m)
M06(m)
M05(d)
7.74
7.75
7.73
7.45
7.43
7.37
7.36
7.34
M01(m)
M02(m)
2.05
2.05
2.04
2.03
2.02
0.86
0.88
0.87
0.85
0.84
0.83
0.81
0.94
0.93
0.97
3.07
1.04
3.16
1.06
1.00
4.12
Chemical Shift (ppm)

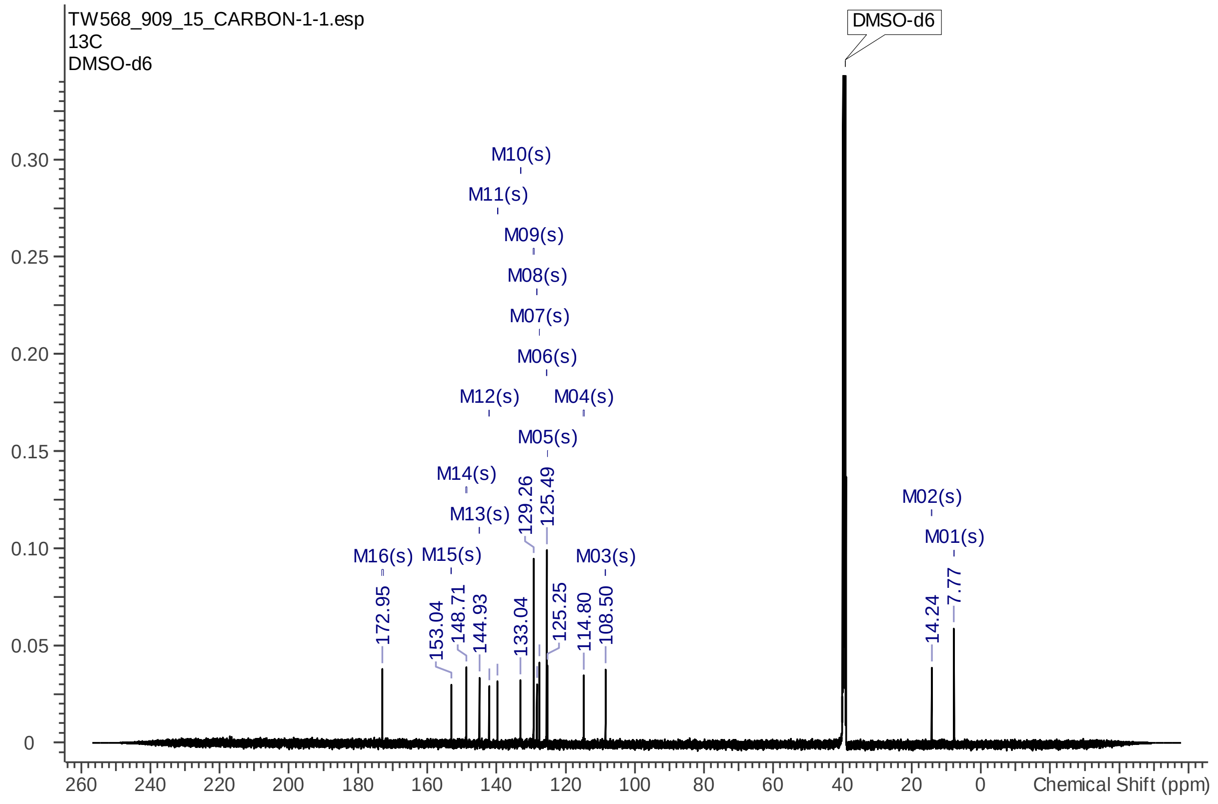
TW568_909_15_CARBON-1-1.esp
13C
DMSO-d6
DMSO-d6
M10(s)
M11(s)
M09(s)
M08(s)
M07(s)
M06(s)
M12(s)
M04(s)
M05(s)
M14(s)
M13(s)
M16(s)
M15(s)
M03(s)
M02(s)
M01(s)
172.95
153.04
148.71
144.93
133.04
129.26
125.49
125.25
114.80
108.50
14.24
7.77
Chemical Shift (ppm)

References:

1. Gundelach, L.; Fox, T.; Tautermann, C.S.; Skylaris, C.K. BRD4: Quantum Mechanical Protein-Ligand Binding Free Energies Using the Full-Protein DFT-Based QM-PBSA Method. *Phys. Chem. Chem. Phys.* **2022**, *24*, 25240–25249, doi:10.1039/d2cp03705j.

2. Kumari, S.; Carmona, A. V.; Tiwari, A.K.; Trippier, P.C. Amide Bond Bioisosteres: Strategies, Synthesis, and Successes. *J. Med. Chem.* **2020**, *63*, 12290–12358, doi:10.1021/acs.jmedchem.0c00530.

3. Groom, C.R.; Bruno, I.J.; Lightfoot, M.P.; Ward, S.C. The Cambridge Structural Database. *Acta Crystallogr. Sect. B Struct. Sci. Cryst. Eng. Mater.* **2016**, *72*, 171–179, doi:10.1107/S2052520616003954.

4. Ranasinghe, K.; Marzilli, P.A.; Pakhomova, S.; Marzilli, L.G. Linear Bidentate Ligands (L) with Two Terminal Pyridyl N-Donor Groups Forming Pt(II)LCl2 Complexes with Rare Eight-Membered Chelate Rings. *Inorg. Chem.* **2018**, *57*, 12756–12768, doi:10.1021/acs.inorgchem.8b01943.

5. Lehtola, S.; Steigemann, C.; Oliveira, M.J.T.; Marques, M.A.L. Recent Developments in LIBXC — A Comprehensive Library of Functionals for Density Functional Theory. *SoftwareX* **2018**, *7*, 1–5, doi:10.1016/j.softx.2017.11.002.

6. Neese, F. Software Update: The ORCA Program System—Version 5.0. *Wiley Interdiscip. Rev. Comput. Mol. Sci.* **2022**, *12*, 1–15, doi:10.1002/wcms.1606.

7. Valeev, E.F. Libint - a Library for the Evaluation of Molecular Integrals of Many-Body Operators over Gaussian Functions, Version 2. Available online: http://libint.valeyev.net/ (accessed on 30 December 2022).
